# Construction of pyrroles, furans and thiophenes *via* intramolecular cascade desulfonylative/dehydrogenative cyclization of vinylidenecyclopropanes induced by NXS (X = I or Br)†

**DOI:** 10.1039/d3sc01542d

**Published:** 2023-06-13

**Authors:** Zhe Meng, Jun Yan, Chao Ning, Min Shi, Yin Wei

**Affiliations:** a Key Laboratory for Advanced Materials and Institute of Fine Chemicals, School of Chemistry & Molecular Engineering, East China University of Science and Technology 130 Meilong Road Shanghai 200237 China; b State Key Laboratory of Organometallic Chemistry, Center for Excellence in Molecular Synthesis, Shanghai Institute of Organic Chemistry, University of Chinese Academy of Sciences, Chinese Academy of Sciences 345 Lingling Road Shanghai 200032 China mshi@mail.sioc.ac.cn weiyin@sioc.ac.cn

## Abstract

Pyrroles, furans, and thiophenes are important structural motifs in biologically active substances, pharmaceuticals and functional materials. In this paper, we disclose an efficient synthetic strategy for the rapid construction of multisubstituted pyrroles, furans, and thiophenes *via* NXS mediated desulfonylative/dehydrogenative cyclization of vinylidenecyclopropanes (VDCPs). The advantages of this method include wide substrate range, high efficiency and synthetic usefulness of the heterocyclic products under metal-free and mild conditions. The derivatization of pyrrole products and the preparation of functional molecules successfully demonstrated the synthetic potential of the products as platform molecules. The reaction mechanism has been investigated on the basis of control experiments and DFT calculations.

## Abstract 2

Pyrroles, furans, and thiophenes were efficiently obtained by NXS mediated desulfonylative/dehydrogenative cyclization of vinylidenecyclopropanes within 0.5 h. The reaction mechanism was investigated by control experiments and DFT calculations.

## Graphical abstract

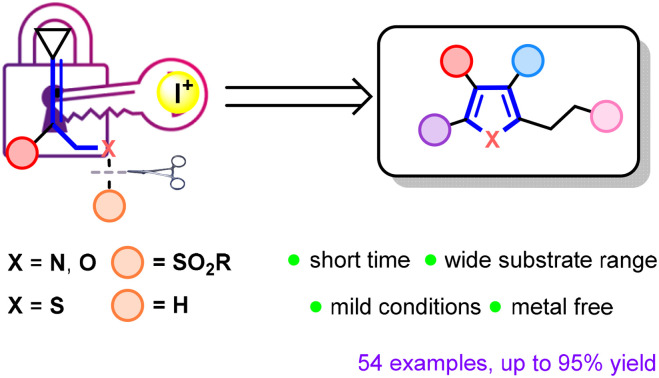

## Introduction

**Scheme 1:**
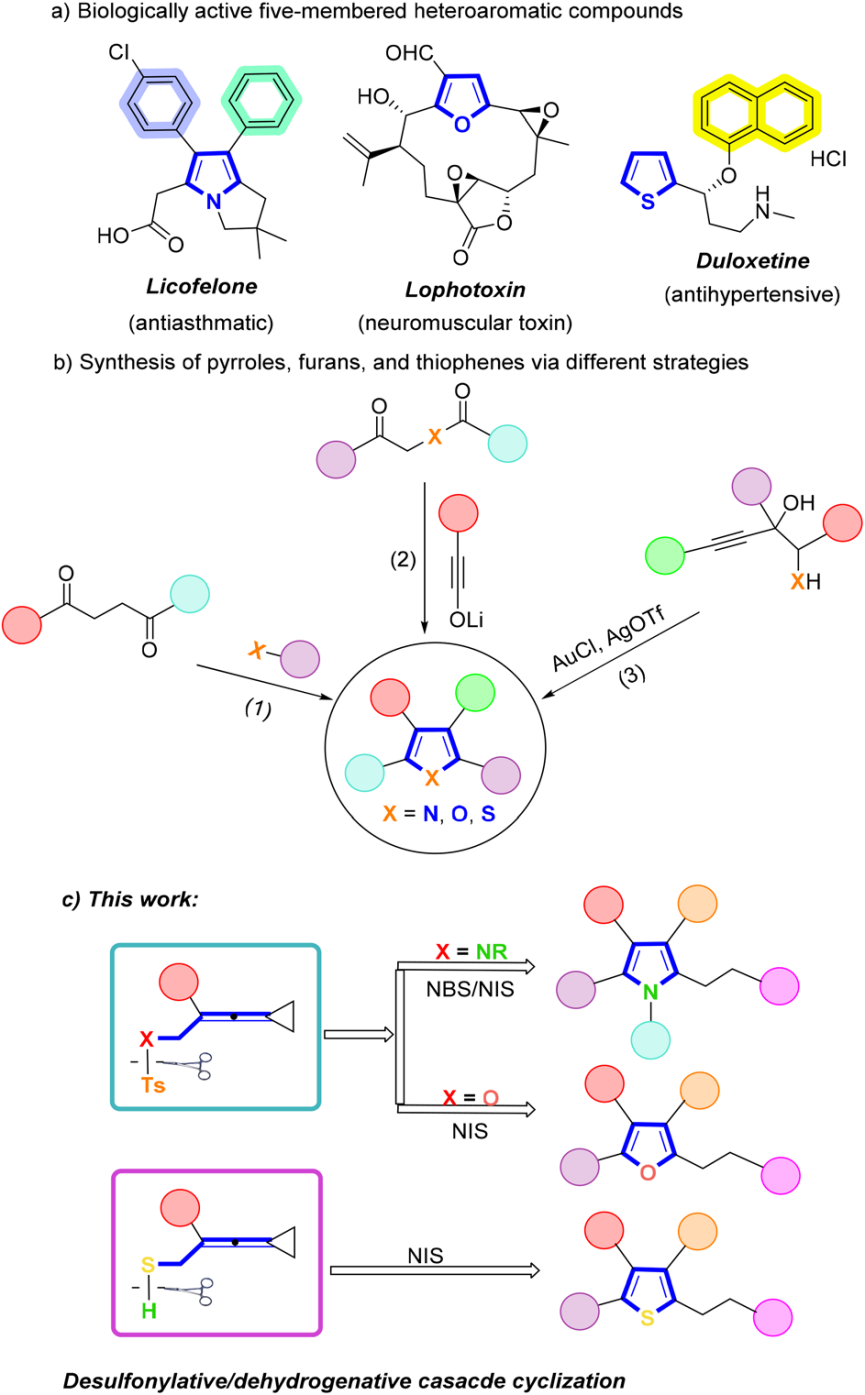
(a) Representative biologically active five-membered heteroaromatic compounds; (b) previous reports on pyrroles, furans and thiophenes synthesis; (c) this work.

Five-membered heterocycles including pyrroles, furans and thiophenes are widely found in a variety of bioactive molecules, functional and polymeric materials.^1^ Moreover, they are also core structural motifs in many natural products and pharmaceuticals. For example, the pyrrole fragment is the key structural unit in licofelone, which possesses significant analgesic, anti-inflammatory, and antiasthmatic activities.^1*b*^ Lophotoxin is a novel neuromuscular toxin that has been isolated from several Pacific gorgonians and contains a furan fragment in its molecular structure.^1*e*^ In addition, thiophene fragment exists in a commercial drug known as duloxetine, which possesses effective antihypertensive activity (Scheme 1a). The synthetic methods of these heterocyclic compounds have been widely reported and are of great concern.^2^ To date, the traditional synthetic methods for these heterocycles, including Paal–Knorr condensation,^3^ Hantzsch reaction,^4^ Feist–Benary reaction,^5^ Hinsberg reaction,^6^ transition metal-catalyzed cyclizations,^7^ and photochemical reactions,^8^ have been developed for many years. However, the above reactions frequently employ different synthetic protocols to obtain the starting substrates depending on the required heteroatoms such as N, O, and S. Thus, the development of new strategies that facilitate rapid construction of each of these heterocycles by a single method and provide functionalized pyrroles, furans, and thiophenes in a one-pot manner would be extremely useful and highly desirable. Nonetheless, thus far, only a few methods to access each of these heterocycles by changing the heteroatoms of substrates have been developed (Scheme 1b).^9^ For example, Shishido's group reported an efficient strategy for the synthesis of pyrroles, furans and thiophenes utilizing ynolates.^9*a*^ In addition, Marques's group developed the preparation of pyrroles, furans and thiophenes by the gold-catalyzed dehydration and cyclization of heteroatom-substituted propargylic alcohols.^9*b*^ Nevertheless, these synthetic methods usually need harsh reaction conditions, metal catalysts, and prolonged reaction time.

In view of the aforementioned research circumstances, we attempted to introduce heteroatoms into vinylidenecyclopropanes (VDCPs)^10^ and assumed that these heteroatomic VDCPs could be excellent candidates for exploiting new methods in cyclization for the rapid construction of heteroaromatics due to their multiple reaction sites. Herein, we describe an intramolecular desulfonylative/dehydrogenative cyclization reaction that proceeds effectively under mild and metal-free conditions, providing functionalized pyrroles, furans, and thiophenes in good yields (Scheme 1c).

## Results and discussion

We began our investigation by exploring the model reaction between vinylidenecyclopropane (VDCP) 1r (0.2 mmol, 1.0 equiv.) and *N*-iodosuccinimide (NIS) 2a (0.7 mmol, 3.5 equiv.), pyridine (Py, 0.5 equiv.) as an organic base and TBAI (0.2 mmol, 1.0 equiv.) as an additive in dichloromethane (DCM) (2.0 mL) at room temperature in an ambient atmosphere for 30 min, affording the desired product 3ra in 95% NMR yield and 93% isolated yield (Table 1, entry 1). The optimization of the reaction conditions indicated that there was a significant decrease in reaction efficiency when TBAI was not added into the reaction mixture (Table 1, entry 2). In addition, we also optimized the reaction conditions in the absence of TBAI to obtain 3ra in 76% yield (see Table S1 in the ESI† for more details). An investigation of base effect revealed that DMAP and Cs_2_CO_3_ turned out to be less efficient (Table 1, entries 3 and 4) (see Table S2 in the ESI† for more details). The screening of solvent effect demonstrated that DCM was more suitable than others such as toluene, DMF and MeCN (Table 1, entries 5–7) (see Table S3 in the ESI† for more information). However, by increasing the employed amount of pyridine to 2.0 equiv. or without the addition of pyridine, the reaction efficiency was reduced (Table 1, entries 8 and 9) (see Table S4 in the ESI† for more information). Moreover, we observed that on prolonging the reaction time to 1.0 h or 12.0 h, the yield of 3ra decreased to 82% and 50%, respectively, indicating that 3ra was labile in the reaction system (Table 1, entries 10 and 11) (see Table S5 in the ESI† for more information). The use of other sources of iodine reagents, such as IPy_2_BF_4_ and I_2_, afforded 3ra in 43% and 15% yields, respectively (Table 1, entries 12 and 13). It was worthy to note that NBS could also be used to react with 1r, giving brominated pyrrole in 44% yield (Table 1, entry 14). However, when NCS was utilized in the reaction, none of the desired products was produced (Table 1, entry 15).

**Table 1:** Optimization of reaction conditions^a^

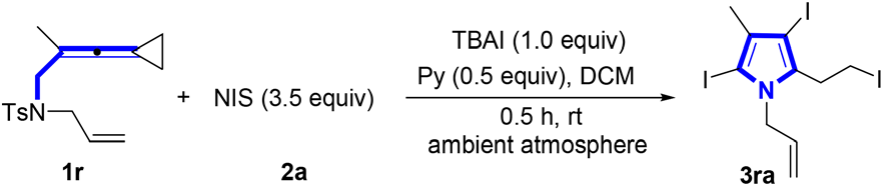		
Entry^a^	Variation from the standard condition	3ra, yield^b^ [%]
1	None	95 (93)^c^
2	Without TBAI	76
3	DMAP instead of Py	70
4	Cs_2_CO_3_ instead of Py	60
5	Toluene instead of DCM	55
6	DMF instead of DCM	29
7	MeCN instead of DCM	85
8	Py (2.0 equiv.) instead of Py (0.5 equiv.)	55
9	Py (0 equiv.) instead of Py (0.5 equiv.)	40
10	1.0 h instead of 0.5 h	82
11	12.0 h instead of 0.5 h	50
12	IPy_2_BF_4_ instead of NIS	43
13	I_2_ instead of NIS	15
14	NBS instead of NIS	44^d^
15	NCS instead of NIS	0

a The reaction was carried out with 1r (0.2 mmol, 1.0 equiv.), 2a (3.5 equiv.) in the presence of an organic or inorganic base and the solvent (2.0 mL).

b ^1^H NMR yield using 1,3,5-trimethoxybenzene as an internal standard.

c Isolated yield.

d Brominated pyrrole.

**Scheme 2:**
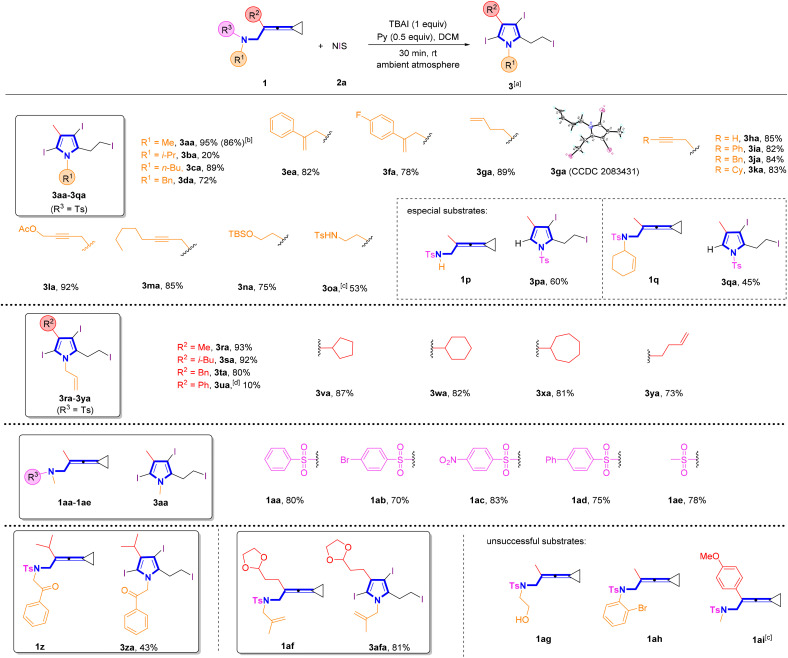
(a) Standard conditions: substrate 1 (0.2 mmol, 1.0 equiv.), 2a (3.5 equiv.), TBAI (1.0 equiv.) and pyridine (0.5 equiv.) in DCM (2 mL), ambient atmosphere, 30 min, rt. (b) Gram scale reaction, 1a (2.0 mmol, 0.55 g), 2a (7.0 mmol, 1.58 g), TBAI (2.0 mmol), pyridine (0.8 mL), DCM (10 mL). (c) A byproduct 3oa′ was afforded in 45% yield (see Fig. S5 on page S182 in the ESI† for more information). (d) 80 °C, 12 h.

With the optimal reaction conditions established, we next explored the substrate scope for the synthesis of pyrroles (Scheme 2). It was found that most of the substrates successfully underwent these reactions, providing the desired products in good to excellent yields. Utilizing VDCP substrates 1a–1d (R^1^ = alkyl group, R^2^ = Me), the desired products 3aa–3da were obtained in 20–95% yields. It is worth noting that increase of the steric hindrance of the alkyl group decreased the yield of 3 such as substrate 1b bearing an isopropyl group. A gram-scale reaction was conducted by employing 0.55 g (2.0 mmol) of 1a and NIS (3.5 mmol, 1.58 g), delivering the desired product 3aa in 86% yield (0.86 g) under the standard conditions. Substrates 1e–1m (R^2^ = Me) having an alkenyl or an alkynyl unit in R^1^ were all tolerated in this reaction, delivering the corresponding products 3ea–3ma in 78–92% yields. The structure of 3ga was unambiguously determined by X-ray crystallographic analysis and its ORTEP drawing is shown in Scheme 2. Notably, substrate 1n having a protected hydroxy group and substrate 1o containing a protected amino group were both well compatible, giving the desired products 3na and 3oa in 53% and 75% yields, respectively. However, for substrate 1p having a TsNH moiety, pyrrole 3pa with the tosyl group was afforded presumably due to the easier departure of H^+^ than that of the tosyl group. In addition, as for the cyclohexene-substituted substrate 1q, the pyrrole product 3qa with the sulfonyl group was also obtained in 45% yield perhaps because during the reaction, the cyclohexenyl group connected with the NT unit was nucleophilically attacked by I^−^, causing the release of this functional group. It was noteworthy that the N atom in products 3pa and 3qa was connected with the electron-withdrawing Ts group to reduce the activity of pyrrole, resulting in the inability to undergo further halogenation reaction under the standard conditions. Next, we shifted our attention to examine the R^2^ group in VDCPs 1 (R^1^ = allyl) and found that introducing alkyl, cycloalkyl and alkenyl substituents in the R^2^ group of VDCPs 1r–1t and 1v–1y gave the desired products 3ra–3ta and 3va–3ya in 73–93% yields. When 1u was used as a substrate under room temperature conditions, only a small amount of product spots was observed on the TLC plate. The desired product 3ua was furnished in 10% yield, when the reaction was carried out at 80 °C, presumably due to the electronic effect. Moreover, the removable R^3^ sulfonyl group was also exploited under the standard conditions and we identified that substrates 1aa–1ae with a variety of sulfonated groups in R^3^ all provided the desired pyrrole in good yields. Further investigation revealed that this reaction also tolerated carbonyl group and acetal group containing VDCPs, giving the corresponding products 3za and 3afa in 43% and 81% yields, respectively. However, substrate 1ag with an unprotected hydroxy group could not deliver the desired product 3aga. The hydroxy group in 1ag was involved in the reaction and the desired pyrrole product was not obtained. Moreover, we observed that the reactions did not proceed when 1ah and 1ai were employed as substrates (see Fig. S1on page S14 in the ESI†).

**Scheme 3:**
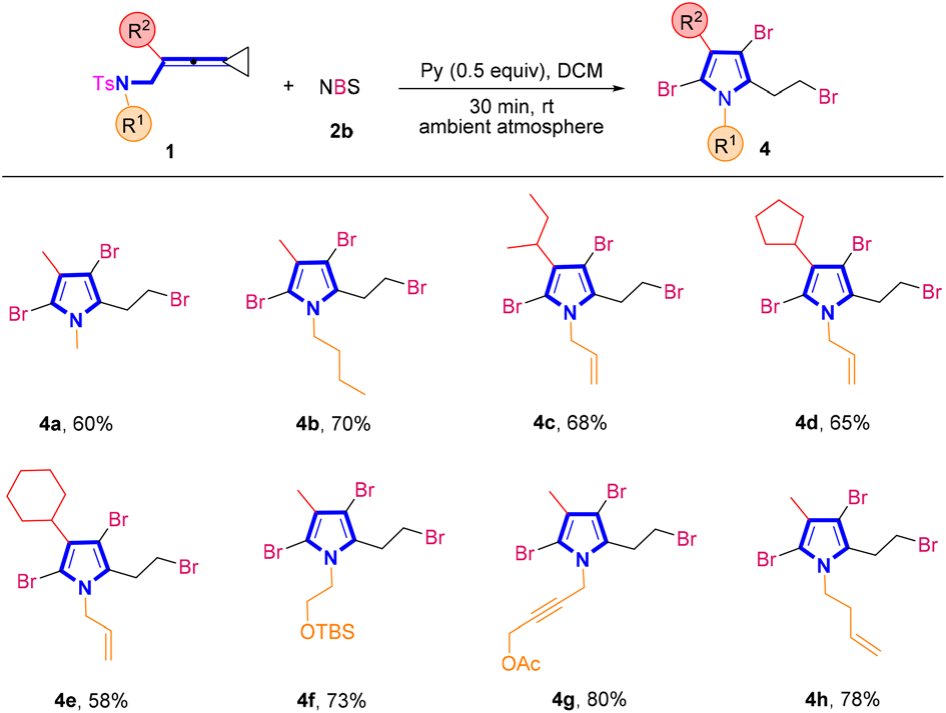
Standard conditions: substrate 1 (0.2 mmol, 1.0 equiv.), 2b (3.5 equiv.) and pyridine (0.5 equiv.) in DCM (2 mL), ambient atmosphere, 30 min, rt.

In order to further demonstrate the generality of this synthetic strategy, we then turned our attention to examining the substrate scope of brominated pyrroles 4 in the presence of NBS 2b (Scheme 3). Similarly, using VDCPs 1 as the substrates, the desired brominated pyrrole products 4a–4h were produced in moderate to good yields ranging from 58% to 80%. In VDCPs 1, R^1^ could be an alkyl, an allyl, an alkyl group bearing a protected hydroxy group, a propargyl group or a homoallyl group and R^2^ could be an alkyl or a cycloalkyl group, smoothly delivering the desired products 4 under the standard conditions.

**Scheme 4:**
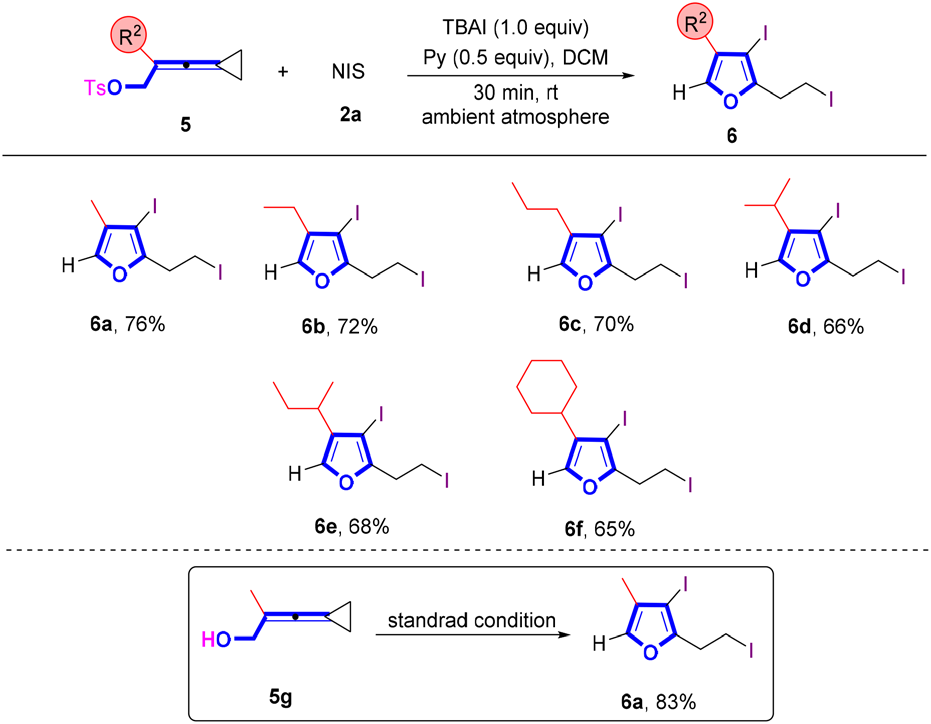
Standard conditions: substrate 5 (0.2 mmol, 1.0 equiv.), 2a (3.5 equiv.) and pyridine (0.5 equiv.) in DCM (2 mL), ambient atmosphere, 30 min, rt.

When VDCPs 5, in which the VDCP moiety is connected with a tosylated oxygen atom, were utilized as substrates to react with NIS under the standard conditions, the corresponding iodinated furan products 6 were obtained in moderate yields (Scheme 4). A variety of VDCPs 5a–5f bearing an alkyl or a cycloalkyl group were tolerated, giving furans 6a–6f in 65–76% yields. Substrate 5g, replacing the OT moiety with the hydroxy group, could also afford the desired product 6a in 83% yield.

**Scheme 5:**
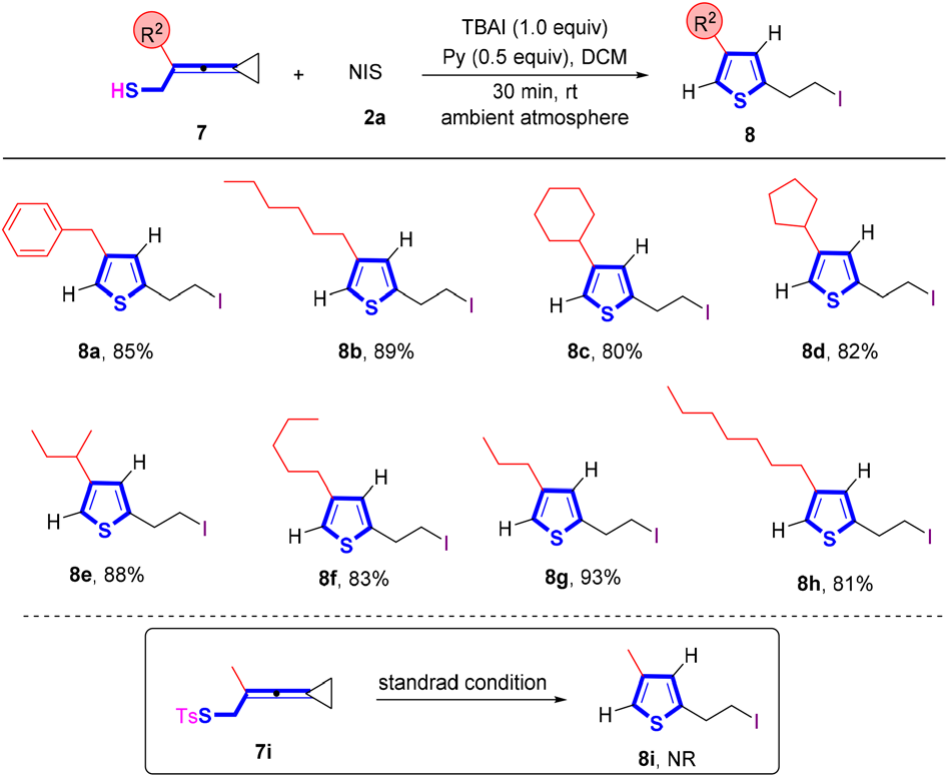
Standard conditions: substrate 7 (0.2 mmol, 1.0 equiv.), 2a (1.0 equiv.) and pyridine (0.5 equiv.) in DCM (2 mL), ambient atmosphere, 30 min, rt.

To synthesize thiophene derivatives, we further prepared VDCP 7i, in which the VDCP moiety is connected with a tosylated sulfur atom (Scheme 5). However, no reaction occurred and the starting materials were fully recovered upon conducting the reaction under the standard protocol, probably because the nucleophilicity of the sulfur atom was weakened by the tosyl group and its steric effect hampered the cyclization. Afterwards, we utilized VDCP substrates 7 having a free thiol group to conduct the reaction and found that the cyclization smoothly took place, affording the desired thiophenes 8 in good yields. As shown in Scheme 5, R^2^ substituents as a broad range of alkyl or cycloalkyl groups were all suitable for this reaction, affording the corresponding thiophenes 8a–8h in 80–93% yields.

**Scheme 6:**
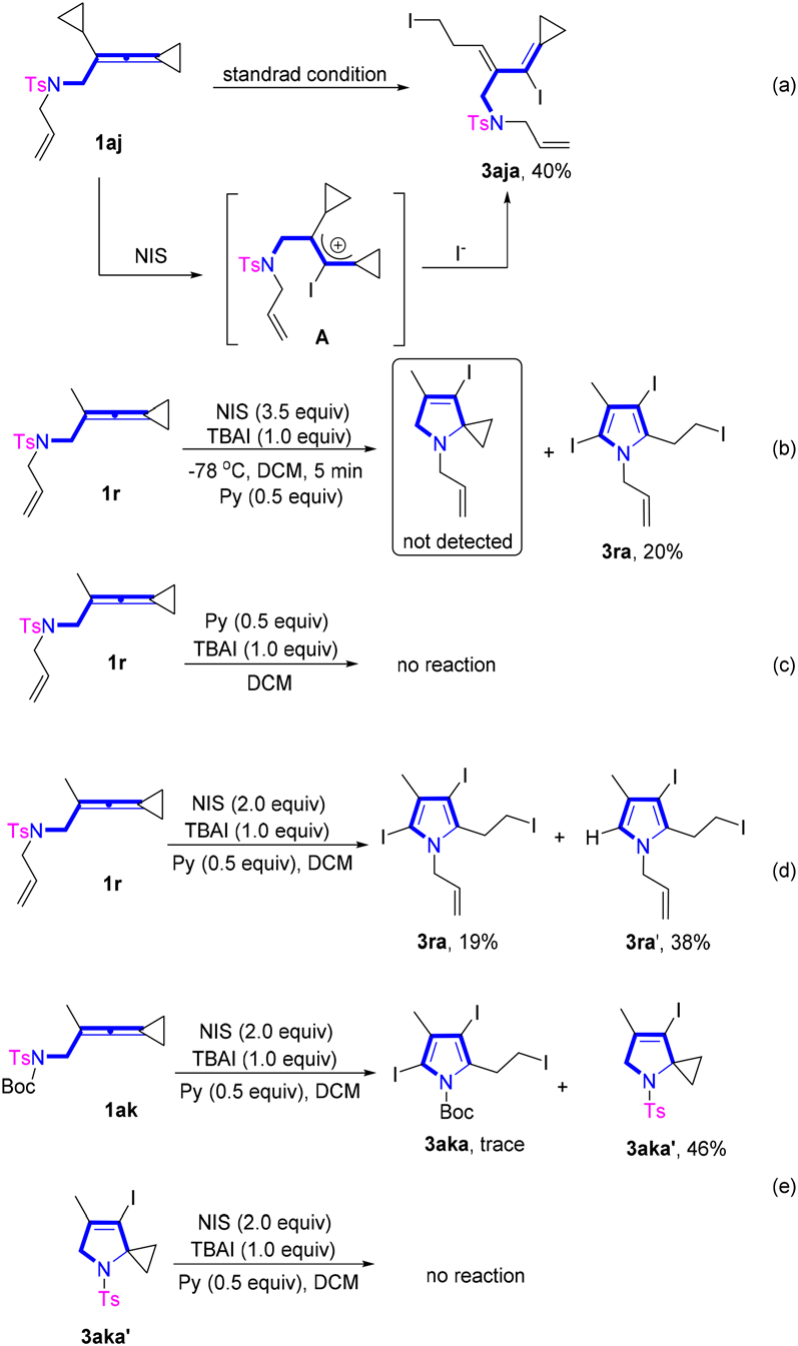
Control experiments.

**Scheme 7:**
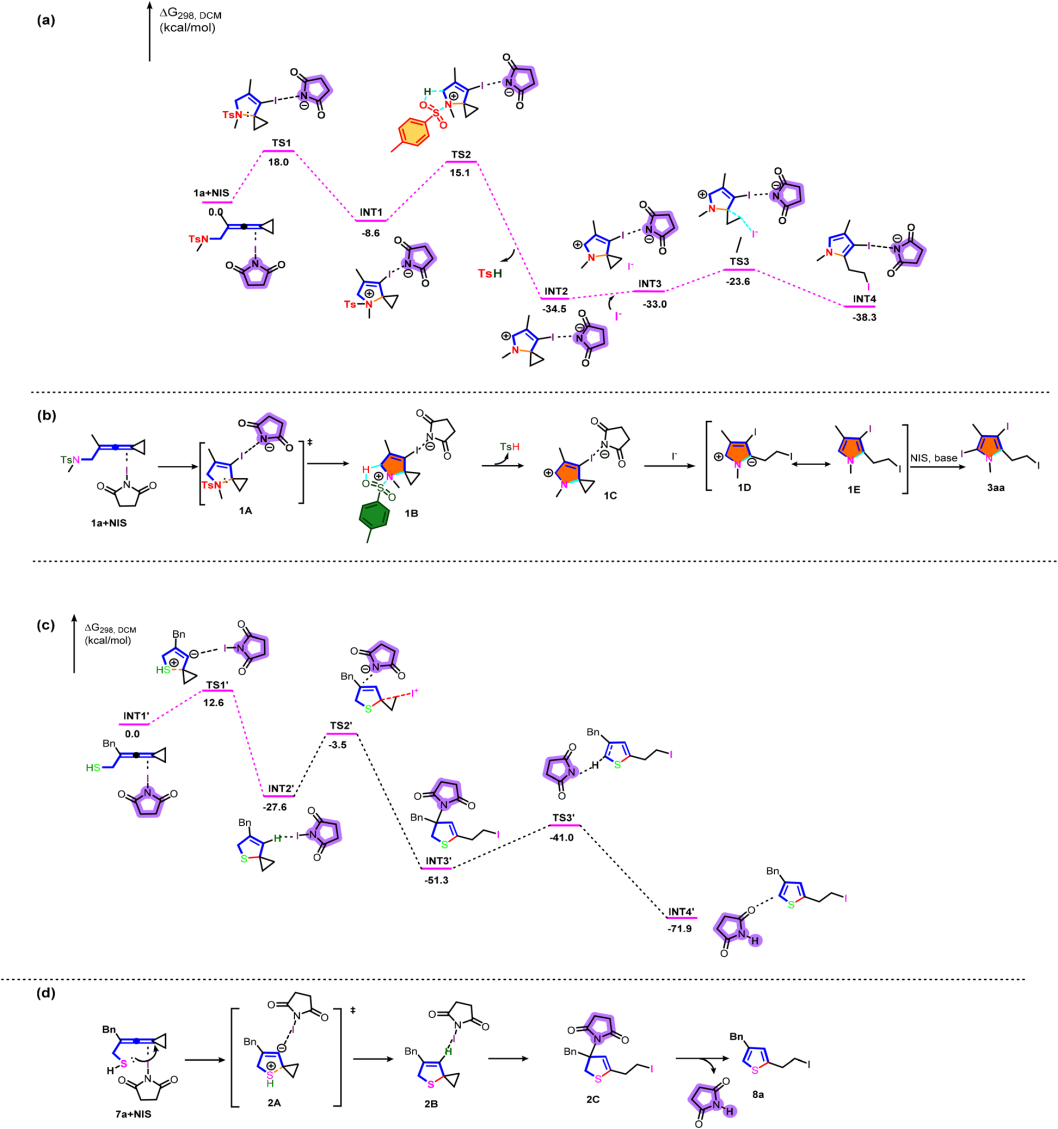
(a) DFT calculation of 3aa. (b) Proposed reaction mechanism of 3aa. (c) DFT calculation of 8a. (d) Proposed reaction mechanism of 8a.

To further unveil the reaction mechanism, we carried out a series of control experiments (Scheme 6) and density functional theory (DFT) calculations (Scheme 7). When VDCP 1aj was utilized as the substrate for the reaction under the standard conditions, instead of the desired pyrrole product, a cyclopropane ring-opened product 3aja was obtained in 40% yield, suggesting that during the reaction of 1aj with NIS, I^+^ tended to add to the middle carbon atom of the allene moiety to generate an allyl cationic intermediate A (Scheme 6a). Afterwards, the cyclopropane in 1aj was attacked by iodine anion to afford the cyclopropane ring-opened product 3aja. Subsequently, we attempted to trap the reaction intermediates in the system at −78 °C with VDCP 1r as the substrate to verify the possible reaction pathway (Scheme 6b). Unfortunately, we did not trap any useful intermediate presumably due to the very fast reaction rate of this transformation. In addition, it was found that using 1r as the substrate, no reaction occurred in the absence of NIS (Scheme 6c). Moreover, when the employed amount of NIS was reduced to 2.0 equiv., a large amount of diiodinated product 3ra′ was afforded under the standard conditions, indicating that the triiodosubstituted product was forged by the iodination of the diiodosubstituted product (Scheme 6d). In addition, when 1ak was used as a substrate, we isolated a by-product 3aka′ in 46% yield. However, 3aka′ was unable to convert to the desired product under standard conditions (Scheme 6e). Therefore, 3aka′ was not an active intermediate for this reaction, thus we exclude the possible halocyclization and deprotection pathway (see page S17 in the ESI†).

We subsequently embarked on DFT calculations to gain insight into the reaction mechanism. All calculations have been performed at the SMD(DCM)/B3LYP/6-311+G(d,p)//B3LYP/6-31G(d) level with the Gaussian 16 program.^11^ The solvation Gibbs free energy profile in dichloromethane for the suggested reaction pathway is shown in Scheme 7a (see Table S7 in the ESI† for more information). We investigated the reaction pathway starting from the complex 1a+NIS shown in Scheme 7a. Firstly, 1a+NIS undergoes cyclization *via*TS1 with an energy barrier of 18.0 kcal mol^−1^ to form a cationic intermediate INT1. The Ts moiety in INT1 promotes subsequent dehydrogenation to generate INT2 and release TsH through an activation barrier of 23.7 kcal mol^−1^, which is a highly exothermic process and indicates that the process shown in Scheme 7a is thermodynamically favorable and has a possibility to take place. In addition, the intermediate INT3 is obtained by adding iodine anion to the system. Then, the intermediate INT3 undergoes a cyclopropane ring-opening step to give the pyrrole intermediate INT4*via*TS3 with an energy barrier of 9.4 kcal mol^−1^, which is also an exothermic process.

Based on the aforementioned experimental results and the detailed DFT calculations, we propose a plausible reaction mechanistic paradigm (Scheme 7b). Initially, in the presence of NIS, the intramolecular nucleophilic attack of the nitrogen atom to the allylic cation moiety *via* transition state 1A affords cyclized cationic intermediate 1B. Then, the Ts moiety in 1B promotes subsequent dehydrogenation to give another cyclized cationic intermediate 1C and release TsH. Subsequently, the iodine anion attacks cyclopropane and undergoes a ring-opening reaction of cyclopropane to yield the zwitterionic intermediate 1D (see Fig. S5 in the ESI† for more information), which undergoes a subsequent aromatization to furnish the desired pyrrole intermediate 1E. Finally, the halogenation reaction of 1E in the presence of base and NIS gives the desired triiodosubstituted product 3aa. Furan derivatives 6 were generated by a similar mechanism. However, the low reactivity of furans made it difficult to perform further halogenation reactions under the standard conditions, only furnishing diiodosubstituted products.

Next, we investigated the mechanistic details for the generation of thiophene derivatives through computational studies (Scheme 7c). The intermediate INT1′ undergoes cyclization and intramolecular hydrogen transfer *via*TS1′ with an energy barrier of 12.6 kcal mol^−1^ to generate a stable spirocyclic intermediate INT2′. The transition state TS1′ is probably stabilized by *N*-iodosuccinimide in the system. Subsequently, the intermediate INT3′ is generated through a cyclopropane ring opening reaction in the presence of NIS, passing through TS2′ with an energy barrier of 24.1 kcal mol^−1^. The following deprotonation takes place to afford the product complex INT3′*via*TS3′ with an energy barrier of 10.3 kcal mol^−1^. On the basis of DFT calculations shown in Scheme 7c, the plausible reaction mechanism for the production of 8a is depicted in Scheme 7d. Initially, 7a is cyclized by intramolecular nucleophilic attack to yield intermediate 2B*via* the transition state 2A. Subsequently, 8a is produced through cyclopropane ring opening and deprotonation induced by *N*-iodosuccinimide.

**Scheme 8:**
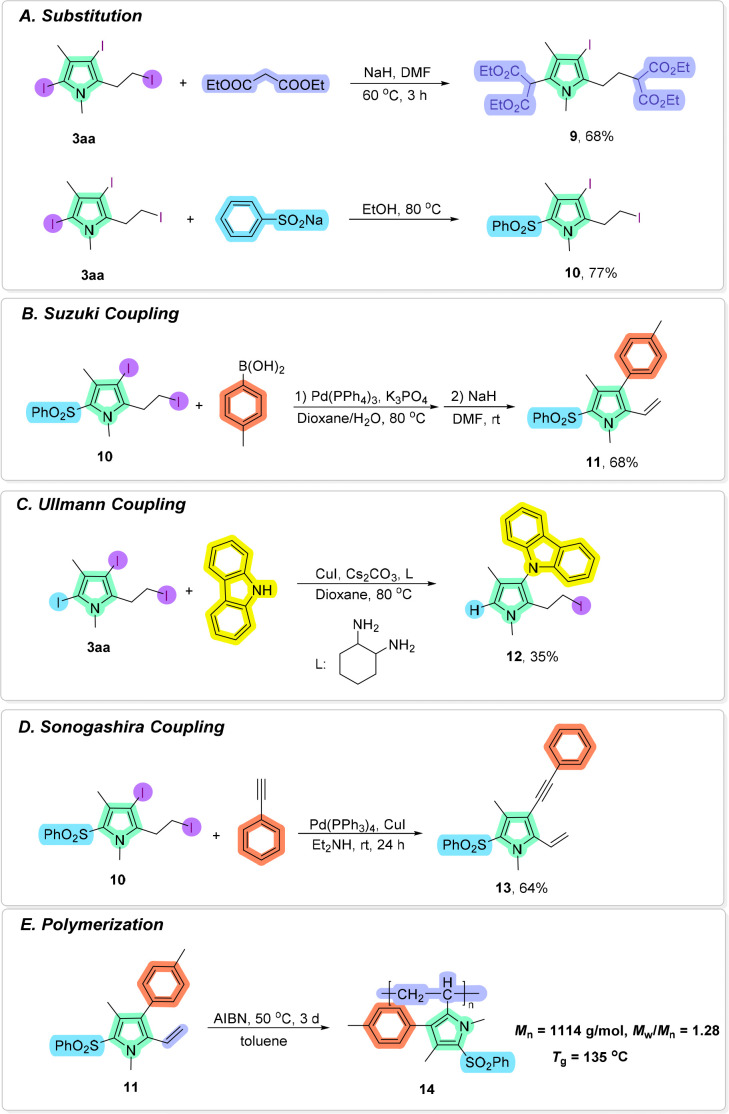
Synthetic transformations. (A) 3aa (0.5 mmol), NaH (2.5 equiv.), DMF (5.0 mL), 60 °C; 3aa (0.5 mmol), PhSO_2_Na (2.0 equiv.), EtOH; (B) 10 (0.2 mmol), 4-MeC_6_H_4_B(OH)_2_ (2.5 equiv.), Pd(PPh_3_)_4_ (10 mol%), K_3_PO_4_ (3.0 equiv.); (C) 3aa (0.2 mmol), carbazole (1.5 equiv.), CuI (20 mol%), Cs_2_CO_3_ (2.0 equiv.); (D) 10 (0.2 mmol), phenylacetylene (2.0 equiv.), Pd(PPh_3_)_4_ (5 mol%), CuI (10 mol%), (E) 11, AIBN, toluene.

The synthetic utility of the current protocol was demonstrated by diverse derivatizations of the obtained pyrrole product 3aa. As illustrated in Scheme 8A, substitution of 3aa with diethyl malonate under basic conditions in DMF successfully provided functionalized pyrrole 9 in 68% yield. In addition, in the presence of sodium benzenesulfinate, 3aa could be substituted to give pyrrole 10 in 77% yield.^12^ Moreover, pyrrole 10 could be effectively transformed into the corresponding product through a Pd-catalyzed Suzuki–Miyaura cross-coupling reaction with 4-methylphenylboronic acid^13^ and a combined elimination reaction under basic conditions in DMF^14^ (for pyrrole 11) (Scheme 8B). A carbazole substituted pyrrole 12 could be obtained in 35% yield through a Cu-catalyzed Ullmann reaction with carbazole (Scheme 8C). Furthermore, the Sonogashira coupling^15^ reaction of pyrrole 10 with phenylacetylene afforded pyrrole 13 in 64% yield (Scheme 8D). In addition, polypyrroles have been widely used in electrochemistry, and biomedical and sensor applications due to their excellent conductive properties.^16^ Polymerization of pyrrole 11 with AIBN as the radical initiator furnished the polymeric product 14 with a molecular weight of 1114 g mol^−1^ (*M*_w_/*M*_n_ = 1.28) and the glass transition temperature (*T*_g_) as 135 °C^17^ (Scheme 8E).

## Conclusions

In summary, we have established a novel and practical strategy for the rapid construction of functionalized pyrroles, furans and thiophenes under metal-free and mild conditions. This synthetic protocol is quite efficient, affording these five-membered heterocycles in moderate to excellent yields within a short reaction time (<30 min.) through NXS mediated desulfonylative/dehydrogenative cyclization of vinylidenecyclopropanes (VDCPs). Compared with traditional reaction modes that frequently employ different synthetic methods to prepare the related substrate depending on the required heteroatom (N, O, S), this reaction can easily provide these heterocycles through a single way. Furthermore, the reaction mechanism was investigated by a series of control experiments and DFT calculations. DFT calculations revealed that a desulfonylative reaction is initiated by the intramolecular nucleophilic attack of the nitrogen atom on the allylic cation moiety and the desulfonylative reaction is the rate-determining step. In addition, the dehydrogenative cyclization may involve a synergistic effect of *N*-iodosuccinimide and iodine anion. Moreover, synthetic transformations demonstrated that this strategy could realize concise synthesis of multi-substituted five-membered heterocyclic compounds and polymers. Further studies on discovering the detailed mechanism and novel reaction types to synthesize other useful compounds are underway.

## Data availability

Experimental and computational data have been made available as the ESI.†

## Author contributions

Z. Meng, J. Yan and C. Ning contributed to the investigation. Z. Meng and Y. Wei contributed to the calculations. Z. Meng, Y. Wei and M. Shi contributed to the conceptualization and writing-original draft.

## Conflicts of interest

There are no conflicts to declare.

## Supplementary Material

SC-014-D3SC01542D-s001

SC-014-D3SC01542D-s002
