# Supplementary material for: Construction of pyrroles, furans and thiophenes via intramolecular cascade desulfonylative/dehydrogenative cyclization of vinylidenecyclopropanes induced by NXS (X = I or Br)
Source: Chem Sci. 2023 Jun 13;14(28):7648–55. doi: 10.1039/d3sc01542d (PMC10355115; doi:10.1039/d3sc01542d)
Supplement: SC-014-D3SC01542D-s001 [file SC-014-D3SC01542D-s001.pdf]

Electronic Supplementary Material (ESI) for Chemical Science.
This journal is © The Royal Society of Chemistry 2023

Supporting Information

Construction of pyrroles and furans via intramolecular cascade desulfonylative/dehydrogenative cyclization of vinylidenecycloproanes induced by NXS (X = I or Br)

Zhe Meng,[a] Jun Yan,[a] Chao Ning,[a] Min Shi*[a,b] and Yin Wei*[b]

[a] *Key Laboratory for Advanced Materials and Institute of Fine Chemicals, Key Laboratory for Advanced Materials and Feringa Nobel Prize Scientist Joint Research Center, School of Chemistry & Molecular Engineering, East China University of Science and Technology, Meilong Road No.130, Shanghai, 200237 (China),* [b] *State Key Laboratory of Organometallic Chemistry, Center for Excellence in Molecular Synthesis, University of Chinese Academy of Sciences, Shanghai Institute of Organic Chemistry, Chinese Academy of Sciences, 345 Lingling Road, Shanghai 200032, P. R. China.* mshi@mail.sioc.ac.cn, weiyin@sioc.ac.cn

Table of Contents

1. General remarks....S2
2. General procedures for the synthesis of substrates **1**, **5** and **7**....S3
3. Optimization of reaction conditions....S9
4. General procedure for the synthesis of **3**, **4**, **6** and **8**....S13
5. Control experiment....S15
6. Gram scale reaction....S17
7. Transformation of product **3aa**....S18
8. Spectroscopic data of substrates **1**, **5** and **7**....S23
9. Spectroscopic data of products **3**, **4**, **6**, **8**, **9**, **11**, **12**, **13**....S78
10. Computational studies.... ....S163
11. X-Ray structures....S1670
12. References....S1893

1. General remarks

^{1}H, ^{13}C and ^{19}F NMR spectra were recorded at 400 MHz or 600 MHz, 100 MHz or 150 MHz and 376 MHz, respectively. HRMS spectra were recorded by EI, ESI, FI method. Infrared spectra were recorded on a Perkin-Elmer PE-983 spectrometer with absorption in cm^{-1}. Mass spectra were recorded by EI, ESI, and HRMS was measured on an Agilent Technologies 6224 TOF LC/MS instrument and a Waters Micromass GCT Permier. Melting points were determined on a digital melting point apparatus and temperatures were uncorrected. X-ray structure was determined on a Bruker Smart-1000 X-ray Diffraction meter. The employed solvents were dried up by standard methods when necessary. Commercially obtained reagents were used without further purification. All reactions were monitored by TLC plate analysis with silica gel coated plates (Huanghai GF254). Flash column chromatography was performed by using 300-400 mesh silica gel eluting with ethyl acetate and petroleum ether at increased pressure.

2. General procedures for the synthesis of substrates 1

Synthesis of substrates **1a-1n**, **1q-1s**, **1r-1y**, and **1aa-1ae**[1]

Step 1

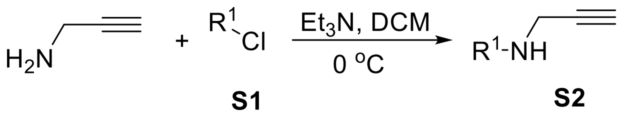

To a solution of **S1** (60 mmol) in DCM (100 mL) was added Et_3N (90 mmol) and 2-propargylamine (90 mmol) at 0 °C and the resulting solution was allowed to stir at 0 °C for 12 h. Extracted with H_2O (20 mL × 3), dried over anhydrous Na_2SO_4, and filtered, the organic phase was concentrated under reduced pressure to dryness to give a yellow solid **S2**.

Step 2

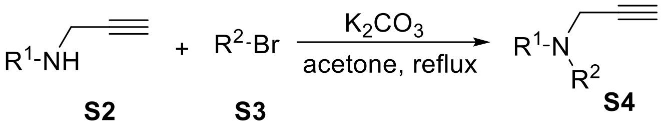

To a solution of **S2** (50 mmol) in acetone (100 mL) was added K_2CO_3 (1.5 equiv) and **S3** (2.0 equiv). The resulting solution was allowed to stir at 70 °C for 12 h. After filtration, the organic phase was concentrated under reduced pressure and the residue was purified by a flash column chromatography on silica gel to give the desired products **S4** (PE:EA = 10:1).

Step 3

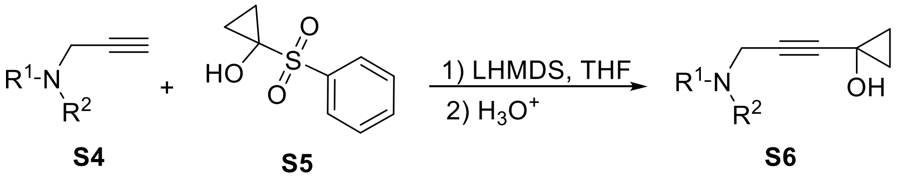

To a solution of compounds **S4** (20 mmol) in THF (30 mL) was slowly added LHMDS (24 mmol, 1.0 M in THF) at -78 °C under the protection of argon and the resulting solution was allowed to stir at -78 °C for 30 min before a solution of **S5** (10 mmol) in THF (10 mL) was added into the above mixture. Consequently, the reaction mixture was allowed to warm up to room temperature and the mixture was left standing overnight. Then, saturated NH_4Cl solution was added to quench the reaction. Extracted with ethyl acetate, dried over anhydrous Na_2SO_4, and filtered, the organic phase

was concentrated under reduce pressure and the residue was purified by a flash column chromatography on silica gel to give the desired products **S6** (PE:EA = 4:1~2:1).

Step 4

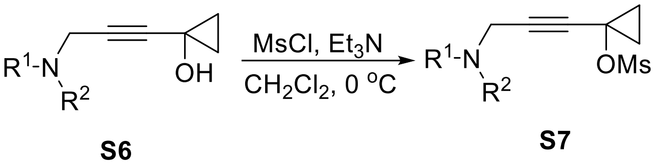

To a solution of **S6** (5.0 mmol) and anhydrous Et_3N (2.0 equiv) in CH_2Cl_2 (20 mL) was added MsCl (2.0 equiv) at 0 °C under the protection of argon and the resulting solution was allowed to stir at 0 °C for 1.0 h before some amounts of water were added to quench the reaction. Extracted with CH_2Cl_2 (20 mL × 3), dried over anhydrous Na_2SO_4, and filtered, the organic phase was concentrated under reduce pressure and the residue purified by a flash column chromatography on silica gel to give the desired products **S7** (PE:EA = 2:1).

Step 5

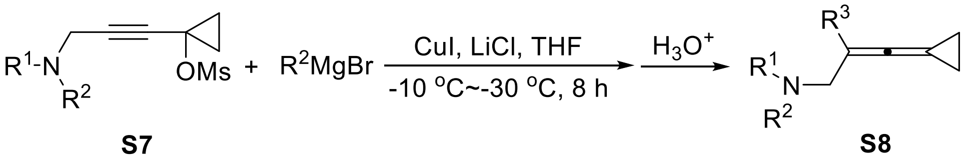

To a flame dried 50 mL three-neck flask was added anhydrous CuI (8.8 mmol), LiCl (8.8 mmol) and the solvent THF (20 mL) under the protection of argon and then, the flask was cooled to -10 °C before the solution of RMgBr or RMgCl (2.0 M, 4.0 mL) was added dropwise into the flask under argon. After 5 minutes, the flask was moved into a -30~-40 °C bath and the reaction mixture was stirred for a while before a solution of **S7** (4.0 mmol) in THF (10 mL) was added dropwise into the above flask. The resulting solution was allowed to stir at -40 °C for 8 h before saturated NH_4Cl solution was added to quench the reaction. Extracted with EA (20 mL × 3), dried over anhydrous Na_2SO_4, and filtered, the organic phase was concentrated under reduce pressure and the residue was purified by a flash column chromatography on silica gel to give the desired products **S8** (PE:EA = 10:1).

Synthesis of substrate **1o**

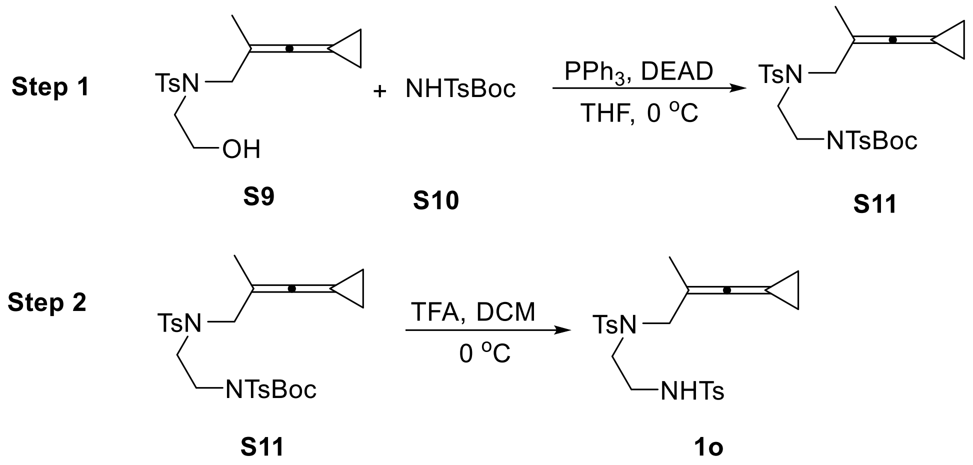

Step 1: To a flame dried 50 mL flask was added **S9** (2 mmol), **S10** (1.2 equiv), PPh_3 (1.2 equiv) and the solvent THF (10 mL) under the protection of argon. Then, the flask was cooled to 0 °C before the DEAD (1.2 equiv) was added dropwise into the flask under argon. The resulting solution was allowed to stir at 0 °C for 12 h before water was added to quench the reaction. Extracted with EA (20 mL × 3), dried over anhydrous Na_2SO_4, filtered, the organic phase was concentrated under reduce pressure and the residue was purified by a flash column chromatography on silica gel to give the desired product **S11** (PE:EA = 4:1).

Step 2: To a solution of **S11** (1.0 mmol) in DCM (10 mL) was added TFA (2.0 mL) at 0 °C and the resulting solution was allowed to stir at 0 °C for 8 h before water was added to quench the reaction. Extracted with DCM, dried over anhydrous Na_2SO_4, filtered, the organic phase was concentrated under reduce pressure and the residue was purified by a flash column chromatography on silica gel to give the desired product **1o** (PE:EA = 2:1).

Synthesis of substrate **1p**.

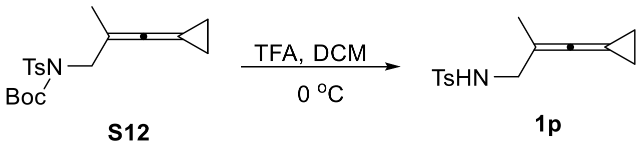

Step 1: To a solution of **S12** (1.0 mmol) in DCM (10 mL) was added TFA (2.0 mL) at 0 °C and the resulting solution was allowed to stir at 0 °C for 8 h before water was added to quench the reaction. Extracted with DCM, dried over anhydrous Na_2SO_4, and filtered, the organic phase was concentrated under reduce pressure and the residue was purified by a flash column chromatography on silica gel to give the desired product **1p** (PE:EA = 2:1).

Synthesis of substrates **5a**-**5f**.

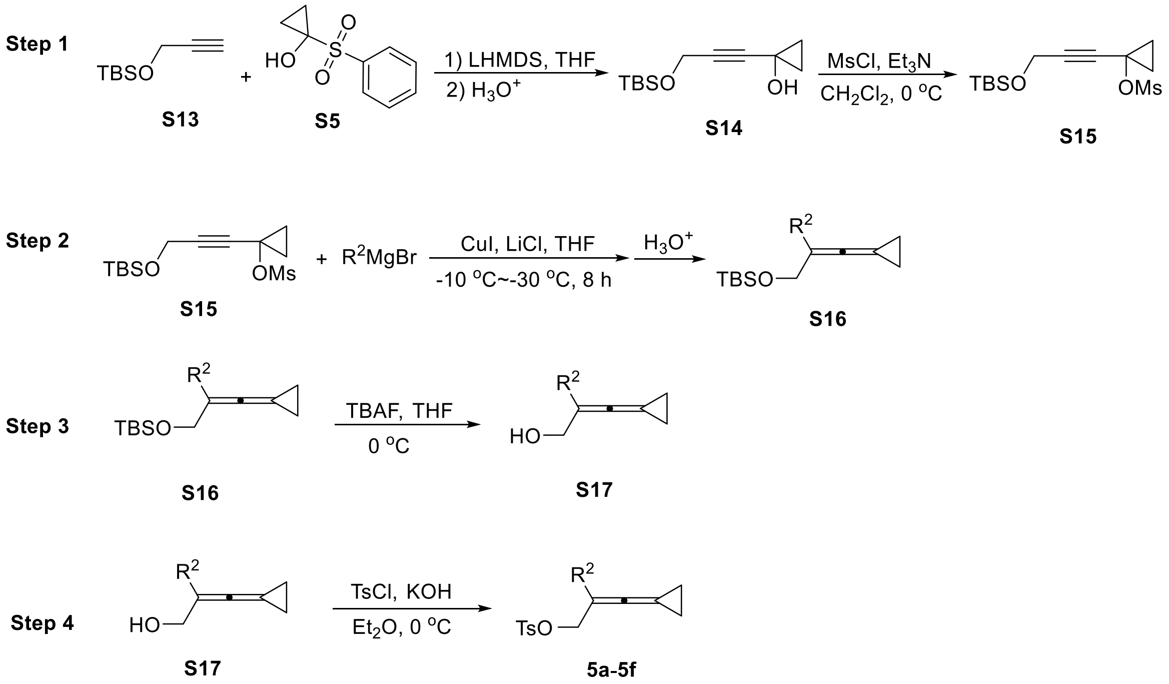

The experimental procedures of step 1 and step 2 were the same as those described above.

Step 3: To a flame dried 50 mL flask was added **S16** (2.0 mmol), and the solvent THF (10 mL) under the protection of argon and then, the flask was cooled to 0 °C before the solution of TBAF (1.0 M, 4.0 mL) was added dropwise into the flask under argon. The resulting solution was allowed to stir at 0 °C for 8.0 h before water was added to quench the reaction. Extracted with EA, dried over anhydrous Na_2SO_4, and filtered, the organic phase was concentrated under reduce pressure and the residue was purified by a flash column chromatography on silica gel to give the desired products **S17** (PE:EA = 4:1).

Step 4: To a solution of **S17** (1.0 mmol) in Et_2O (10 mL) was added TsCl (1.5 equiv) and KOH (5.0 equiv) at 0 °C and the resulting solution was allowed to stir at 0 °C for 12 h before water was added to quench the reaction. Extracted with EA, dried over anhydrous Na_2SO_4, and filtered, the organic phase was concentrated under reduce pressure and the residue was purified by a flash column chromatography on silica gel to give the desired products **5a**-**5f** (PE:EA = 4:1).

Synthesis of substrates **7a**-**7h**.

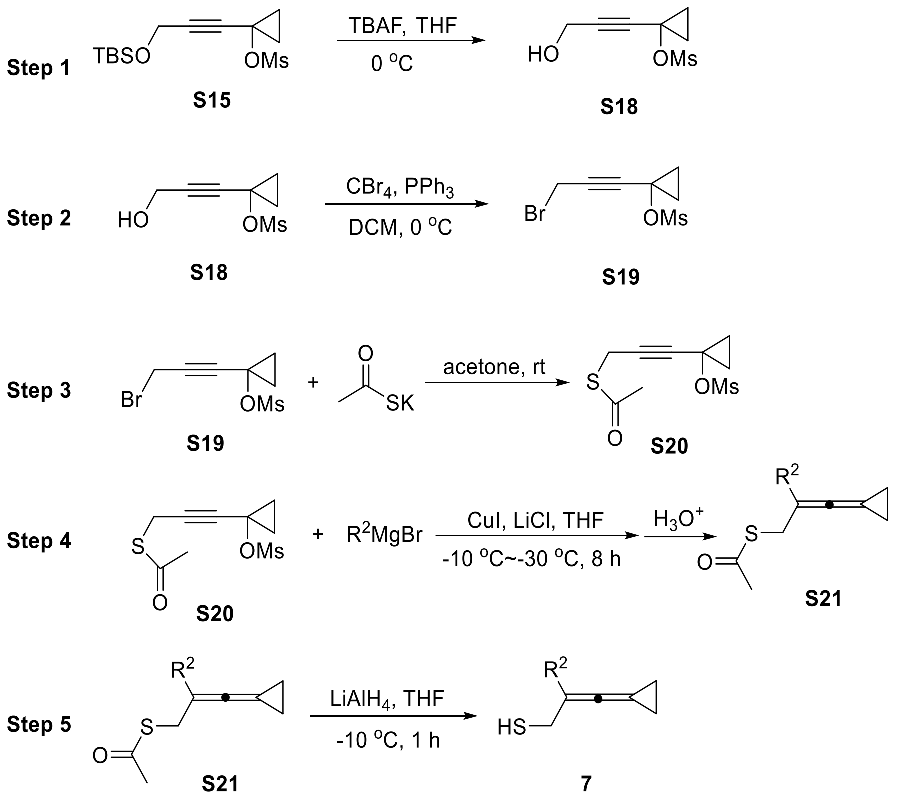

Step 1: To a flame dried 50 mL flask was added **S15** (2.0 mmol), and the solvent THF (10 mL) under the protection of argon and then, the flask was cooled to 0 °C before the solution of TBAF (1.0 M, 4.0 mL) was added dropwise into the flask under argon. The resulting solution was allowed to stir at 0 °C for 8.0 h before water was added to quench the reaction. Extracted with EA, dried over anhydrous Na_2SO_4, and filtered, the organic phase was concentrated under reduce pressure and the residue was purified by a flash column chromatography on silica gel to give the desired products **S18** (PE:EA = 1:1).

Step 2: To a solution of **S18** (1.0 mmol) in DCM (10 mL) was added CBr_4 (1.1 equiv) and the resulting solution was allowed to stir at 0 °C for 5 min before a solution of PPh_3 (1.2 equiv) in DCM (10 mL) was added dropwise into the above flask. The resulting solution was allowed to stir at 0 °C for 2 h before filtered, the organic phase was concentrated under reduce pressure and the residue was purified by a flash column chromatography on silica gel to give the desired products **S19** (PE:EA = 4:1).

Step 3: To a solution of **S19** (1.0 mmol) in acetone (5 mL) was added potassium thioacetate (1.5

equiv) and the resulting solution was allowed to stir for 6 h before water was added to quench the reaction. Extracted with EA, dried over anhydrous Na_2SO_4, and filtered, the organic phase was concentrated under reduce pressure and the residue was purified by a flash column chromatography on silica gel to give the desired products **S20** (PE:EA = 2:1).

Step 4: To a flame dried 25 mL three-neck flask was added anhydrous CuI (2.2 mmol), LiCl (2.2 mmol) and the solvent THF (10 mL) under the protection of argon and then, the flask was cooled to -10 ^oC before the solution of RMgBr or RMgCl (1.0 M, 2.0 mL) was added dropwise into the flask under argon. After 5 minutes, the flask was moved into a -30~-40 ^oC bath and the reaction mixture was stirred for a while before a solution of **S20** (1.0 mmol) in THF (10 mL) was added dropwise into the above flask. The resulting solution was allowed to stir at -40 ^oC for 8 h before saturated NH_4Cl solution was added to quench the reaction. Extracted with EA (10 mL × 3), dried over anhydrous Na_2SO_4, and filtered, the organic phase was concentrated under reduce pressure and the residue was purified by a flash column chromatography on silica gel to give the desired products **S21** (PE:EA = 4:1).

Step 5: To a flame dried 25 mL flask was added $LiAlH_4$ (2 mmol), and the solvent THF (10 mL) under the protection of argon and then, the flask was cooled to -10 ^oC before the solution of **S21** (1 mmol) was added dropwise into the flask under argon. The resulting solution was allowed to stir at -10 ^oC for 1 h before NH_4Cl was added to quench the reaction. Extracted with EA, dried over anhydrous Na_2SO_4, and filtered, the organic phase was concentrated under reduce pressure and the residue was purified by a flash column chromatography on silica gel to give the desired products **7** (PE:EA = 10:1).

3. Optimization of reaction conditions

Table S1. Optimization of reaction conditions using **1r** as a template substrate (without TBAI).[a]

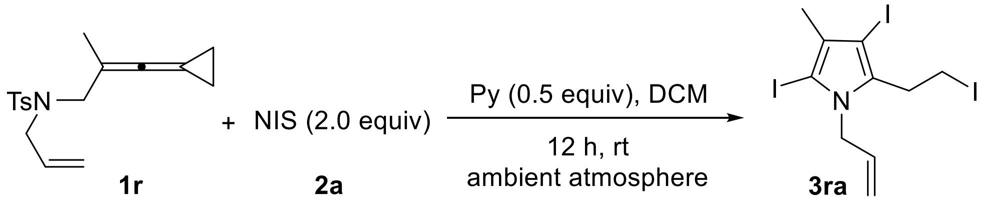

entry[a]	variation from standard condition	**3ra**, yield[b] [%]
1	none	60
2	Add $AgNTf_2$ (0.1 equiv)	30
3	Py (2.0 equiv) instead of Py (0.5 equiv)	76
4	Add $LiNTf_2$ (0.1 equiv)	60
5	Add $LiNTf_2$ (0.5 equiv)	65
6	Ar instead of air	59
7	Py (0 equiv) instead of Py (0.5 equiv)	40
8	Only add IPy_2BF_4	43
9	NCS instead of NIS	nd
10	NBS instead of NIS	44
11	3.0 equiv NIS instead of 2.0 equiv NIS	52
12	3.0 equiv I_2 insead of 2.0 equiv NIS	15

[a] Reaction carried out with **1r** (0.2 mmol, 1 equiv), **2a** (2.0 equiv), additive, DCM (2.0 mL).
[b] ^{1}H NMR yield using 1, 3, 5-trimethoxybenzene as an internal standard.

Table S2. Optimization of reaction conditions using **1r** as a template substrate (base).[a]

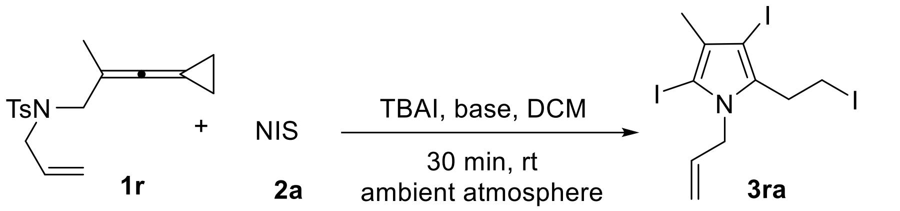

entry[a]	base	**3ra**, yield[b] [%]
1	Py	>95
2	DABCO	30
3	DMAP	76
4	Cs_2CO_3	60

[a] Reaction carried out with **1r** (0.1 mmol), **2a** (3.5 equiv), base (0.5 equiv), TBAI (1.0 equiv), DCM (2.0 mL). [b] ^{1}H NMR yield using 1, 3, 5-trimethoxybenzene as an internal standard.

Table S3. Optimization of reaction conditions using **1r** as a template substrate (solvent).[a]

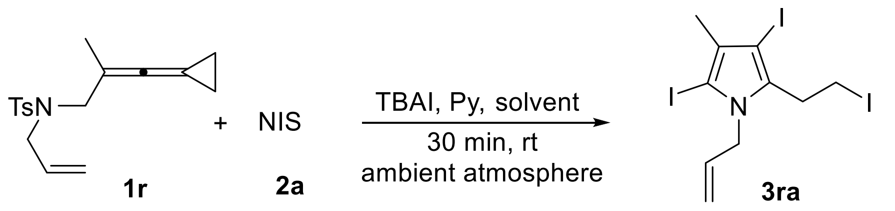

entry[a]	solvent	**3ra**, yield[b] [%]
1	DCM	>95
2	CH_3CN	85
3	THF	10
4	Dioxane	23
5	DMF	29
6	DMSO	33
7	toluene	55

[a] Reaction carried out with **1r** (0.1 mmol), **2a** (3.5 equiv), Py (0.5 equiv), TBAI (1.0 equiv), solvent (2.0 mL). [b] ^{1}H NMR yield using 1, 3, 5-trimethoxybenzene as an internal standard.

Table S4. Optimization of reaction conditions using **1r** as a template substrate (equiv of Py).[a]

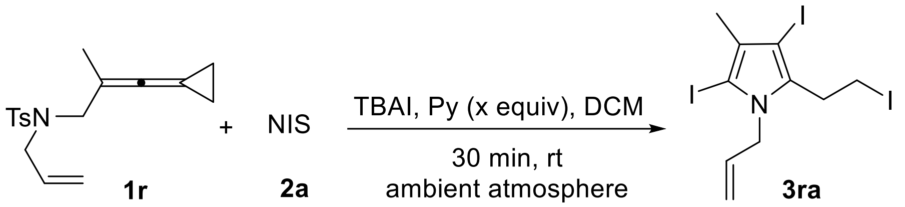

entry[a]	X	**3ra**, yield[b] [%]
1	0	59
2	0.5	>95
3	1.0	68
4	2.0	55
5	3.0	50

[a] Reaction carried out with **1r** (0.1 mmol), **2a** (3.5 equiv), Py (x equiv), TBAI (1.0 equiv), DCM (2.0 mL). [b] ^{1}H NMR yield using 1, 3, 5-trimethoxybenzene as an internal standard.

Table S5. Optimization of reaction conditions using **1r** as a template substrate (time).[a]

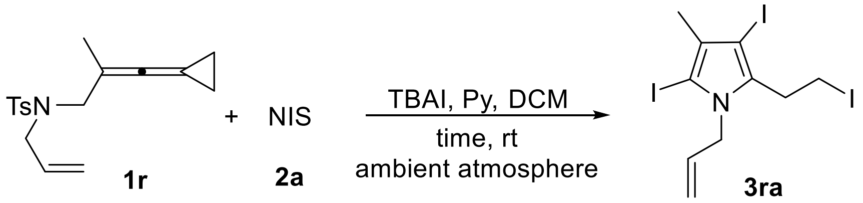

entry[a]	Time	**3ra**, yield[b] [%]
1	1 h	82
2	30 min	>95
3	2 h	76
4	4 h	60
5	12 h	50

[a] Reaction carried out with **1r** (0.1 mmol), **2a** (3.5 equiv), Py (0.5 equiv), TBAI (1.0 equiv), DCM (2.0 mL). [b] ^{1}H NMR yield using 1, 3, 5-trimethoxybenzene as an internal standard.

Table S6. Optimization of reaction conditions using **7g** as a template substrate (equiv of NIS).[a]

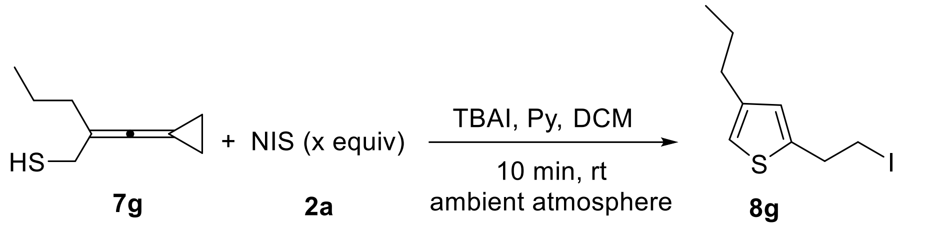

entry[a]	X	yield[%][b]
1	0	<3
2	0.1	12
3	0.5	46
4	1.0	93

[a] Reaction carried out with **7g** (0.1 mmol), **2a** (x equiv), Py (0.5 equiv), TBAI (1.0 equiv), DCM (2.0 mL). [b] ^{1}H NMR yield using 1, 3, 5-trimethoxybenzene as an internal standard.

4. General procedure for the synthesis of 3, 4, 6 and 8

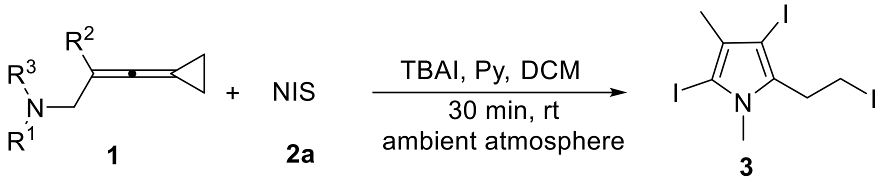

To a 4.0 mL tube were added substrate **1** (0.10 mmol, 1.0 equiv), NIS **2a** (0.35 mmol, 3.5 equiv) and TBAI (0.1 mmol, 1.0 equiv) and then DCM (2.0 mL) and pyridine (Py) (0.05 mmol, 0.5 equiv) were added to this flask via a syringe. The resulting mixture was stirred for 30 min. Then, the solvent was removed under vacuum and the residue was purified by a silica gel column chromatography (PE:EA = 50:1) to give the desired products **3**.

General procedure for the synthesis of 4

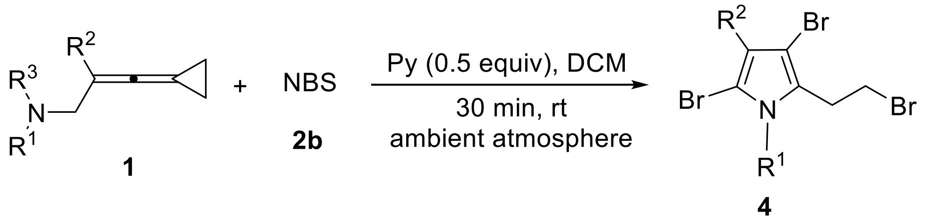

To a 4.0 mL tube were added substrate **1** (0.10 mmol, 1.0 equiv) and NBS **2b** (0.3 mmol, 3.0 equiv) and then DCM (2.0 mL) and pyridine (Py) (0.05 mmol, 0.5 equiv) were added to this flask via a syringe. The resulting mixture was stirred for 30 min. Then, the solvent was removed under vacuum and the residue was purified by a silica gel column chromatography (PE:EA = 50:1) to give the desired products **4**.

General procedure for the synthesis of 6

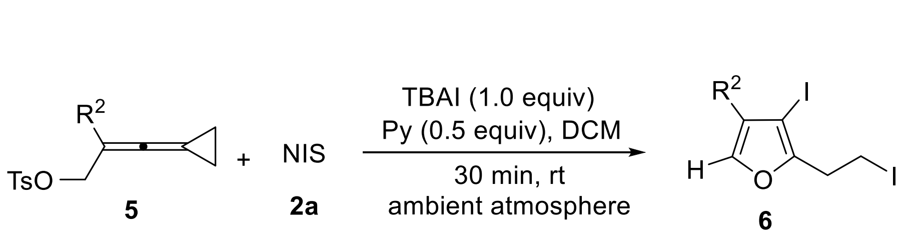

To a 4.0 mL tube were added substrate **5** (0.10 mmol, 1.0 equiv) and NIS **2a** (0.2 mmol, 2.0 equiv) and then DCM (2.0 mL) and pyridine (Py) (0.05 mmol, 0.5 equiv) were added to this flask via a

syringe. The resulting mixture was stirred for 30 min. Then, the solvent was removed under vacuum and the residue was purified by a silica gel column chromatography (PE:EA = 50:1) to give the desired products **6**.

General procedure for the synthesis of 8

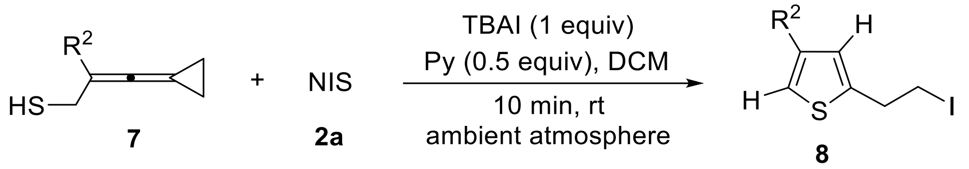

To a 4.0 mL tube were added substrate **7** (0.10 mmol, 1.0 equiv) and NIS **2a** (0.2 mmol, 1.0 equiv) and then DCM (2.0 mL) and pyridine (Py) (0.05 mmol, 0.5 equiv) were added to this flask via a syringe. The resulting mixture was stirred for 30 min. Then, the solvent was removed under vacuum and the residue was purified by a silica gel column chromatography (PE:EA = 50:1) to give the desired products **8**.

Figure S1

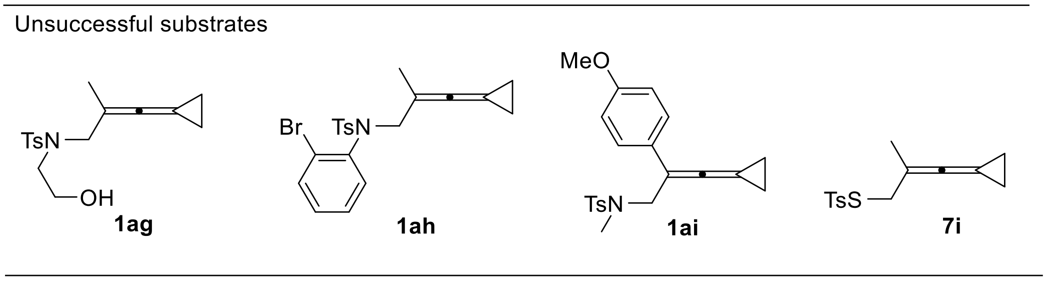

5. Control experiment

(a)

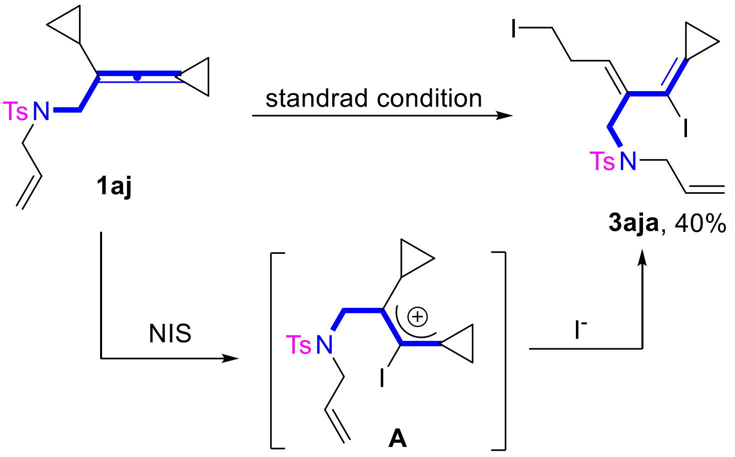

To a 4.0 mL tube were added substrate **1aj** (0.10 mmol, 1.0 equiv), **NIS** (0.35 mmol, 3.5 equiv) and TBAI (0.1 mmol, 1.0 equiv) and then DCM (2.0 mL) and pyridine (Py) (0.05 mmol, 0.5 equiv) were added to this tube via a syringe. The resulting mixture was stirred for 30 min. Then, the solvent was removed under vacuum and the residue was purified by a silica gel column chromatography (PE:EA = 50:1) to give the product **3aja**.

(b)

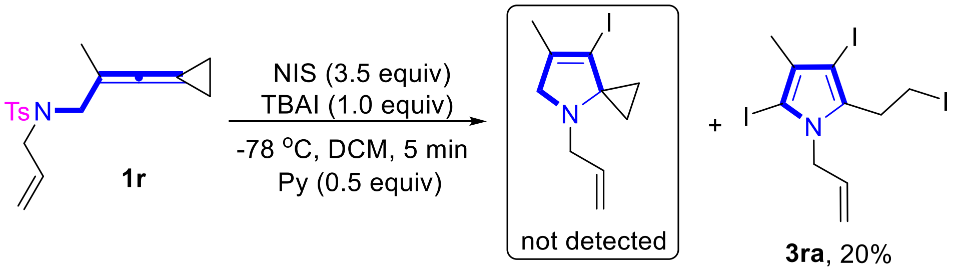

To a 4.0 mL tube were added substrate **1r** (0.10 mmol, 1.0 equiv), **NIS** (0.35 mmol, 3.5 equiv) and TBAI (0.1 mmol, 1.0 equiv) and then DCM (2.0 mL) and pyridine (Py) (0.05 mmol, 0.5 equiv) were added to this tube via a syringe, and the flask was cooled to -78 °C. The resulting mixture was stirred for 5 min. Then, the solvent was removed under vacuum and the residue was purified by a silica gel column chromatography (PE:EA = 50:1) to give the product **3ra** in 20% yield. This result indicated that substrate **1r** did not give the expected intermediate under the standard conditions, but the corresponding product was obtained directly.

(c)

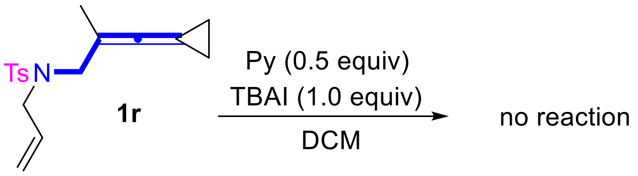

To a 4.0 mL tube were added substrate **1r** (0.10 mmol, 1.0 equiv), TBAI (0.1 mmol, 1.0 equiv) and then DCM (2.0 mL), pyridine (Py) (0.05 mmol, 0.5 equiv) were added to this tube via a syringe. The resulting mixture was stirred for 5 min. After the removal of solvent under reduced pressure, the residue was purified by a column chromatography on silica gel (PE / EA = 50:1). This result indicated that substrate **1r** did not react in the absence of NIS.

(d)

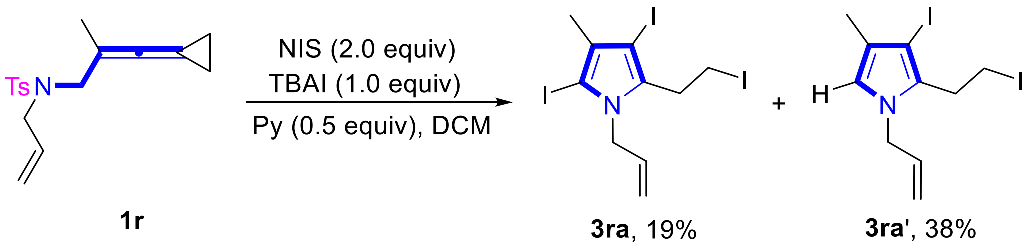

To a 4.0 mL tube were added substrate **1r** (0.10 mmol, 1.0 equiv), **NIS** (0.20 mmol, 2.0 equiv), TBAI (0.1 mmol, 1.0 equiv) and then DCM (2.0 mL), pyridine (Py) (0.05 mmol, 0.5 equiv) were added to this tube via a syringe. The resulting mixture was stirred for 30 min. Then, the solvent was removed under vacuum and the residue was purified by a silica gel column chromatography (PE:EA = 50:1) to give the products **3ra** and **3ra'**. This result indicated that substrate **1r** produced diiodosubstituted product **3ra'** under the condition with insufficient amount of **NIS**, rendering that the triiodosubstituted product was afforded by the further iodination of the corresponding diiodosubstituted product.

(e)

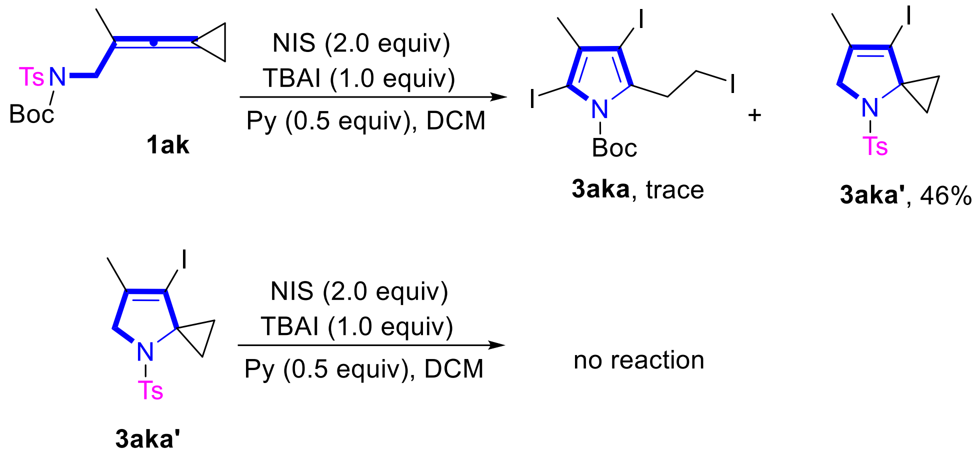

To a 4.0 mL tube were added substrate **1ak** (0.10 mmol, 1.0 equiv), NIS (0.20 mmol, 2.0 equiv), TBAI (0.1 mmol, 1.0 equiv) and then DCM (2.0 mL), pyridine (Py) (0.05 mmol, 0.5 equiv) were added to this tube via a syringe. The resulting mixture was stirred for 30 min. Then, the solvent was removed under vacuum and the residue was purified by a silica gel column chromatography (PE:EA = 50:1) to give the products **3aka'**. Then, to a 4.0 mL tube were added substrate **1ak** (0.05 mmol, 1.0 equiv), NIS (0.10 mmol, 2.0 equiv), TBAI (0.05 mmol, 1.0 equiv) and then DCM (1.0 mL), pyridine (Py) (0.025 mmol, 0.5 equiv) were added to this tube via a syringe. The resulting mixture was stirred for 30 min. No reaction occurs. These results indicated that **3aka'** was not the active intermediate for this reaction. Thus, we exclude the following reaction pathway.

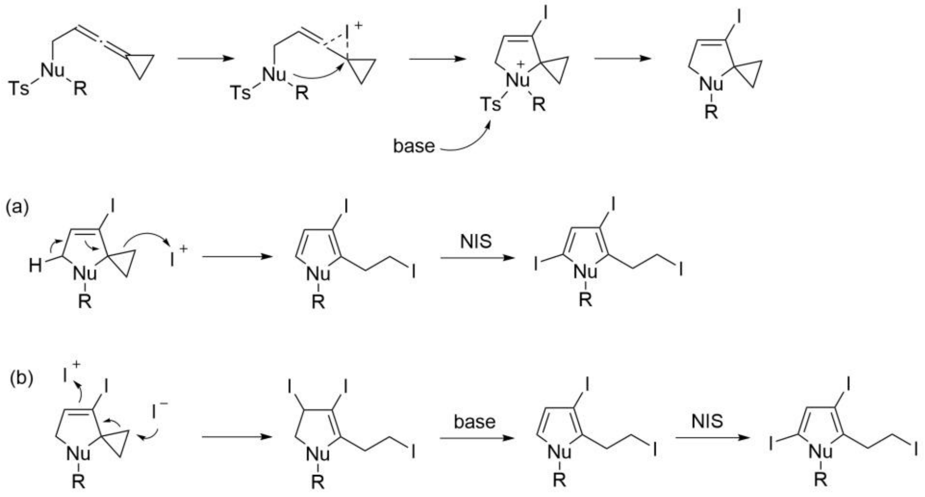

6. Gram scale reaction

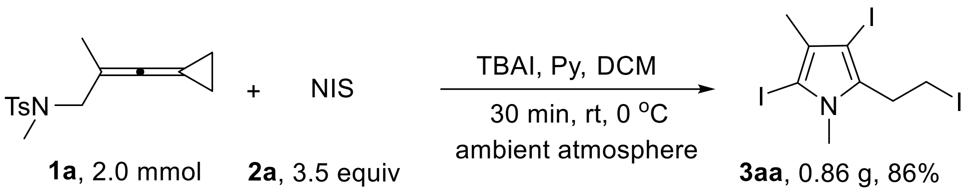

To a flame 50 mL flask was added **1a** (2.0 mmol, 0.55 g, 1.0 equiv), TBAI (2.0 mmol, 0.74 g, 1.0 equiv), pyridine (Py) (1.0 mmol, 80 uL, 0.5 equiv) and the solvent DCM (10 mL) and then, the flask was cooled to 0 °C before NIS **2a** (7.0 mmol, 1.58 g, 3.5 equiv) was added into the flask. The resulting solution was allowed to stir for 30 min before saturated water was added to quench the reaction. Extracted with EA (10 mL x 3), dried over anhydrous Na_2SO_4, and filtered, the organic phase was concentrated under reduce pressure and the residue was purified by a flash column chromatography on silica gel to give the desired product **3aa** (PE:EA = 100:1).

7. Transformation of product 3aa

A. Substitution

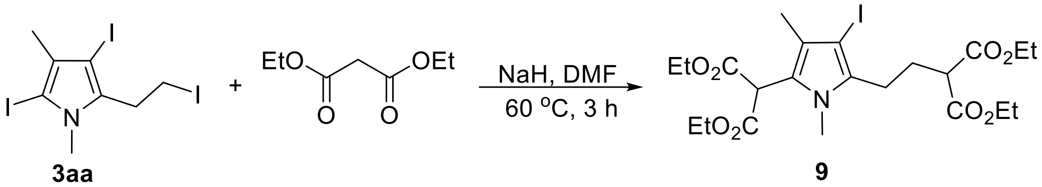

To a flame dried 50 mL flask was added NaH, diethyl malonate and the solvent DMF (20 mL) under the protection of argon and then, the flask was heated to 60 °C before substrate **3aa** was added dropwise into the flask under argon. The resulting solution was allowed to stir for 3 h before saturated NH_4Cl solution was added to quench the reaction. Extracted with EA (20 mL × 3), dried over anhydrous Na_2SO_4, and filtered, the organic phase was concentrated under reduce pressure and the residue was purified by a flash column chromatography on silica gel to give the desired product **9** (PE:EA = 2:1).[2]

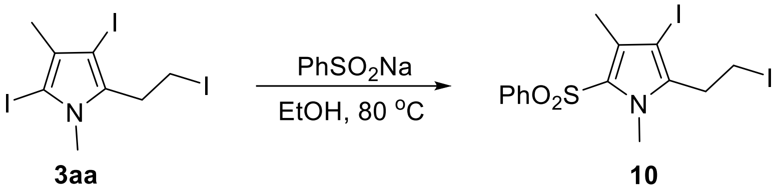

To a 50 mL flask was added **3aa**, $PhSO_2Na$ and the solvent EtOH (20 mL) and then, the flask was heated to 80 °C. The resulting solution was allowed to stir for 12 h before the water was added to quench the reaction. Extracted with EA (20 mL × 3), dried over anhydrous Na_2SO_4, and filtered, the organic phase was concentrated under reduce pressure and the residue was purified by a flash column chromatography on silica gel to give the desired product **10** (PE:EA = 4:1).[2]

B. Suzuki Coupling

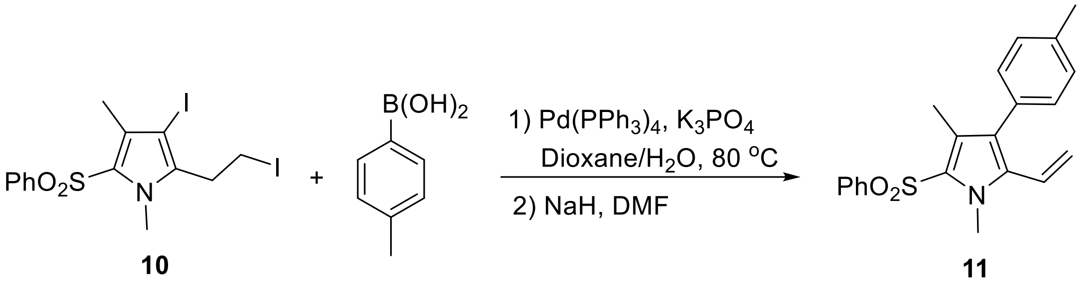

(1) To a flame dried 25 mL flask was added **10** (0.2 mmol), 4-tolylboronic acid (2.0 equiv), $Pd(PPh_3)_4$ (5 mol%), K_3PO_4 (2.5 equiv) and the solvent dioxane/H_2O (2.0+2.0 mL) under the protection of argon and then, the flask was heated to 80 ^oC. The resulting solution was allowed to stir for 12 h before saturated NH_4Cl solution was added to quench the reaction. Extracted with EA (10 mL × 3), dried over anhydrous Na_2SO_4, and filtered, the organic phase was concentrated under reduce pressure and the residue was purified by a flash column chromatography on silica gel to give the intermediate product (PE:EA = 4:1).[3]

(2) To a 25 mL flask was added the intermediate product, NaH, and the solvent DMF and then, the resulting solution was allowed to stir for 12 h. Extracted with EA (10 mL × 3), dried over anhydrous Na_2SO_4, and filtered, the organic phase was concentrated under reduce pressure and the residue was purified by a flash column chromatography on silica gel to give the desired product **11** (PE:EA = 4:1).[4]

C. Ullmann Coupling

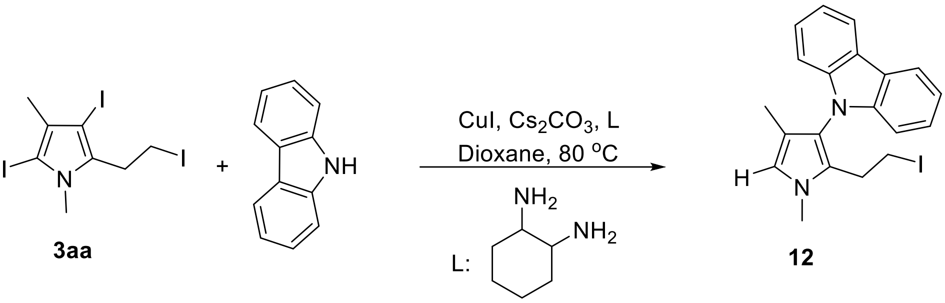

To a flame dried 25 mL flask was added **3aa** (0.2 mmol), carbazole (1.5 equiv), CuI (20 mol%), Cs_2CO_3 (2.0 equiv) and the solvent of dioxane (2.0 mL) under the protection of argon and then, the flask was heated to 80 °C. The resulting solution was allowed to stir for 12 h before water was added to quench the reaction. Extracted with EA (10 mL × 3), dried over anhydrous Na_2SO_4, and filtered, the organic phase was concentrated under reduce pressure and the residue was purified by a flash column chromatography on silica gel to give the desired product **12** (PE:EA = 4:1).

D. Sonogashira Coupling

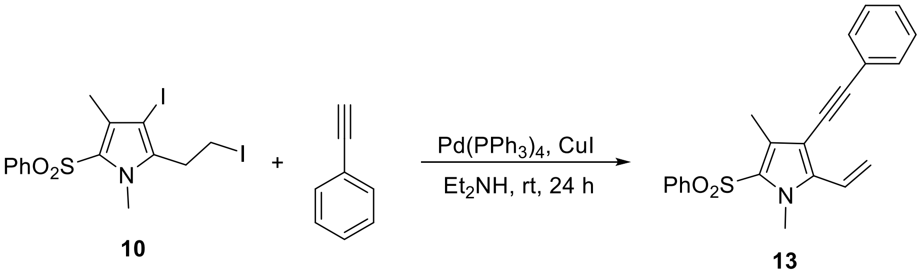

To a flame dried 25 mL flask was added **10** (0.2 mmol), phenylacetylene (2.0 equiv), $Pd(PPh_3)_4$ (5 mol%), CuI (10 mol%) and the solvent Et_2NH (2.0 mL) under the protection of argon and then, the resulting solution was allowed to stir for 12 h before water was added to quench the reaction. Extracted with EA (10 mL×3), dried over anhydrous Na_2SO_4, and filtered, the organic phase was concentrated under reduce pressure and the residue was purified by a flash column chromatography on silica gel to give the desired product **13** (PE:EA = 4:1).[5]

E. Polymerization

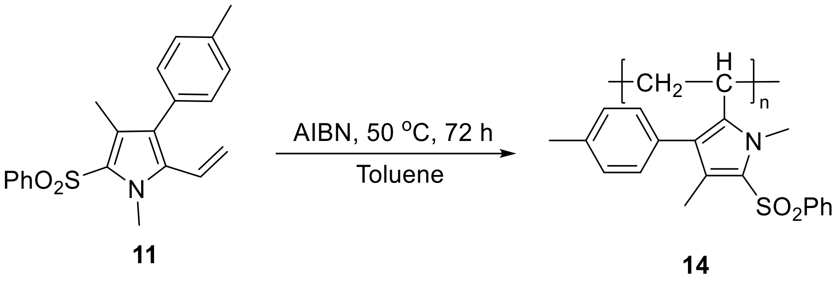

To a flame dried 10 mL flask was added **11** (150 mg), AIBN (15 mg), and the solvent of toluene (1.0 mL) under the protection of argon and then, the resulting solution was allowed to stir for 72 h. The reaction mixture was poured into n-pentane (10 mL), filtered and washed with n-pentane give 53 mg of a yellow solid **14**.[6] The obtained polymeric product was analyzed by ^{1}H NMR spectroscopic data and GPC.

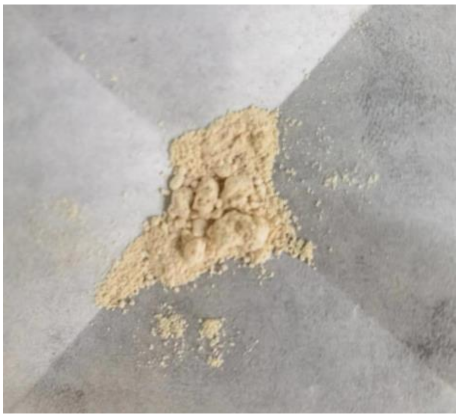

Figure S2. Picture of **14**

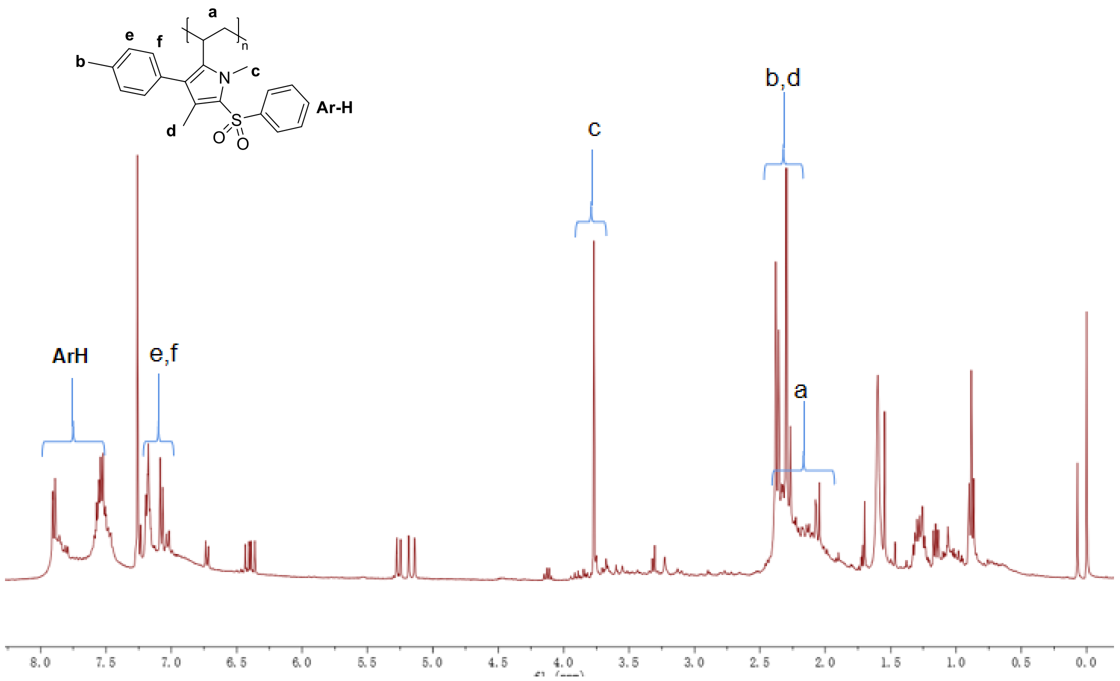

Figure S3. ^{1}H NMR spectra of **14**

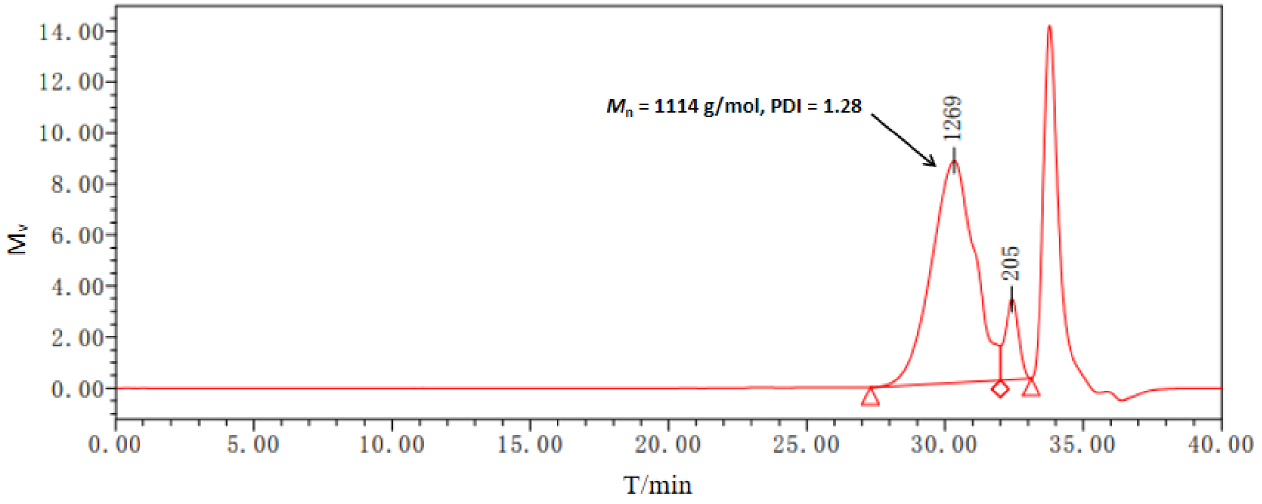

Figure S4. GPC analysis of **14**

8. Spectroscopic data of substrates 1, 5, 7

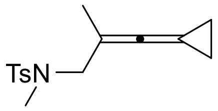

***N*-(3-cyclopropylidene-2-methyl-3λ^5-allyl)-*N*,4-dimethylbenzenesulfonamide (1a)**

A white solid, 58% yield, 178.8 mg. M.P.: 85-87 °C. ^{1}H NMR ($CDCl_3$, TMS, 600 MHz) δ 7.67 (d, *J* = 8.3 Hz, 2H), 7.31 (d, *J* = 8.0 Hz, 2H), 3.59 (s, 2H), 2.67 (s, 3H), 2.42 (s, 3H), 1.79 (s, 3H), 1.47 - 1.37 (m, 4H). ^{13}C NMR ($CDCl_3$, TMS, 150 MHz) δ 188.8, 143.2, 134.4, 129.6, 127.4, 97.1, 77.4, 54.7, 33.8, 21.4, 16.7, 7.0. IR (neat) ν 733, 771, 894, 1045, 1089, 1155, 1339, 1443, 1605, 2019 cm^{-1}. HRMS (ESI) calcd. for $C_{15}H_{19}NO_2SNa$ (M+Na): 300.1034, Found: 300.1036.

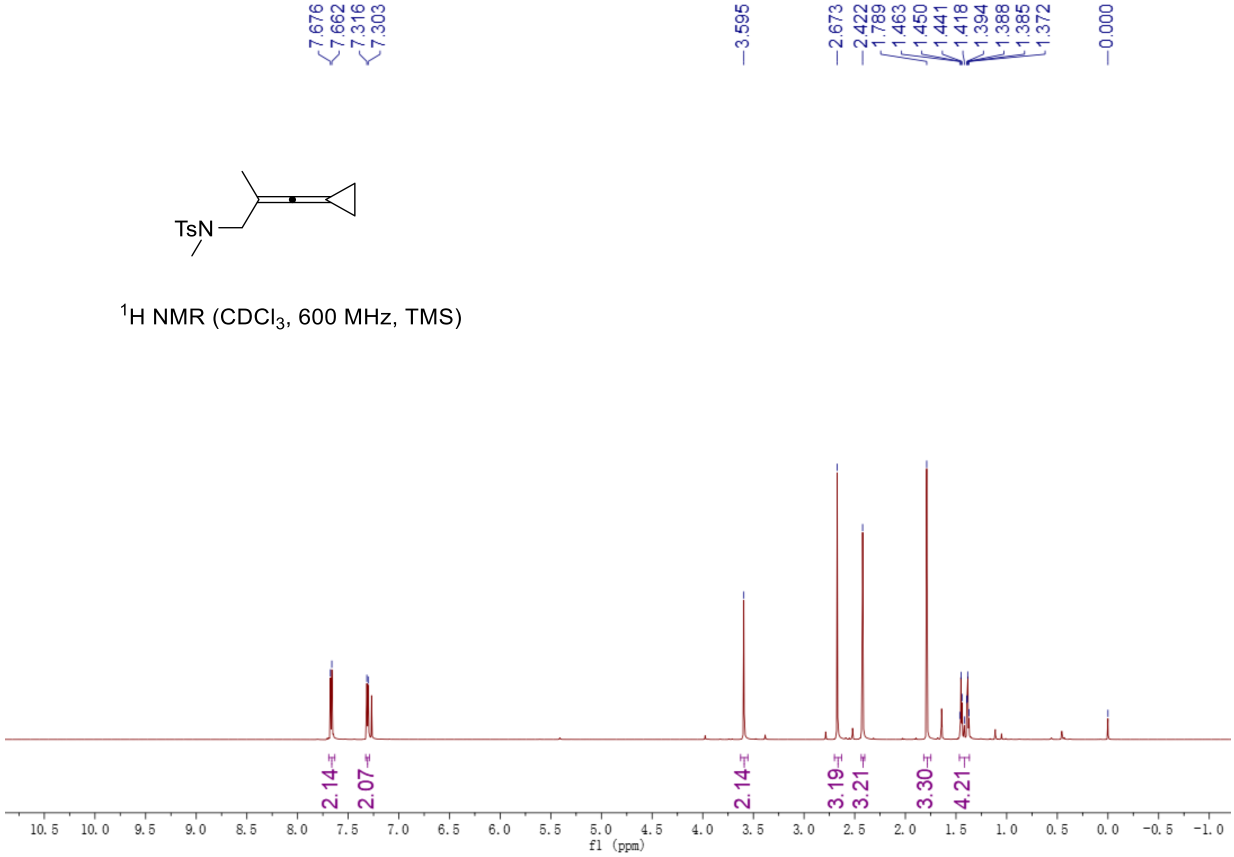

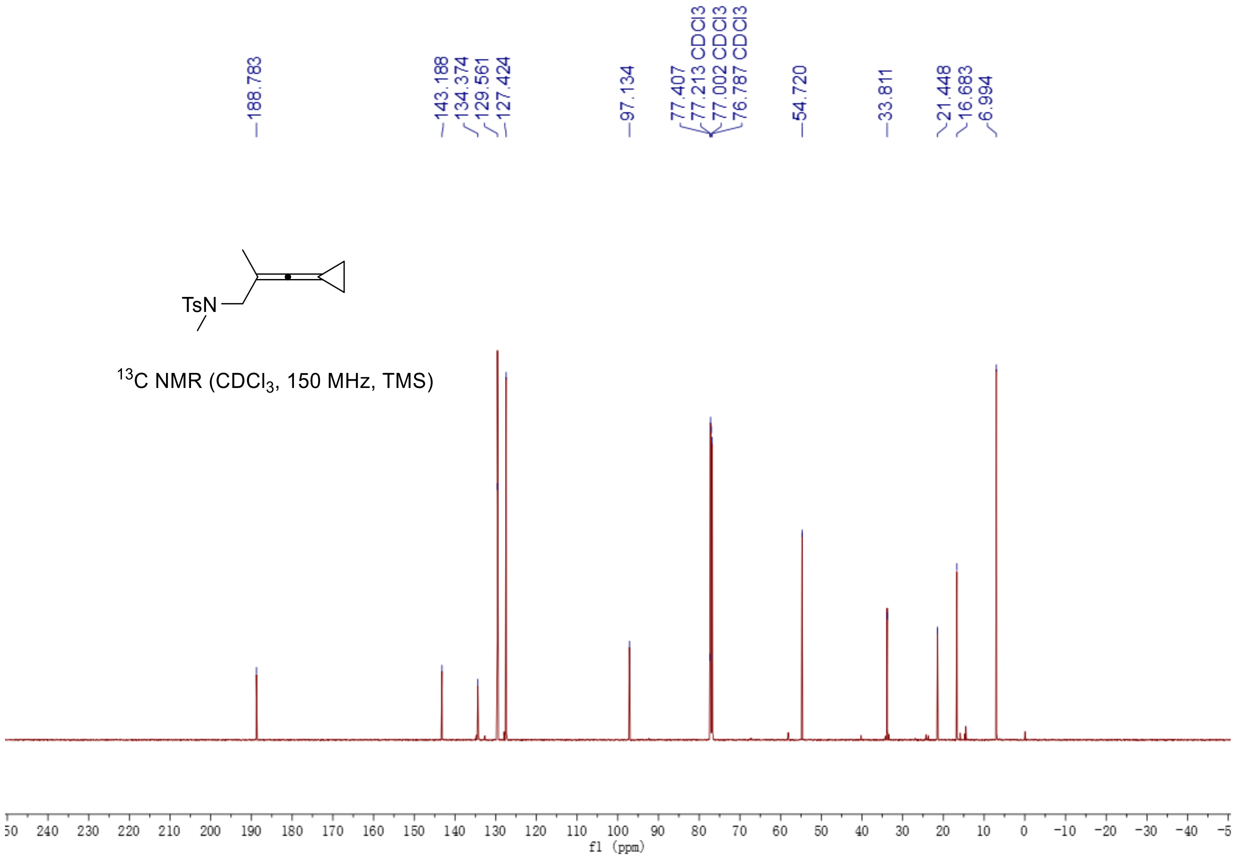

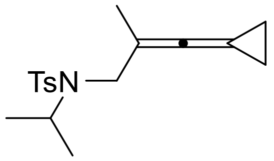

N-(3-cyclopropylidene-2-methyl-3λ^5-allyl)-N-isopropyl-4-methylbenzenesulfonamide (1b)

A yellow oil, 51% yield, 155.7 mg. ^{1}H NMR ($CDCl_3$, TMS, 400 MHz) δ 7.68 (d, *J* = 7.9 Hz, 2H), 7.26 - 7.24 (m, 2H), 4.10 - 3.99 (m, 1H), 3.85 (s, 2H), 2.41 (s, 3H), 1.82 (s, 3H), 1.58 (s, 3H), 1.48 - 1.39 (m, 4H), 1.08 (d, *J* = 6.8 Hz, 6H). ^{13}C NMR ($CDCl_3$, TMS, 100 MHz) δ 188.0, 142.7, 138.3, 129.5, 126.9, 100.5, 78.1, 49.8, 47.5, 21.5, 20.7, 16.8, 6.5. IR (neat) ν 733, 771, 894, 1045, 1089, 1155, 1339, 1443, 1605, 2019, 2917 cm^{-1}. HRMS (ESI) calcd. for $C_{17}H_{23}NO_2SNa$ (M+Na): 328.1342, Found: 328.1343.

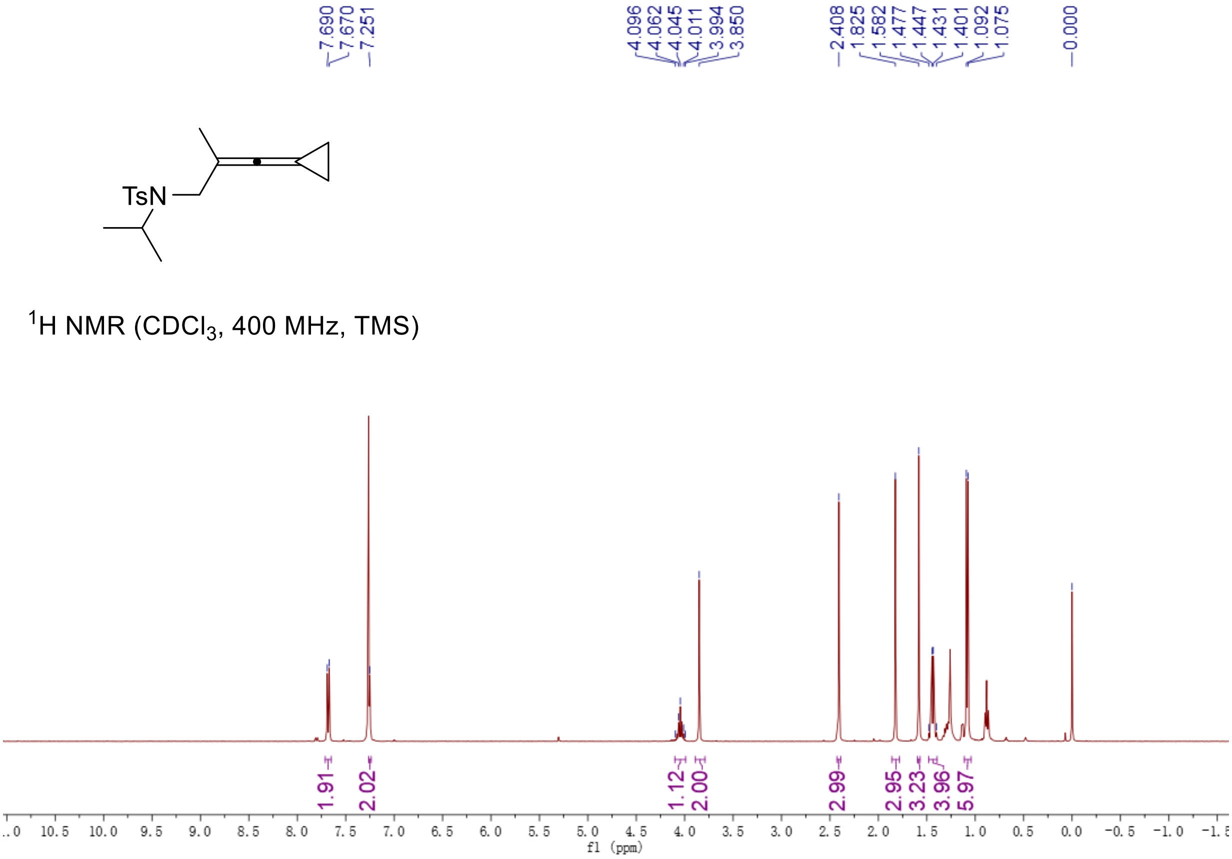

^{1}H NMR ($CDCl_3$, 400 MHz, TMS)

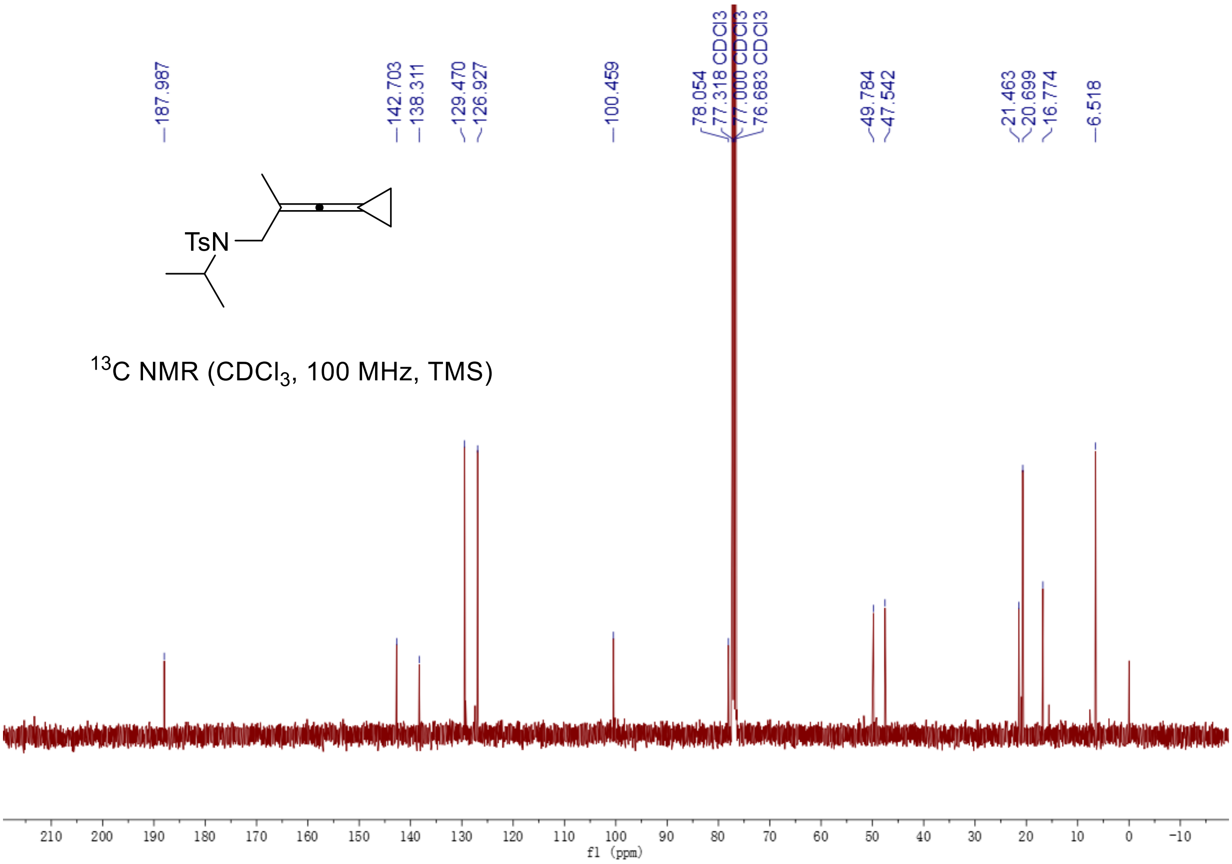

^{13}C NMR ($CDCl_3$, 100 MHz, TMS)

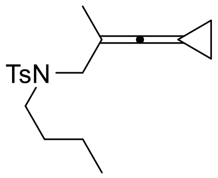

***N*-butyl-*N*-(3-cyclopropylidene-2-methyl-3λ⁵-allyl)-4-methylbenzenesulfonamide (1c)**

A colorless oil, 50% yield, 159.7 mg. ^{1}H NMR ($CDCl_3$, TMS, 600 MHz) δ 7.69 (d, *J* = 8.3 Hz, 2H), 7.28 (d, *J* = 8.1 Hz, 2H), 3.79 (s, 2H), 3.16 - 3.07 (m, 2H), 2.41 (s, 3H), 1.73 (s, 3H), 1.50 - 1.43 (m, 4H), 1.39 - 1.34 (m, 2H), 1.27 - 1.21 (m, 2H), 0.86 (t, *J* = 7.4 Hz, 3H). ^{13}C NMR ($CDCl_3$, TMS, 150 MHz) δ 188.7, 142.8, 137.4, 129.5, 127.1, 97.8, 77.5, 52.4, 47.3, 29.9, 21.5, 20.0, 16.9, 13.7, 6.9. IR (neat) ν 727, 835, 921, 985, 1083, 1157, 1249, 1339, 1461, 2021, 2928 cm^{-1}. HRMS (ESI) calcd. for $C_{18}H_{25}NO_2NaS$ (M+Na): 342.1498, Found: 342.1505.

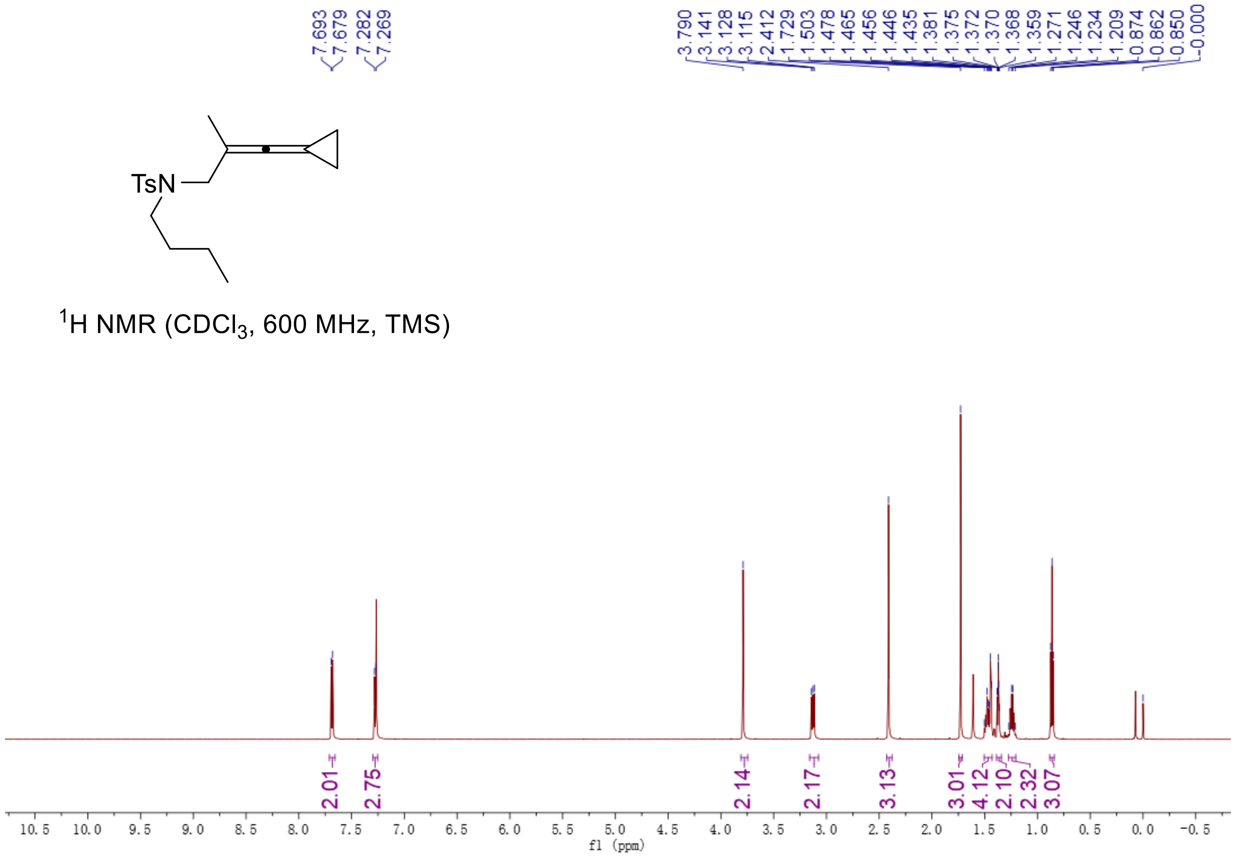

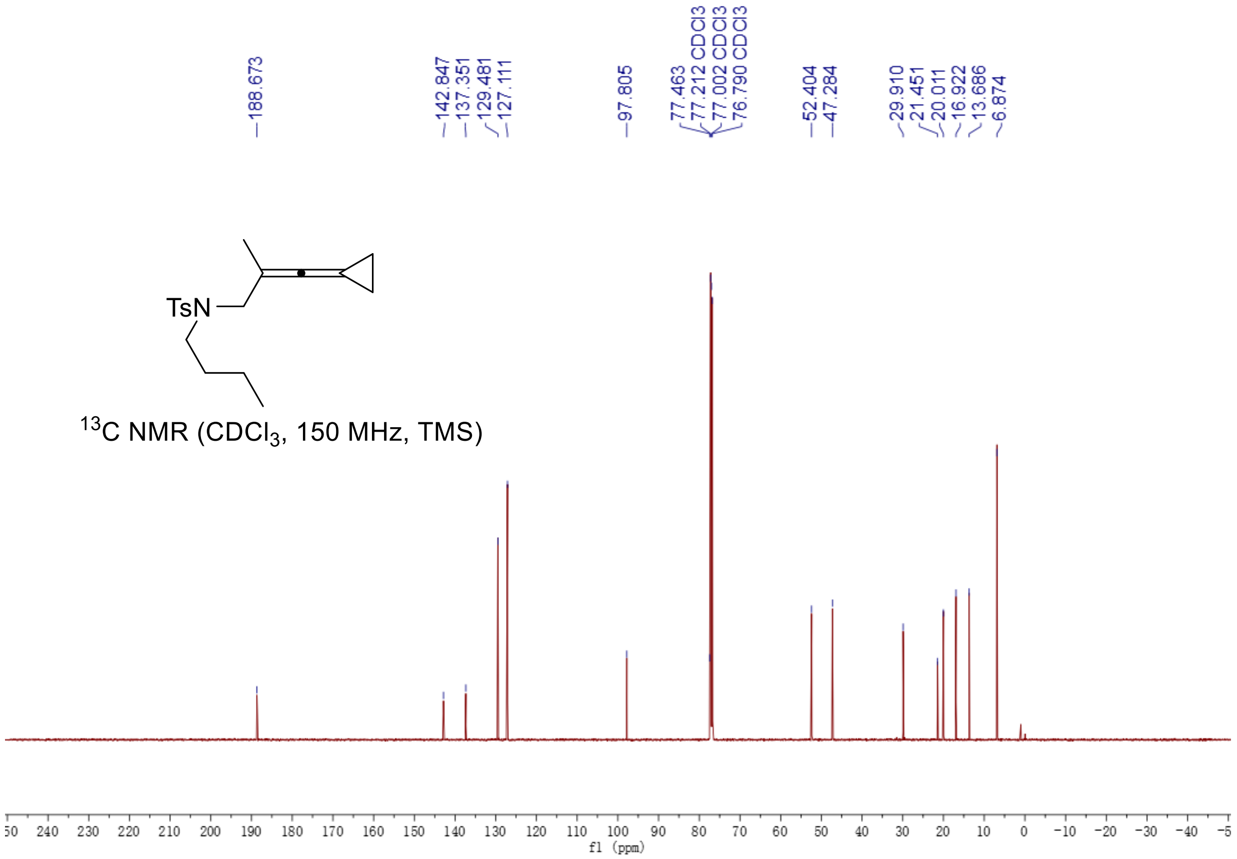

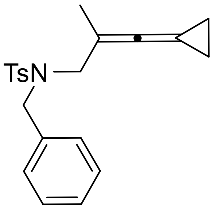

***N*-benzyl-*N*-(3-cyclopropylidene-2-methyl-3λ^5-allyl)-4-methylbenzenesulfonamide (1d)**

A colorless oil, 41% yield, 144.9 mg. ^{1}H NMR ($CDCl_3$, TMS, 400 MHz) δ 7.71 (d, J = 8.3 Hz, 2H), 7.28 (d, J = 8.1 Hz, 2H), 7.24 (s, 5H), 4.37 (s, 2H), 3.77 (s, 2H), 2.42 (s, 3H), 1.53 (s, 3H), 1.43 - 1.30 (m, 4H). ^{13}C NMR ($CDCl_3$, TMS, 100 MHz) δ 188.8, 143.0, 137.6, 136.1, 129.5, 128.8, 128.2, 127.5, 127.2, 97.3, 77.6, 51.4, 50.4, 21.5, 16.9, 6.8. IR (neat) ν 860, 891, 1008, 1088, 1157, 1331, 2029, 2930 cm^{-1}. HRMS (ESI) calcd. for $C_{21}H_{23}NO_2NaS$ (M+Na): 376.1342, Found: 376.1346.

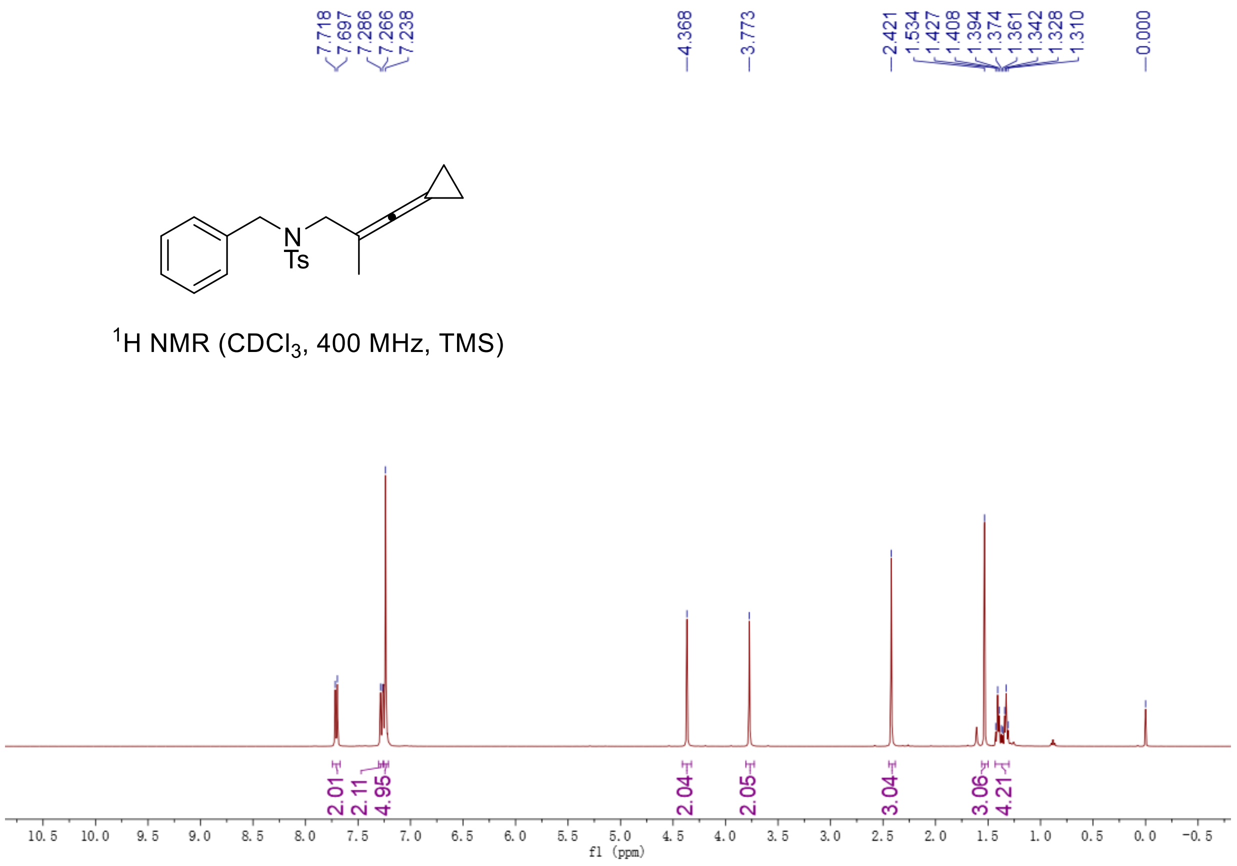

7.718
7.697
7.286
7.266
7.238
4.368
3.773
2.421
1.534
1.427
1.408
1.394
1.374
1.361
1.342
1.328
1.310
0.000
N
Ts
1H NMR (CDCl3, 400 MHz, TMS)
2.01
2.11
4.95
2.04
2.05
3.04
3.06
4.21
f1 (ppm)

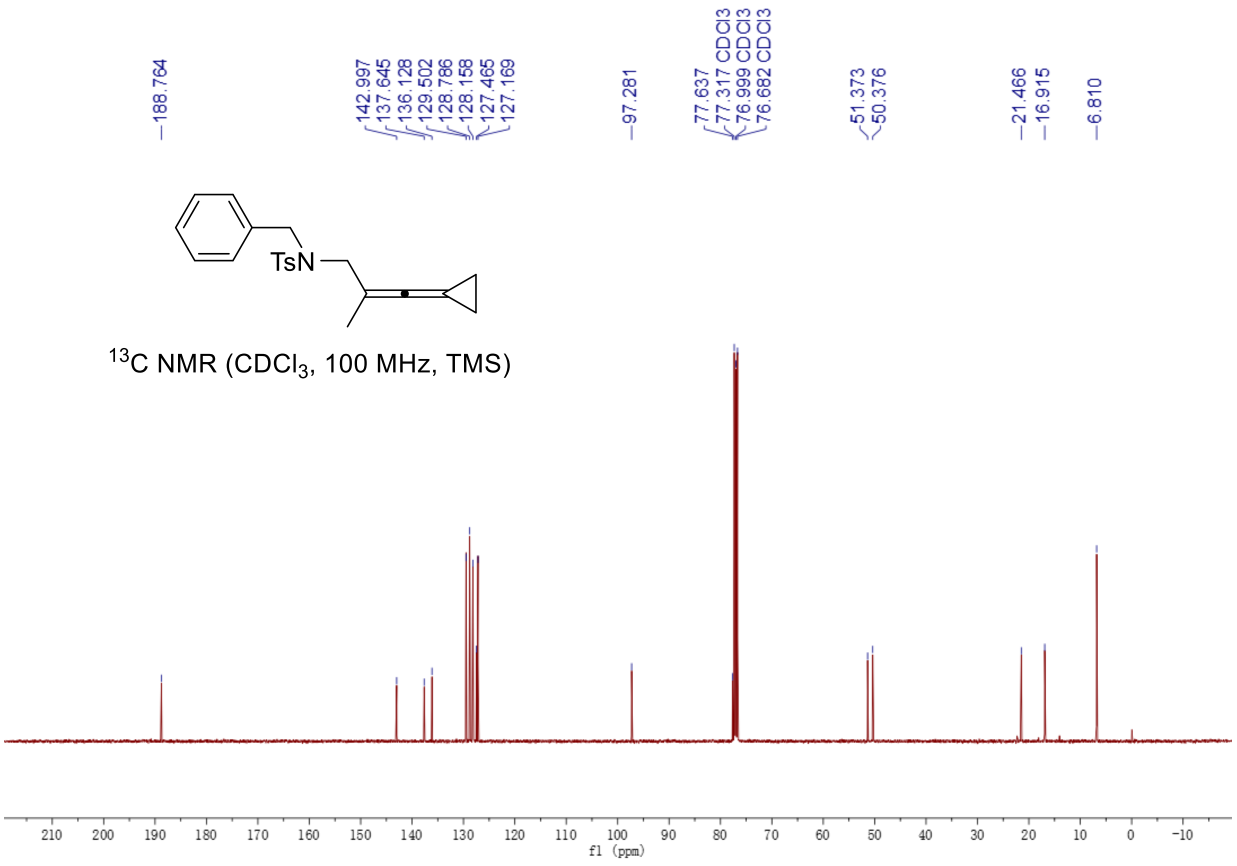

188.764
142.997
137.645
136.128
129.502
128.786
128.158
127.465
127.169
97.281
77.637
77.317 CDCl3
76.999 CDCl3
76.682 CDCl3
51.373
50.376
21.466
16.915
6.810
TsN
13C NMR (CDCl3, 100 MHz, TMS)
f1 (ppm)

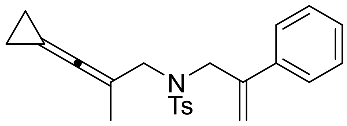

N-(3-cyclopropylidene-2-methyl-3λ^5-allyl)-4-methyl-N-(2-phenylallyl)benzenesulfonamide (1e)

This is a known compound and its spectroscopic data are consistent with those reported in the previous literature,[1] 46% yield, 174.6 mg. ^{1}H NMR (CDCl$_3$, TMS, 400 MHz) δ 7.62 (d, *J* = 8.4 Hz, 2H), 7.22 - 7.34 (m, 7H), 5.36 (s, 1H), 5.20 (s, 1H), 4.26 (s, 2H), 3.79 (s, 2H), 2.41 (s, 3H), 1.54 (s, 3H), 1.38 - 1.42 (m, 2H), 1.29 - 1.32 (m, 2H). ^{13}C NMR (CDCl$_3$, TMS, 100 MHz) δ 188.7, 142.9, 142.8, 139.1, 137.2, 129.4, 128.2, 127.7, 127.2, 126.6, 116.1, 97.7, 77.6, 51.1, 51.6, 50.8, 21.4, 17.0, 6.6.

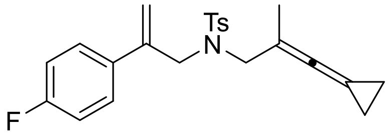

N-(3-cyclopropylidene-2-methyl-3λ^5-allyl)-N-(2-(4-fluorophenyl)allyl)-4-methylbenzenesulfonamide (1f)

This is a known compound and its spectroscopic data are consistent with those reported in the previous literature,[1] 41% yield, 163.0 mg. ^{1}H NMR (CDCl$_3$, TMS, 400 MHz) δ 7.62 (d, *J* = 8.0 Hz, 2H), 7.29 - 7.32 (m, 2H), 7.24 (d, *J* = 8.0 Hz, 2H), 6.94 (t, *J* = 8.4 Hz, 2H), 5.31 (s, 1H), 5.19 (s, 1H), 4.22 (s, 2H), 3.77 (s, 2H), 2.42 (s, 3H), 1.52 (s, 3H), 1.39 - 1.42 (m, 2H), 1.30 - 1.33 (m, 2H). ^{13}C NMR (CDCl$_3$, TMS, 100 MHz) δ 188.7, 162.4 (d, *J* = 245.0 Hz), 143.0, 142.0, 137.1, 135.0 (d, *J* = 2.9 Hz), 129.4, 128.3 (d, *J* = 8.0 Hz), 127.2, 116.2, 115.0 (d, *J* = 20.0 Hz), 97.7, 77.5, 51.7, 51.2, 21.4, 16.9, 6.6. ^{19}F NMR (376 MHz, CDCl$_3$) δ -114.5 (s).

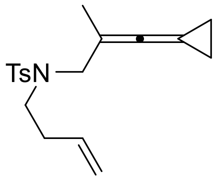

N-(but-3-en-1-yl)-N-(3-cyclopropylidene-2-methyl-3λ^5-allyl)-4-methylbenzenesulfonamide (1g)

This is a known compound and its spectroscopic data are consistent with those reported in the previous literature,[7] 53% yield, 168.2 mg. ^{1}H NMR (CDCl$_3$, TMS, 400 MHz) δ 7.69 (d, *J* = 8.4 Hz, 2H), 7.28 (d, *J* = 8.4 Hz, 2H), 5.62 - 5.73 (m, 1H), 4.99 - 5.03 (m, 1H), 4.97 - 4.99 (m, 1H), 3.81 (m,

2H), 3.18 - 3.22 (m, 2H), 2.41 (s, 3H), 2.26 (q, *J* = 8.4 Hz, 2H), 1.73 (s, 3H), 1.43-1.47 (m, 2H), 1.36-1.41 (m, 2H). ^{13}C NMR ($CDCl_3$, TMS, 100 MHz) δ 188.7, 143.0, 137.2, 135.0, 129.5, 127.1, 116.7, 97.5, 77.5, 52.5, 46.9, 32.5, 21.4, 16.9, 6.9.

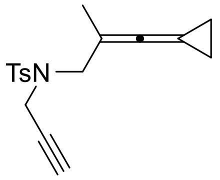

***N*-(3-cyclopropylidene-2-methyl-3λ^5-allyl)-4-methyl-*N*-(prop-2-yn-1-yl)benzenesulfonamide (1h)**

This is a known compound and its spectroscopic data are consistent with those reported in the previous literature,[8] 49% yield, 147.7 mg. ^{1}H NMR ($CDCl_3$, TMS, 400 MHz) δ 7.73 (d, *J* = 8.0 Hz, 2H), 7.28 (d, *J* = 8.0 Hz, 2H), 4.14 (s, 2H), 3.82 (s, 2H), 2.41 (s, 3H), 1.92 (t, *J* = 2.4 Hz, 1H), 1.77 (s, 3H), 1.45 - 1.48 (m, 2H), 1.39 - 1.43 (m, 2H). ^{13}C NMR ($CDCl_3$, TMS, 100 MHz) δ 188.9, 143.4, 136.1, 129.3, 127.7, 96.2, 77.7, 76.5, 73.4, 50.7, 35.4, 21.5, 16.7, 7.2.

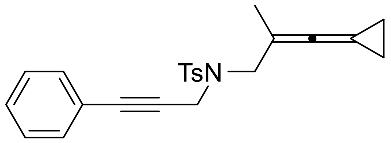

***N*-(3-cyclopropylidene-2-methyl-3λ^5-allyl)-4-methyl-*N*-(3-phenylprop-2-yn-1-yl)benzenesulfon amide (1i)**

This is a known compound and its spectroscopic data are consistent with those reported in the previous literature,[8] 43% yield, 162.3 mg. ^{1}H NMR ($CDCl_3$, TMS, 400 MHz) δ 7.78 - 7.76 (m, 2H), 7.26 - 7.20 (m, 5H) 7.00 - 6.98 (m, 2H), 4.35 (s, 2H), 3.89 (s, 2H), 2.31 (s, 3H), 1.83 (s, 3H), 1.48 - 1.43 (m, 2H), 1.42 - 1.36 (m, 2H). ^{13}C NMR ($CDCl_3$, TMS, 100 MHz) δ 189.0, 143.3, 136.1, 131.4, 129.4, 128.2, 128.0, 127.8, 122.4, 96.4, 85.4, 81.8, 51.0, 36.4, 21.4, 16.8, 7.2.

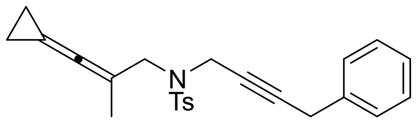

***N*-(3-cyclopropylidene-2-methyl-3λ^5-allyl)-4-methyl-*N*-(4-phenylbut-2-yn-1-yl)benzenesulfona mide (1j)**

This is a known compound and its spectroscopic data are consistent with those reported in the previous literature,[8] 48% yield, 187.9 mg. ^{1}H NMR ($CDCl_3$, TMS, 400 MHz) δ 7.72 (d, J = 8.0 Hz, 2H), 7.16 - 7.25 (m, 3H), 7.15 (d, J = 8.0 Hz, 2H), 7.04 (d, J = 8.4 Hz, 2H), 4.18 (s, 2H), 3.83 (s, 2H), 3.26 (s, 2H), 2.30 (s, 3H), 1.78 (s, 3H), 1.41 - 1.44 (m, 2H), 1.30 - 1.32 (m, 2H); ^{13}C NMR ($CDCl_3$, TMS, 100 MHz) δ 188.9, 143.1, 136.19, 136.16, 129.2, 128.3, 127.8, 127.6, 126.5, 96.4, 83.1, 77.5, 74.6, 50.8, 35.9, 24.8, 21.4, 16.7, 7.1.

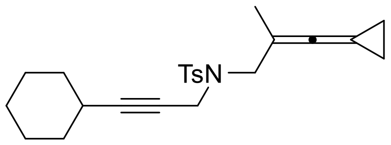

_N_-(3-cyclohexylprop-2-yn-1-yl)-_N_-(3-cyclopropylidene-2-methyl-3λ^5-allyl)-4-methylbenzenesulfonamide (1k)

This is a known compound and its spectroscopic data are consistent with those reported in the previous literature,[8] 40% yield, 153.4 mg. ^{1}H NMR ($CDCl_3$, TMS, 400 MHz) δ 7.73 (d, J = 8.4 Hz, 2H), 7.27 (d, J = 7.2 Hz, 2H), 4.13 (d, J = 2.0 Hz, 2H), 3.83 (s, 2H), 2.40 (s, 3H), 2.05 (s, 1H), 1.79 (s, 3H), 1.38 - 1.49 (m, 9H), 1.02 - 1.18 (m, 5H); ^{13}C NMR ($CDCl_3$, TMS, 100 MHz) δ 188.9, 143.0, 136.4, 129.3, 127.7, 96.5, 90.0, 77.4, 72.1, 50.5, 36.0, 32.1, 28.5, 25.8, 24.4, 21.4, 16.8, 7.1.

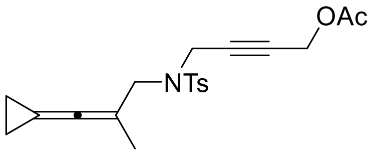

4-((_N_-(3-cyclopropylidene-2-methyl-3λ^5-allyl)-4-methylphenyl)sulfonamido)but-2-yn-1-yl acetate (1l)

This is a known compound and its spectroscopic data are consistent with those reported in the previous literature,[8] 33% yield, 123.3 mg. ^{1}H NMR ($CDCl_3$, TMS, 400 MHz) δ 7.73 (d, J = 8.0 Hz, 2H), 7.29 (d, J = 7.6 Hz, 2H), 4.33 (s, 2H), 4.16 (s, 2H), 3.79 (s, 2H), 2.42 (s, 3H), 2.04 (s, 3H), 1.77 (s, 3H), 1.46 - 1.49 (m, 2H), 1.40 - 1.43 (m, 2H); ^{13}C NMR ($CDCl_3$, TMS, 100 MHz) δ 188.8, 169.8, 143.3, 136.0, 129.2, 127.7, 96.2, 79.4, 79.1, 77.6, 51.7, 50.9, 35.6, 21.4, 20.5, 16.7, 7.1.

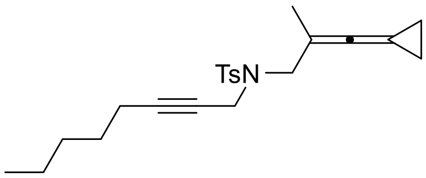

***N*-(3-cyclopropylidene-2-methyl-3λ^5-allyl)-4-methyl-*N*-(oct-2-yn-1-yl)benzenesulfonamide (1m)**

This is a known compound and its spectroscopic data are consistent with those reported in the previous literature,[8] 45% yield, 167.2 mg. ^{1}H NMR ($CDCl_3$, TMS, 400 MHz) δ 7.73 (d, *J* = 7.2 Hz, 2H), 7.27 (d, *J* = 8.0 Hz, 2H), 4.11 (s, 2H), 3.81 (s, 2H), 2.40 (s, 3H), 1.83 (t, *J* = 6.0 Hz, 2H), 1.78 (s, 3H), 1.44 - 1.48 (m, 2H), 1.38 - 1.42 (m, 2H), 1.12 - 1.22 (m, 6H), 0.84 (t, *J* = 6.8 Hz, 3H); ^{13}C NMR ($CDCl_3$, TMS, 100 MHz) δ 188.8, 143.0, 136.3, 129.1, 127.8, 96.5, 85.8, 77.4, 72.2, 50.5, 35.9, 30.7, 27.9, 22.0, 21.4, 18.3, 16.7, 13.8, 7.1.

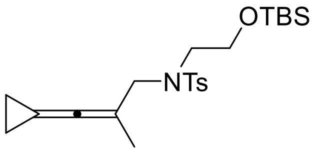

***N*-(2-((tert-butyldimethylsilyl)oxy)ethyl)-*N*-(3-cyclopropylidene-2-methyl-3λ^5-allyl)-4-methylbenzenesulfonamide (1n)**

A colorless oil, 44%, 185.5 mg. ^{1}H NMR ($CDCl_3$, TMS, 400 MHz) δ 7.71 (d, *J* = 8.2 Hz, 2H), 7.30 - 7.27 (m, 2H), 3.86 (s, 2H), 3.73 (t, *J* = 6.9 Hz, 2H), 3.23 (t, *J* = 6.9 Hz, 2H), 2.41 (s, 3H), 1.72 (s, 3H), 1.46 - 1.42 (m, 2H), 1.38 - 1.35 (m, 2H), 0.85 (s, 9H), 0.01 (s, 6H). ^{13}C NMR ($CDCl_3$, TMS, 100 MHz) δ 188.6, 143.0, 137.2, 129.5, 127.2, 97.9, 77.7, 61.7, 53.7, 49.0, 31.9, 29.7, 29.4, 25.8, 22.7, 21.5, 18.2, 16.9, 14.1, 7.0, -5.5. IR (neat) ν 2921, 2851, 1710, 1609, 1586, 1431, 1317, 1061, 1024, 998, 916 cm^{-1}. HRMS (ESI) calcd. for $C_{22}H_{35}NO_3NaSiS$ (M+Na): 444.1999, Found: 444.1996.

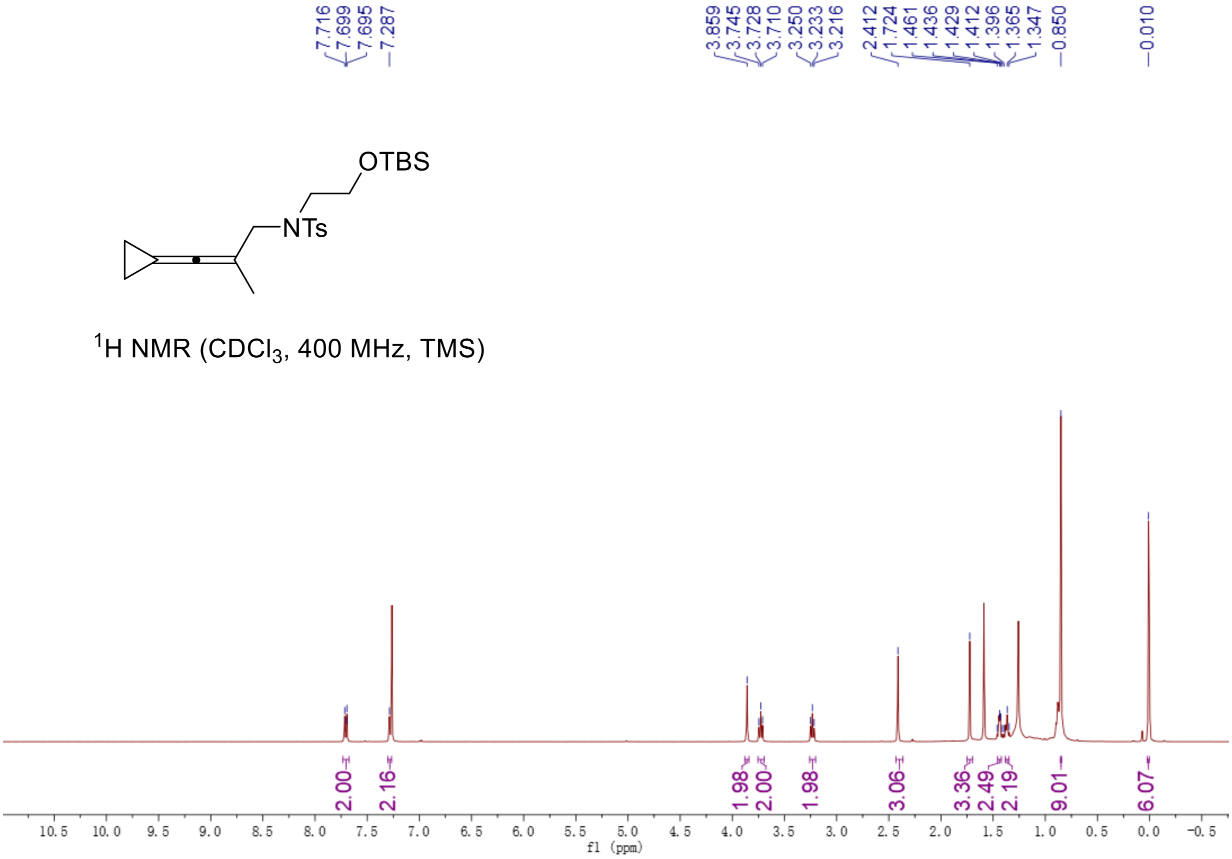
7.716
7.699
7.695
7.287
3.859
3.745
3.728
3.710
3.250
3.233
3.216
2.412
1.724
1.461
1.436
1.429
1.412
1.396
1.365
1.347
0.850
-0.010
OTBS
NTs
^{1}H NMR ($CDCl_3$, 400 MHz, TMS)
2.00
2.16
1.98
2.00
1.98
3.06
3.36
2.49
2.19
9.01
6.07
f1 (ppm)

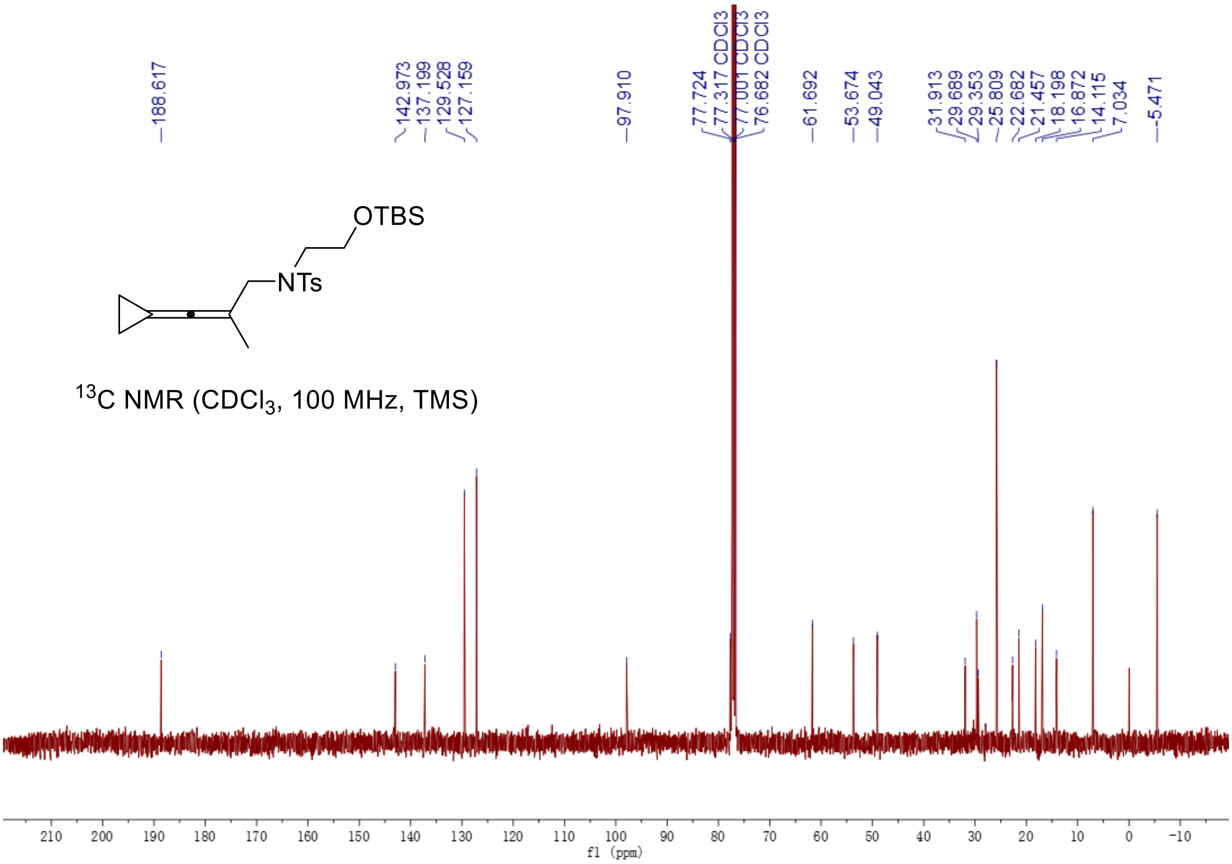
188.617
142.973
137.199
129.528
127.159
97.910
77.724
77.317 CDCl3
77.001 CDCl3
76.682 CDCl3
61.692
53.674
49.043
31.913
29.689
29.353
25.809
22.682
21.457
18.198
16.872
14.115
7.034
-5.471
OTBS
NTs
^{13}C NMR ($CDCl_3$, 100 MHz, TMS)
f1 (ppm)

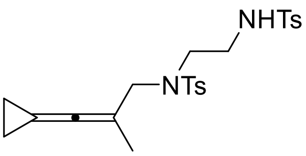

N-(3-cyclopropylidene-2-methyl-3λ⁵-allyl)-4-methyl-N-(2-((4-methylphenyl)sulfonamido)ethyl) benzenesulfonamide (1o)

A colorless oil, 40% yield, 184.2 mg. ^{1}H NMR ($CDCl_3$, TMS, 400 MHz) δ 7.76 (d, *J* = 8.3 Hz, 2H), 7.62 (d, *J* = 8.3 Hz, 2H), 7.34 - 7.25 (m, 4H), 5.14 (s, 1H), 3.67 (s, 2H), 3.19 - 3.10 (m, 4H), 2.43 (d, *J* = 7.4 Hz, 6H), 1.49 - 1.33 (m, 4H). ^{13}C NMR ($CDCl_3$, TMS, 150 MHz) δ 188.6, 143.6, 143.4, 136.8, 135.7, 129.73, 129.66, 127.20, 127.16, 97.3, 78.2, 54.0, 47.6, 42.2, 21.48, 21.47, 16.8, 7.2. IR (neat) ν 3026, 2964, 1712, 1644, 1359, 1316, 1219, 1090, 774, 698 cm^{-1}. HRMS (ESI) calcd. for $C_{23}H_{28}N_2O_4S_2Na$ (M+Na): 483.1383, Found: 483.1382.

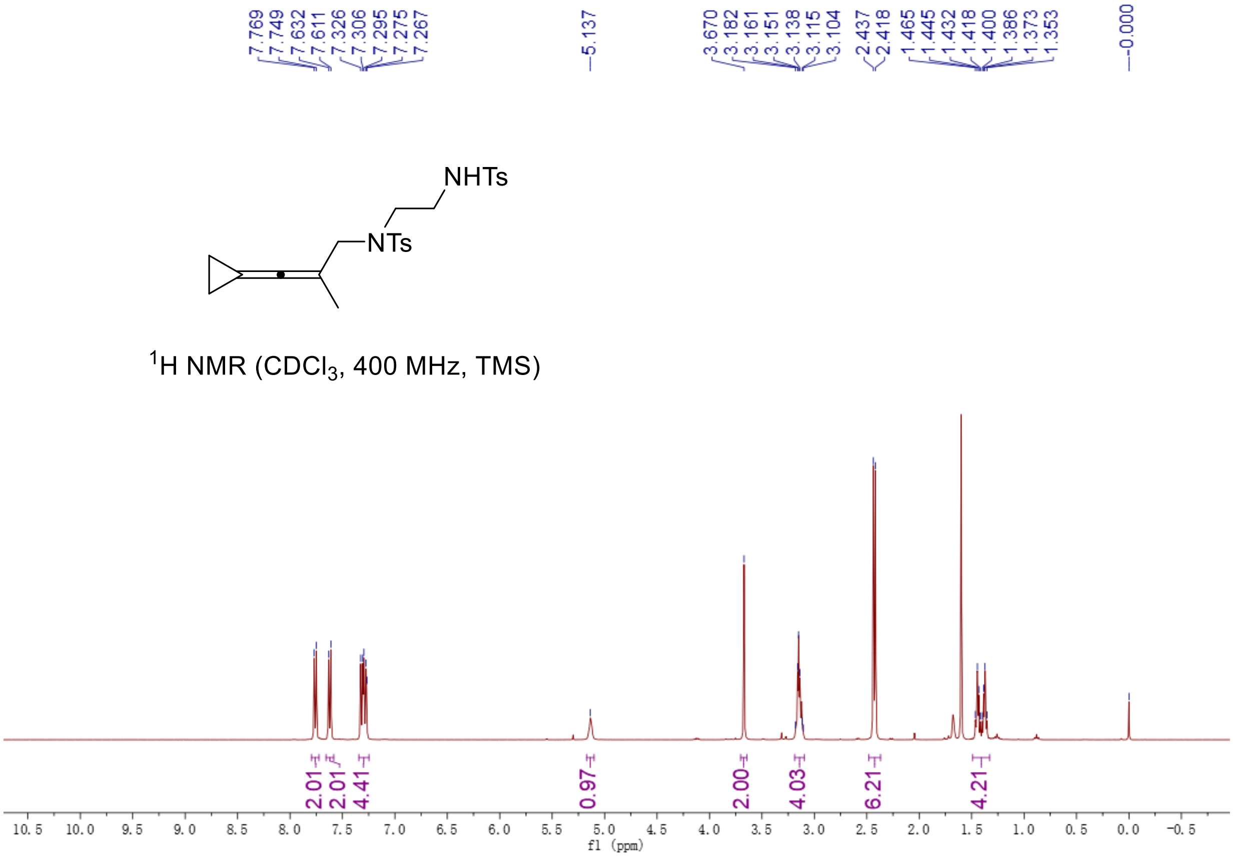

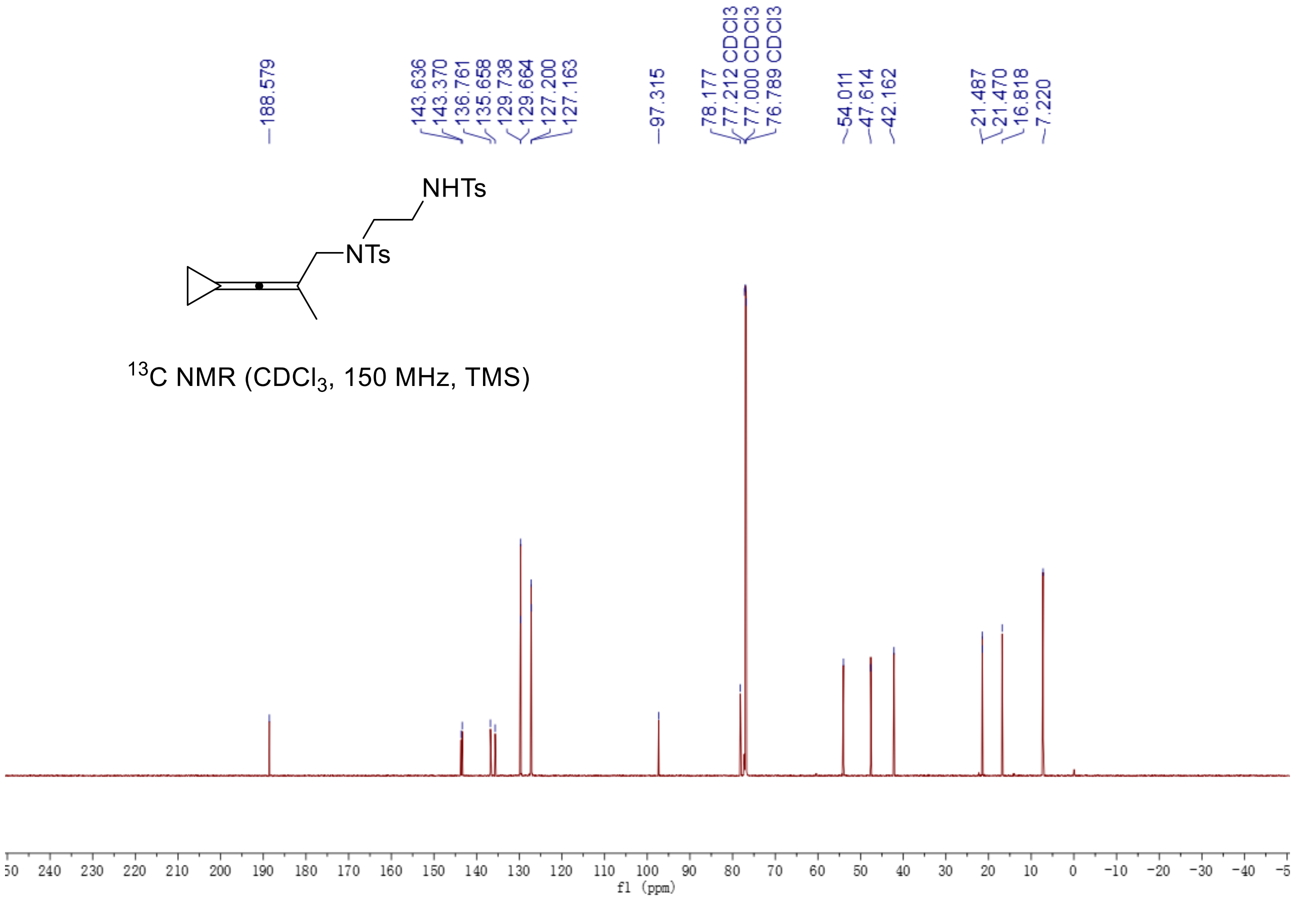

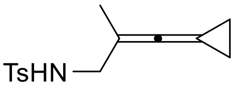

N-(3-cyclopropylidene-2-methyl-3λ^5-allyl)-4-methylbenzenesulfonamide (1p)

A colorless oil, 38% yield, 100.1 mg. ^{1}H NMR ($CDCl_3$, TMS, 400 MHz) δ 7.74 (d, *J* = 8.2 Hz, 2H), 7.30 (d, *J* = 8.0 Hz, 2H), 4.48 (s, 1H), 3.53 (d, *J* = 5.3 Hz, 2H), 2.43 (s, 3H), 1.69 (s, 3H), 1.45 (s, 4H). ^{13}C NMR ($CDCl_3$, TMS, 100 MHz) δ 185.8, 143.3, 136.9, 129.6, 127.1, 98.8, 80.6, 46.1, 21.5, 17.3, 7.1. IR (neat) ν 2917, 1694, 1511, 1477, 1464, 1396, 317, 1169, 1048, 964, 833 cm^{-1}. HRMS (ESI) calcd. for $C_{14}H_{17}NO_2NaS$ (M+Na): 286.0872, Found: 286.0873.

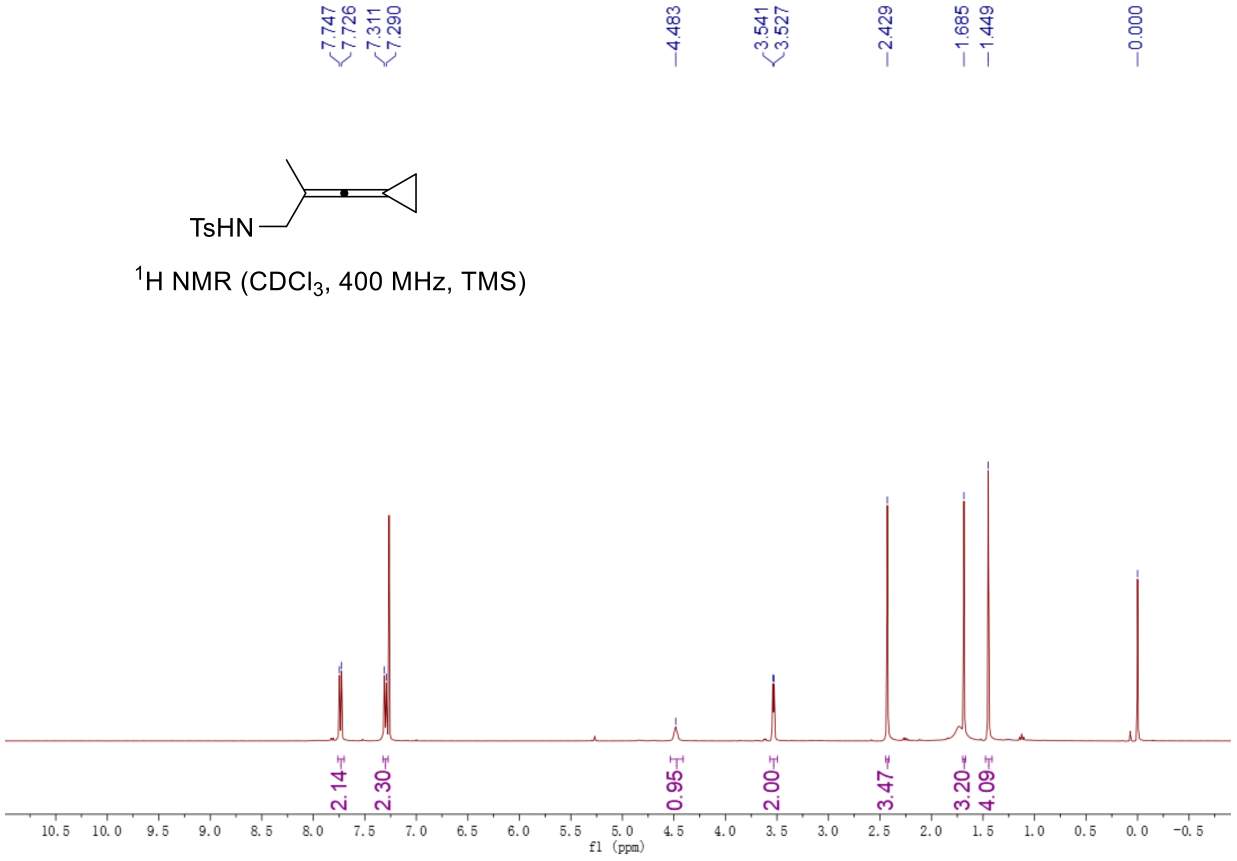
7.747
7.726
7.311
7.290
4.483
3.541
3.527
2.429
1.685
1.449
0.000
TsHN
1H NMR (CDCl3, 400 MHz, TMS)
2.14
2.30
0.95
2.00
3.47
3.20
4.09
10.5 10.0 9.5 9.0 8.5 8.0 7.5 7.0 6.5 6.0 5.5 5.0 4.5 4.0 3.5 3.0 2.5 2.0 1.5 1.0 0.5 0.0 -0.5
f1 (ppm)

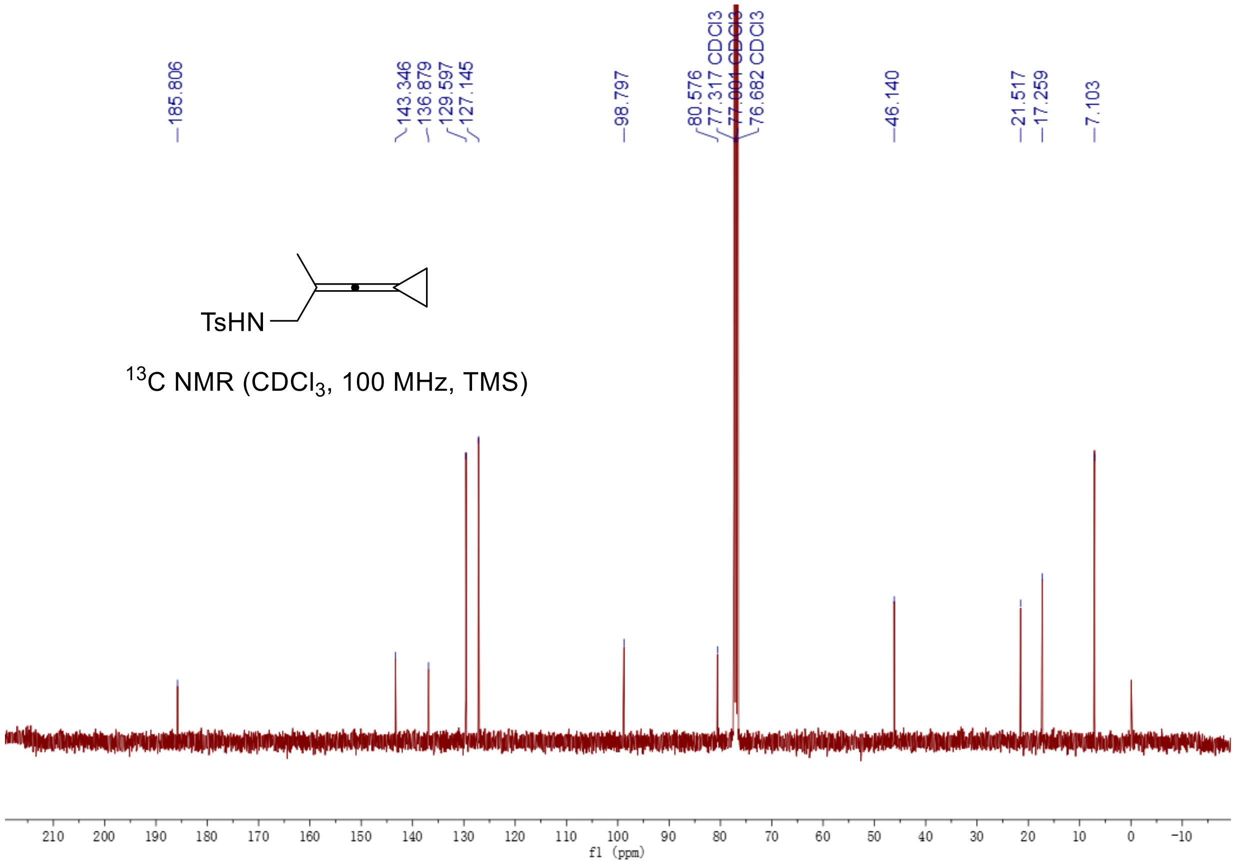
185.806
143.346
136.879
129.597
127.145
98.797
80.576
77.317 CDCl3
77.001 CDCl3
76.682 CDCl3
46.140
21.517
17.259
7.103
TsHN
13C NMR (CDCl3, 100 MHz, TMS)
210 200 190 180 170 160 150 140 130 120 110 100 90 80 70 60 50 40 30 20 10 0 -10
f1 (ppm)

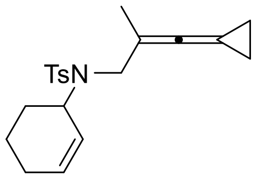

***N*-(cyclohex-2-en-1-yl)-*N*-(3-cyclopropylidene-2-methyl-3λ⁵-allyl)-4-methylbenzenesulfonamide (1q)**

A colorless oil, 22% yield, 75.6 mg. ^{1}H NMR ($CDCl_3$, TMS, 400 MHz) δ 7.71 (d, *J* = 8.3 Hz, 2H), 7.27 (d, *J* = 7.9 Hz, 2H), 5.80 - 5.70 (m, 1H), 5.06 (d, *J* = 10.2 Hz, 1H), 4.50 - 4.40 (m, 1H), 3.94 (d, *J* = 15.6 Hz, 1H), 3.64 (d, *J* = 15.6 Hz, 1H), 2.41 (s, 3H), 1.95 - 1.88 (m, 2H), 1.85 - 1.69 (m, 6H), 1.58 - 1.50 (m, 1H), 1.47 - 1.37 (m, 4H). ^{13}C NMR ($CDCl_3$, TMS, 100 MHz) δ 188.0, 142.8, 138.4, 132.0, 129.5, 127.3, 127.1, 100.8, 78.5, 55.2, 48.1, 28.4, 24.5, 21.9, 21.5, 17.0, 6.6, 6.5. IR (neat) ν 2983, 1613, 1512, 1463, 1398, 1385, 1318, 1220, 1033, 765, 713 cm^{-1}. HRMS (ESI) calcd. for $C_{20}H_{25}NO_2NaS$ (M+Na): 366.1498, Found: 366.1493.

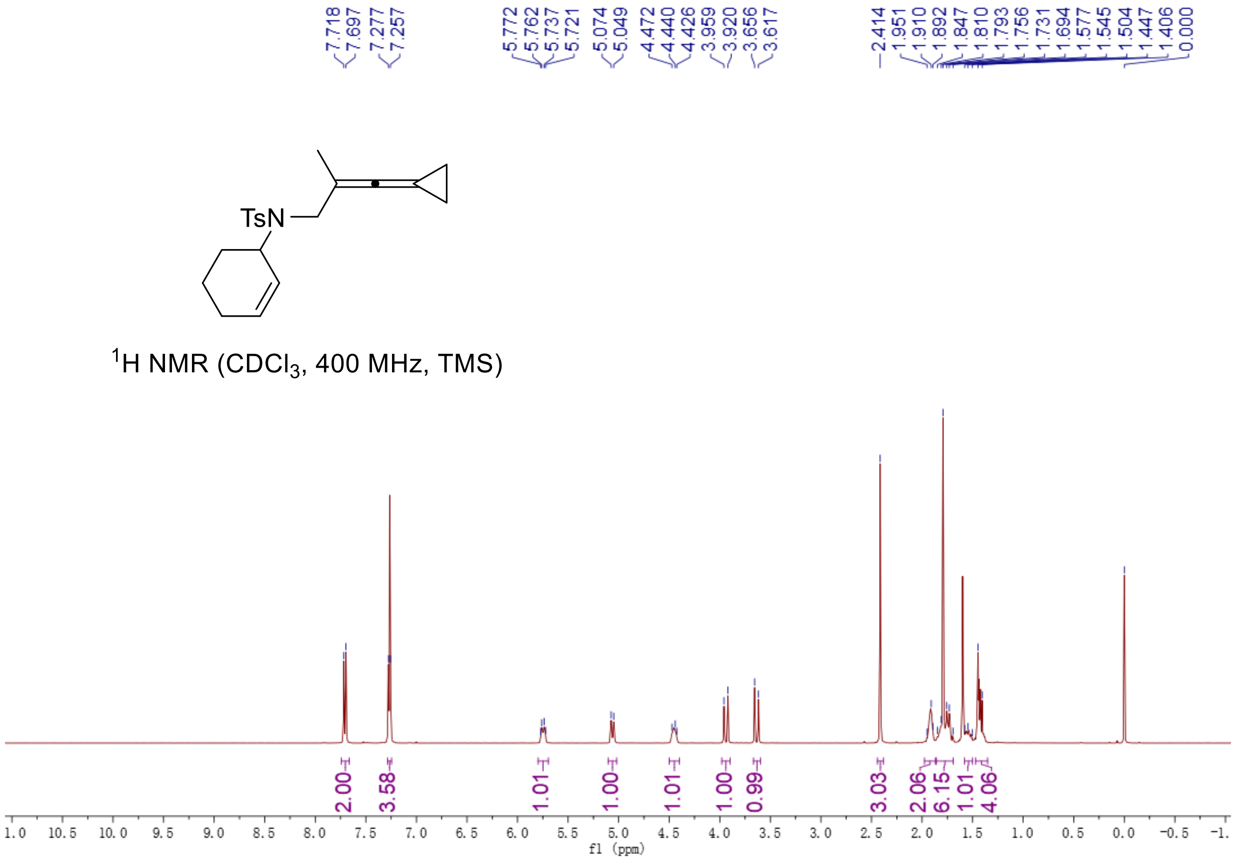

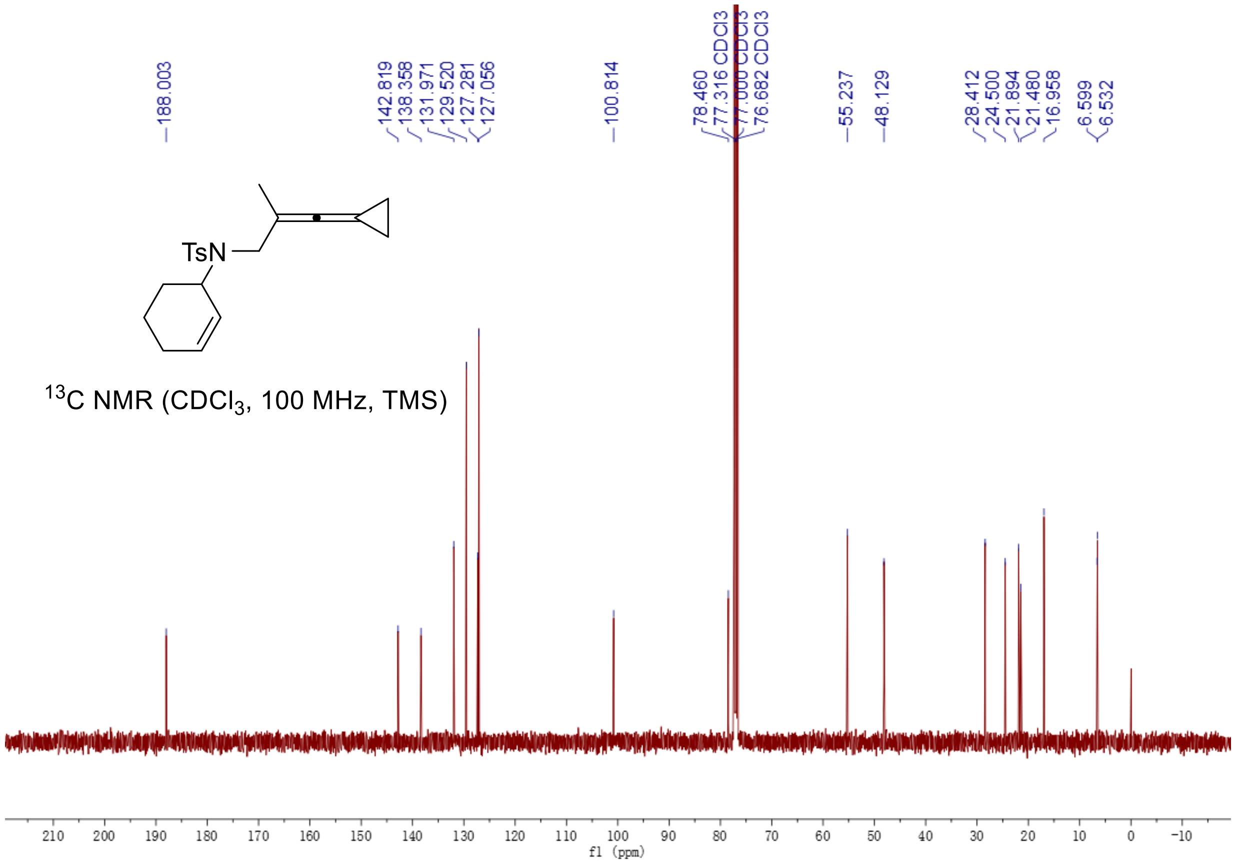

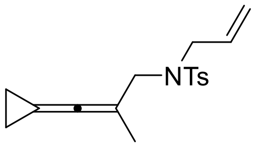

***N*-allyl-*N*-(3-cyclopropylidene-2-methyl-3λ^5-allyl)-4-methylbenzenesulfonamide (1r)**

This is a known compound and its spectroscopic data are consistent with those reported in the previous literature,[7] 51%, 154.7 mg. ^{1}H NMR ($CDCl_3$, TMS, 400 MHz) δ 1.36-1.41 (m, 2H), 1.43-1.47 (m, 2H), 1.73 (s, 3H), 2.26 (q, J = 8.4 Hz, 2H), 2.41 (s, 3H), 3.18-3.22 (m, 2H), 3.81 (m, 2H), 4.97-4.99 (m, 1H), 4.99-5.03 (m, 1H), 5.62-5.73 (m, 1H), 7.28 (d, J = 8.4 Hz, 2H), 7.69 (d, J = 8.4 Hz, 2H). ^{13}C NMR ($CDCl_3$, TMS, 100 MHz) δ 6.9, 16.9, 21.4, 32.5, 46.9, 52.5, 77.5, 97.5, 116.7, 127.1, 129.5, 135.0, 137.2, 143.0, 188.7.

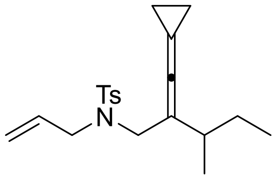

***N*-allyl-*N*-(2-(cyclopropylidene-λ^5-methylene)-3-methylpentyl)-4-methylbenzenesulfonamide**

(1s)

This is a known compound and its spectroscopic data are consistent with those reported in the previous literature,[9] 46% yield, 159.0 mg. ^{1}H NMR ($CDCl_3$, TMS, 400 MHz) δ 7.70 (d, *J* = 8.2 Hz, 2H), 7.27 (d, *J* = 8.0 Hz, 2H), 5.58 - 5.48 (m, 1H), 5.13 - 5.06 (m, 2H), 3.86 - 3.81 (m, 4H), 2.42 (s, 3H), 2.03 - 1.98 (m, 1H), 1.50 - 1.40 (m, 3H), 1.37 - 1.24 (m, 3H), 1.00 (d, *J* = 6.8 Hz, 3H), 0.86 (t, *J* = 7.4 Hz, 3H). ^{13}C NMR ($CDCl_3$, TMS, 100 MHz) δ 187.9, 142.9, 137.7, 132.4, 129.5, 127.2, 119.1, 107.1, 79.6, 49.0, 48.6, 35.1, 28.2, 21.5, 19.0, 11.5, 6.9, 6.8.

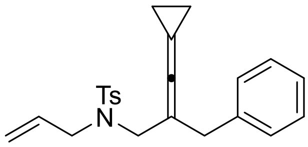

***N*-allyl-*N*-(2-benzyl-3-cyclopropylidene-3λ^5-allyl)-4-methylbenzenesulfonamide (1t)**

This is a known compound and its spectroscopic data are consistent with those reported in the previous literature,[7] 48% yield, 174.6 mg. ^{1}H NMR ($CDCl_3$, TMS, 400 MHz) δ 1.35-1.42 (m, 4H), 2.41 (s, 3H), 3.34 (s, 2H), 3.79 (s, 2H), 3.83 (d, *J* = 7.2 Hz, 2H), 5.03 (s, 1H), 5.07 (d, *J* = 8.0 Hz, 1H), 5.46-5.56 (m, 1H), 7.18-7.27 (m, 7H), 7.67 (d, *J* = 8.0 Hz, 2H). ^{13}C NMR ($CDCl_3$, TMS, 100 MHz) δ 7.1, 21.5, 37.0, 49.1, 49.3, 78.9, 101.5, 119.2, 126.1, 127.2, 128.1, 129.0, 129.5, 132.2, 137.5, 143.0, 189.6.

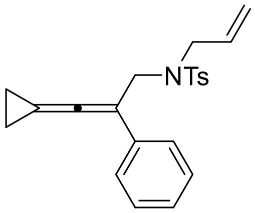

***N*-allyl-*N*-(3-cyclopropylidene-2-phenyl-3λ^5-allyl)-4-methylbenzenesulfonamide (1u)**

A colorless oil, 41% yield, 149.9 mg. ^{1}H NMR ($CDCl_3$, TMS, 400 MHz) δ 7.77 (d, *J* = 8.0 Hz, 2H), 7.43 (d, *J* = 8.0 Hz, 2H), 7.33 - 7.26 (m, 4H), 7.19 (t, *J* = 7.2 Hz, 1H), 5.62 - 5.45 (m, 1H), 5.16 - 4.99 (m, 2H), 4.32 (s, 2H), 3.90 - 3.76 (m, 2H), 2.43 (s, 3H), 1.71 - 1.62 (m, 2H), 1.56 (s, 2H). ^{13}C NMR ($CDCl_3$, TMS, 150 MHz) δ 190.5, 143.1, 137.2, 135.5, 132.3, 129.6, 128.4, 127.4, 126.7, 126.3, 119.0, 103.1, 80.3, 49.2, 47.8, 21.5, 8.4. IR (neat) ν 2996, 2929, 2008, 1596, 1442, 1091, 897, 752 cm^{-1}. HRMS (ESI) calcd. for $C_{22}H_{23}NO_2NaS$ $(M+Na)^+$: 388.1341, Found: 388.1349.

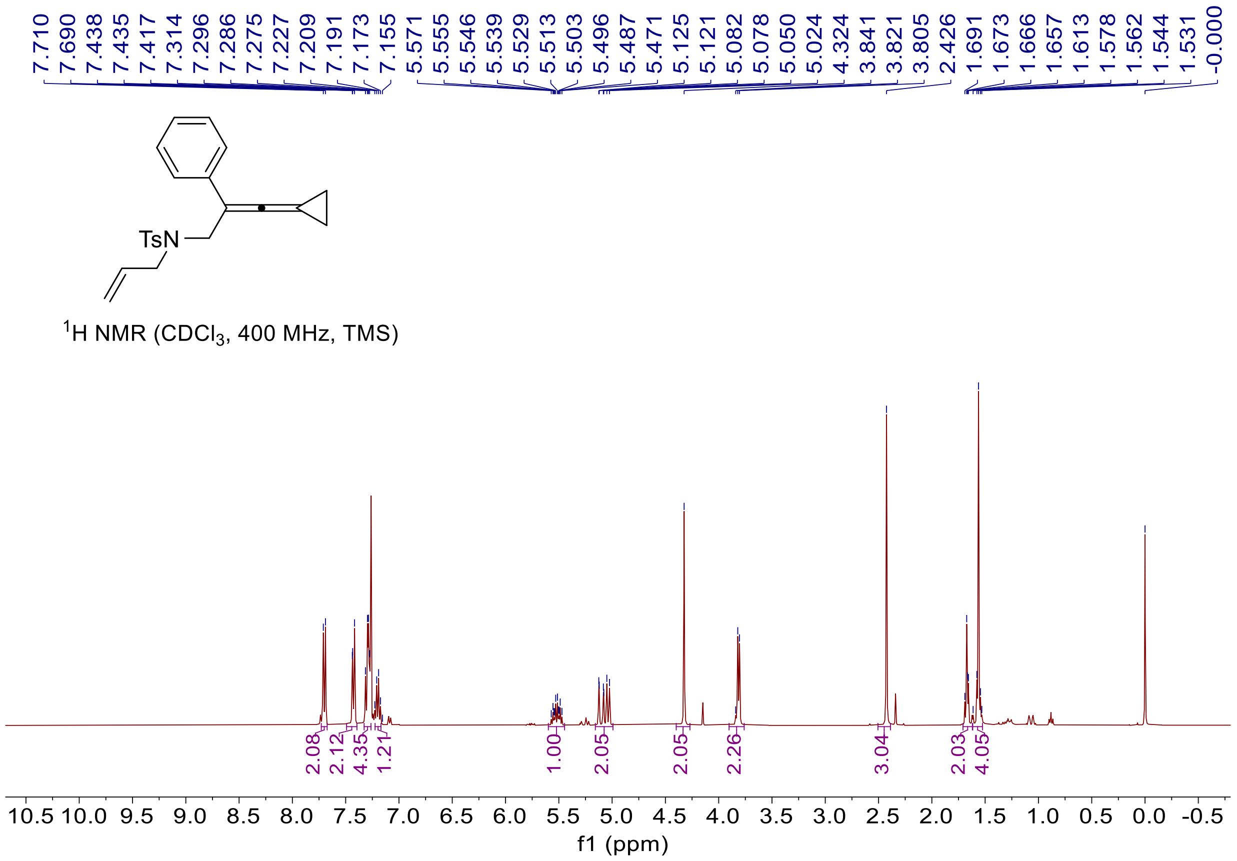
7.710 7.690 7.438 7.435 7.417 7.314 7.296 7.286 7.275 7.227 7.209 7.191 7.173 7.155 5.571 5.555 5.546 5.539 5.529 5.513 5.503 5.496 5.487 5.471 5.125 5.121 5.082 5.078 5.050 5.024 4.324 3.841 3.821 3.805 2.426 1.691 1.673 1.666 1.657 1.613 1.578 1.562 1.544 1.531 -0.000
TsN
^{1}H NMR (CDCl$_3$, 400 MHz, TMS)
2.08 2.12 4.35 1.21 1.00 2.05 2.05 2.26 3.04 2.03 4.05
10.5 10.0 9.5 9.0 8.5 8.0 7.5 7.0 6.5 6.0 5.5 5.0 4.5 4.0 3.5 3.0 2.5 2.0 1.5 1.0 0.5 0.0 -0.5
f1 (ppm)

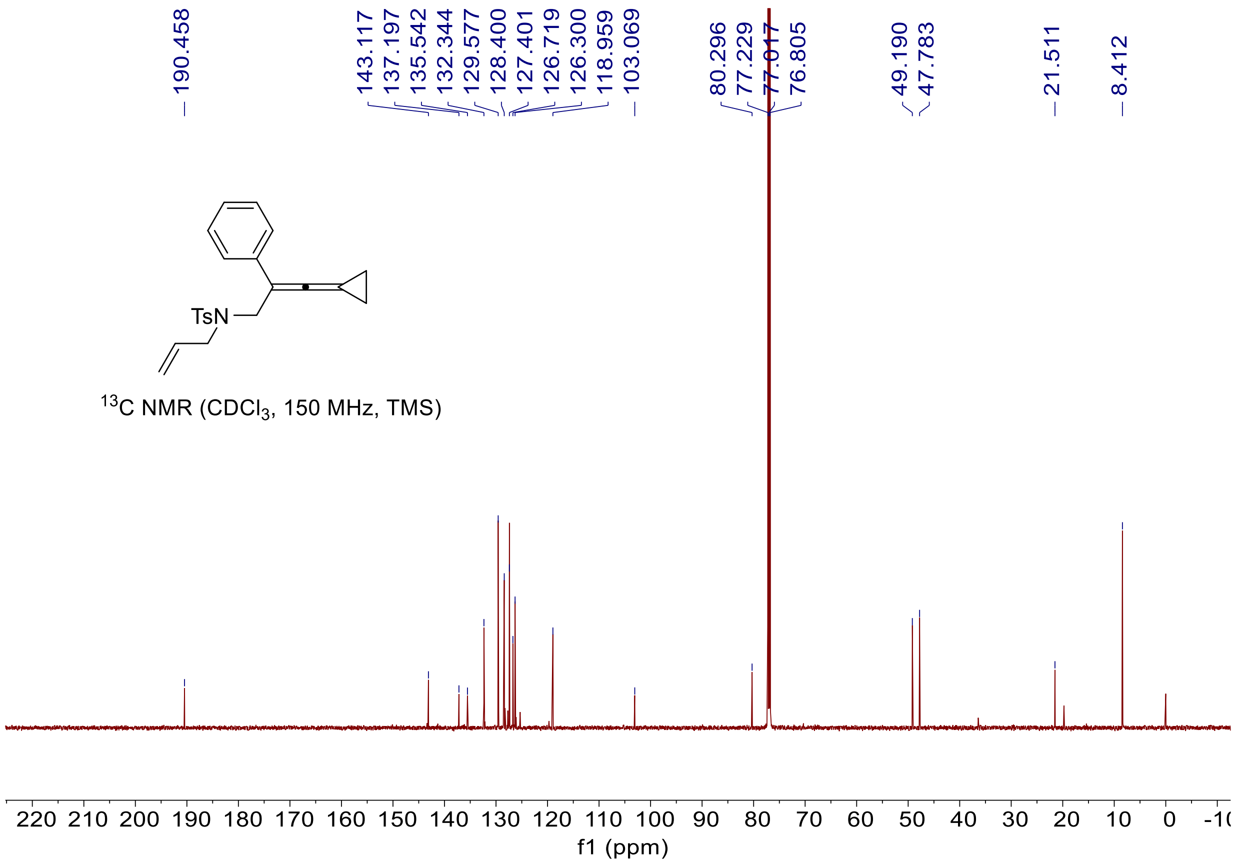
190.458 143.117 137.197 135.542 132.344 129.577 128.400 127.401 126.719 126.300 118.959 103.069 80.296 77.229 77.017 76.805 49.190 47.783 21.511 8.412
TsN
^{13}C NMR (CDCl$_3$, 150 MHz, TMS)
220 210 200 190 180 170 160 150 140 130 120 110 100 90 80 70 60 50 40 30 20 10 0 -10
f1 (ppm)

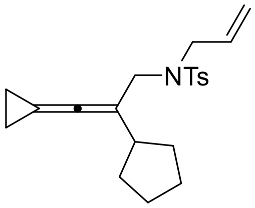

***N*-allyl-*N*-(2-cyclopentyl-3-cyclopropylidene-3λ^5-allyl)-4-methylbenzenesulfonamide (1v)**

This is a known compound and its spectroscopic data are consistent with those reported in the previous literature,[9] 51% yield, 182.3 mg. ^{1}H NMR (CDCl$_3$, TMS, 400 MHz) δ 7.70 (d, *J* = 8.0 Hz, 2H), 7.27 (d, *J* = 8.4 Hz, 2H), 5.54 (td, *J* = 16.8, 6.6 Hz, 1H), 5.14 - 5.06 (m, 2H), 3.86 - 3.82 (m, 4H), 2.42 (s, 3H), 2.39 - 2.33 (m, 1H), 1.85 - 1.77 (m, 2H), 1.65 - 1.59 (m, 2H), 1.53 - 1.50 (m, 2H), 1.46 - 1.43 (m, 2H), 1.39 - 1.33 (m, 4H). ^{13}C NMR (CDCl$_3$, TMS, 100 MHz) δ 187.1, 142.9, 137.6, 132.3, 129.5, 127.2, 125.2, 119.1, 106.6, 105.0, 79.6, 49.5, 49.1, 39.9, 31.8, 29.7, 24.9, 21.5, 6.9.

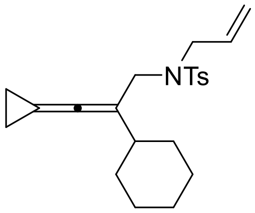

***N*-allyl-*N*-(2-cyclohexyl-3-cyclopropylidene-3λ^5-allyl)-4-methylbenzenesulfonamide (1w)**

A white solid, this is a known compound and its spectroscopic data are consistent with those reported in the previous literature,[9] 46% yield, 170.9 mg, M.P.: 87-89 °C. ^{1}H NMR (CDCl$_3$, TMS, 300 MHz) δ 1.03 - 1.25 (m, 5H), 1.33 - 1.38 (m, 2H), 1.42 - 1.46 (m, 2H), 1.61 - 1.86 (m, 6H), 2.42 (s, 3H), 3.82 (d, *J* = 6.6 Hz, 2H), 3.85 (s, 2H), 5.08 (d, *J* = 8.4 Hz, 1H), 5.11 (d, *J* = 15.3 Hz, 1H), 5.49 - 5.62 (m, 1H), 7.28 (d, *J* = 7.8 Hz, 2H), 7.70 (d, *J* = 7.8 Hz, 2H). ^{13}C NMR (CDCl$_3$, TMS, 75 MHz) δ 7.0, 21.4, 26.2, 26.3, 32.1, 37.7, 48.3, 49.0, 79.3, 107.5, 119.0, 127.2, 129.5, 132.4, 137.6, 142.9, 188.1.

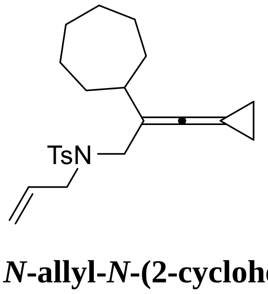

***N*-allyl-*N*-(2-cycloheptyl-3-cyclopropylidene-3λ^5-allyl)-4-methylbenzenesulfonamide (1x)**

A white solid, 47% yield, 181.2 mg, M.P.: 83-86 °C. ^{1}H NMR ($CDCl_3$, TMS, 400 MHz) δ 7.70 (d, *J* = 8.0 Hz, 2H), 7.28 (d, *J* = 8.0 Hz, 2H), 5.63 - 5.48 (m, 1H), 5.15 - 5.04 (m, 2H), 3.93 - 3.76 (m, 4H), 2.42 (s, 3H), 2.12 - 1.96 (m, 1H), 1.83 - 1.73 (m, 2H), 1.64 - 1.53 (m, 4H) 1.49 - 1.29 (m, 10H). ^{13}C NMR ($CDCl_3$, TMS, 100 MHz) δ 188.0, 143.0, 137.7, 132.5, 129.5, 127.2, 119.0, 108.6, 79.5, 49.1, 48.9, 39.4, 33.7, 28.3, 26.2, 21.5, 7.0. IR (neat) ν 2922, 2853, 2018, 1598, 1444, 1091, 905, 764 cm^{-1}. HRMS (ESI) calcd. for $C_{23}H_{31}NO_2NaS$ $(M+Na)^+$: 408.1967, Found: 408.1972.

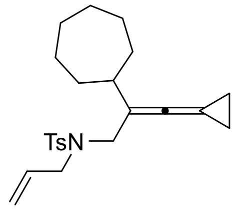

^{1}H NMR ($CDCl_3$, 400 MHz, TMS)

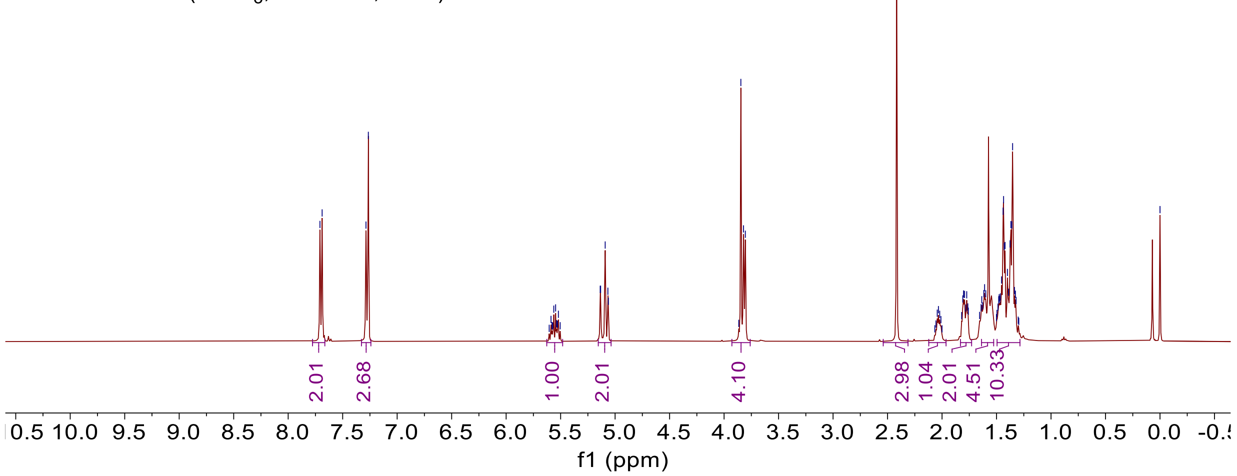

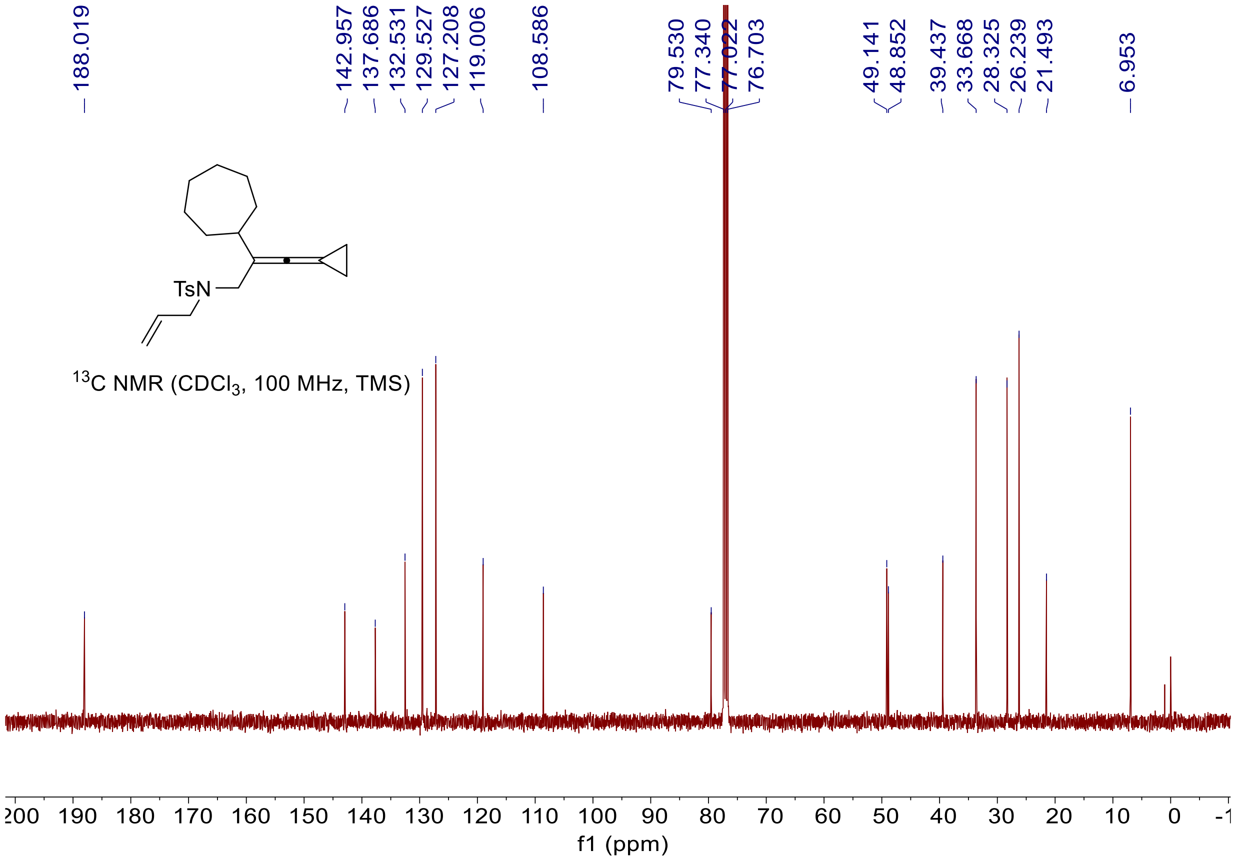

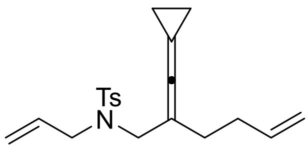

***N*-allyl-*N*-(2-(cyclopropylidene-λ^5-methylene)hex-5-en-1-yl)-4-methylbenzenesulfonamide (1y)**

A colorless oil, 39% yield, 134.0 mg. ^{1}H NMR ($CDCl_3$, TMS, 400 MHz) δ 7.70 (d, *J* = 8.2 Hz, 2H), 7.28 (d, *J* = 8.3 Hz, 2H), 5.87 - 5.72 (m, 1H), 5.61 - 5.45 (m, 1H), 5.16 - 5.04 (m, 2H), 5.03 - 4.90 (m, 2H), 3.83 (s, 4H), 2.42 (s, 3H), 2.19 - 2.11 (m, 2H), 2.11 - 2.04 (m, 2H), 1.47 - 1.36 (m, 4H). ^{13}C NMR ($CDCl_3$, TMS, 100 MHz) δ 188.5, 143.0, 138.3, 137.6, 132.2, 129.5, 127.2, 119.2, 114.6, 101.5, 79.2, 49.9, 49.1, 31.6, 29.3, 21.5, 7.0. IR (Acetone) ν 2923, 1710, 1597, 1461, 1396, 1220, 1025, 991, 890, 803, 697 cm^{-1}. HRMS (ESI) calcd. for $C_{20}H_{25}NO_2NaS$ (M+Na): 366.1492, Found: 366.1502.

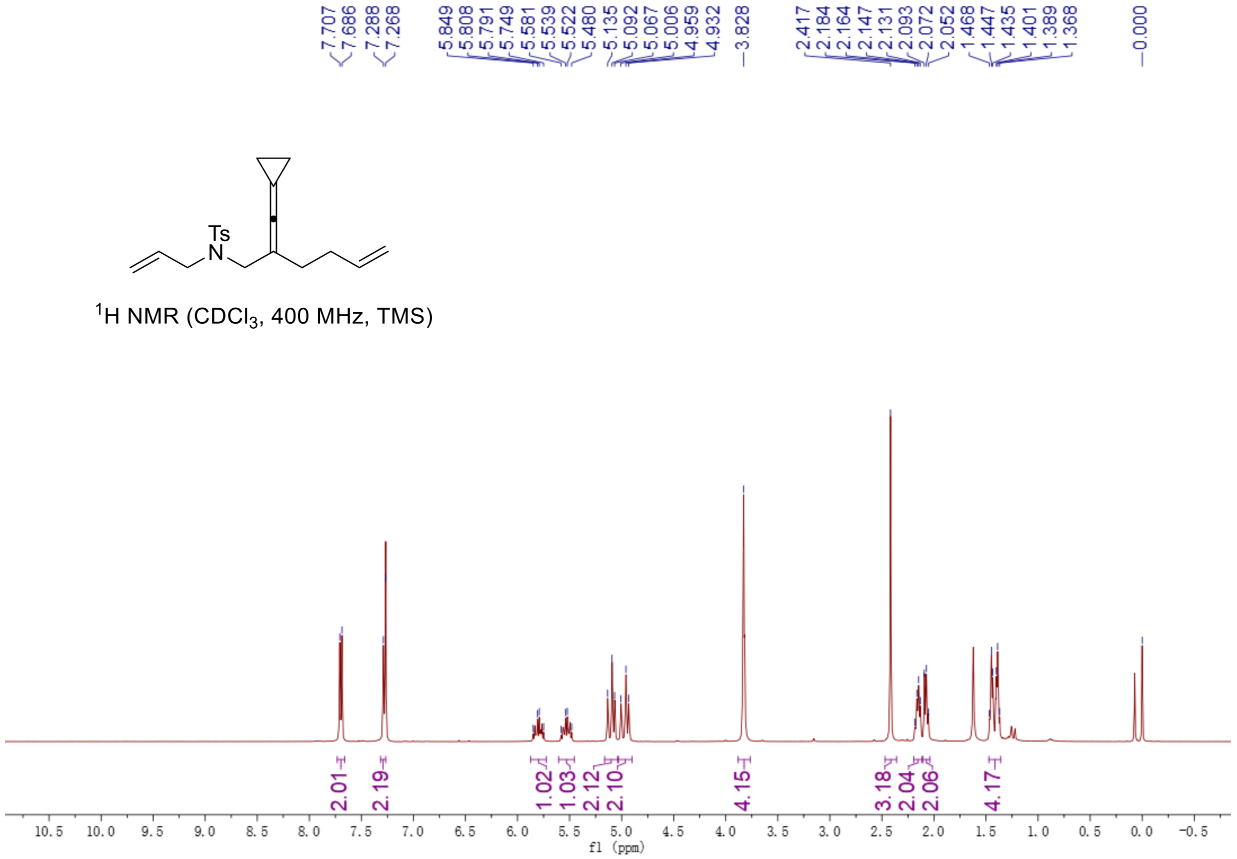
7.707
7.686
7.288
7.268
5.849
5.808
5.791
5.749
5.581
5.539
5.522
5.480
5.135
5.092
5.067
5.006
4.959
4.932
3.828
2.417
2.184
2.164
2.147
2.131
2.093
2.072
2.052
1.468
1.447
1.435
1.401
1.389
1.368
0.000
Ts
N
^{1}H NMR (CDCl$_3$, 400 MHz, TMS)
2.01
2.19
1.02
1.03
2.12
2.10
4.15
3.18
2.04
2.06
4.17
f1 (ppm)

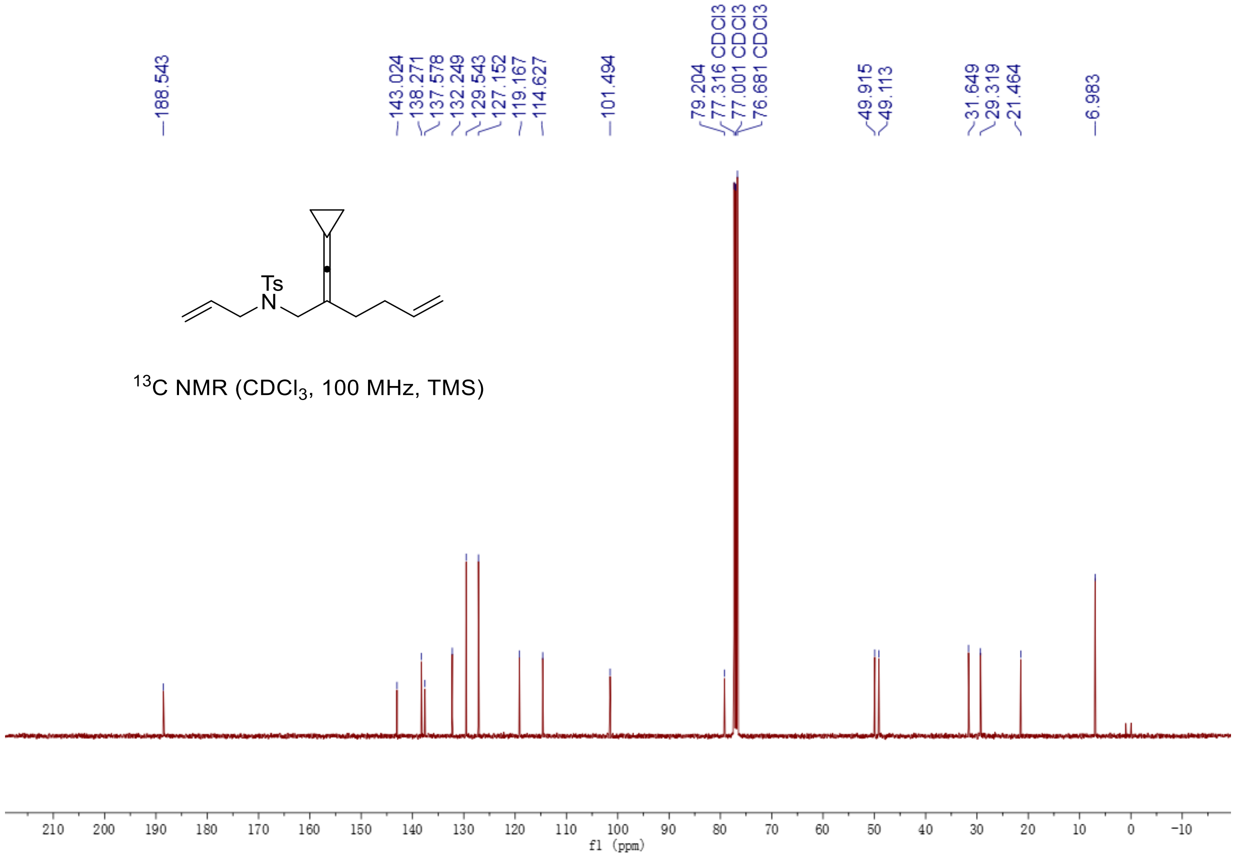
188.543
143.024
138.271
137.578
132.249
129.543
127.152
119.167
114.627
101.494
79.204
77.316 CDCl3
77.001 CDCl3
76.681 CDCl3
49.915
49.113
31.649
29.319
21.464
6.983
Ts
N
^{13}C NMR (CDCl$_3$, 100 MHz, TMS)
f1 (ppm)

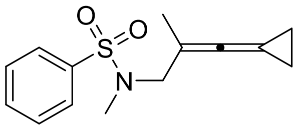

N-(3-cyclopropylidene-2-methyl-3λ^5-allyl)-N-methylbenzenesulfonamide (1aa)

A colorless oil, 50% yield, 131.7 mg. ^{1}H NMR ($CDCl_3$, TMS, 400 MHz) δ 7.79 (d, J = 7.3 Hz, 2H), 7.63 - 7.48 (m, 3H), 3.62 (s, 2H), 2.70 (s, 3H), 1.79 (s, 3H), 1.48 - 1.42 (m, 2H), 1.41 - 1.36 (m, 2H). ^{13}C NMR ($CDCl_3$, TMS, 100 MHz) δ 188.8, 137.4, 132.5, 129.0, 127.4, 97.1, 77.5, 54.7, 46.9, 33.8, 16.7, 7.0. IR (neat) ν 2921, 2855, 1711, 1488, 1396, 1332, 1219, 1034, 991, 790, 759 cm^{-1}. HRMS (ESI) calcd. for $C_{14}H_{17}NO_2NaS$ (M+Na): 286.0872, Found: 286.0873.

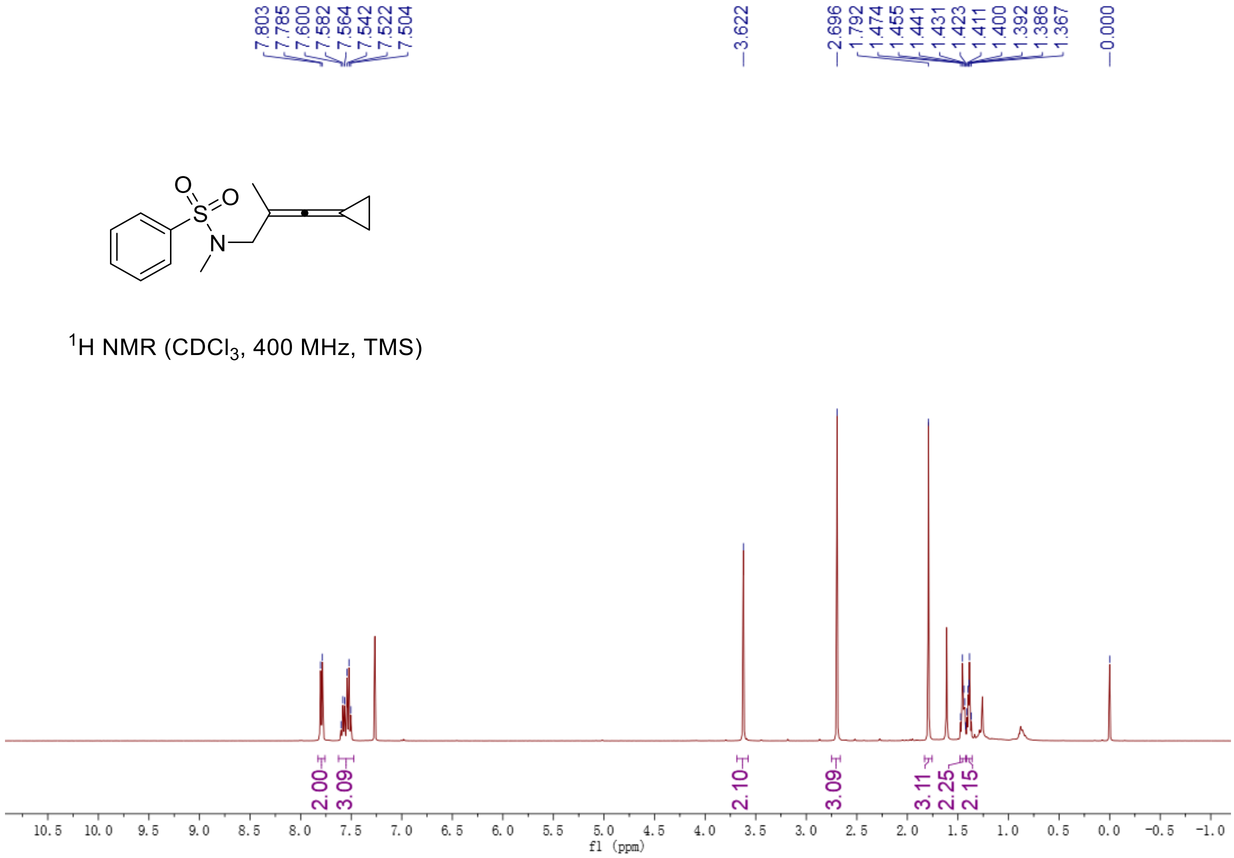

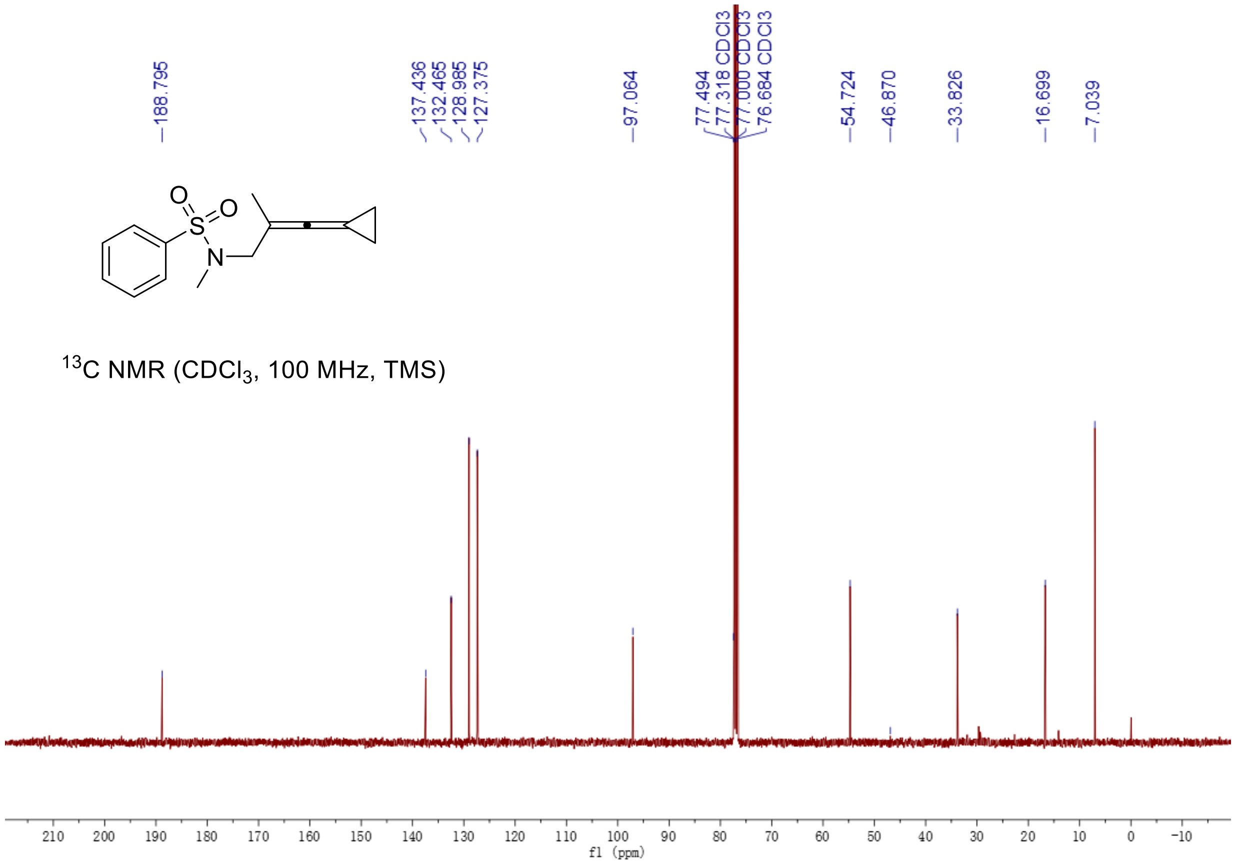

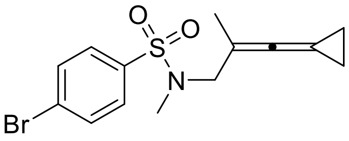

4-bromo-*N*-(3-cyclopropylidene-2-methyl-3l5-allyl)-N-methylbenzenesulfonamide (1ab)

A colorless oil, 43% yield, 147.2 mg. ^{1}H NMR ($CDCl_3$, TMS, 600 MHz) δ 7.65 (s, 4H), 3.62 (s, 2H), 2.70 (s, 3H), 1.78 (s, 3H), 1.49 - 1.37 (m, 4H). ^{13}C NMR ($CDCl_3$, TMS, 150 MHz) δ 188.8, 136.6, 132.3, 128.9, 127.4, 96.8, 77.7, 54.7, 33.8, 16.7, 7.1. IR (neat) ν 2942, 1658, 1428, 1343, 1253, 1221, 1161, 992, 945, 803, 754, 699 cm^{-1}. HRMS (ESI) calcd. for $C_{14}H_{16}NO_2NaSBr$ (M+Na): 363.9977, Found: 363.9977.

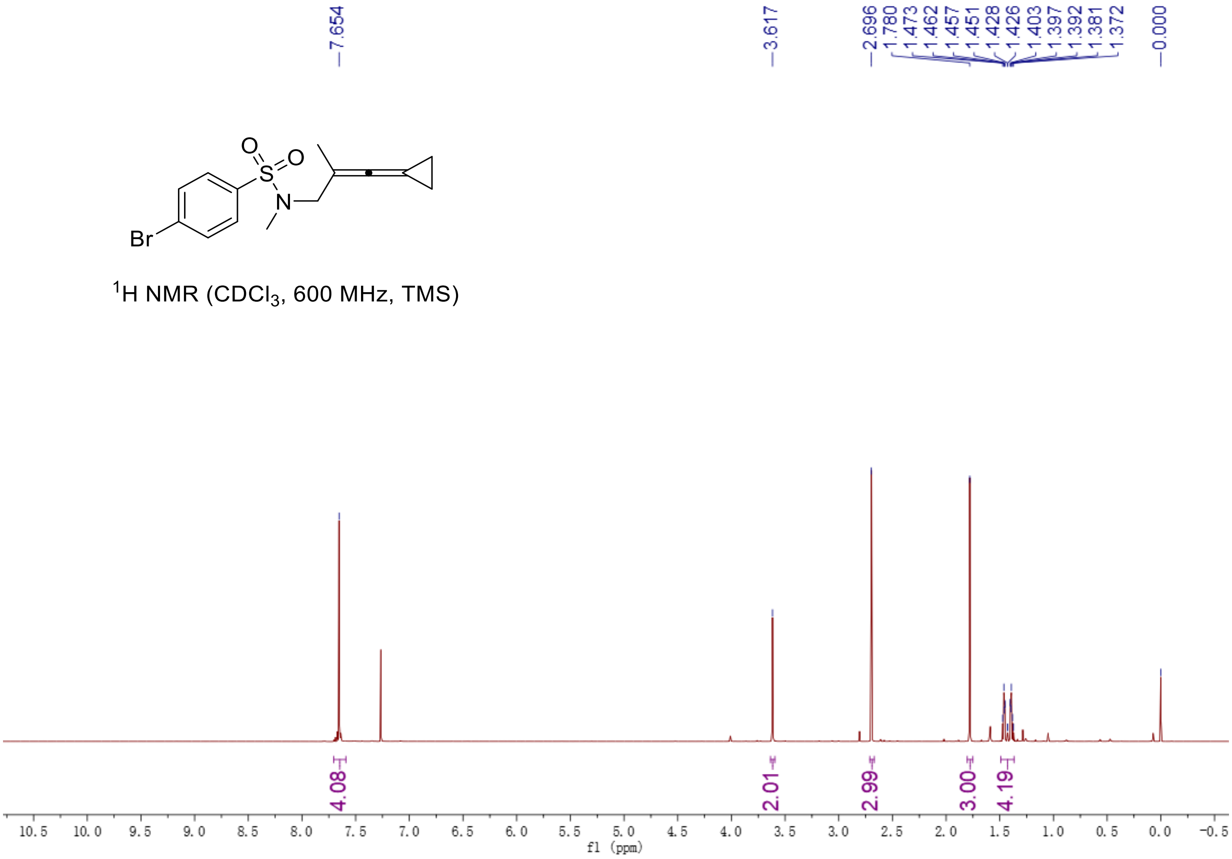
7.654
3.617
2.696
1.780
1.473
1.462
1.457
1.451
1.428
1.426
1.403
1.397
1.392
1.381
1.372
0.000
O
S
O
N
Br
^{1}H NMR ($CDCl_3$, 600 MHz, TMS)
4.08
2.01
2.99
3.00
4.19
f1 (ppm)

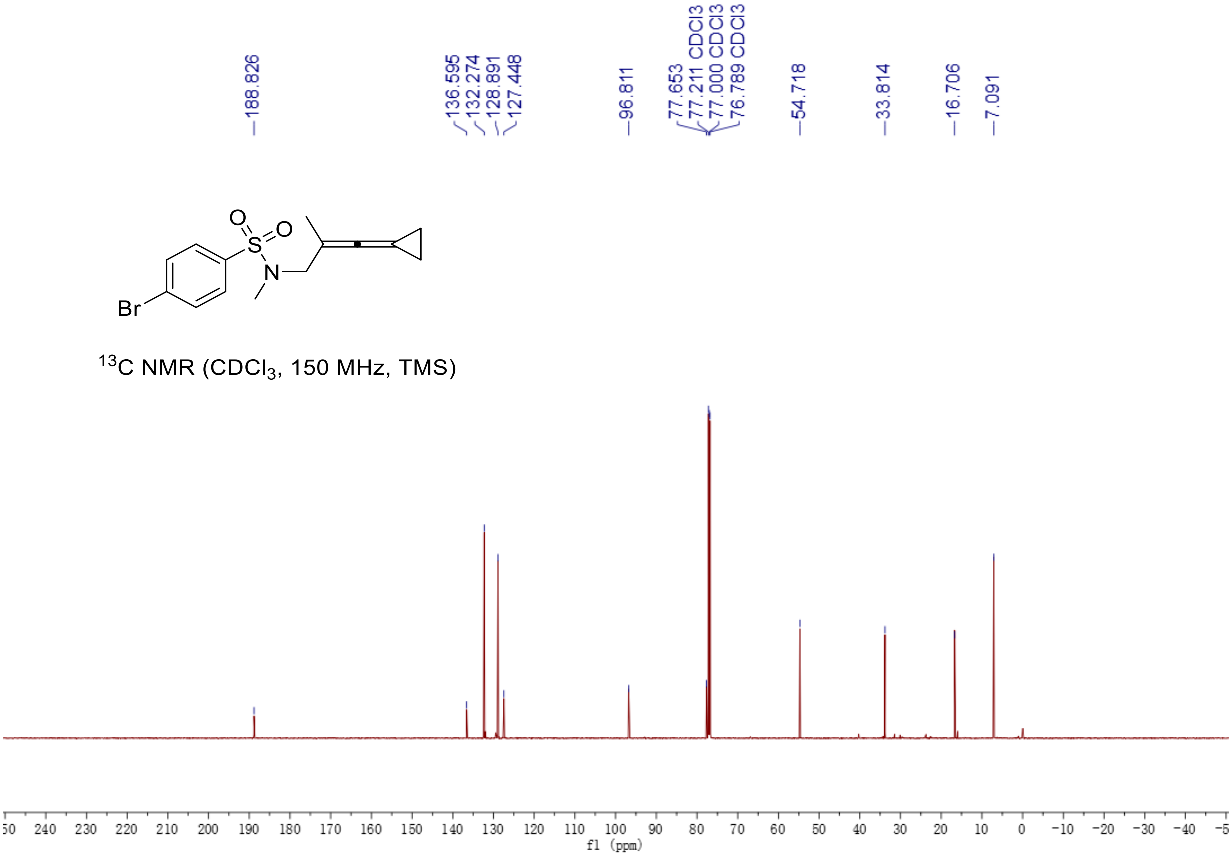
188.826
136.595
132.274
128.891
127.448
96.811
77.653
77.211 CDCl3
77.000 CDCl3
76.789 CDCl3
54.718
33.814
16.706
7.091
O
S
O
N
Br
^{13}C NMR ($CDCl_3$, 150 MHz, TMS)
f1 (ppm)

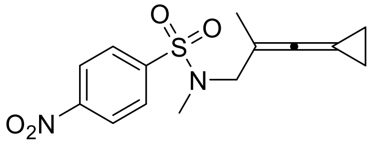

N-(3-cyclopropylidene-2-methyl-3λ^5-allyl)-N-methyl-4-nitrobenzenesulfonamide (1ac)

A yellow oil, 40% yield, 123.4 mg. ^{1}H NMR ($CDCl_3$, TMS, 400 MHz) δ 8.37 (d, *J* = 8.9 Hz, 2H), 7.98 (d, *J* = 8.8 Hz, 2H), 3.69 (s, 2H), 2.76 (s, 3H), 1.78 (s, 3H), 1.49 - 1.37 (m, 4H). ^{13}C NMR ($CDCl_3$, TMS, 100 MHz) δ 188.8, 150.0, 143.7, 128.5, 124.3, 96.4, 77.9, 54.7, 33.9, 16.7, 7.2. IR (neat) ν 2966, 1674, 1480, 1448, 1396, 1219, 991, 756, 738, 722, 697 cm^{-1}. HRMS (ESI) calcd. for $C_{14}H_{17}N_2O_4S$ (M+H): 309.0905, Found: 309.0904.

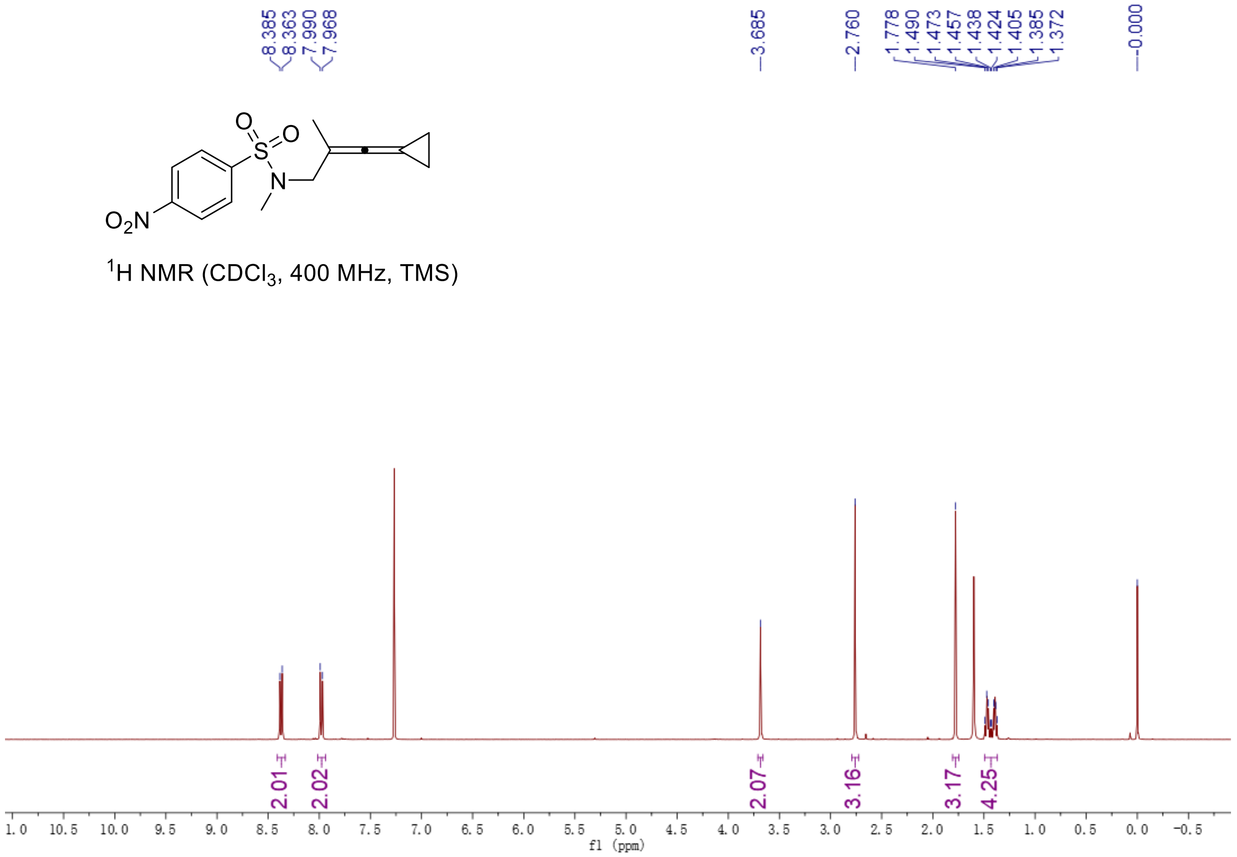

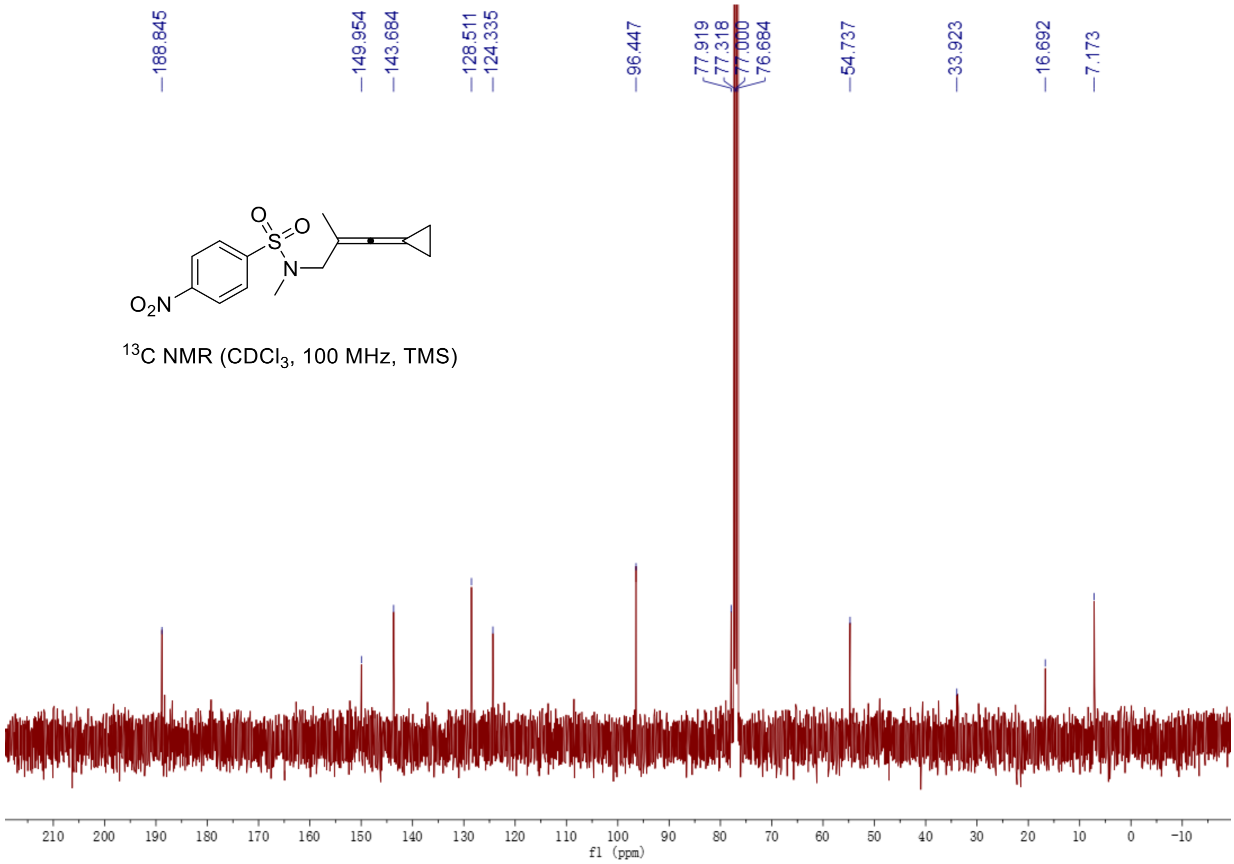

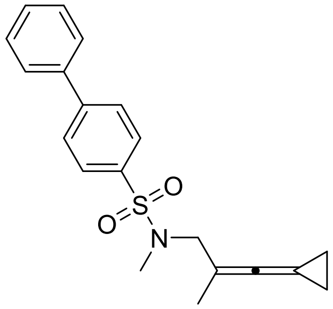

_N_-(3-cyclopropylidene-2-methyl-3λ[5]-allyl)-_N_-methyl-[1,1'-biphenyl]-4-sulfonamide (1ad)

A colorless oil, 44% yield, 149.4 mg. ^{1}H NMR ($CDCl_3$, TMS, 400 MHz) δ 7.85 (d, J = 8.5 Hz, 2H), 7.72 (d, J = 8.4 Hz, 2H), 7.62 - 7.58 (m, 2H), 7.51 - 7.39 (m, 3H), 3.67 (s, 2H), 2.74 (s, 3H), 1.81 (s, 3H), 1.48 - 1.36 (m, 4H). ^{13}C NMR ($CDCl_3$, TMS, 100 MHz) δ 188.8, 145.4, 139.3, 136.0, 129.0, 128.4, 127.9, 127.6, 127.3, 97.1, 77.5, 54.8, 33.9, 16.7, 7.1. IR (neat) ν 2932, 1712, 1588, 1461, 1418, 1396, 1034, 991, 891, 747, 697 cm^{-1}. HRMS (ESI) calcd. for $C_{20}H_{22}NO_2S$ (M+H): 340.1365, Found: 340.1366.

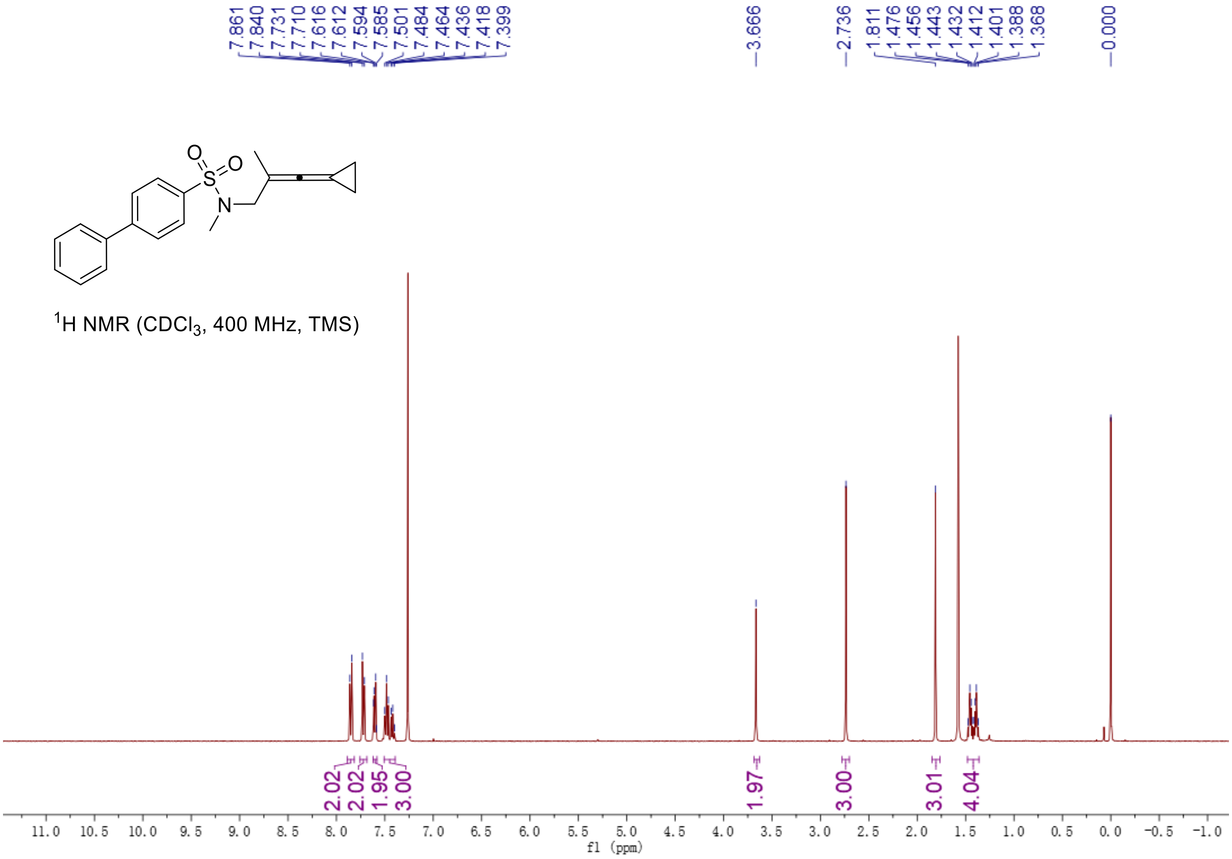

7.861
7.840
7.731
7.710
7.616
7.612
7.594
7.585
7.501
7.484
7.464
7.436
7.418
7.399
3.666
2.736
1.811
1.476
1.456
1.443
1.432
1.412
1.401
1.388
1.368
0.000
^{1}H NMR (CDCl$_3$, 400 MHz, TMS)
2.02
2.02
1.95
3.00
1.97
3.00
3.01
4.04
f1 (ppm)

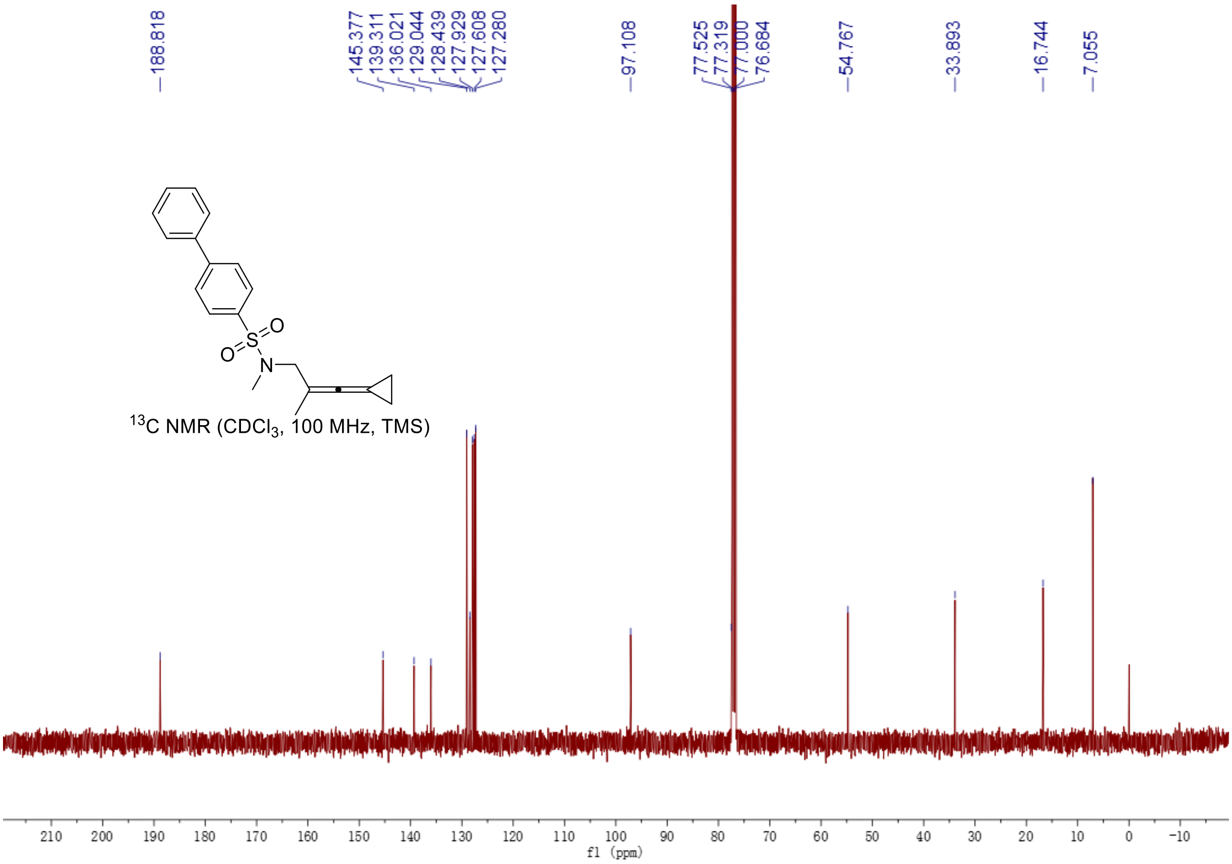

188.818
145.377
139.311
136.021
129.044
128.439
127.929
127.608
127.280
97.108
77.525
77.319
77.000
76.684
54.767
33.893
16.744
7.055
^{13}C NMR (CDCl$_3$, 100 MHz, TMS)
f1 (ppm)

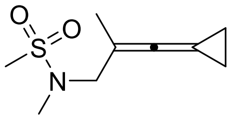

N-(3-cyclopropylidene-2-methyl-3l5-allyl)-N-methylmethanesulfonamide (1ae)

A colorless oil, 52% yield, 104.7 mg. ^{1}H NMR ($CDCl_3$, TMS, 400 MHz) δ 3.77 (s, 2H), 2.85 (s, 3H), 2.81 (s, 3H), 1.78 (s, 3H), 1.54 - 1.44 (m, 4H). ^{13}C NMR ($CDCl_3$, TMS, 150 MHz) δ 188.3, 97.5, 78.2, 77.2, 77.0, 76.8, 54.1, 36.2, 34.0, 16.9, 7.0. IR (neat) ν 2917, 1634, 1493, 1456, 1397, 1301, 1221, 1034, 991, 746, 697 cm^{-1}. HRMS (ESI) calcd. for $C_9H_{15}NO_2NaS$ (M+Na): 224.0721, Found: 224.0732.

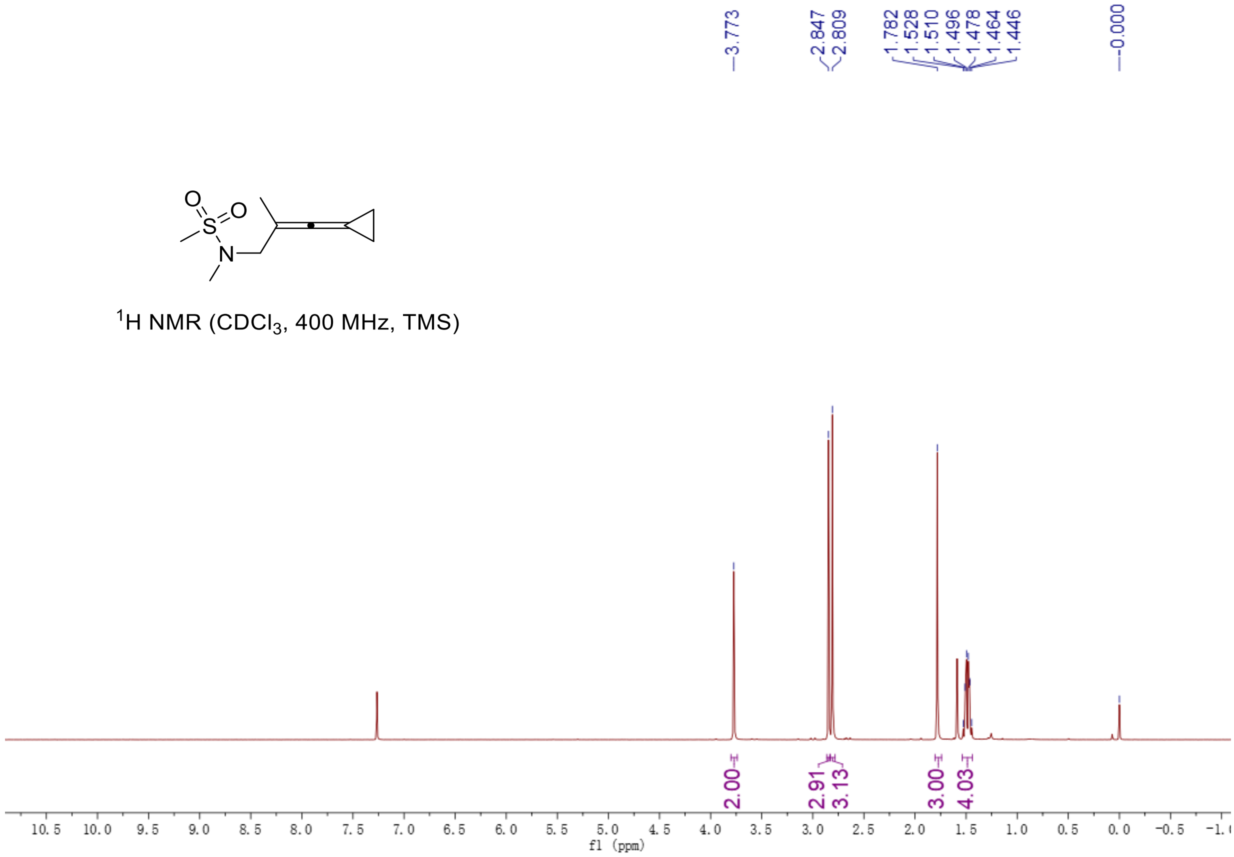

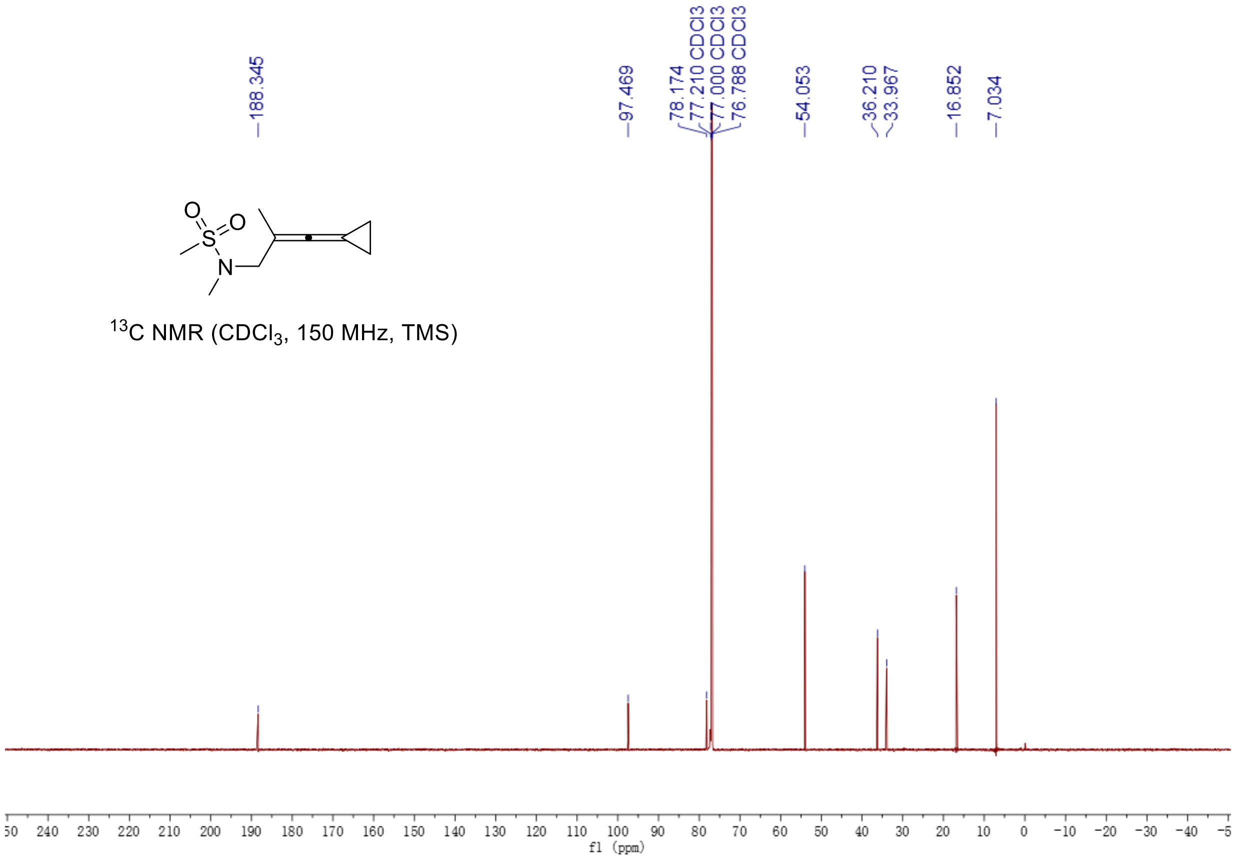

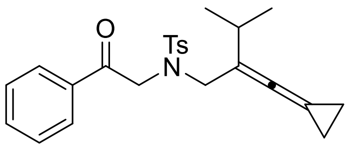

_N_-(2-(cyclopropylidene-l5-methylene)-3-methylbutyl)-4-methyl-_N_-(2-oxo-2-phenylethyl)benzenesulfonamide (1z)

A light yellow oil, this is a known compound and its spectroscopic data are consistent with those reported in the previous literature,[10] 33% yield, 135.1 mg. ^{1}H NMR ($CDCl_3$, TMS, 400 MHz) δ 0.95 (d, J = 6.8 Hz, 6H), 1.13-1.16 (m, 2H), 1.28-1.31 (m, 2H), 2.19-2.26 (m,1H), 2.43 (s, 3H),4.00 (s, 2H), 4.73 (s, 2H), 7.29 (d, J = 8.4 Hz, 2H), 7.46 (dd, J_1 = 7.6 Hz, J_2 = 8.0 Hz, 2H), 7.58 (dd, J_1 = 7.2 Hz, J_2 = 7.6 Hz, 1H), 7.75 (d, J = 8.0 Hz, 2H), 7.89 (d, J = 7.2 Hz, 2H). ^{13}C NMR ($CDCl_3$, TMS, 100 MHz) δ 7.1, 21.49, 21.54, 28.4, 49.8, 51.8, 79.4, 108.0, 127.5, 127.9, 128.7, 129.4, 133.5, 135.3, 137.0, 143.2, 188.0, 193.9.

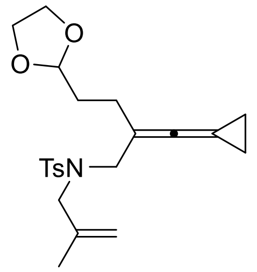

***N*-(2-(cyclopropylidene-λ^5-methylene)-4-(1,3-dioxolan-2-yl)butyl)-4-methyl-*N*-(2-methylallyl)benzenesulfonamide (1af)**

A yellow oil, this is a known compound and its spectroscopic data are consistent with those reported in the previous literature,[1] 35% yield, 141.2 mg. ^{1}H NMR ($CDCl_3$, TMS, 400 MHz) δ 1.32- 1.36 (m, 2H), 1.38-1.42 (m, 2H), 1.63 (s, 3H), 1.66-1.71 (m, 2H), 2.00 (t, *J* = 8.0 Hz, 3H), 2.41 (s, 3H), 3.75 (s, 2H), 3.82-3.84 (m, 4H), 3.92-3.96 (m, 2H), 4.81(t, *J* = 4.8 Hz, 1H), 4.84-4.86 (m, 2H), 7.27 (d, *J* = 8.0 Hz, 2H), 7.70 (d, *J* = 8.0 Hz, 2H). ^{13}C NMR ($CDCl_3$, TMS, 100 MHz) δ 6.8, 19.9, 21.4, 23.9, 24.6, 31.8, 33.7, 50.1, 52.9, 64.7, 79.8, 101.8, 103.9, 104.4, 114.3, 127.1, 129.3, 137.5, 140.0, 142.8, 187.9

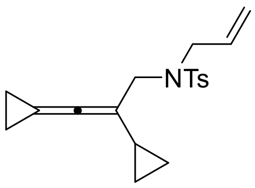

***N*-allyl-*N*-(2-cyclopropyl-3-cyclopropylidene-3-allyl)-4-methylbenzenesulfonamide (1aj)**

A yellow oil, this is a known compound and its spectroscopic data are consistent with those reported in the previous literature,[11] 39% yield, 128.3 mg. ^{1}H NMR ($CDCl_3$, TMS, 400 MHz) δ 7.71 (d, *J* = 8.3 Hz, 2H), 7.28 (d, *J* = 8.0Hz, 2H), 5.67 - 5.56 (m, 1H), 5.19 - 4.94 (m, 2H), 3.94 (s, 2H), 3.89 - 3.84 (m, 2H), 2.41 (s, 3H), 1.46 - 1.32 (m, 4H), 1.23 - 1.17 (m, 1H), 0.70 - 0.59 (m, 2H), 0.40 - 0.27 (m, 2H). ^{13}C NMR ($CDCl_3$, TMS, 100 MHz) δ 185.5, 141.9, 136.8, 131.4, 128.5, 126.2, 118.1, 104.6, 79.4, 48.9, 48.1, 20.5, 9.8, 6.12, 6.07.

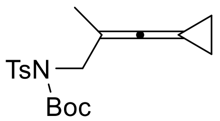

***tert*-butyl (3-cyclopropylidene-2-methyl-3λ^5-allyl)(tosyl)carbamate (1ak)**

A colorless oil, 60% yield, 217.8 mg. ^{1}H NMR ($CDCl_3$, TMS, 400 MHz) δ 7.82 (d, *J* = 8.3 Hz, 2H),

7.28 (d, J = 7.8 Hz, 2H), 4.44 (s, 2H), 2.43 (s, 3H), 1.78 (s, 3H), 1.43 - 1.39 (m, 2H), 1.35 (s, 9H), 1.31 - 1.27 (m, 2H). 13C NMR (CDCl3, TMS, 100 MHz) δ 186.4, 150.9, 143.9, 137.4, 129.1, 128.3, 99.7, 83.8, 80.6, 49.5, 27.9, 21.6, 17.2, 6.8. IR (neat) ν 2919, 1810, 1618, 1329, 1307, 1289, 1103, 981, 867, 709, 698 cm^{-1}. HRMS (ESI) calcd. for $C_{19}H_{26}O_4NS$ (M+H): 364.1577, Found: 364.1563.

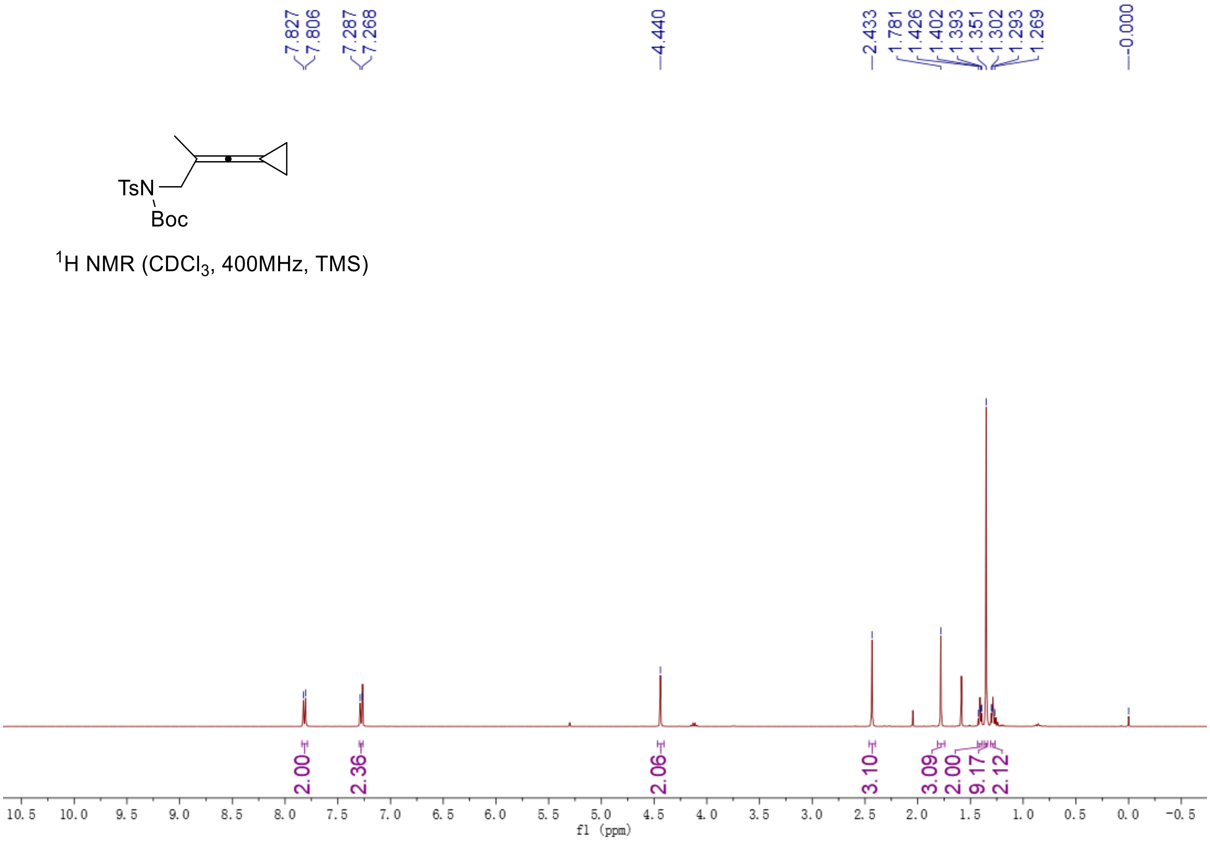

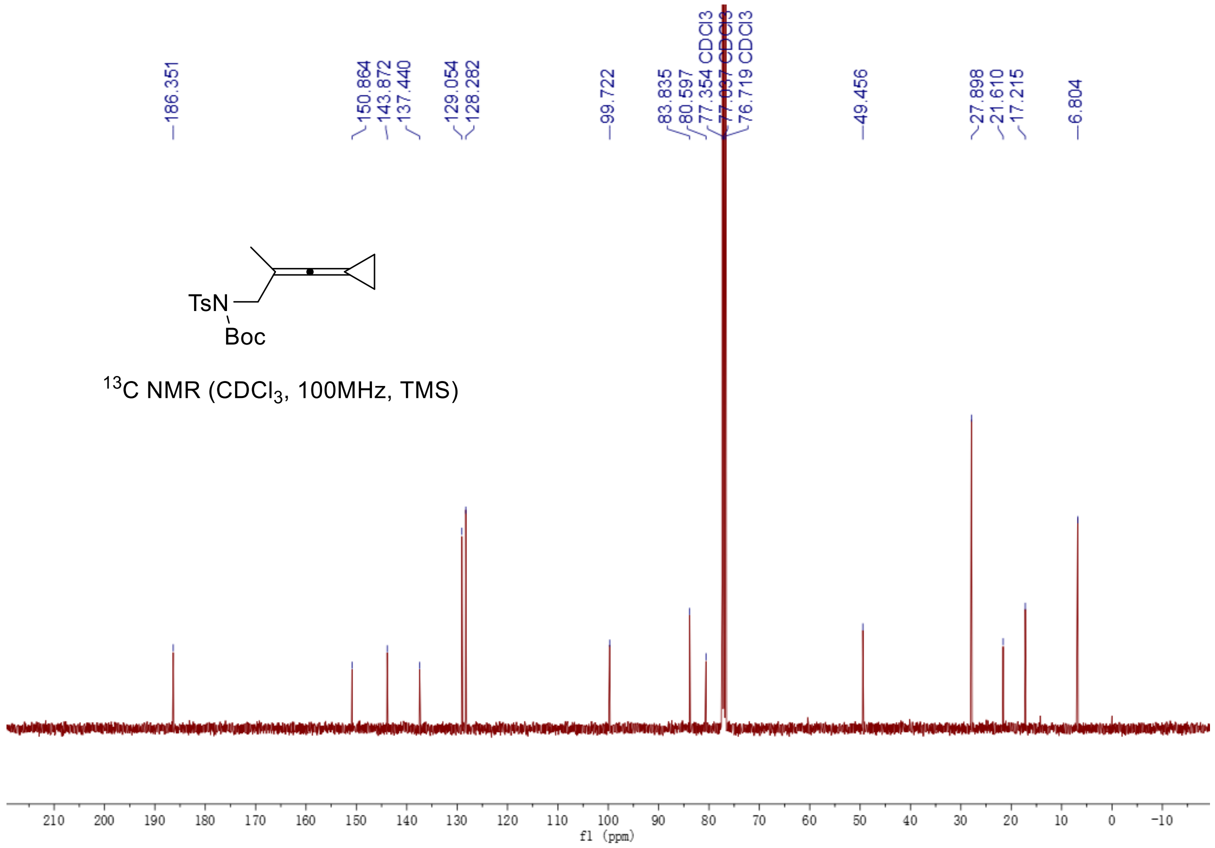

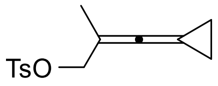

3-cyclopropylidene-2-methyl-3λ[5]-allyl 4-methylbenzenesulfonate (5a)

A colorless oil, 46% yield, 121.6 mg. ^{1}H NMR ($CDCl_3$, TMS, 600 MHz) δ 7.81 (d, *J* = 8.3 Hz, 2H), 7.32 (d, *J* = 8.1 Hz, 2H), 5.21 (s, 1H), 4.95 (s, 1H), 2.45 (s, 3H), 1.52 - 1.46 (m, 2H), 1.04 - 0.98 (m, 2H). ^{13}C NMR ($CDCl_3$, TMS, 150 MHz) δ 144.7, 139.0, 137.4, 134.4, 129.6, 128.2, 114.6, 113.4, 21.7, 20.2, 6.7, 4.2. IR (neat) ν 2925, 1710, 1508, 1437, 1397, 1358, 1335, 1219, 1025, 991, 888, 739, 697 cm^{-1}. HRMS (ESI) calcd. for $C_{14}H_{16}O_3NaS$ (M+Na): 287.0735, Found: 287.0718.

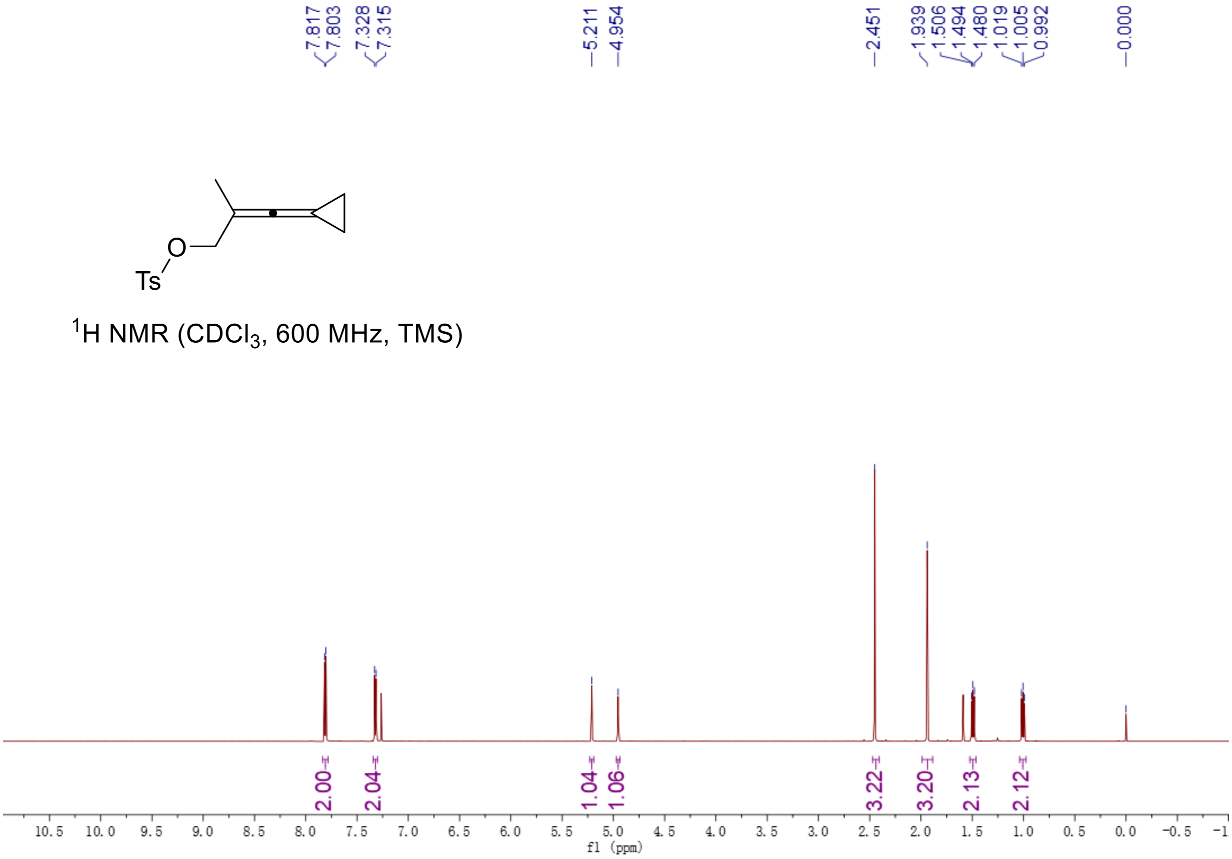

^{1}H NMR ($CDCl_3$, 600 MHz, TMS)

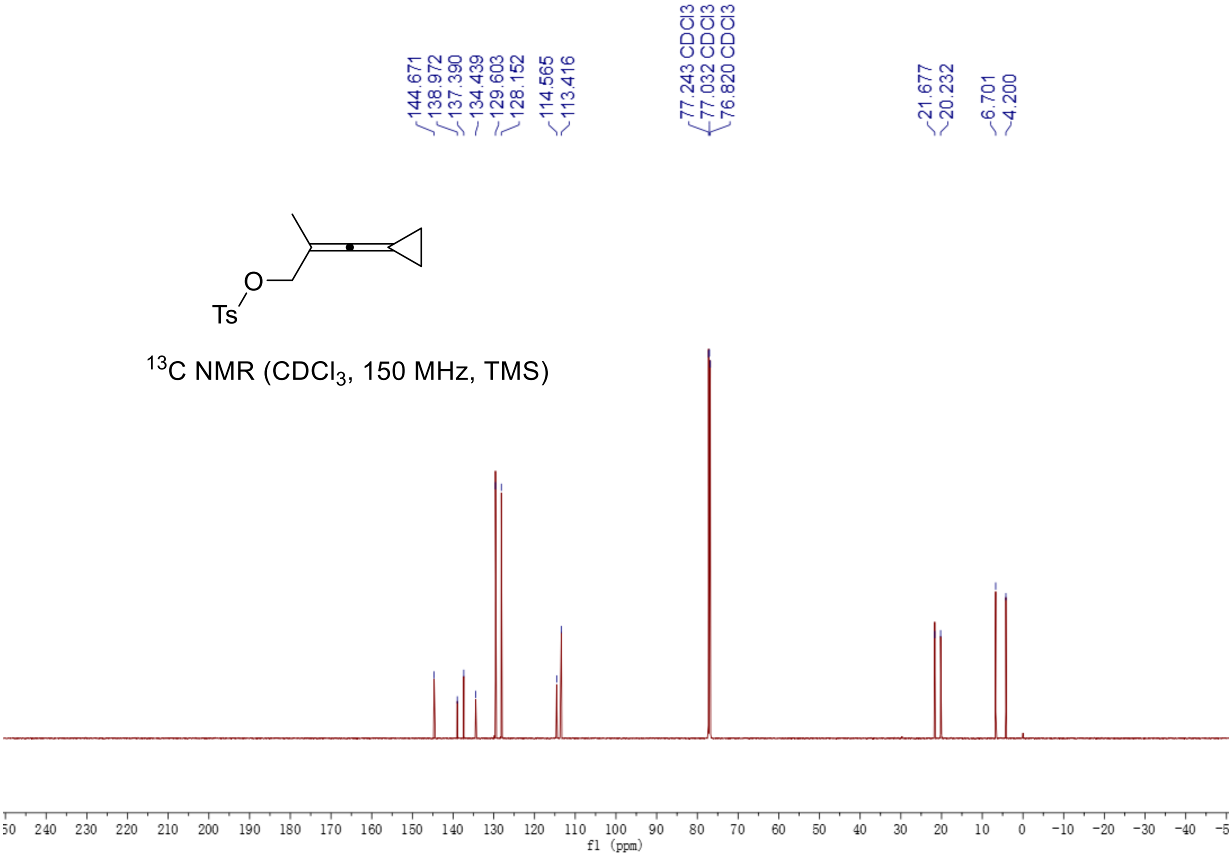

^{13}C NMR ($CDCl_3$, 150 MHz, TMS)

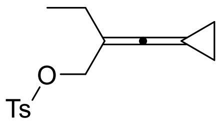

2-(cyclopropylidene-λ^5-methylene)butyl 4-methylbenzenesulfonate (5b)

A colorless oil, 43% yield, 119.7 mg. ^{1}H NMR ($CDCl_3$, TMS, 600 MHz) δ 7.82 (d, *J* = 8.4 Hz, 2H), 7.35 (d, *J* = 8.1 Hz, 2H), 4.85 (s, 1H), 4.84 (s, 2H), 2.71 - 2.69 (m, 2H), 2.46 (s, 3H), 2.18 - 2.14 (m, 2H), 2.10 (q, *J* = 7.5 Hz, 2H), 0.97 (t, *J* = 7.4 Hz, 3H). ^{13}C NMR ($CDCl_3$, TMS, 150 MHz) δ 145.3, 142.8, 133.5, 132.5, 129.9, 128.0, 111.8, 30.5, 26.2, 21.7, 21.6, 12.7. IR (neat) ν 2915, 1665, 1466, 1310, 1230, 1025, 991, 737, 698 cm^{-1}. HRMS (ESI) calcd. for $C_{15}H_{18}O_3NaS$ (M+Na): 301.0887, Found: 301.0874.

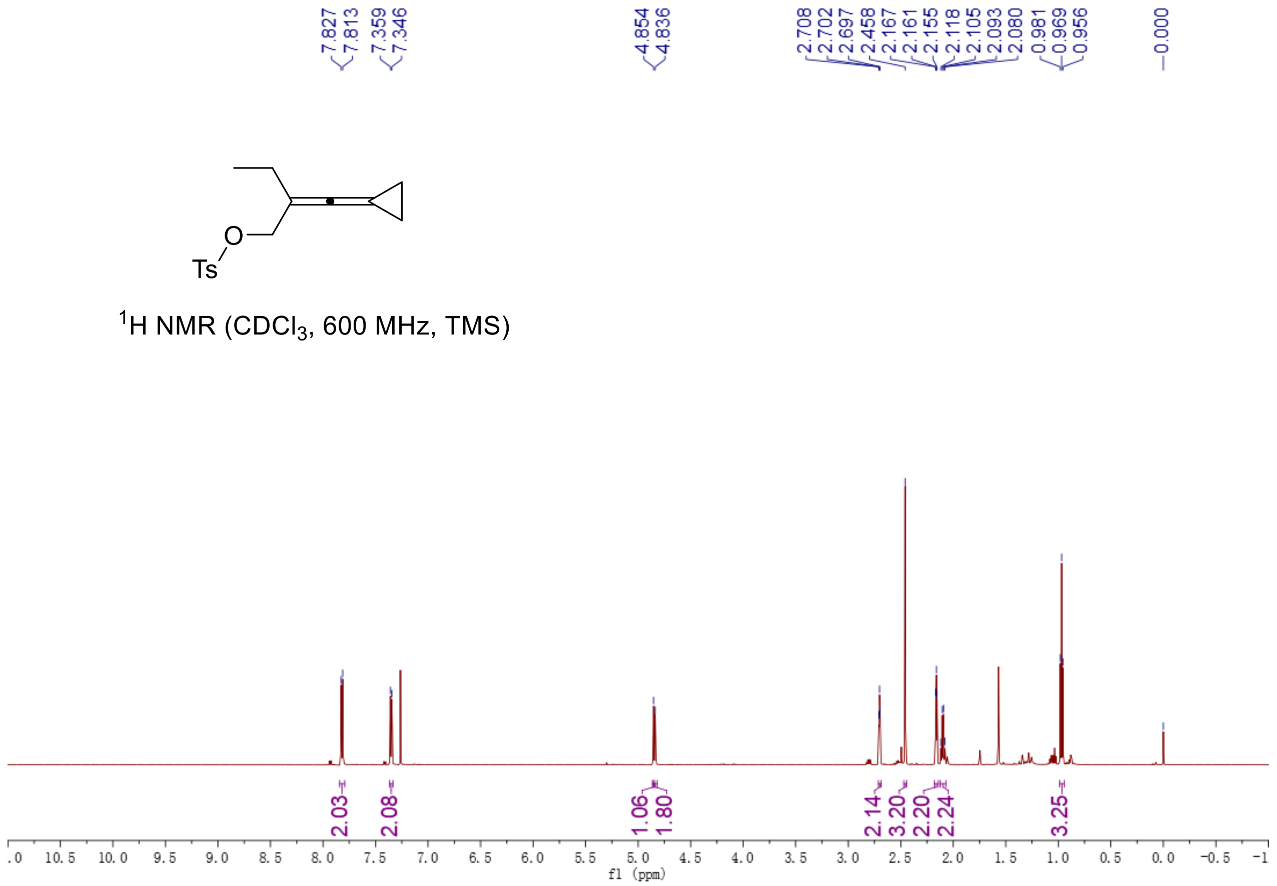

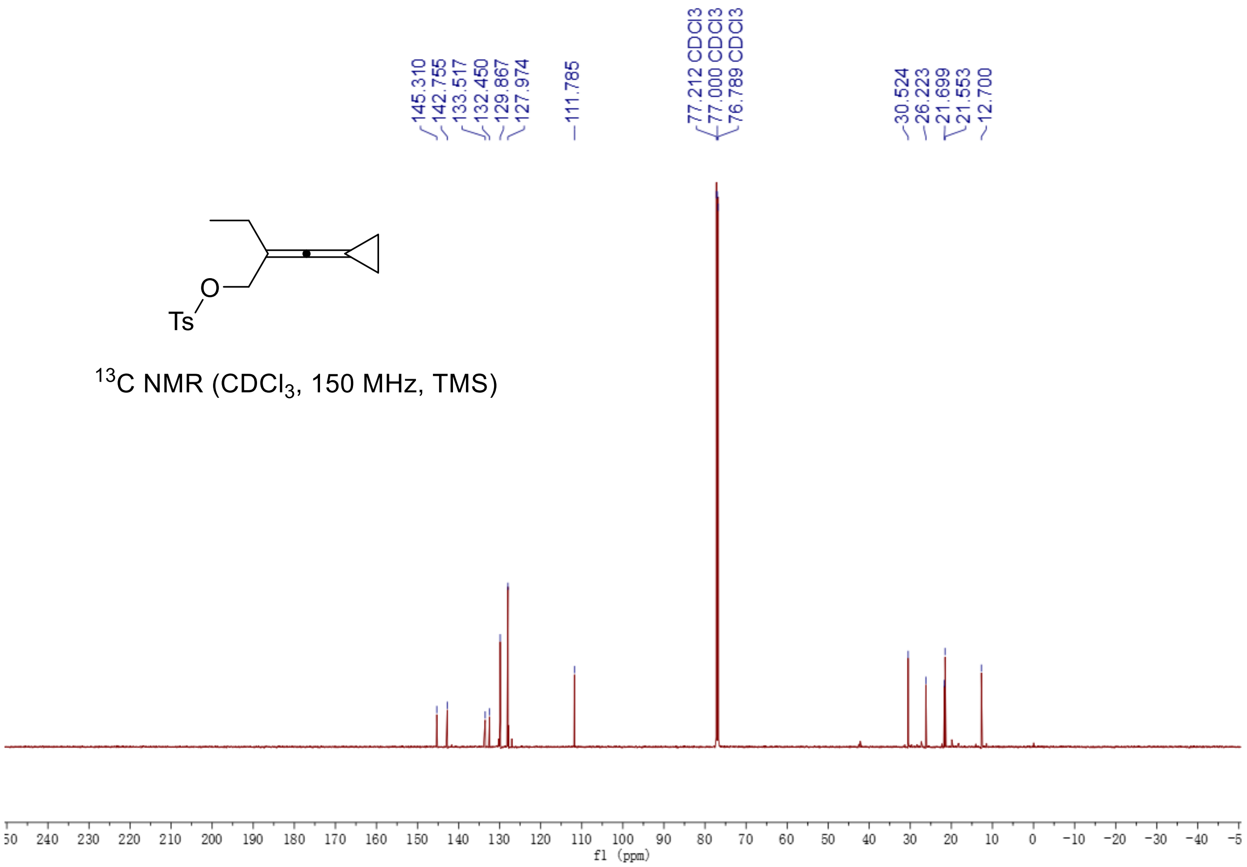

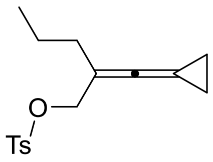

2-(cyclopropylidene-λ^5-methylene)pentyl 4-methylbenzenesulfonate (5c)

A colorless oil, 45% yield, 131.6 mg. ^{1}H NMR ($CDCl_3$, TMS, 600 MHz) δ 7.80 (d, *J* = 8.4 Hz, 2H), 7.31 (d, *J* = 8.0 Hz, 2H), 5.25 (s, 1H), 4.94 (s, 1H), 2.45 (s, 3H), 2.27 - 2.21 (m, 2H), 1.49 - 1.45 (m, 2H), 1.44 - 1.38 (m, 2H), 1.07 - 1.00 (m, 2H), 0.88 (t, *J* = 7.4 Hz, 3H). ^{13}C NMR ($CDCl_3$, TMS, 150 MHz) δ 144.6, 141.5, 138.7, 134.4, 129.5, 128.2, 113.9, 112.9, 35.2, 21.9, 21.6, 13.7, 6.6, 4.2. IR (neat) ν 3075, 2895, 2859, 1781, 1675, 1566, 1440, 1386, 1325, 1226, 1025, 981, 747, 688 cm^{-1}. HRMS (ESI) calcd. for $C_{16}H_{20}O_3NaS$ (M+Na): 315.1048, Found: 315.1031.

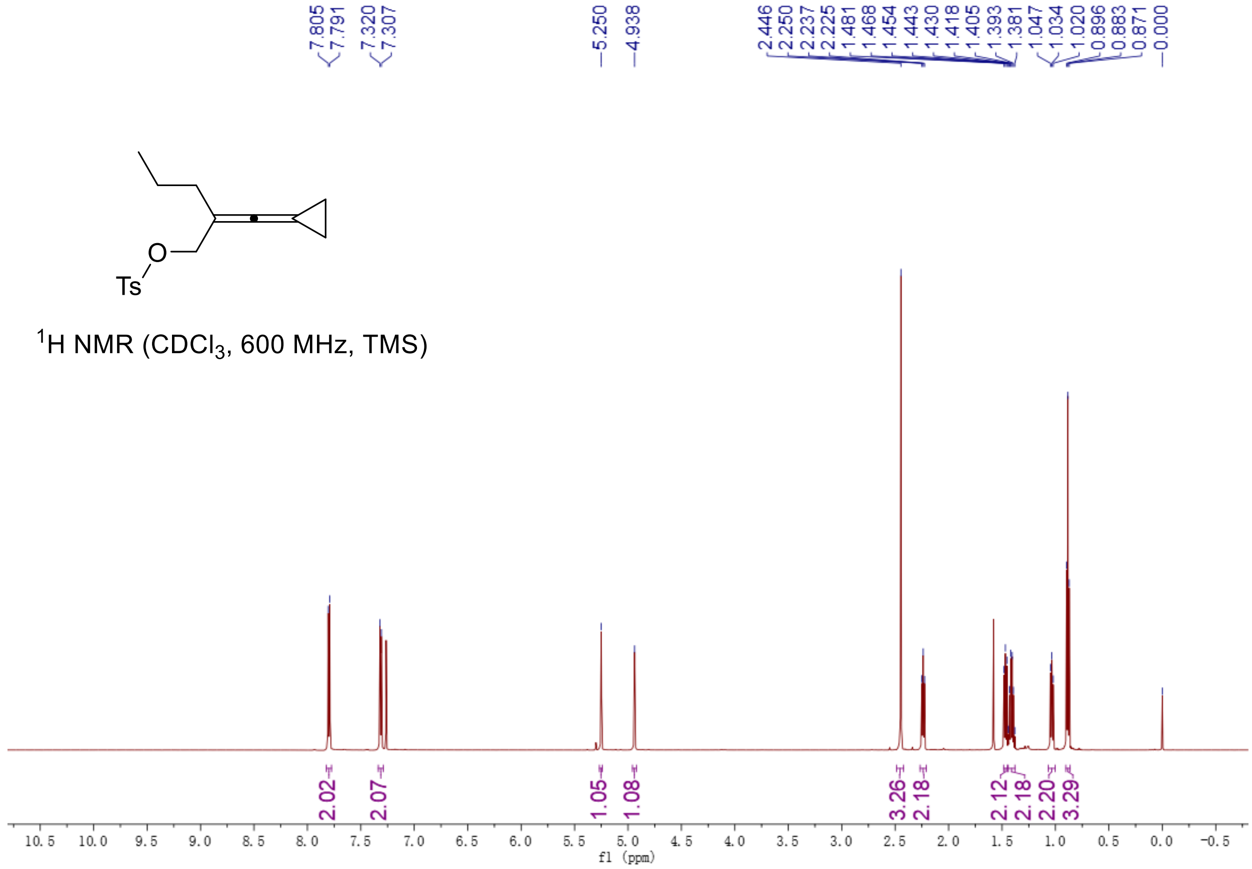
7.805
7.791
7.320
7.307
5.250
4.938
2.446
2.250
2.237
2.225
1.481
1.468
1.454
1.443
1.430
1.418
1.405
1.393
1.381
1.047
1.034
1.020
0.896
0.883
0.871
0.000
Ts
O
1H NMR (CDCl3, 600 MHz, TMS)
2.02
2.07
1.05
1.08
3.26
2.18
2.12
2.18
2.20
3.29
f1 (ppm)

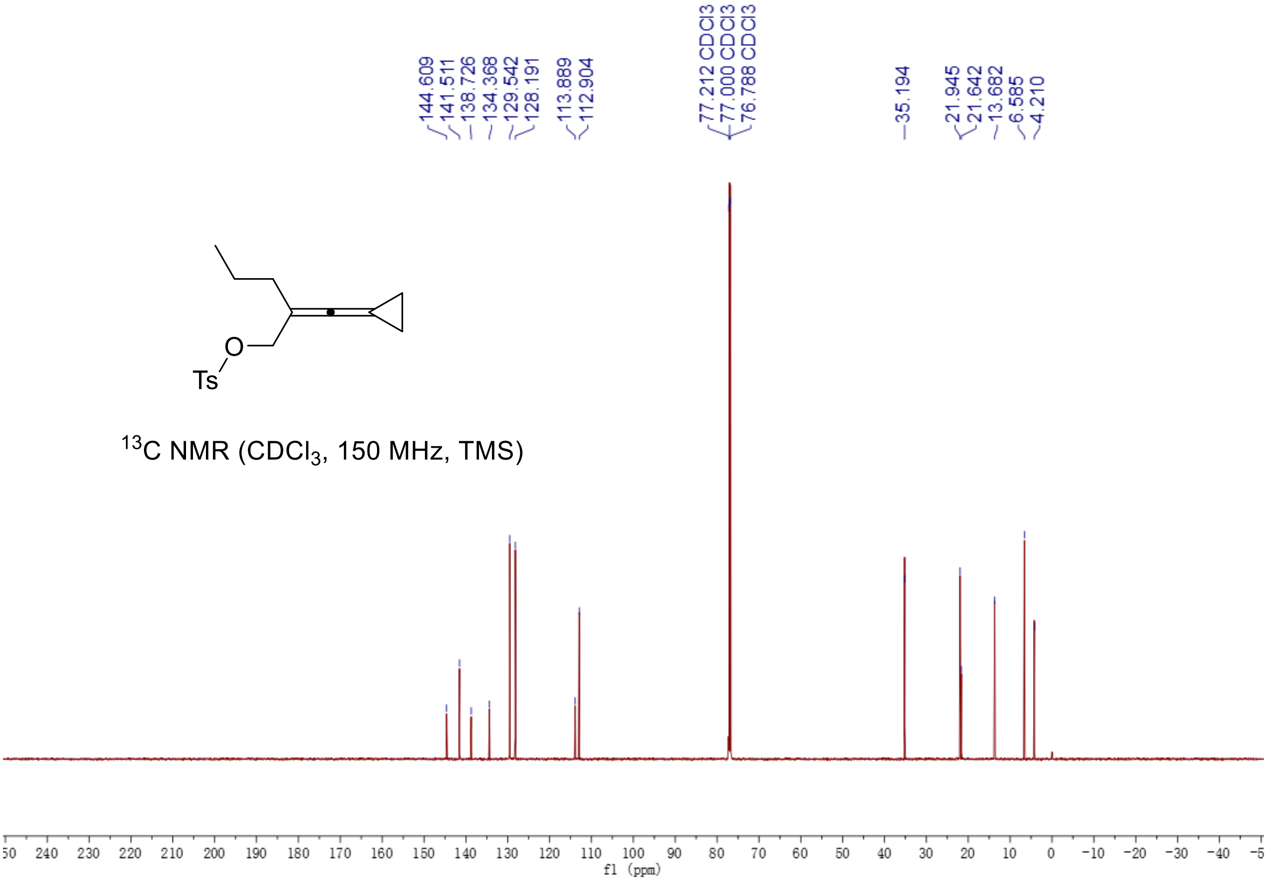
144.609
141.511
138.726
134.368
129.542
128.191
113.889
112.904
77.212 CDCl3
77.000 CDCl3
76.788 CDCl3
35.194
21.945
21.642
13.682
6.585
4.210
Ts
O
13C NMR (CDCl3, 150 MHz, TMS)
f1 (ppm)

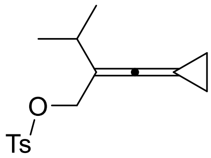

2-(cyclopropylidene-λ5-methylene)-3-methylbutyl 4-methylbenzenesulfonate (5d)

A colorless oil, 44% yield, 128.7 mg. ^{1}H NMR ($CDCl_3$, TMS, 400 MHz) δ 7.79 (d, *J* = 8.1 Hz, 2H), 7.31 (d, *J* = 8.0 Hz, 2H), 5.27 (s, 1H), 4.97 (s, 1H), 2.77 - 2.66 (m, 1H), 2.44 (s, 3H), 1.51 - 1.43 (m, 2H), 1.09 - 1.05 (m, 2H), 1.02 (d, *J* = 6.8 Hz, 6H). ^{13}C NMR ($CDCl_3$, TMS, 100 MHz) δ 148.1, 144.6, 138.8, 134.4, 129.5, 128.2, 113.4, 110.1, 29.1, 22.2, 21.6, 6.7, 4.2. IR (neat) ν 2959, 1663, 1596, 1437, 1314, 1219, 1035, 990, 783, 736, 696 cm^{-1}. HRMS (ESI) calcd. for $C_{16}H_{20}O_3NaS$ (M+Na): 315.1058, Found: 315.1031.

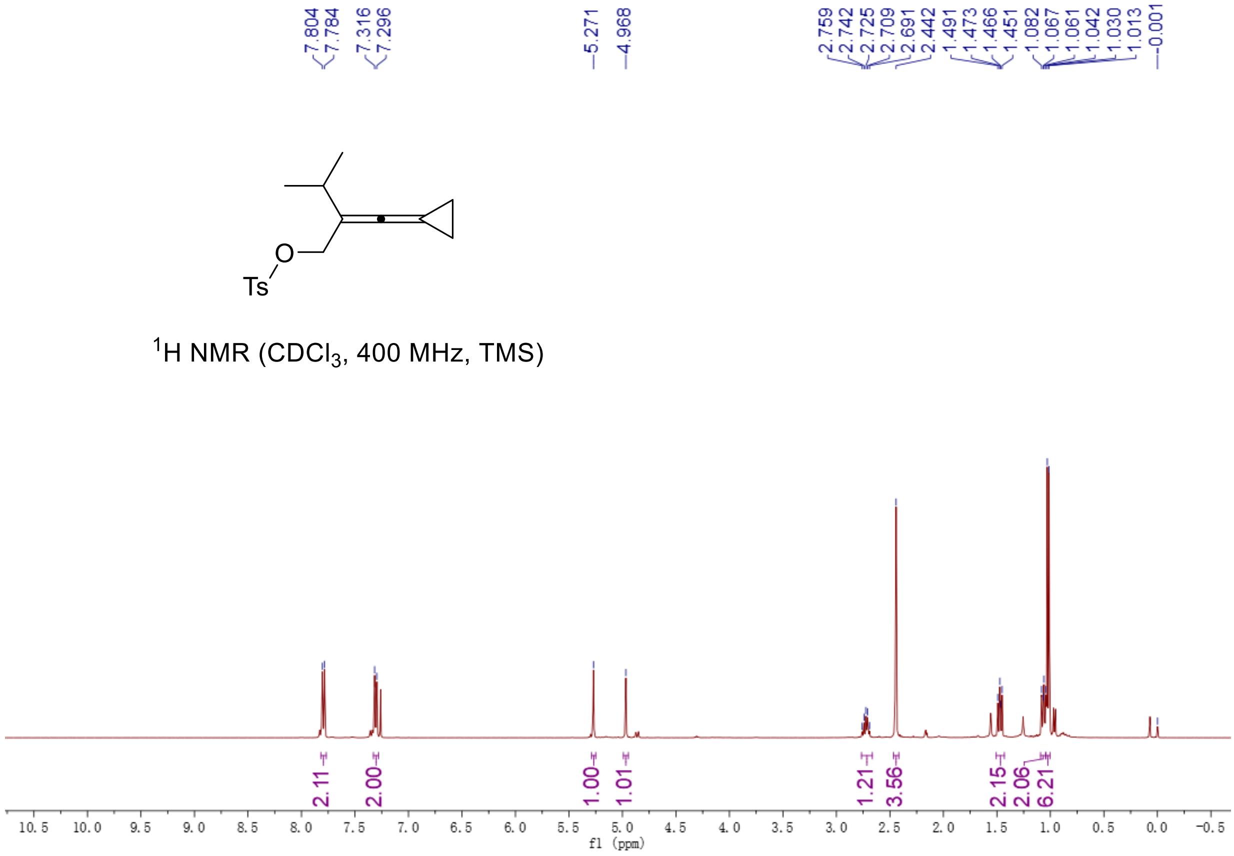

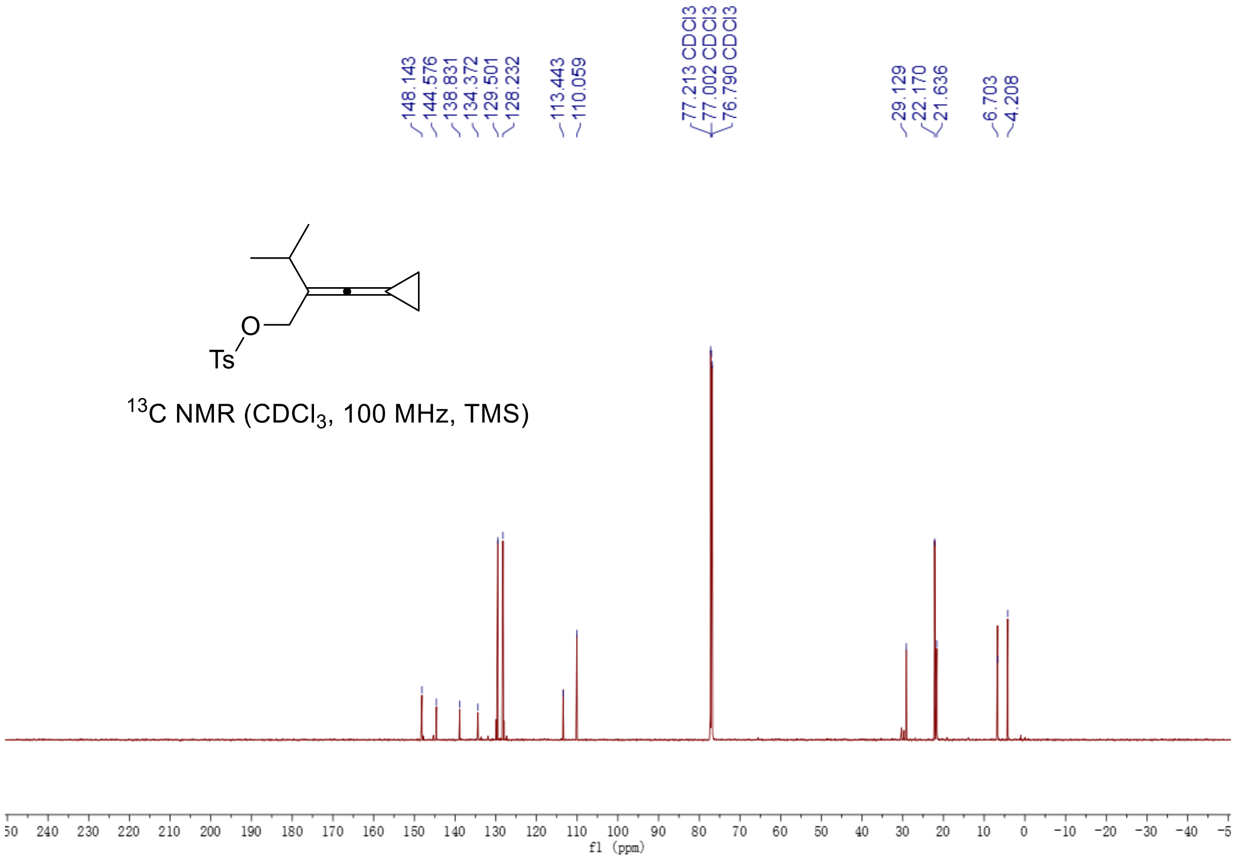

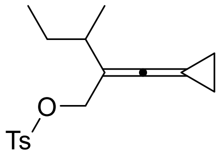

2-(cyclopropylidene-λ^5-methylene)-3-methylpentyl 4-methylbenzenesulfonate (5e)

A colorless oil, 43% yield, 131.7 mg. ^{1}H NMR ($CDCl_3$, TMS, 600 MHz) δ 7.80 (d, *J* = 8.3 Hz, 2H), 7.31 (d, *J* = 8.0 Hz, 2H), 5.31 (s, 1H), 4.94 (s, 1H), 2.57 - 2.51 (m, 1H), 2.45 (s, 3H), 1.53 - 1.45 (m, 3H), 1.31 - 1.23 (m, 1H), 1.09 - 1.02 (m, 2H), 1.00 (d, *J* = 6.9 Hz, 3H), 0.83 (t, *J* = 7.4 Hz, 3H). ^{13}C NMR ($CDCl_3$, TMS, 150 MHz) δ 146.9, 144.6, 139.1, 134.3, 129.5, 128.2, 113.4, 110.8, 35.7, 29.0, 21.6, 19.5, 11.6, 6.8, 4.1. IR (neat) ν 2914, 1594, 1496, 1448, 1396, 1333, 1318, 1036, 992, 740, 709 cm^{-1}. HRMS (ESI) calcd. for $C_{17}H_{22}O_3NaS$ (M+Na): 329.1187, Found: 329.1195.

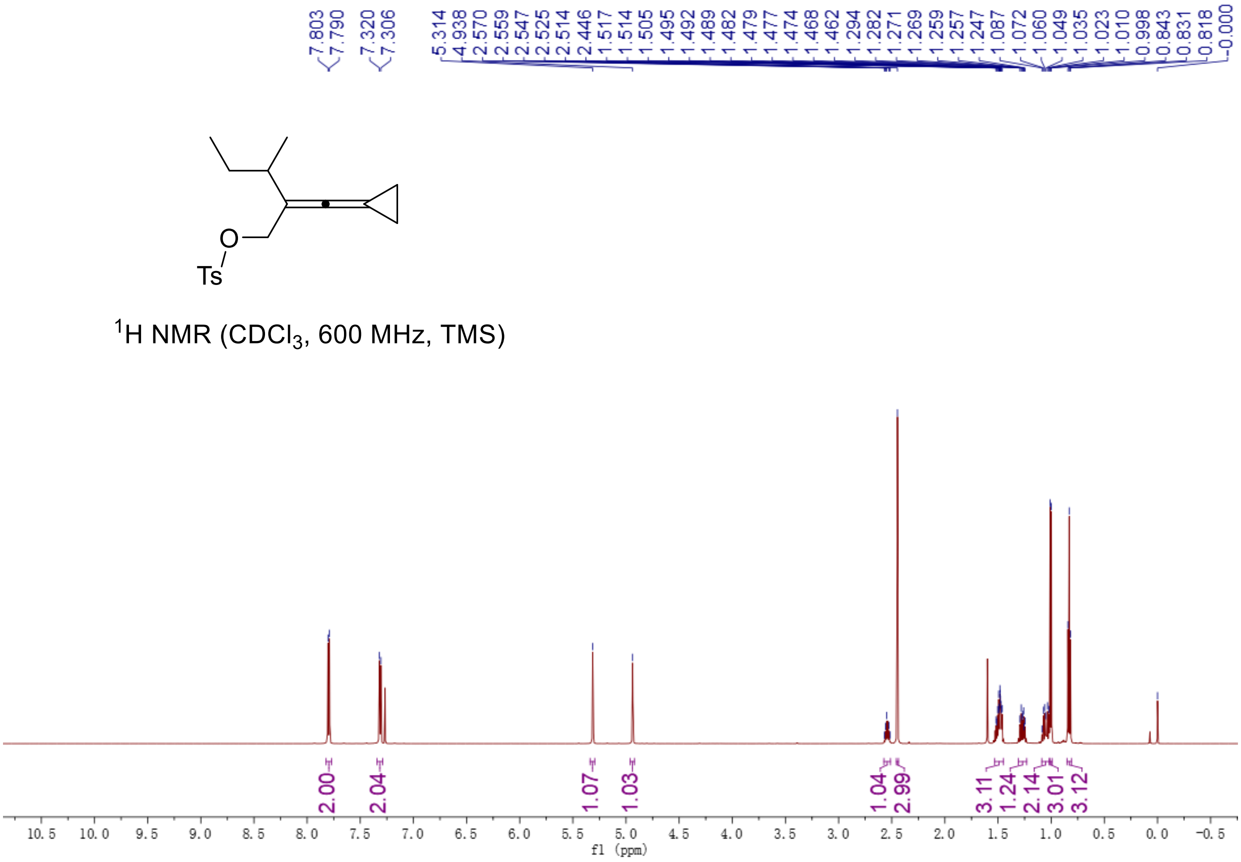
Ts
O
1H NMR (CDCl3, 600 MHz, TMS)
f1 (ppm)

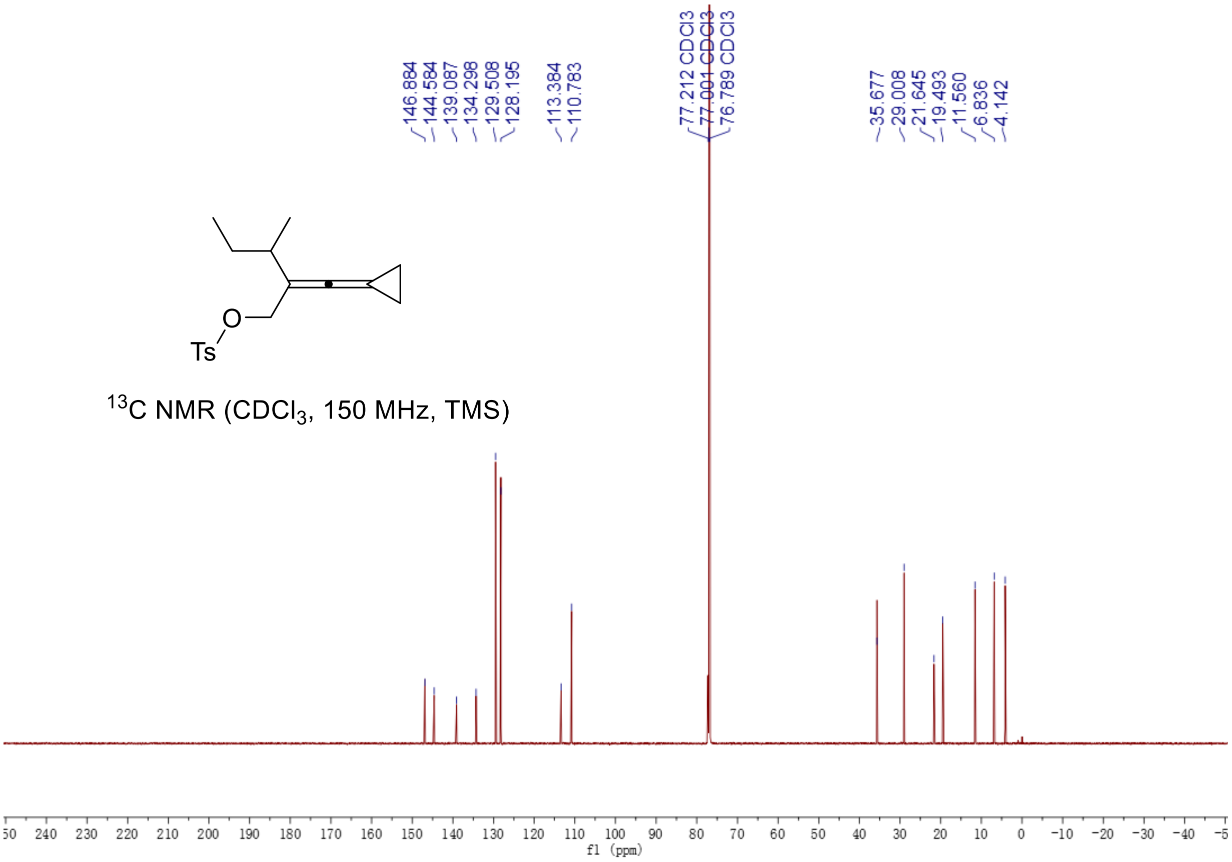
Ts
O
13C NMR (CDCl3, 150 MHz, TMS)
f1 (ppm)

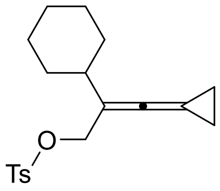

2-cyclohexyl-3-cyclopropylidene-3λ^5-allyl 4-methylbenzenesulfonate (5f)

A colorless oil, 45%, 149.6 mg. ^{1}H NMR ($CDCl_3$, TMS, 600 MHz) δ 7.79 (d, *J* = 8.3 Hz, 2H), 7.31 (d, *J* = 8.0 Hz, 2H), 5.25 (s, 1H), 4.92 (s, 1H), 2.45 (s, 3H), 2.36 - 2.30 (m, 1H), 1.78 - 1.72 (m, 4H), 1.71 - 1.67 (m, 1H), 1.49 - 1.45 (m, 2H), 1.31 - 1.22 (m, 3H), 1.19 - 1.11 (m, 2H), 1.07 - 1.03 (m, 2H). ^{13}C NMR ($CDCl_3$, TMS, 150 MHz) δ 147.2, 144.6, 138.9, 134.4, 129.5, 128.2, 113.2, 110.4, 39.6, 33.0, 26.8, 26.4, 21.6, 6.6, 4.1. IR (neat) ν 2845, 1663, 1510, 1439, 1402, 1325, 1220, 1032, 741, 703 cm^{-1}. HRMS (ESI) calcd. for $C_{19}H_{24}O_3NaS$ (M+Na): 355.1344, Found: 355.1365.

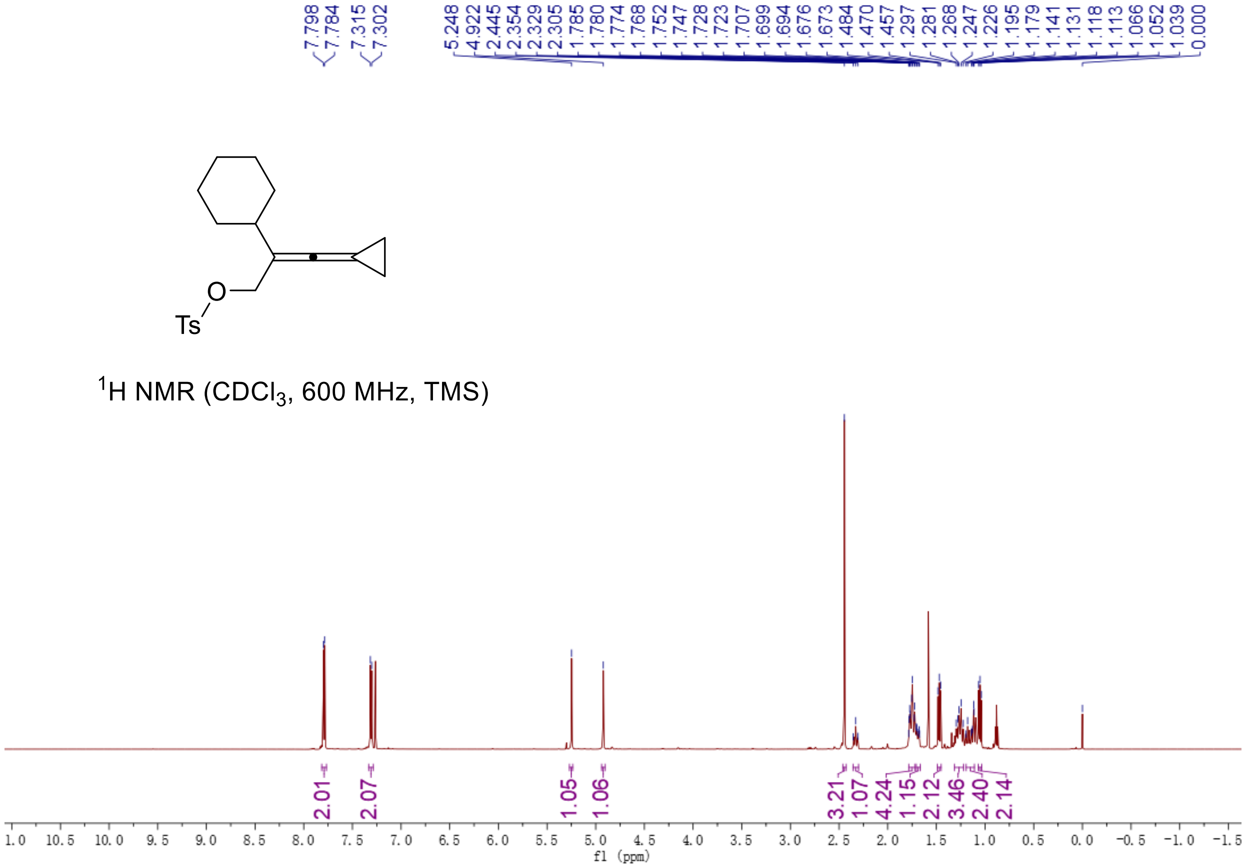

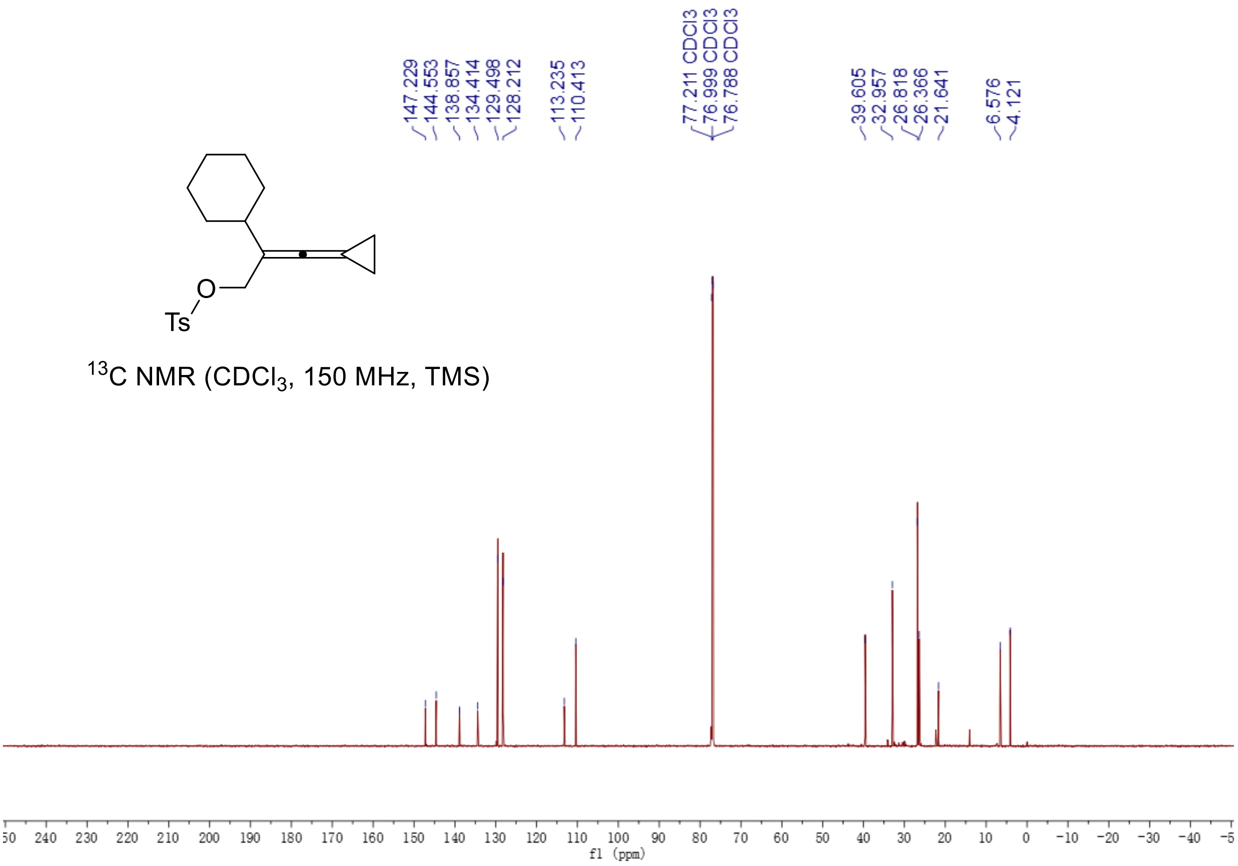

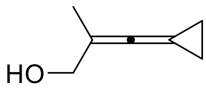

3-cyclopropylidene-2-methyl-3λ^5-prop-2-en-1-ol (5g)

A colorless oil, 40%, 44.0 mg. ^{1}H NMR ($CDCl_3$, TMS, 600 MHz) δ 4.09 (s, 2H), 1.80 (s, 3H), 1.52 - 1.49 (m, 4H), 1.47 - 1.43 (m, 1H). ^{13}C NMR ($CDCl_3$, TMS, 100 MHz) δ 185.1, 134.8, 103.4, 80.3, 64.5, 16.2, 7.0. IR (neat) ν 3016, 2985, 1663, 1510, 1439, 1300, 1087, 701, 695 cm^{-1}. HRMS (EI) calcd. for $C_7H_{10}O$ (M): 110.0726, Found: 110.0726.

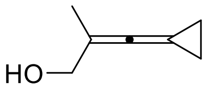

^{1}H NMR ($CDCl_3$, 600 MHz, TMS)

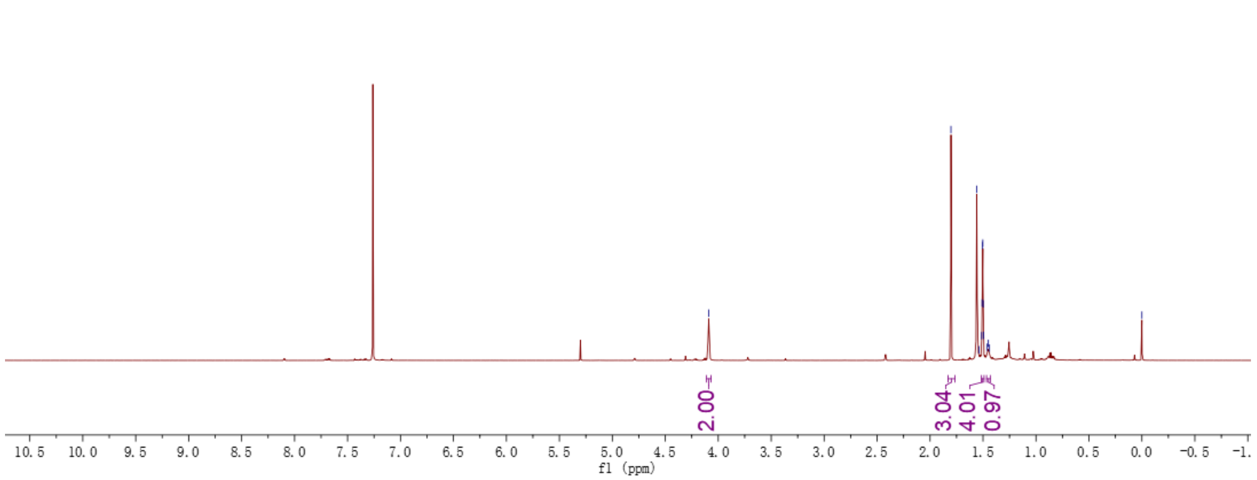

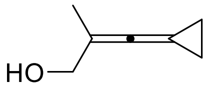

^{13}C NMR ($CDCl_3$, 100 MHz, TMS)

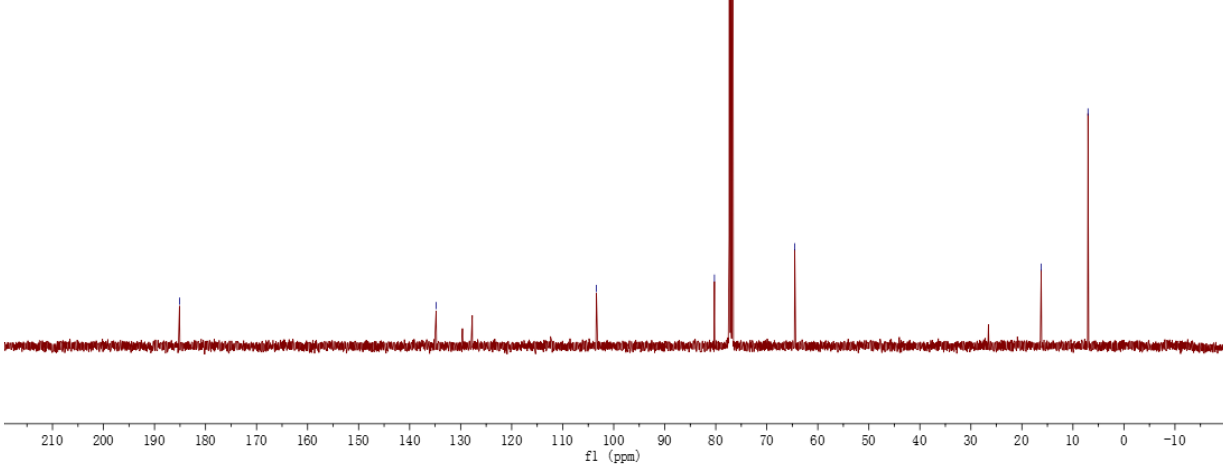

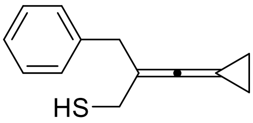

2-benzyl-3-cyclopropylidene-3λ[5]-prop-2-ene-1-thiol (7a)

A colorless oil, 33%, 66.7 mg. ^{1}H NMR ($CDCl_3$, TMS, 600 MHz) δ 7.23 (t, *J* = 7.5 Hz, 2H), 7.17 - 7.11 (m, 3H), 4.85 (s, 1H), 3.75 (s, 2H), 3.35 (s, 2H), 0.90 - 0.85 (m, 2H), 0.82 - 0.78 (m, 2H). ^{13}C NMR ($CDCl_3$, TMS, 150 MHz) δ 139.3, 138.8, 132.0, 128.8, 128.5, 126.4, 41.0, 37.9, 36.6, 14.7. IR (neat) ν 733, 906, 1077, 1255, 1388, 2924, 2990 cm^{-1}. HRMS (EI) calcd. for $C_{13}H_{14}S$ (M^+): 202.0809, Found: 202.0811.

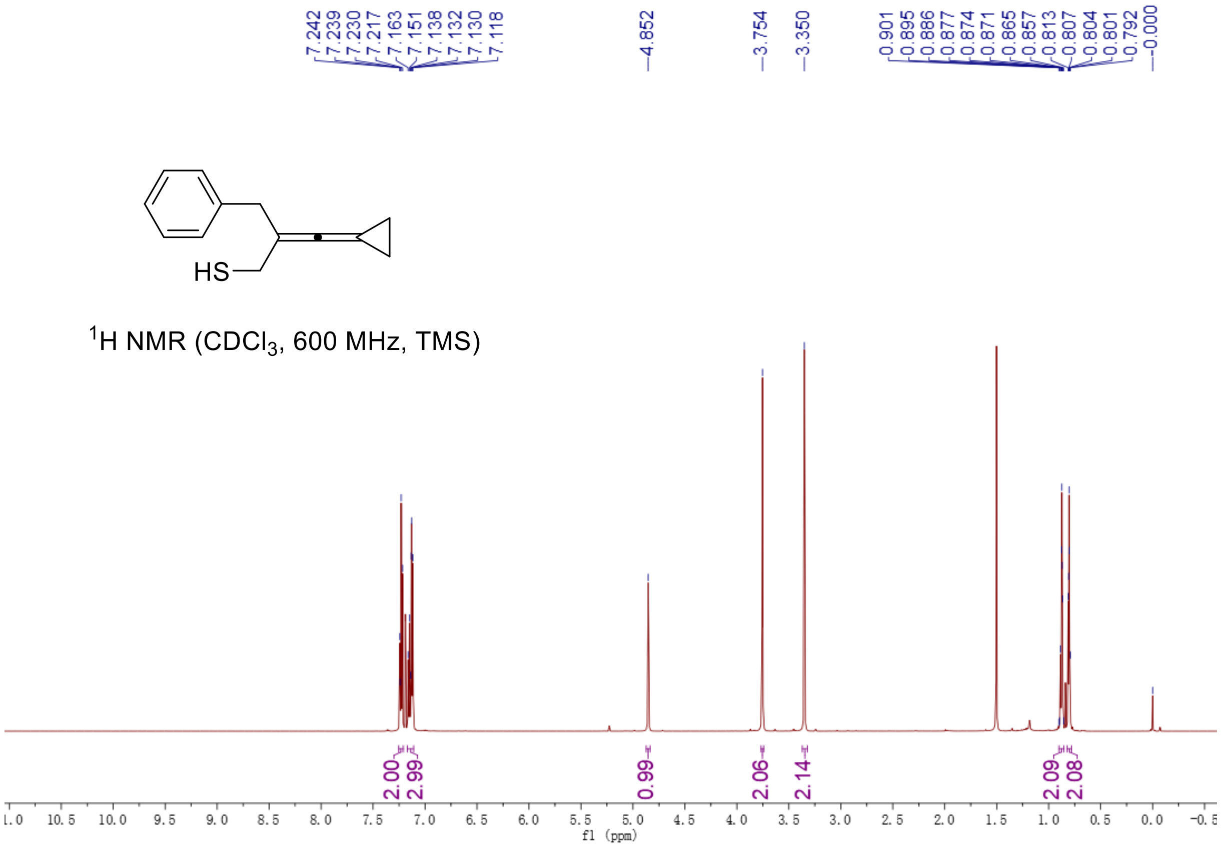

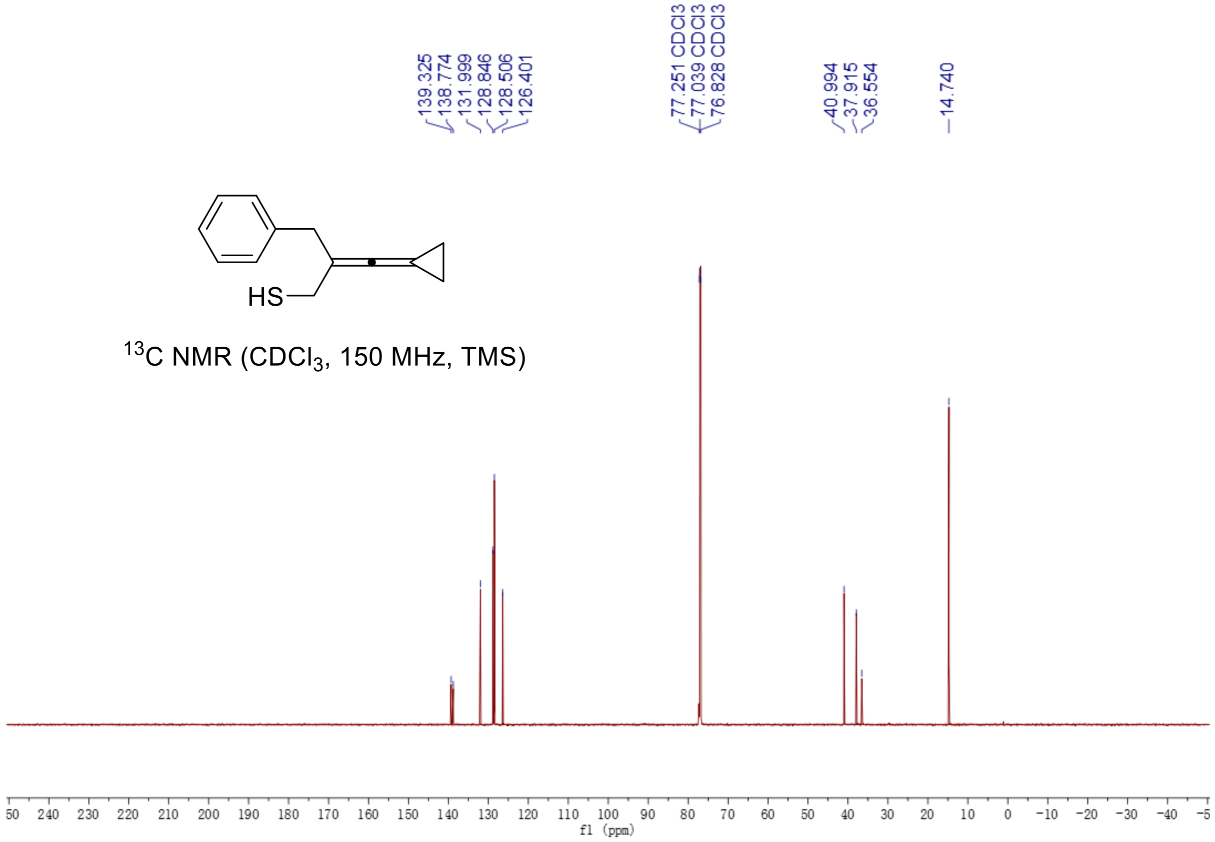

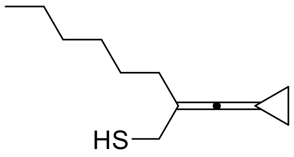

2-(cyclopropylidene-λ^5-methylene)octane-1-thiol (7b)

A colorless oil, 30%, 58.9 mg. ^{1}H NMR ($CDCl_3$, TMS, 600 MHz) δ 4.93 (s, 1H), 3.87 (s, 2H), 2.09 (t, J = 7.7 Hz, 2H), 1.50 - 1.39 (m, 2H), 1.35 - 1.21 (m, 8H), 0.96 - 0.93 (m, 2H), 0.89 - 0.86 (m, 5H). ^{13}C NMR ($CDCl_3$, TMS, 150 MHz) δ 140.4, 129.7, 41.3, 36.6, 34.1, 31.6, 31.3, 29.1, 27.9, 22.6, 22.3, 14.6, 14.1. IR (neat) ν 700, 928, 961, 1044, 1083, 1249, 1641, 2902 cm^{-1}. HRMS (EI) calcd. for $C_{12}H_{20}S$ (M^+): 196.1278, Found: 196.1280.

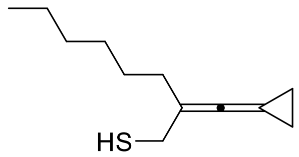

^{1}H NMR ($CDCl_3$, 600 MHz, TMS)

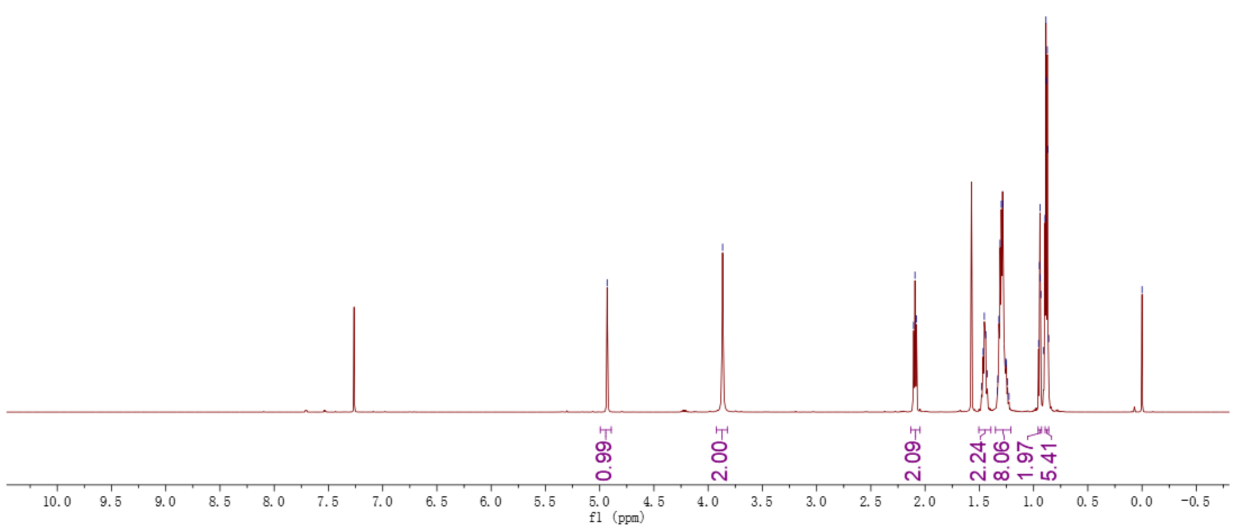

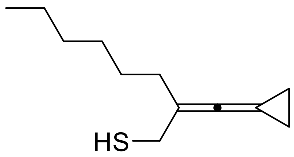

^{13}C NMR ($CDCl_3$, 150 MHz, TMS)

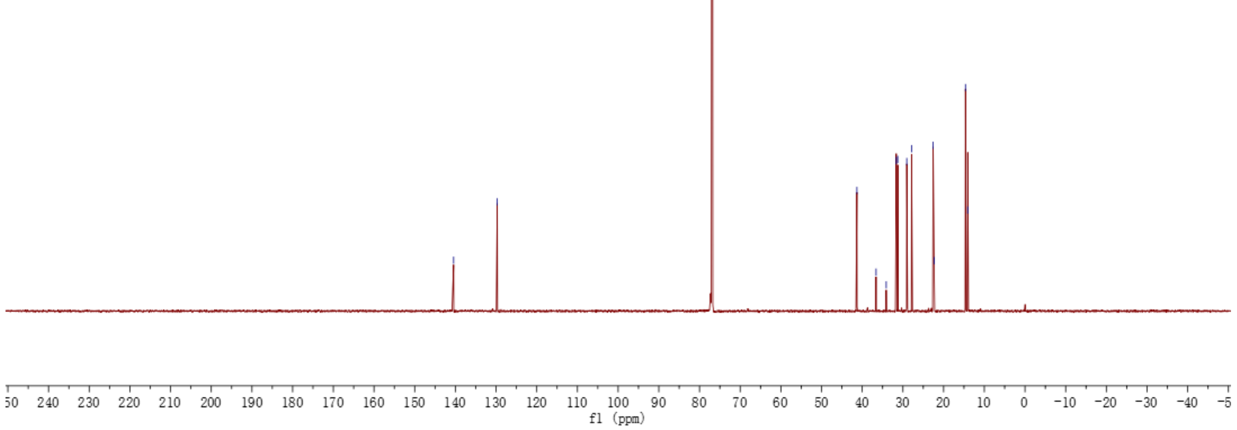

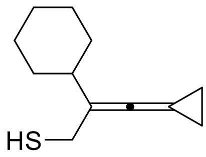

2-cyclohexyl-3-cyclopropylidene-3λ⁵-prop-2-ene-1-thiol (7c)

A colorless oil, 35%, 68.0 mg. ^{1}H NMR ($CDCl_3$, TMS, 600 MHz) δ 4.84 (s, 1H), 3.84 (s, 2H), 1.98 - 1.92 (m, 1H), 1.78 - 1.73 (m, 2H), 1.69 (dt, *J* = 12.9, 3.4 Hz, 2H), 1.63 - 1.58 (m, 1H), 1.22 - 1.16 (m, 2H), 1.13 - 1.05 (m, 3H), 0.90 - 0.85 (m, 2H), 0.83 - 0.77 (m, 2H). ^{13}C NMR ($CDCl_3$, TMS, 150 MHz) δ 145.5, 128.1, 40.2, 39.9, 36.5, 32.4, 32.2, 26.4, 26.2, 14.8. IR (neat) ν 699, 922, 950, 1084, 1103, 1284, 1643, 2900 cm^{-1}. HRMS (EI) calcd. for $C_{12}H_{20}S$ (M^+): 194.1122, Found: 194.1124.

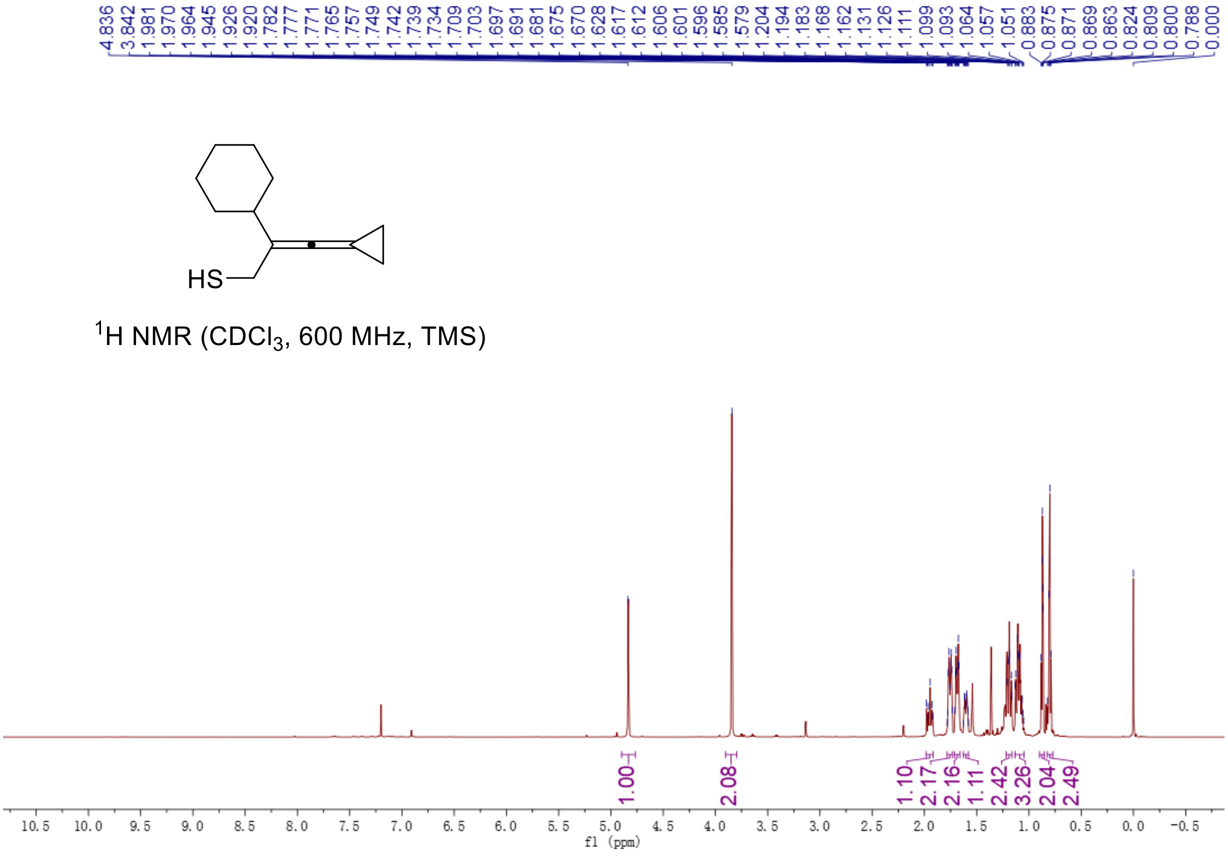

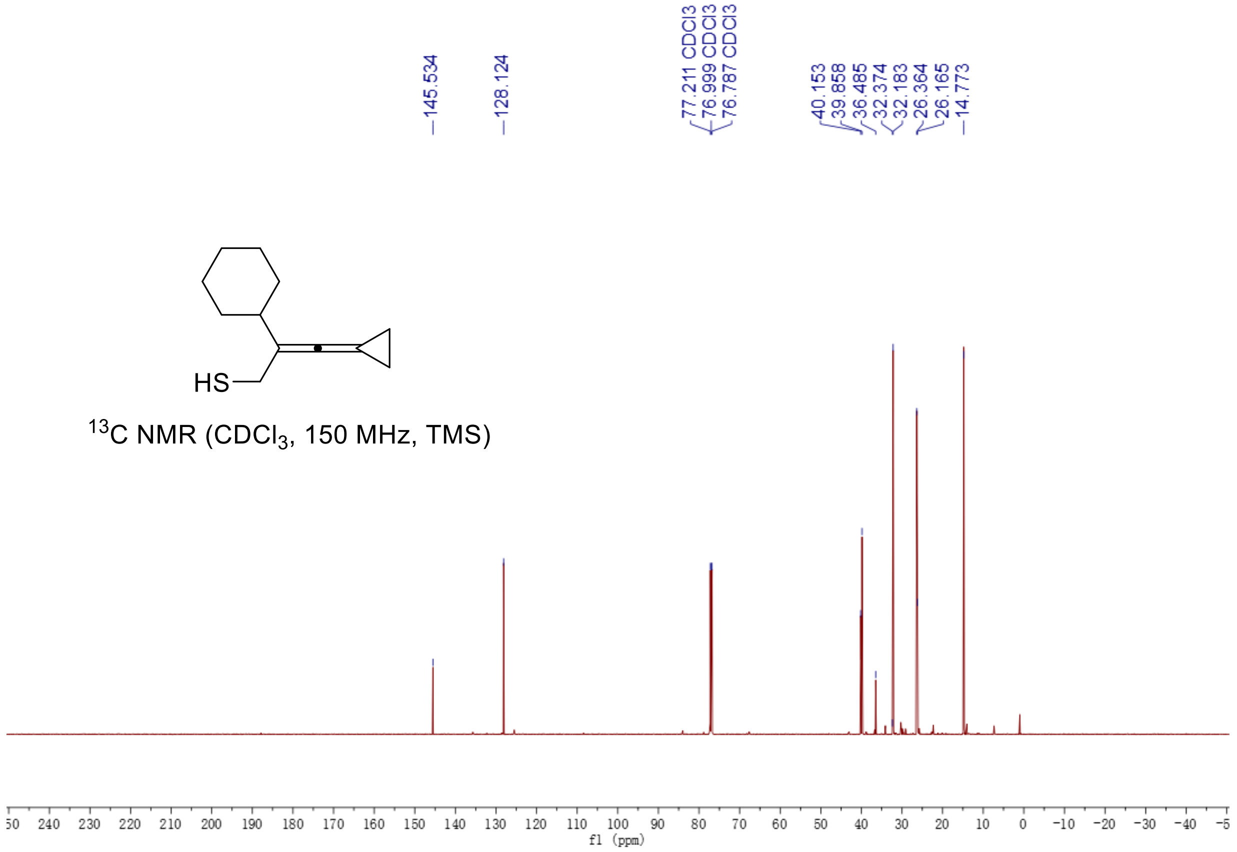

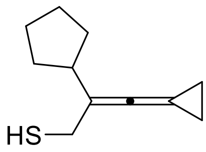

2-cyclopentyl-3-cyclopropylidene-3λ^{5}-prop-2-ene-1-thiol (7d)

A colorless oil, 33%, 59.5 mg. ^{1}H NMR ($CDCl_3$, TMS, 400 MHz) δ 4.94 (s, 1H), 3.91 (s, 2H), 2.56 - 2.42 (m, 1H), 1.88 - 1.79 (m, 2H), 1.72 - 1.63 (m, 2H), 1.61 - 1.56 (m, 2H), 1.47 - 1.34 (m, 2H), 0.97 - 0.92 (m, 2H), 0.90 - 0.86 (m, 2H). ^{13}C NMR ($CDCl_3$, TMS, 150 MHz) δ 143.8, 128.3, 41.9, 40.3, 31.6, 25.0, 22.6, 14.7, 14.1. IR (neat) ν 701, 902, 946, 1101, 1133, 1254, 1601, 2913 cm^{-1}. HRMS (EI) calcd. for $C_{11}H_{16}S$ (M^+): 180.0966, Found: 180.0967.

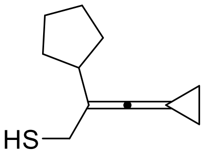

^{1}H NMR ($CDCl_3$, 400 MHz, TMS)

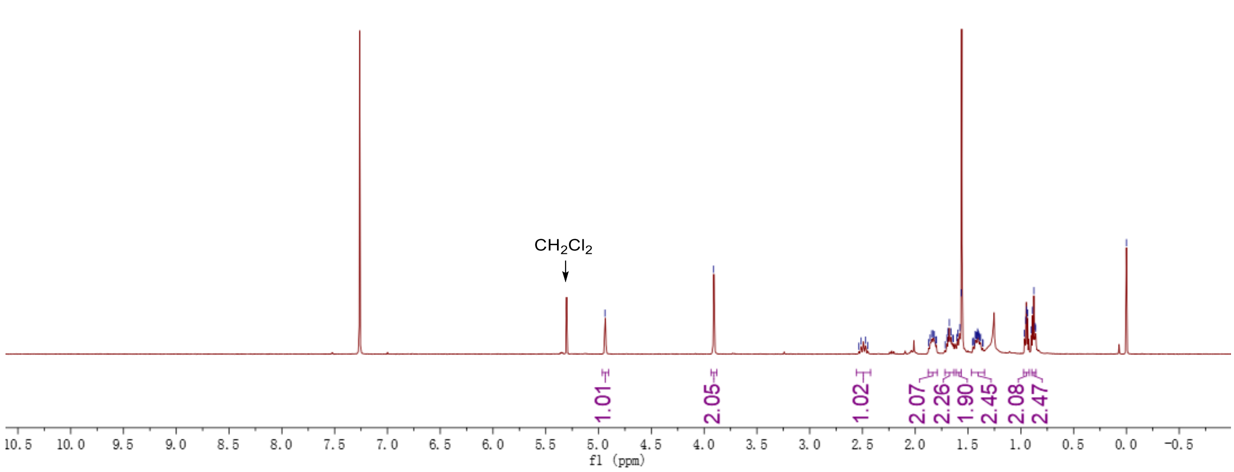

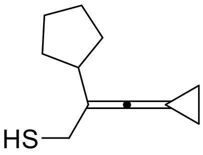

^{13}C NMR ($CDCl_3$, 150 MHz, TMS)

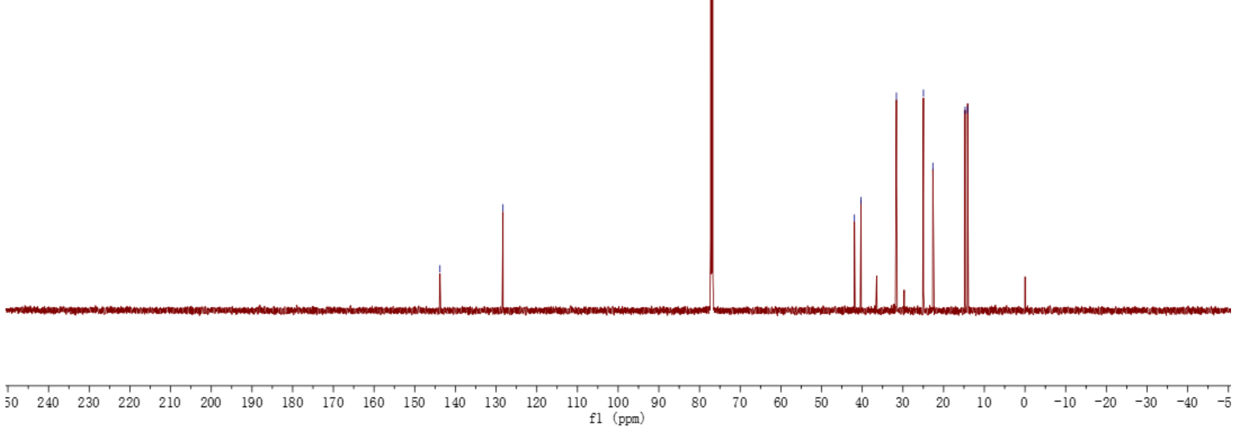

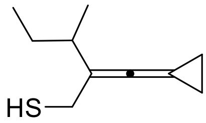

2-(cyclopropylidene-λ⁵-methylene)-3-methylpentane-1-thiol (7e)

A colorless oil, 36%, 60.6 mg. ^{1}H NMR ($CDCl_3$, TMS, 600 MHz) δ 4.93 (s, 1H), 3.90 (d, *J* = 14.2 Hz, 1H), 3.84 (d, *J* = 14.2 Hz, 1H), 2.26 - 2.17 (m, 1H), 1.49 - 1.43 (m, 1H), 1.34 (dq, *J* = 14.4 Hz, 1H), 1.06 (d, *J* = 6.9 Hz, 3H), 0.97 - 0.93 (m, 2H), 0.90 - 0.85 (m, 5H). ^{13}C NMR ($CDCl_3$, TMS, 150 MHz) δ 144.8, 129.4, 39.0, 37.2, 36.3, 28.1, 19.1, 14.8, 11.7. IR (neat) ν 720, 902, 961, 1034, 1083, 1264, 1603, 2920 cm^{-1}. HRMS (EI) calcd. for $C_{10}H_{16}S$ (M^+): 168.0966, Found: 168.0967.

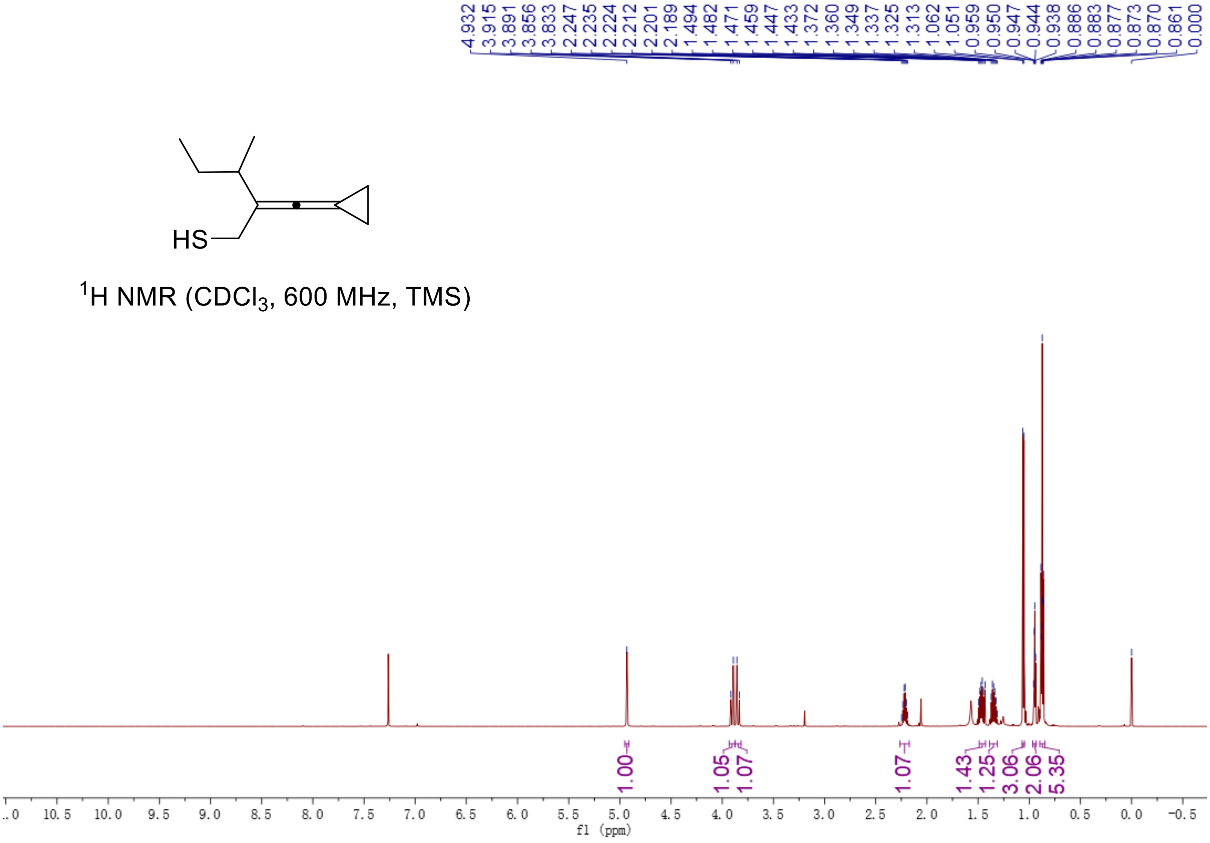

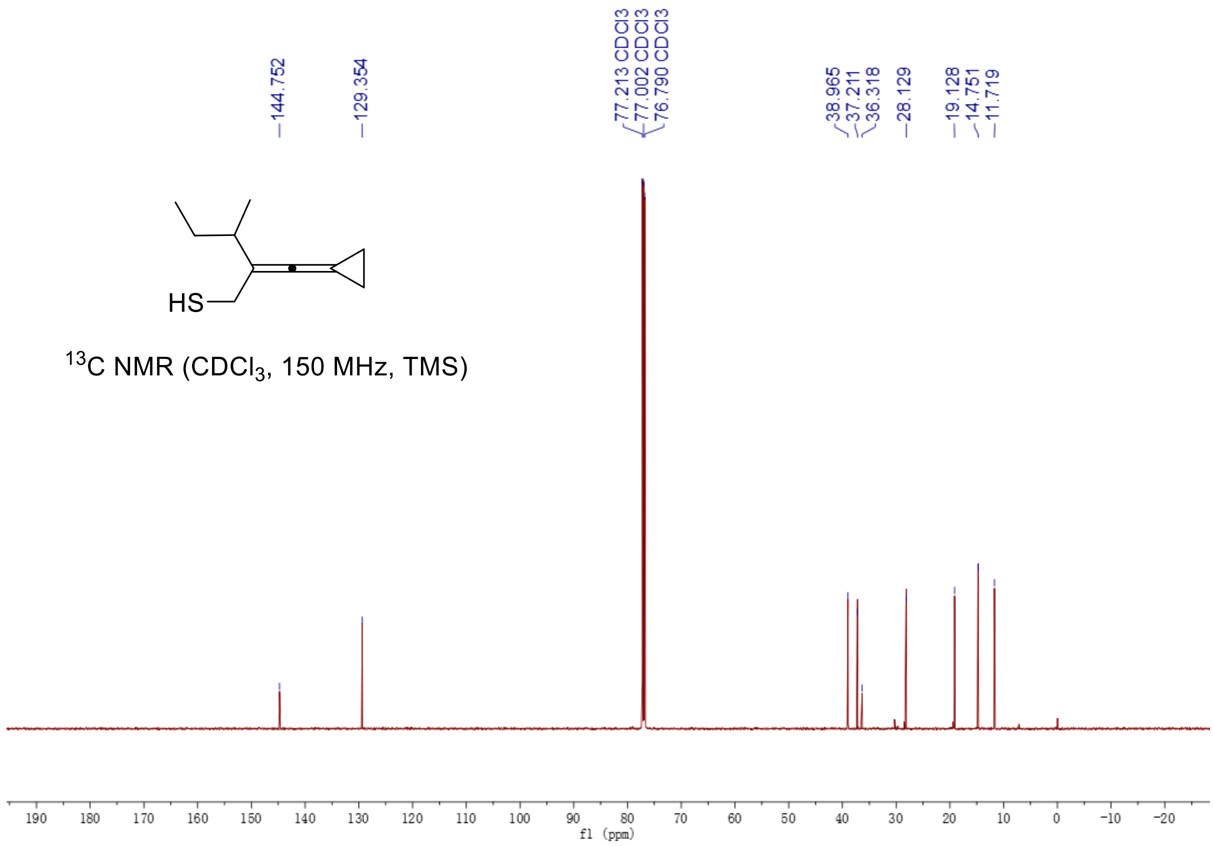

^{13}C NMR ($CDCl_3$, 150 MHz, TMS)

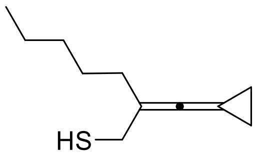

2-(cyclopropylidene-λ⁵-methylene)heptane-1-thiol (7f)

A colorless oil, 40%, 72.9 mg. ^{1}H NMR ($CDCl_3$, TMS, 600 MHz) δ 4.93 (s, 1H), 3.87 (s, 2H), 2.09 (t, *J* = 7.7 Hz, 2H), 1.49 - 1.43 (m, 2H), 1.34 - 1.26 (m, 4H), 0.96 - 0.93 (m, 2H), 0.91 - 0.86 (m, 5H). ^{13}C NMR ($CDCl_3$, TMS, 150 MHz) δ 140.4, 129.7, 41.3, 36.6, 31.6, 31.2, 27.6, 22.5, 14.6, 14.0. IR (neat) ν 715, 916, 931, 1064, 1093, 1224, 1613, 2933 cm^{-1}. HRMS (EI) calcd. for $C_{11}H_{18}S$ (M^+): 182.1122, Found: 182.1124.

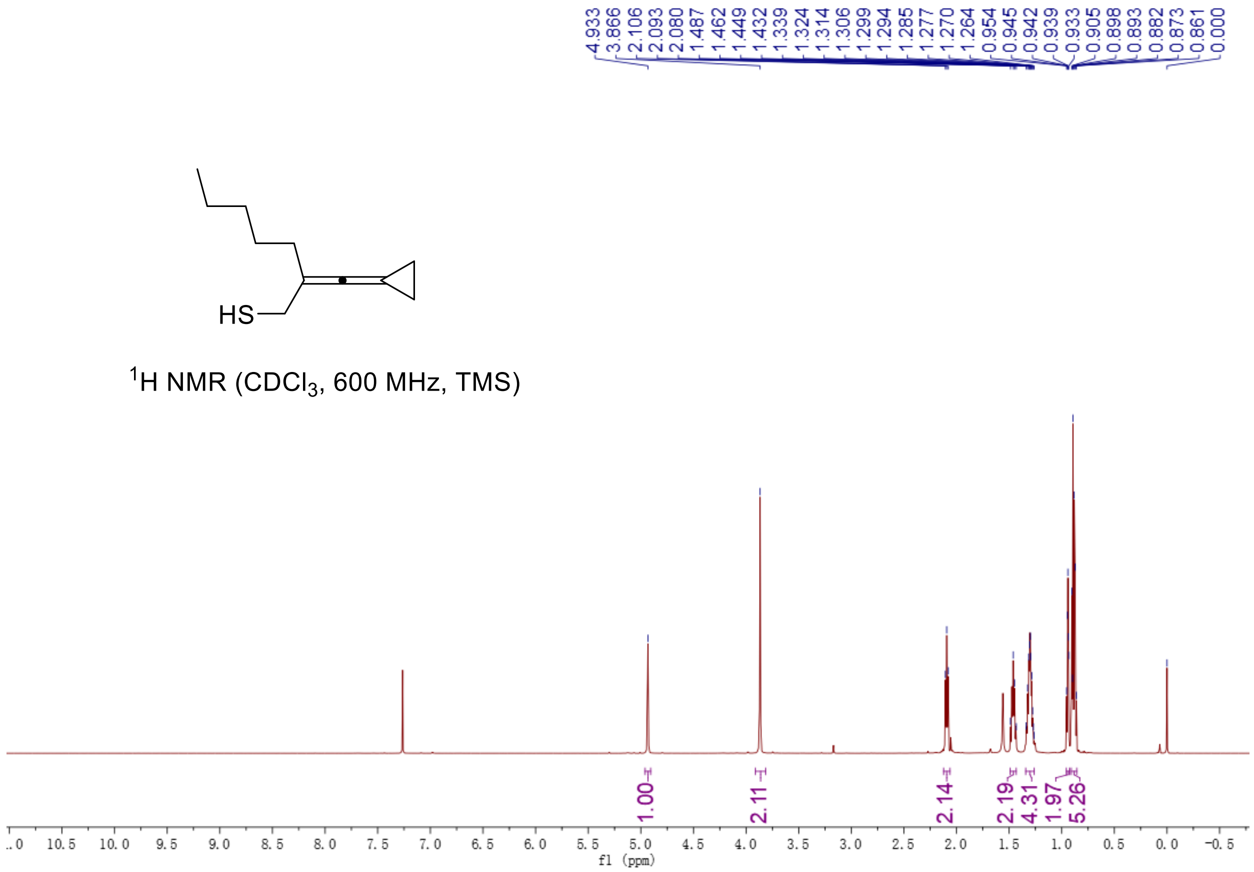

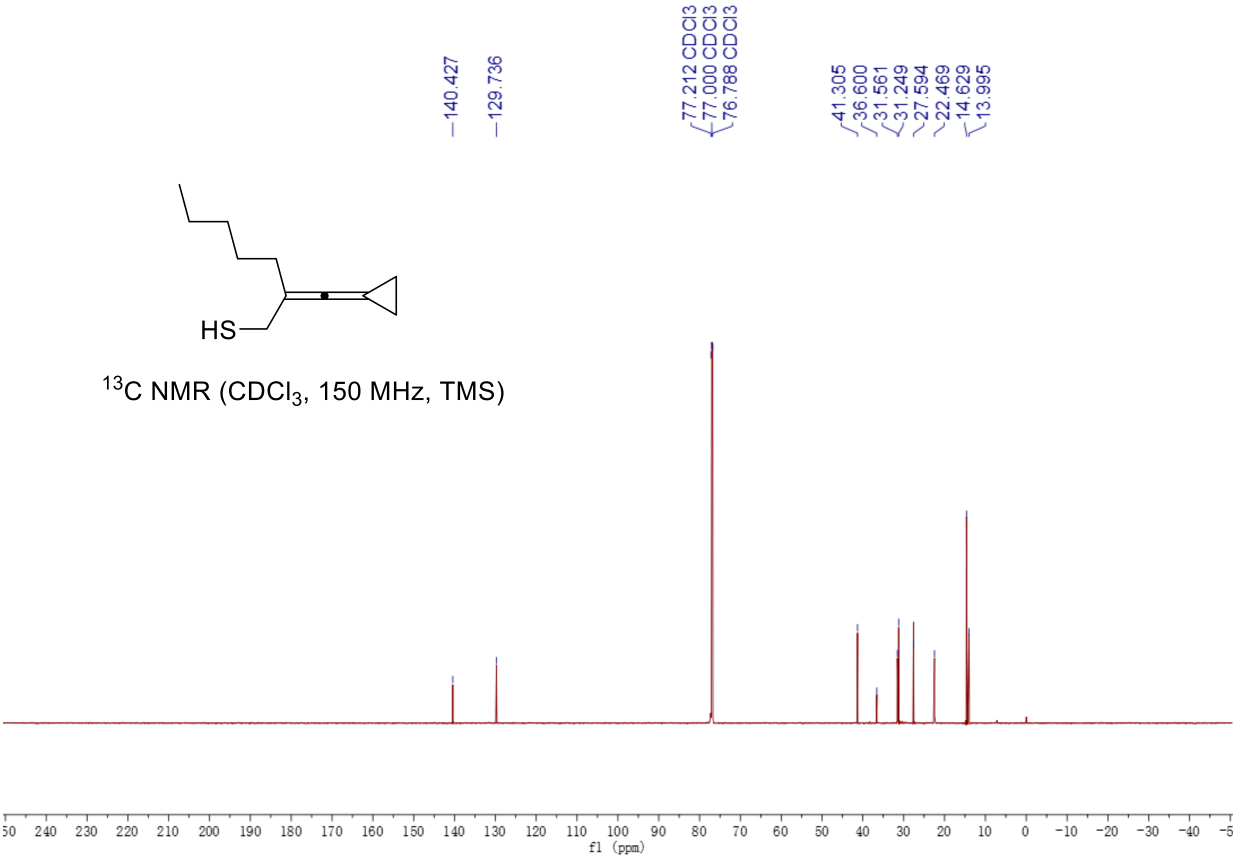

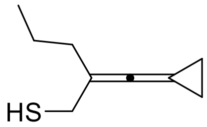

2-(cyclopropylidene-λ^5-methylene)pentane-1-thiol (7g)

A colorless oil, 46%, 71.0 mg. ^{1}H NMR ($CDCl_3$, TMS, 600 MHz) δ 4.94 - 4.93 (m, 1H), 3.87 - 3.86 (m, 2H), 2.10 - 2.06 (m, 2H), 1.52 - 1.46 (m, 2H), 0.96 - 0.90 (m, 5H), 0.89 - 0.86 (m, 2H). ^{13}C NMR ($CDCl_3$, TMS, 150 MHz) δ 140.2, 129.9, 41.3, 36.6, 33.4, 21.1, 14.6, 13.9. IR (neat) ν 716, 921, 965, 1033, 1088, 1270, 1611, 2914 cm^{-1}. HRMS (EI) calcd. for $C_9H_{14}S$ (M^+): 154.0811, Found: 154.0811.

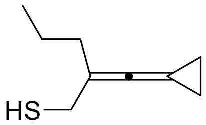

^{1}H NMR ($CDCl_3$, 600 MHz, TMS)

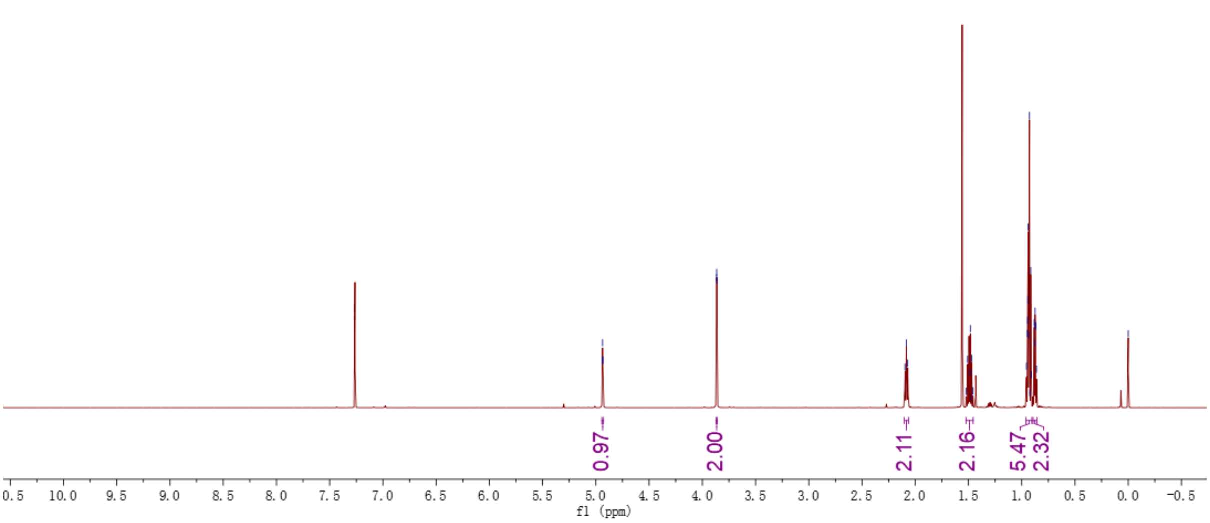

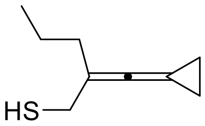

^{13}C NMR ($CDCl_3$, 150 MHz, TMS)

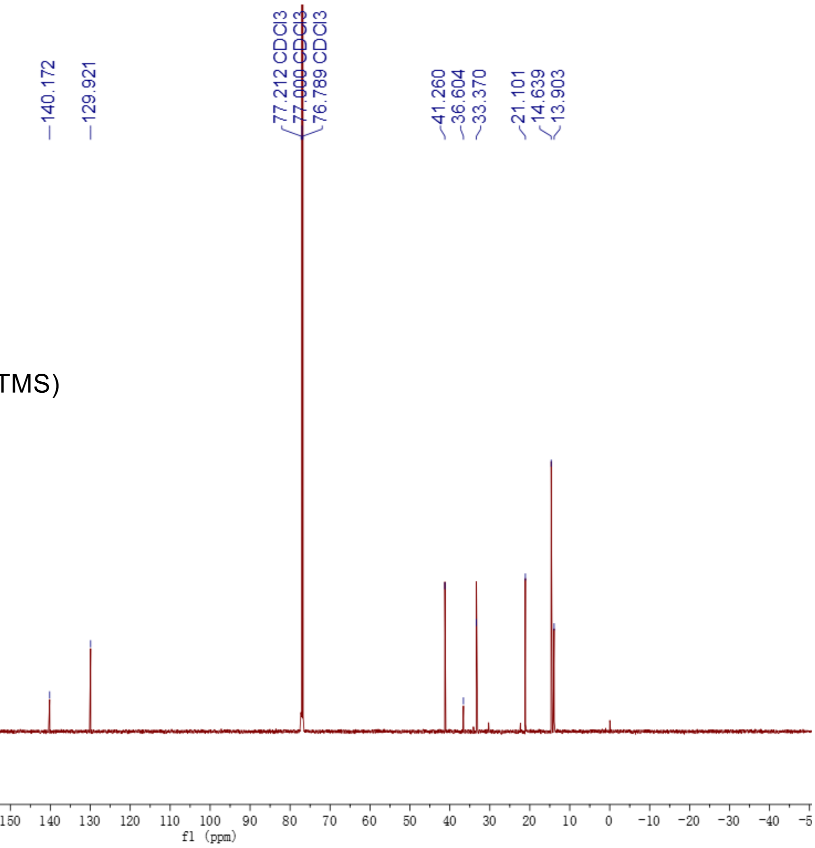

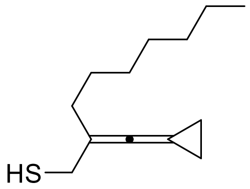

2-(cyclopropylidene-λ^{5}-methylene)nonane-1-thiol (7h)

A colorless oil, 39%, 82.0 mg. ^{1}H NMR ($CDCl_3$, TMS, 600 MHz) δ 4.93 (s, 1H), 3.86 (s, 2H), 2.11 - 2.07 (m, 2H), 1.48 - 1.43 (m, 2H), 1.31 - 1.26 (m, 8H), 0.96 - 0.93 (m, 2H), 0.90 - 0.86 (m, 5H). ^{13}C NMR ($CDCl_3$, TMS, 150 MHz) δ 140.4, 129.7, 61.8, 41.3, 36.6, 31.8, 31.3, 29.3, 29.1, 27.9, 22.6, 14.6, 14.1. IR (neat) ν 718, 900, 966, 1038, 1072, 1253, 1631, 2928 cm^{-1}. HRMS (EI) calcd. for $C_{13}H_{22}S$ (M^{+}): 210.1439, Found: 210.1437.

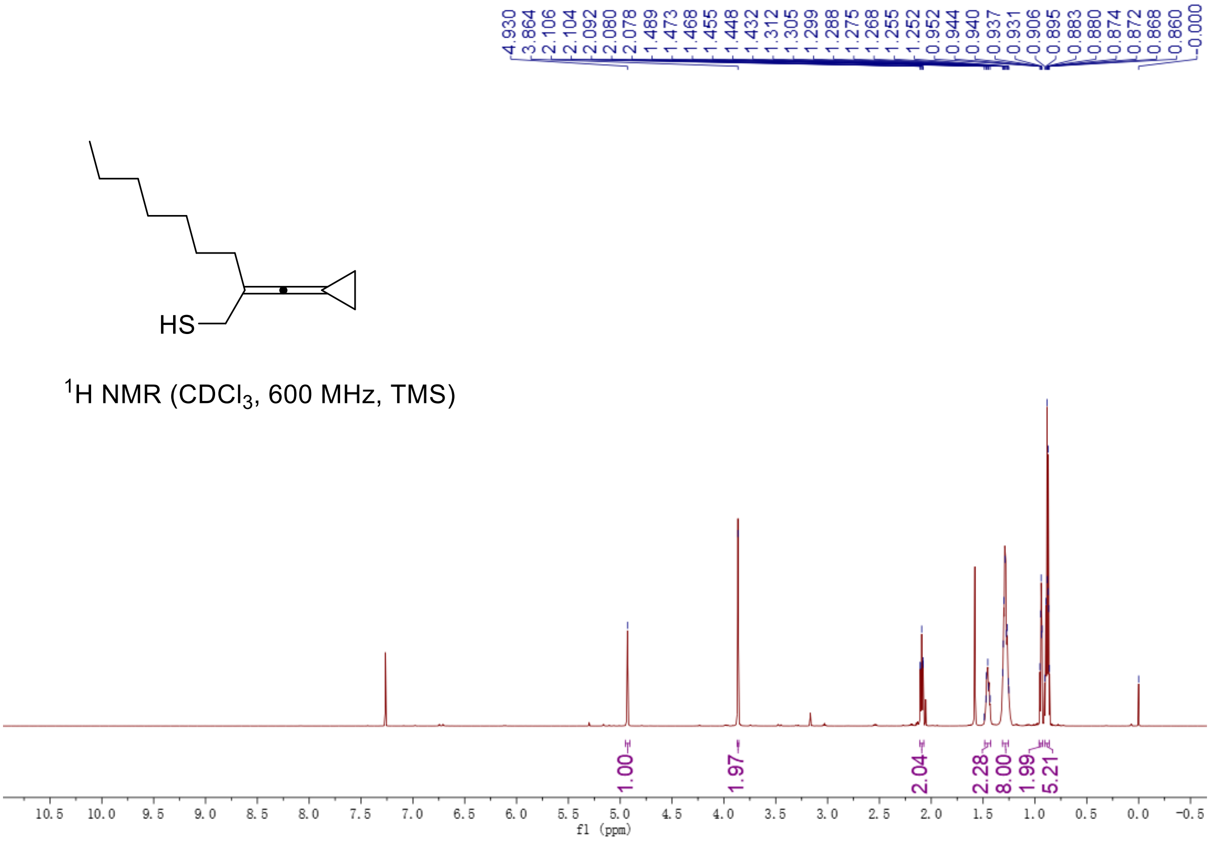

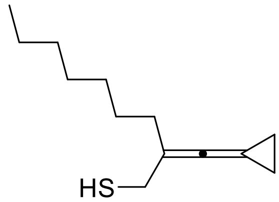

^{13}C NMR ($CDCl_3$, 150 MHz, TMS)

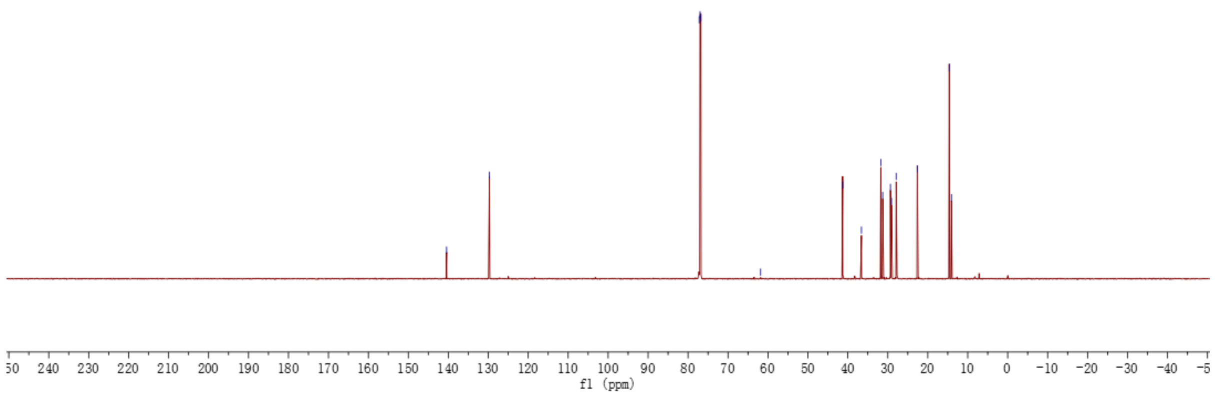

9. Spectroscopic data of products 3, 4, 6, 8, 9, 11, 12, 13.

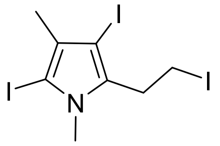

2,4-diiodo-5-(2-iodoethyl)-1,3-dimethyl-1H-pyrrole (3aa)

A colorless oil, 45.1 mg, 95% yield. ^{1}H NMR ($CDCl_3$, TMS, 600 MHz) δ 3.60 (s, 3H), 3.31 - 3.23 (m, 2H), 3.18 - 3.10 (m, 2H), 2.04 (s, 3H). ^{13}C NMR ($CDCl_3$, TMS, 150 MHz) δ 136.8, 133.2, 126.6, 117.0, 71.1, 68.5, 51.4, 33.1, 16.7, 1.9. IR (Neat) ν 722, 1007, 1051, 1168, 1332, 1387, 1431, 2843, 2920 cm^{-1}. HRMS (EI) calcd. for $C_8H_{10}I_3N$: 500.7947, Found: 500.7942.

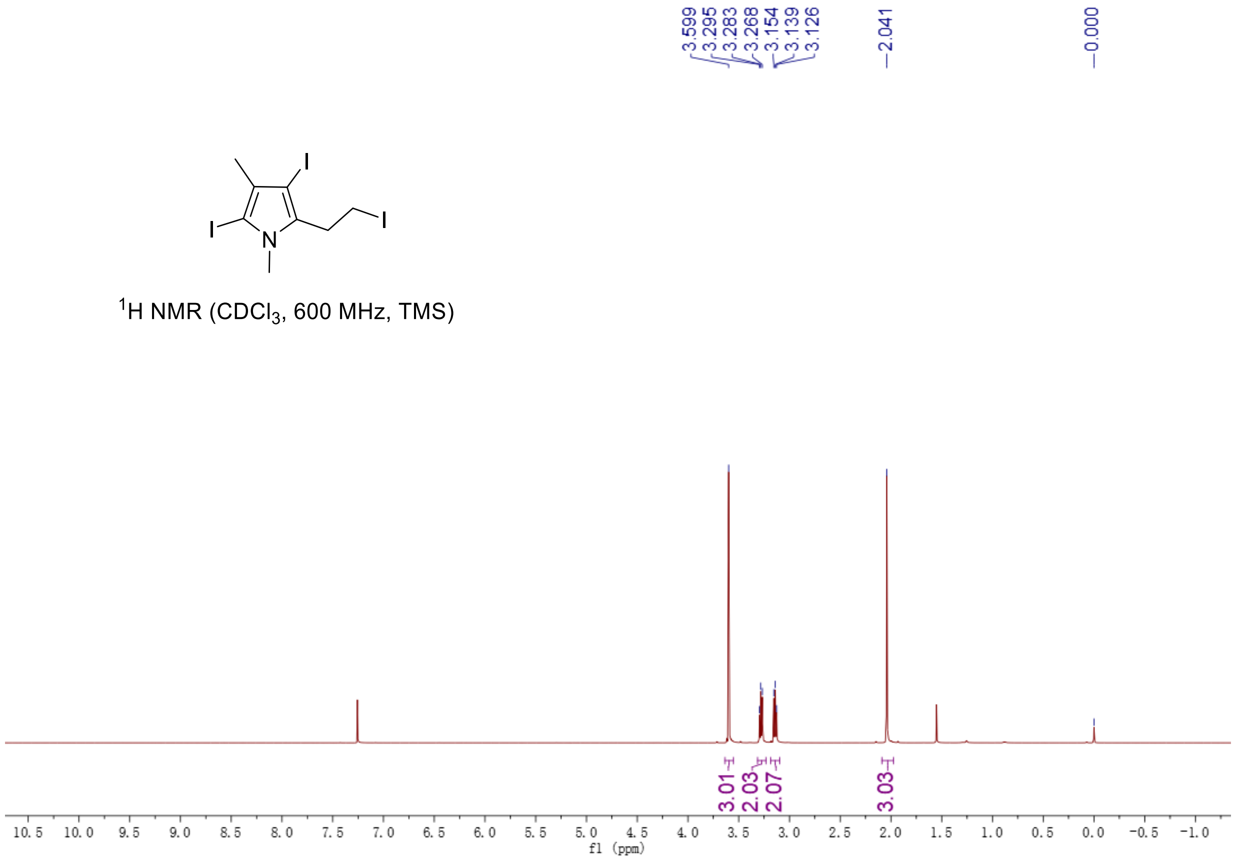

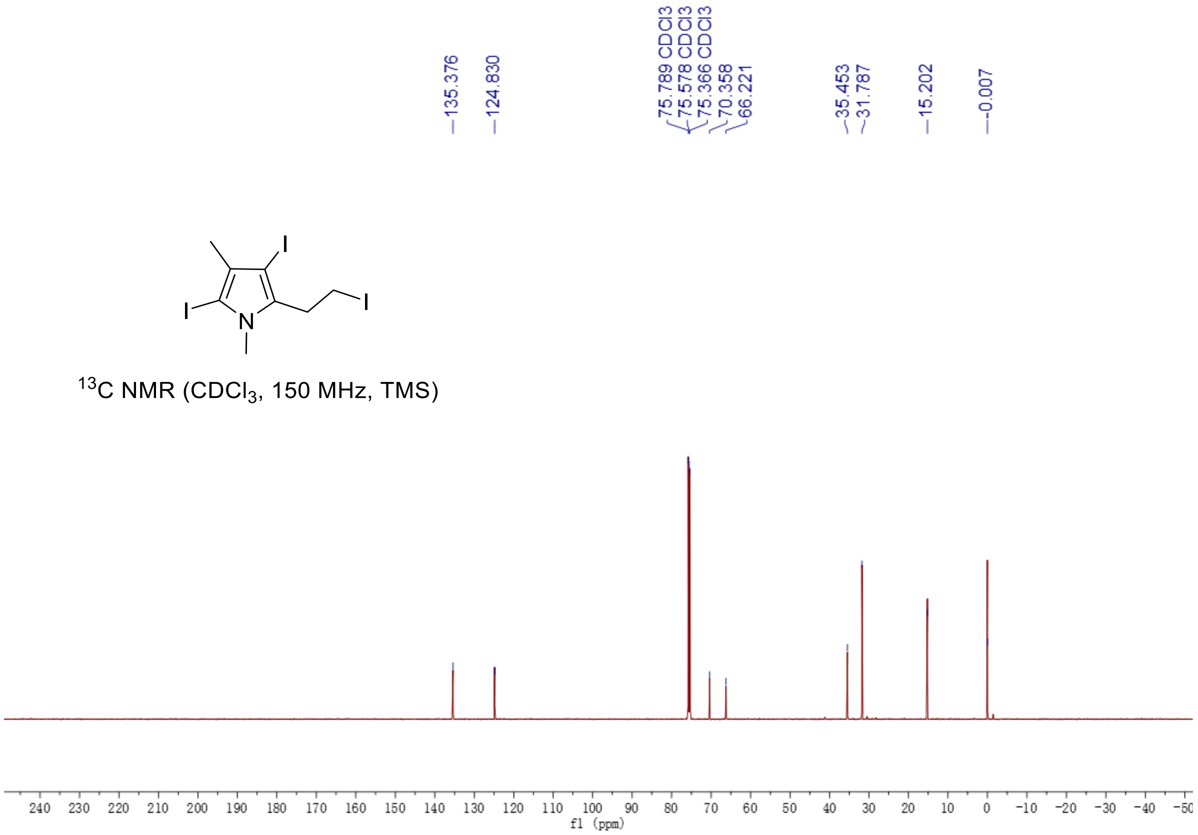

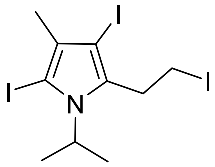

2,4-diiodo-5-(2-iodoethyl)-1-isopropyl-3-methyl-1H-pyrrole (3ba)

A colorless oil, 51.6 mg, 20% yield. ^{1}H NMR ($CDCl_3$, TMS, 600 MHz) 4.63 (s, 1H), 3.29 (s, 2H), 3.14 (s, 2H), 2.04 (s, 3H), 1.52 (s, 6H). ^{13}C NMR ($CDCl_3$, TMS, 150 MHz) δ 135.9, 126.2, 114.2, 54.5, 34.2, 22.6, 17.0, 1.5. IR (Neat) ν 2960, 2917, 1486, 1456, 1392, 1352, 1311, 1219, 1069, 1035, 1008, 820, 748, 735, 721 cm^{-1}. HRMS (ESI) calcd. for $C_{10}H_{15}I_3N$ (M+H): 529.8260, Found: 529.8271.

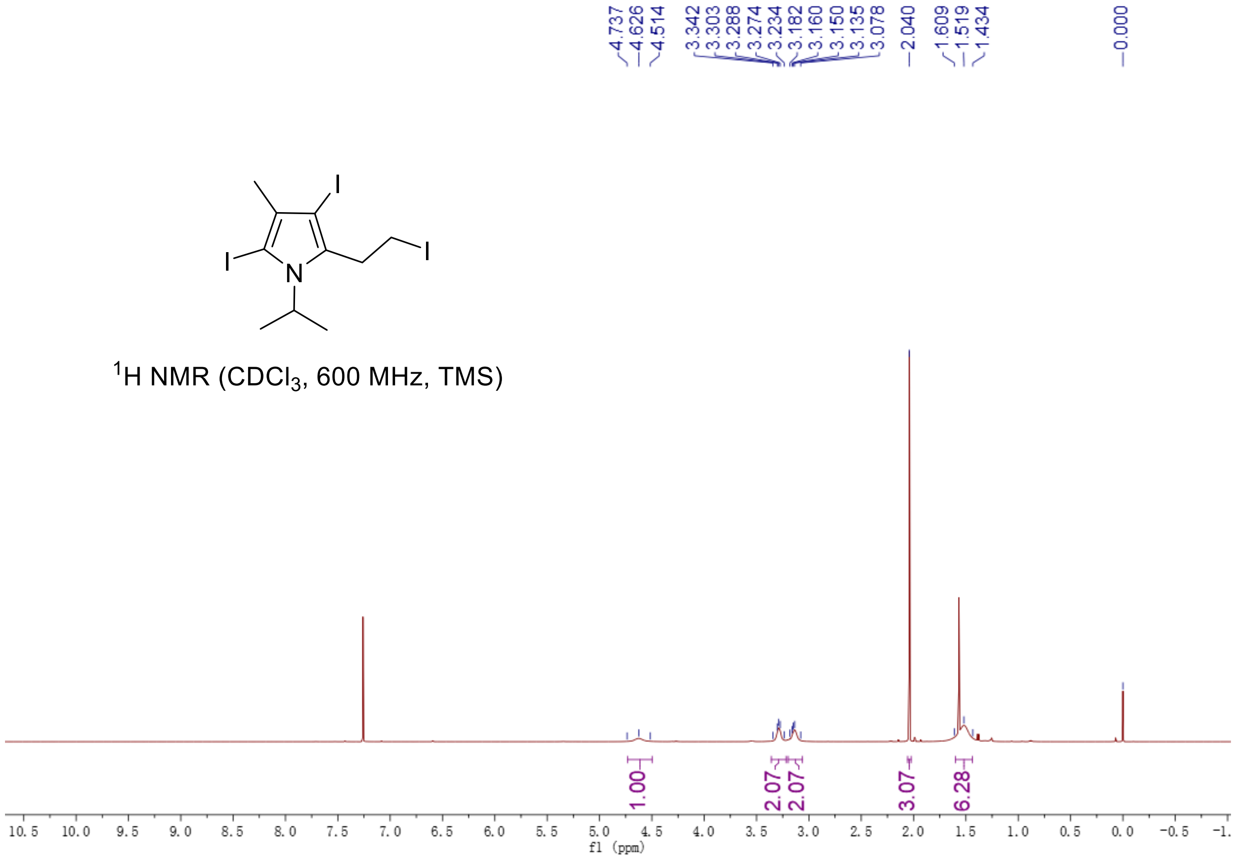

^{1}H NMR ($CDCl_3$, 600 MHz, TMS)

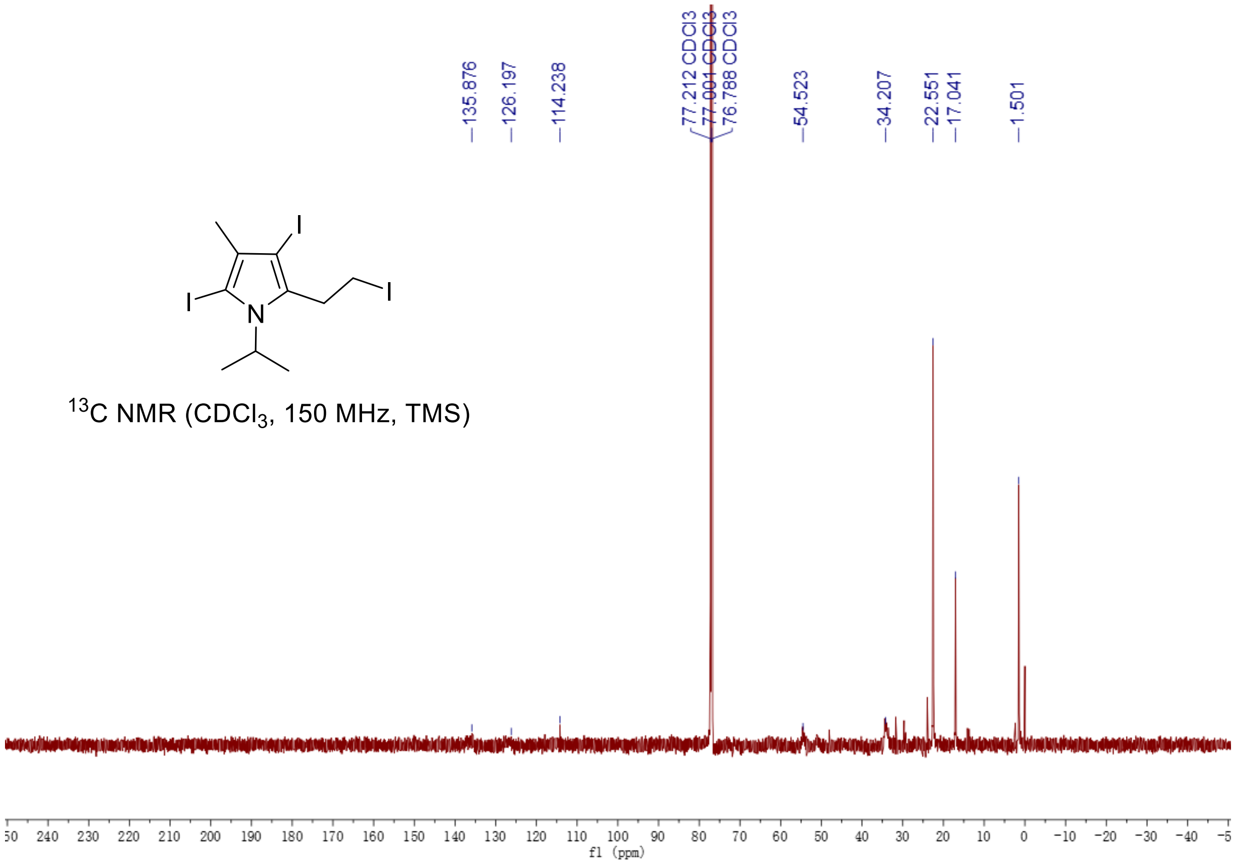

^{13}C NMR ($CDCl_3$, 150 MHz, TMS)

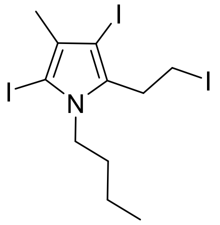

1-butyl-2,4-diiodo-5-(2-iodoethyl)-3-methyl-1H-pyrrole (3ca)

A colorless oil, 48.3 mg, 89% yield. ^{1}H NMR ($CDCl_3$, TMS, 600 MHz) δ 3.93 - 3.86 (m, 2H), 3.28 - 3.20 (m, 2H), 3.18 - 3.12 (m, 2H), 2.03 (s, 3H), 1.63 - 1.56 (m, 3H), 1.43 - 1.33 (m, 2H), 0.98 (t, *J* = 7.4 Hz, 3H). ^{13}C NMR ($CDCl_3$, TMS, 150 MHz) δ 136.3, 126.3, 70.5, 68.0, 49.3, 33.6, 33.2, 32.7, 19.85, 19.83, 16.6, 13.8, 1.7. IR (Neat) ν 731, 1009, 1099, 1244, 1380, 1465, 2864, 2956 cm^{-1}. HRMS (EI) calcd. for $C_{11}H_{16}I_3N$: 542.8428, Found: 542.8411.

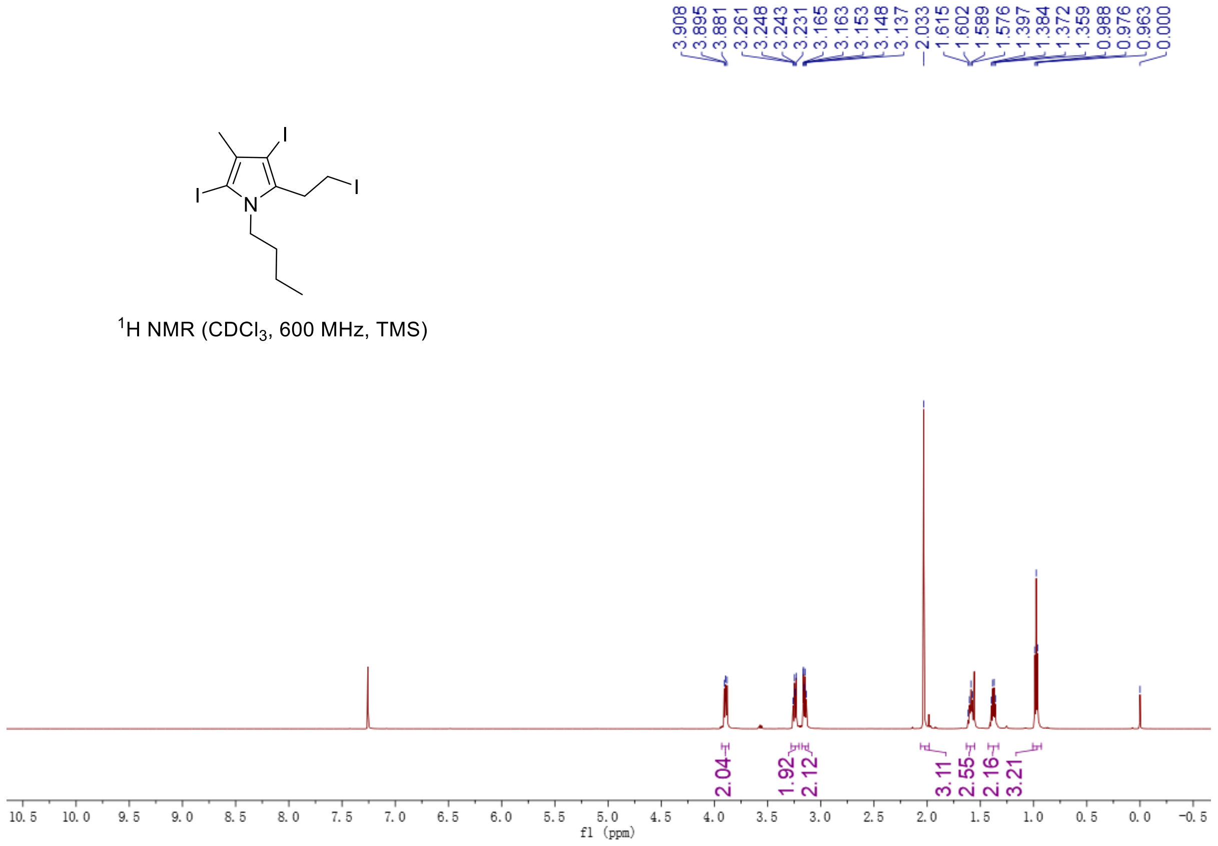

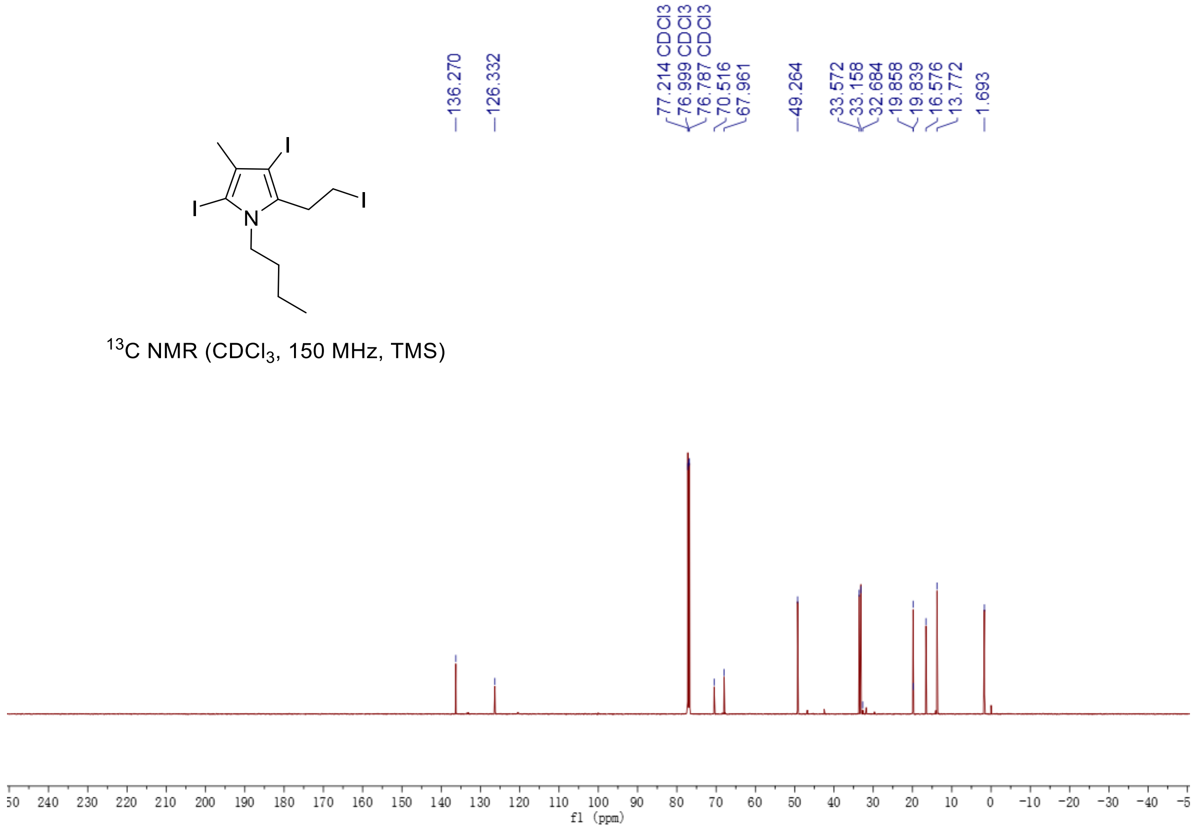

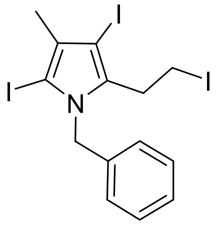

1-benzyl-2,4-diiodo-5-(2-iodoethyl)-3-methyl-1H-pyrrole (3da)

A colorless oil, 41.5 mg, 72% yield. ^{1}H NMR ($CDCl_3$, TMS, 400 MHz) δ 7.37 - 7.27 (m, 3H), 6.95 (d, J = 7.5 Hz, 2H), 5.21 (s, 2H), 3.21 - 3.10 (m, 2H), 2.90 - 2.79 (m, 2H), 2.10 (s, 3H). ^{13}C NMR ($CDCl_3$, TMS, 100 MHz) δ 137.1, 129.0, 127.7, 126.8, 125.9, 72.1, 69.1, 52.7, 33.3, 16.8, 1.5. IR (Neat) ν 693, 727, 1029, 1173, 1443, 1495, 2927 cm^{-1}. HRMS (ESI) calcd. for $C_{14}H_{15}I_3N$ (M+H): 577.8342, Found: 577.8490.

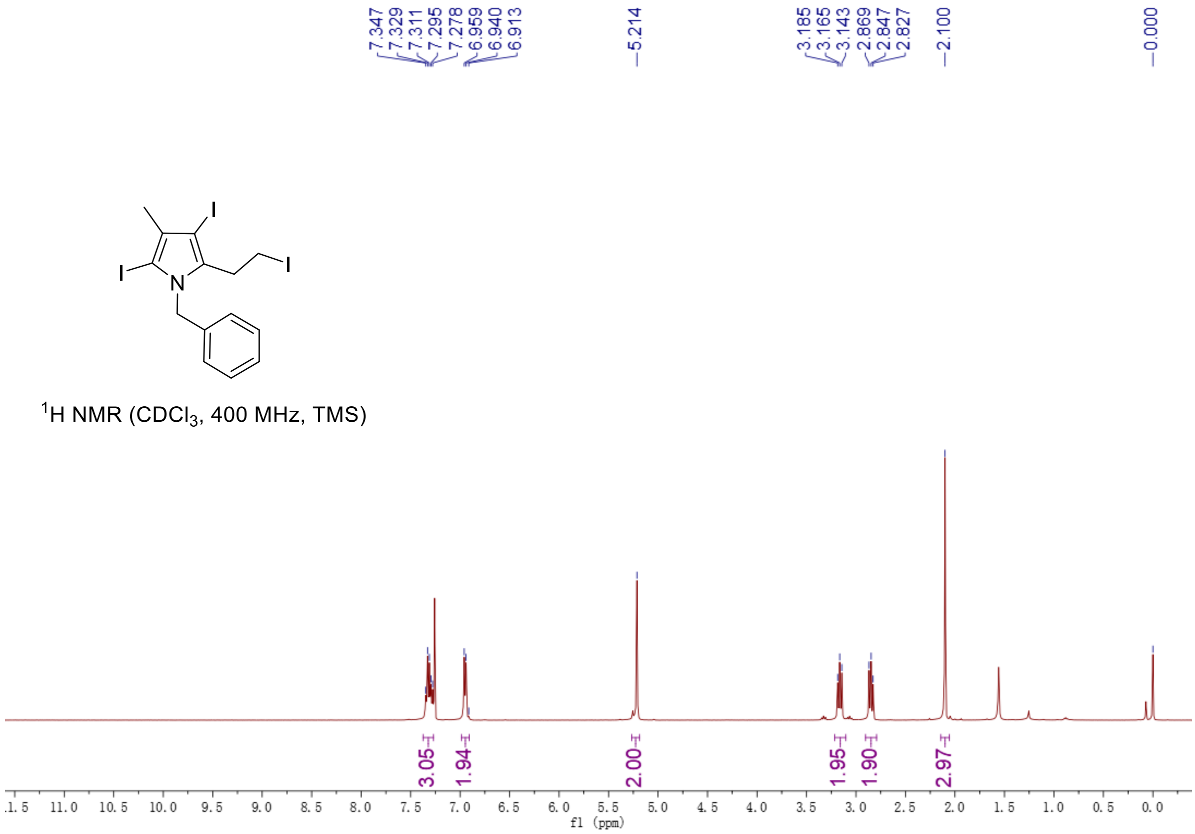

^{1}H NMR ($CDCl_3$, 400 MHz, TMS)

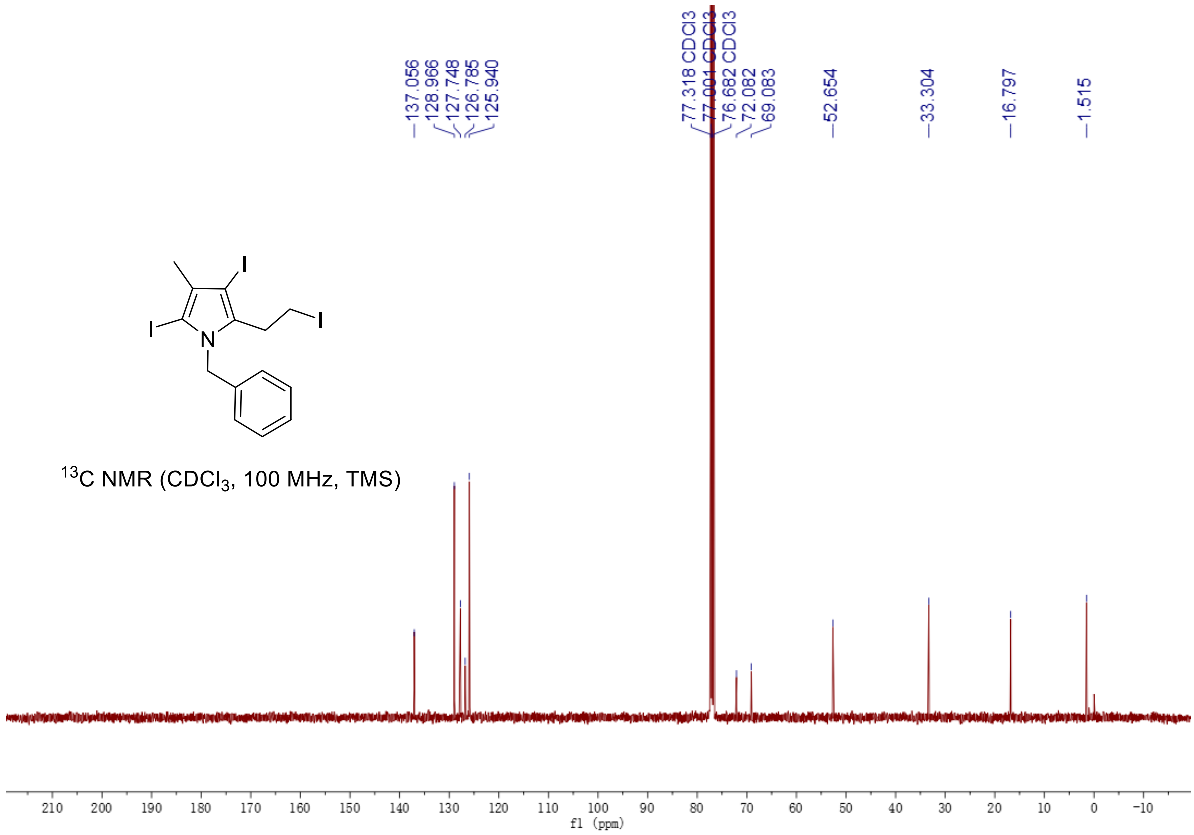

^{13}C NMR ($CDCl_3$, 100 MHz, TMS)

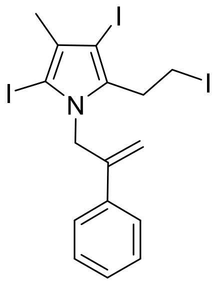

2,4-diiodo-5-(2-iodoethyl)-3-methyl-1-(2-phenylallyl)-1H-pyrrole (3ea)

A colorless oil, 40.2 mg, 82% yield. ^{1}H NMR ($CDCl_3$, TMS, 400 MHz) δ 7.50 - 7.32 (m, 5H), 5.38 (s, 1H), 4.91 (s, 2H), 4.25 (s, 1H), 3.26 - 3.14 (m, 4H), 2.10 (s, 3H). ^{13}C NMR ($CDCl_3$, TMS, 100 MHz) δ 143.7, 138.2, 136.9, 128.7, 128.4, 126.7, 125.9, 113.1, 71.9, 68.7, 52.5, 33.1, 16.7, 2.1. IR (Neat) ν 705, 779, 898, 1164, 1257, 1333, 1430, 2846, 2922 cm^{-1}. HRMS (EI) calcd. for $C_{16}H_{16}I_3N$: 602.8436, Found: 602.8411.

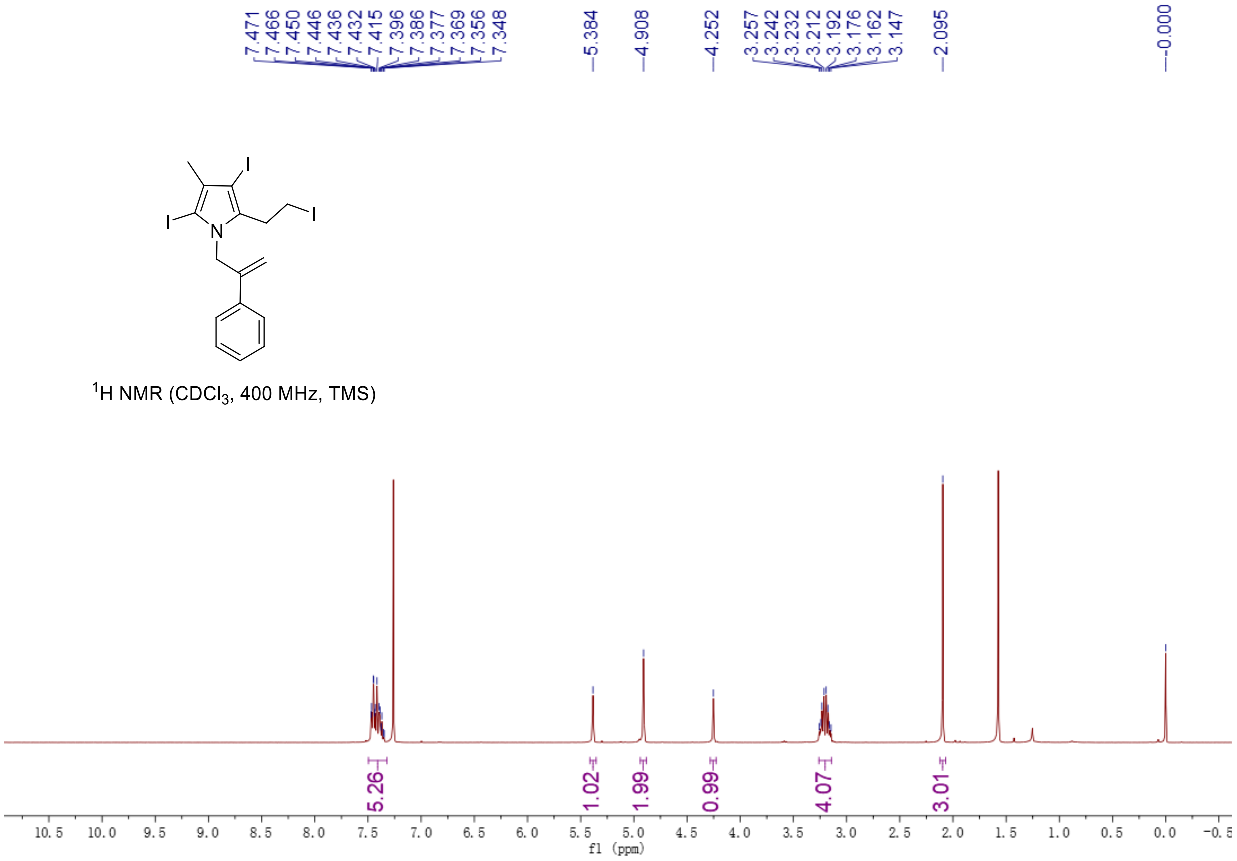

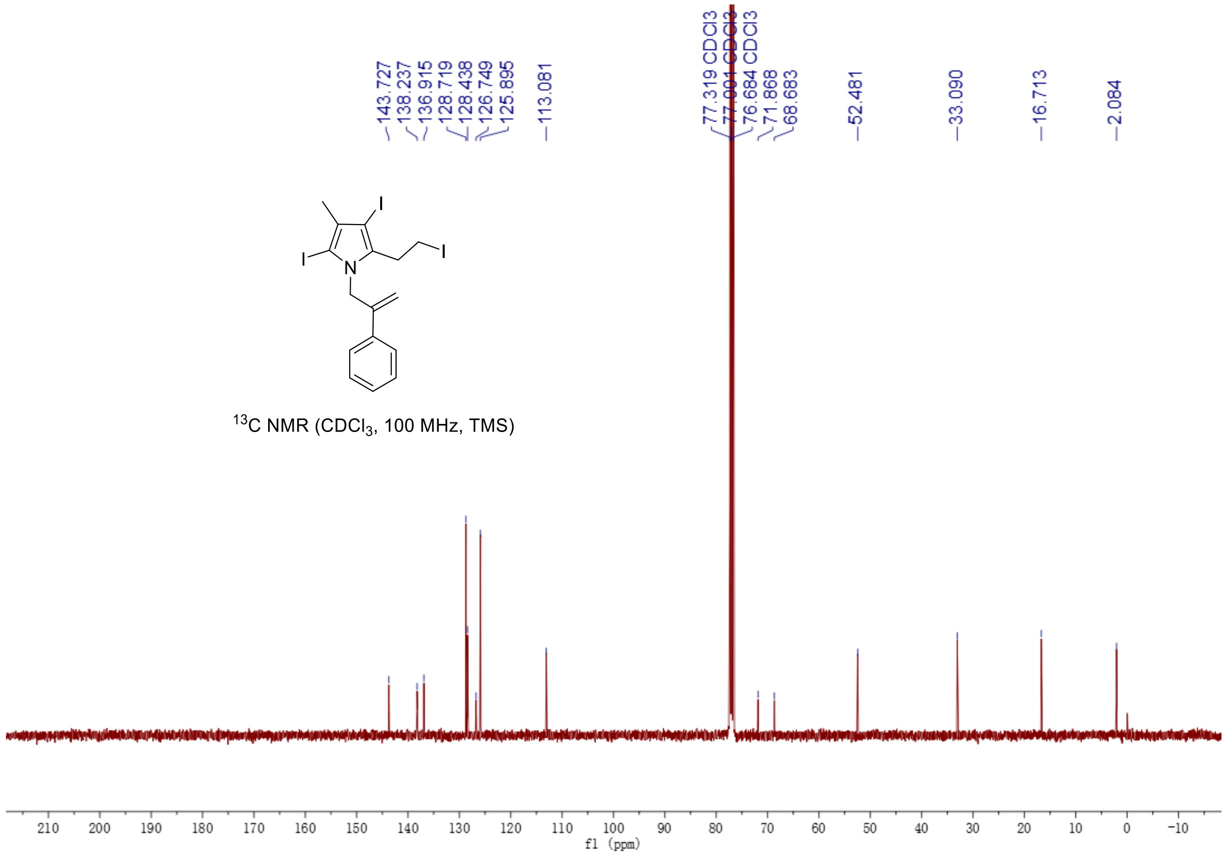

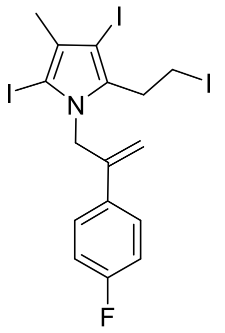

1-(2-(4-fluorophenyl)allyl)-2,4-diiodo-5-(2-iodoethyl)-3-methyl-1H-pyrrole (3fa)

A colorless oil, 48.4 mg, 78% yield. ^{1}H NMR ($CDCl_3$, TMS, 400 MHz) δ δ 7.47 - 7.37 (m, 2H), 7.15 - 7.04 (m, 2H), 5.33 (s, 1H), 4.87 (s, 2H), 4.26 (s, 1H), 3.27 - 3.12 (m, 4H), 2.09 (s, 3H). ^{13}C NMR ($CDCl_3$, TMS, 100 MHz) δ 162.8 (d, J = 240.0 Hz) 142.8, 136.9, 134.3 (d, J = 3.6 Hz), 127.6 (d, J = 8.0 Hz), 126.9, 115.6 (d, J = 21.6 Hz), 113.2, 71.8, 68.8, 52.5, 33.1, 16.7, 2.0. ^{19}F NMR ($CDCl_3$, TMS, 376 MHz) δ -113.3. IR (Neat) ν 734, 835, 906, 1161, 1233, 1331, 1399, 1508, 1602, 2919 cm^{-1}. HRMS (EI) calcd. for $C_{16}H_{15}FI_3N$: 620.8336, Found: 620.8317.

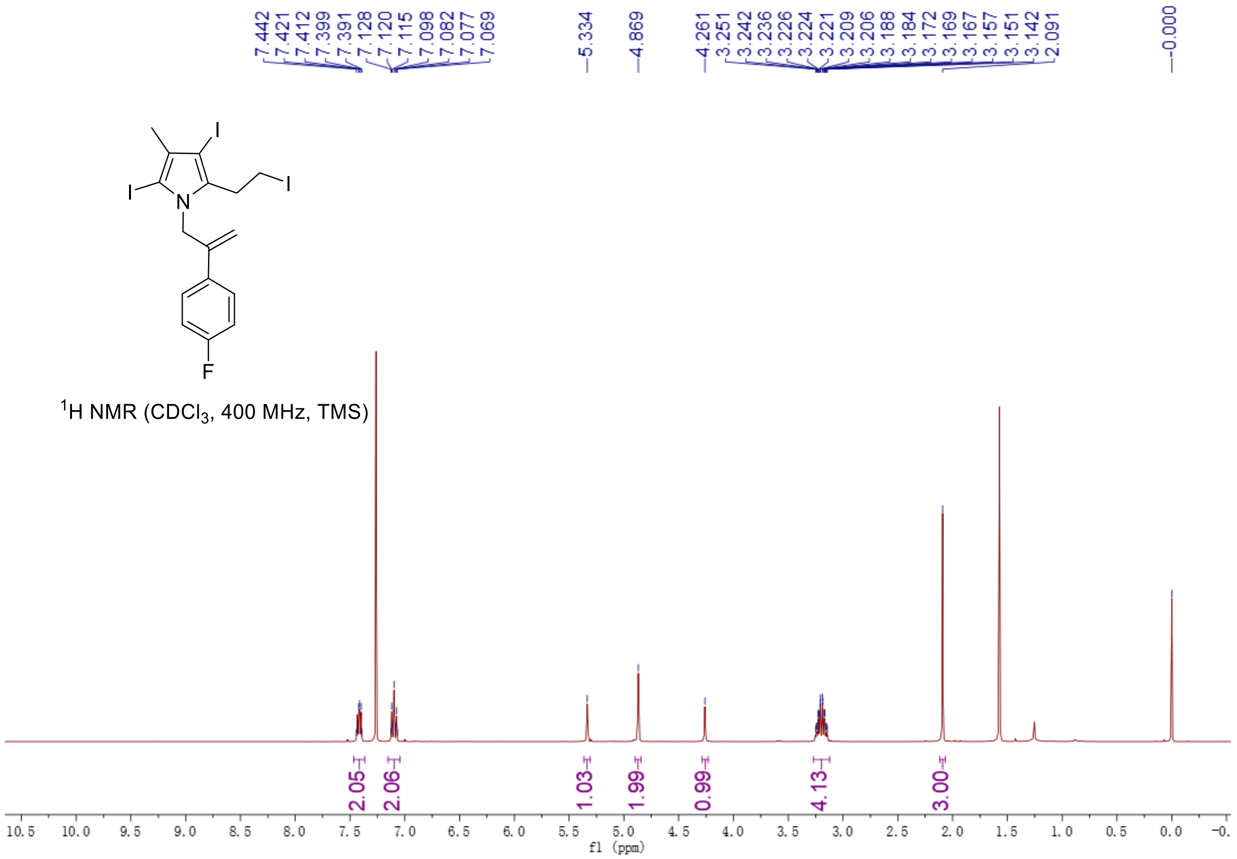
7.442
7.421
7.412
7.399
7.391
7.128
7.120
7.115
7.098
7.082
7.077
7.069
5.334
4.869
4.261
3.251
3.242
3.236
3.226
3.224
3.221
3.209
3.206
3.188
3.184
3.172
3.169
3.167
3.157
3.151
3.142
2.091
0.000
I
I
I
N
F
^{1}H NMR ($CDCl_3$, 400 MHz, TMS)
2.05
2.06
1.03
1.99
0.99
4.13
3.00
f1 (ppm)

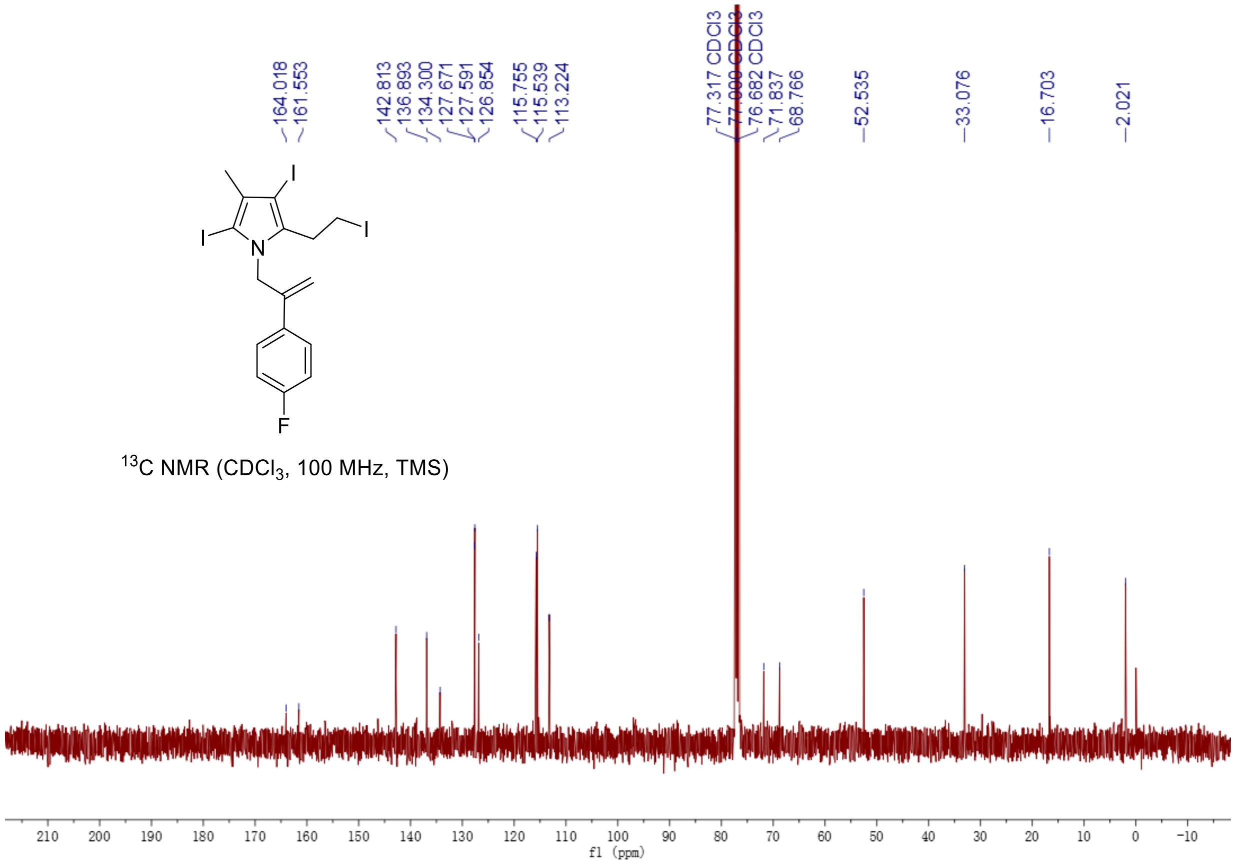
164.018
161.553
142.813
136.893
134.300
127.671
127.591
126.854
115.755
115.539
113.224
77.317 CDCl3
77.000 CDCl3
76.682 CDCl3
71.837
68.766
52.535
33.076
16.703
2.021
I
I
I
N
F
^{13}C NMR ($CDCl_3$, 100 MHz, TMS)
f1 (ppm)

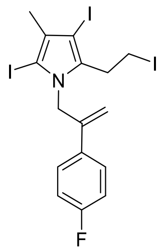

^{19}F NMR ($CDCl_3$, 376 MHz, TMS)

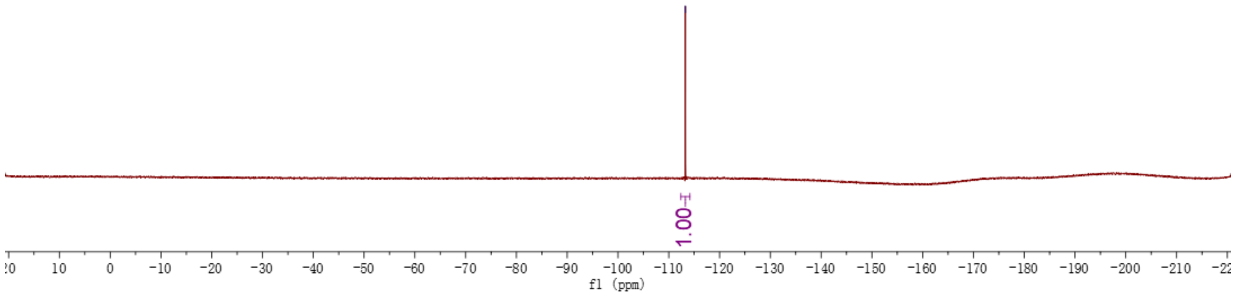

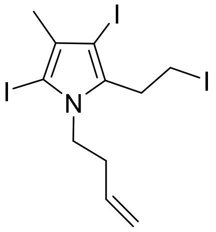

1-(but-3-en-1-yl)-2,4-diiodo-5-(2-iodoethyl)-3-methyl-1H-pyrrole (3ga)

A colorless oil, 48.1 mg, 89% yield. ^{1}H NMR ($CDCl_3$, TMS, 600 MHz) δ 5.80 - 5.72 (m, 1H), 5.17 - 5.09 (m, 2H), 4.00 - 3.94 (m, 2H), 3.27 - 3.23 (m, 2H), 3.18 - 3.14 (m, 2H), 2.40 - 2.32 (m, 2H), 2.04 (s, 3H). ^{13}C NMR ($CDCl_3$, TMS, 100 MHz) δ 136.4, 133.4, 126.6, 118.0, 70.3, 68.2, 48.7, 35.6, 33.1, 16.6, 1.7. IR (Acetone) ν 927, 989, 1165, 1260, 1323, 1446, 2961 cm^{-1}. HRMS (ESI) calcd. for $C_{11}H_{15}I_3N$ (M+H): 541.8333, Found: 541.8342.

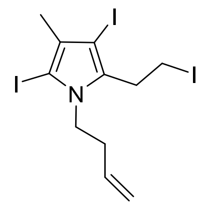

^{1}H NMR ($CDCl_3$, 600 MHz, TMS)

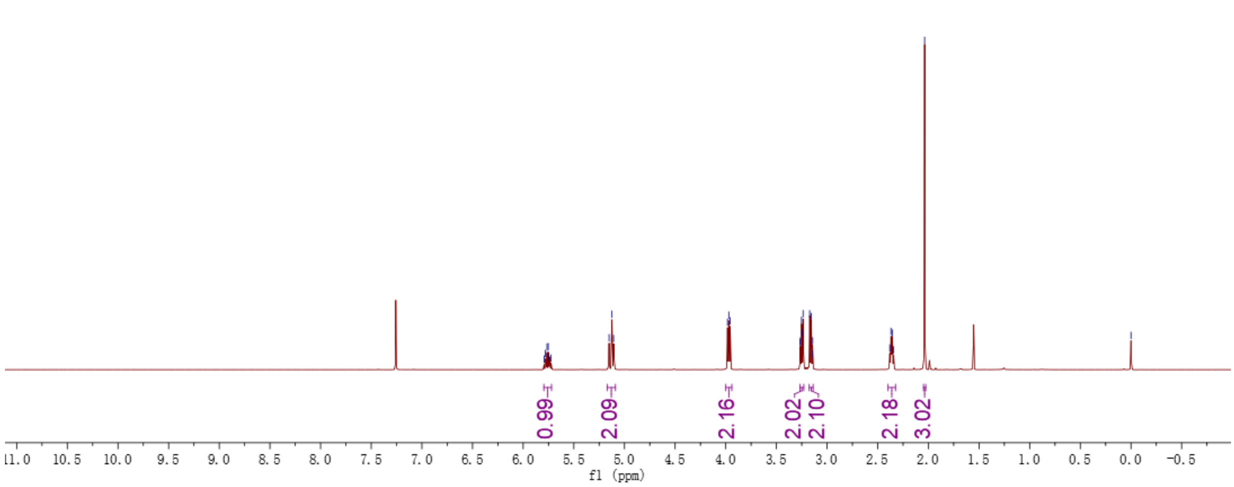

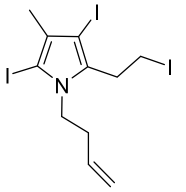

^{13}C NMR ($CDCl_3$, 100 MHz, TMS)

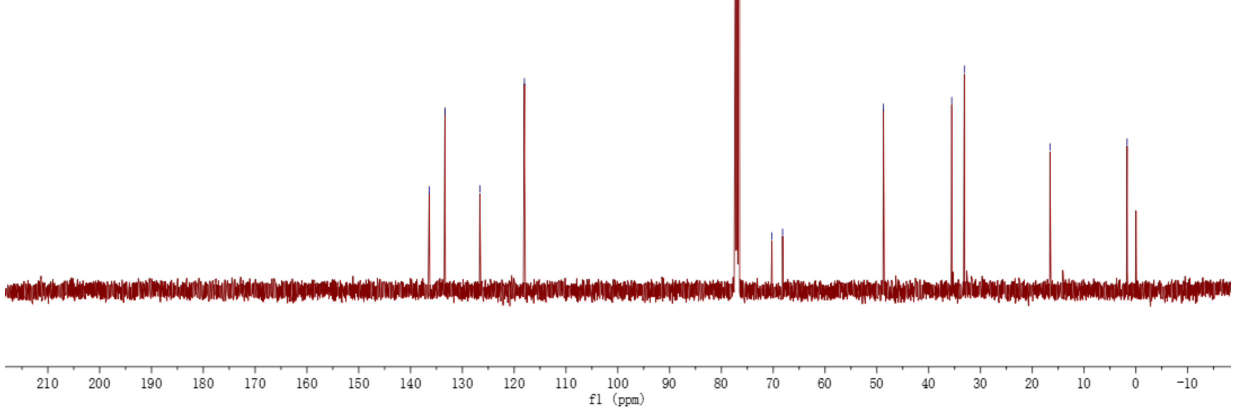

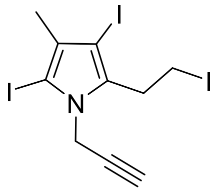

2,4-diiodo-5-(2-iodoethyl)-3-methyl-1-(prop-2-yn-1-yl)-1H-pyrrole (3ha)

A colorless oil, 44.6 mg, 85% yield. ^{1}H NMR ($CDCl_3$, TMS, 400 MHz) δ 4.75 (d, J = 2.5 Hz, 2H), 3.38 - 3.30 (m, 2H), 3.29 - 3.20 (m, 2H), 2.40 (t, *J* = 2.5 Hz, 1H), 2.05 (s, 3H). ^{13}C NMR ($CDCl_3$, TMS, 100 MHz) δ 136.7, 127.3, 77.8, 73.7, 70.8, 69.5, 39.2, 33.2, 16.7, 1.5. IR (Neat) ν 675, 734, 946, 1174, 1325, 1378, 1442, 2919, 3281 cm^{-1}. HRMS (EI) calcd. for $C_{10}H_{10}I_3N$: 524.7960, Found: 524.7942.

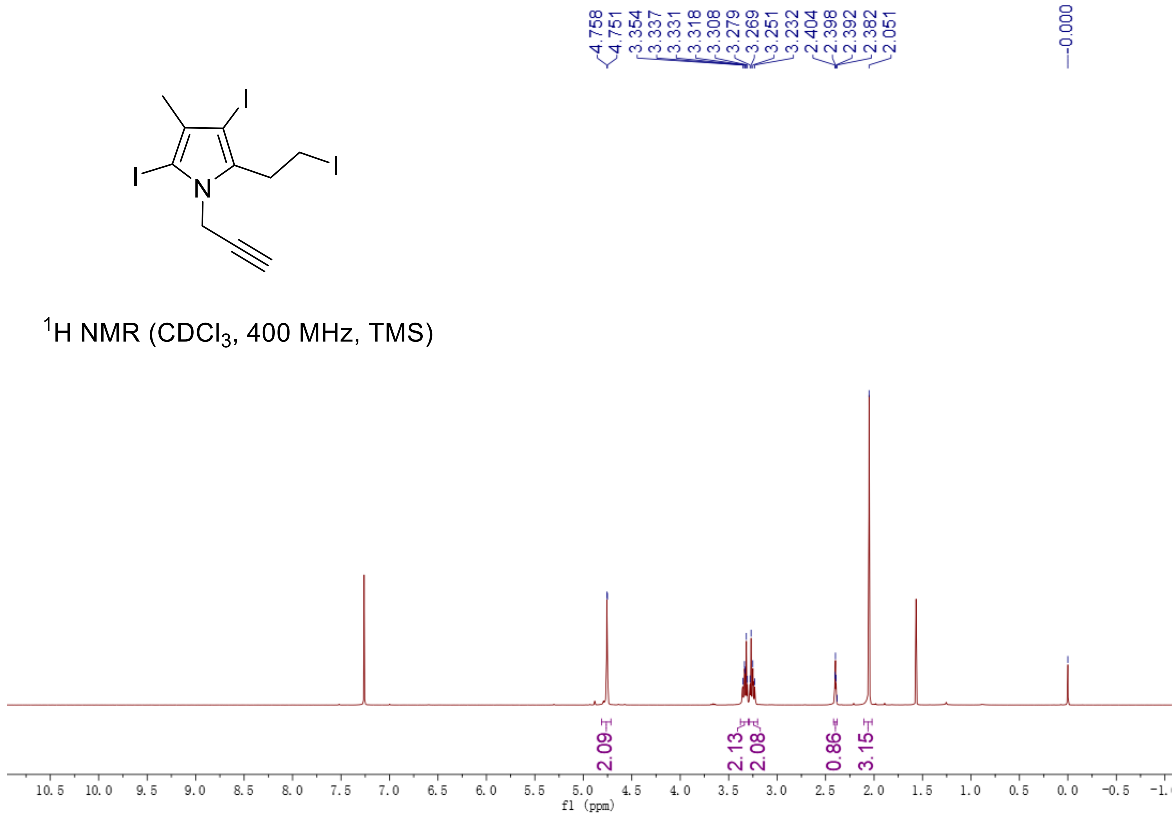

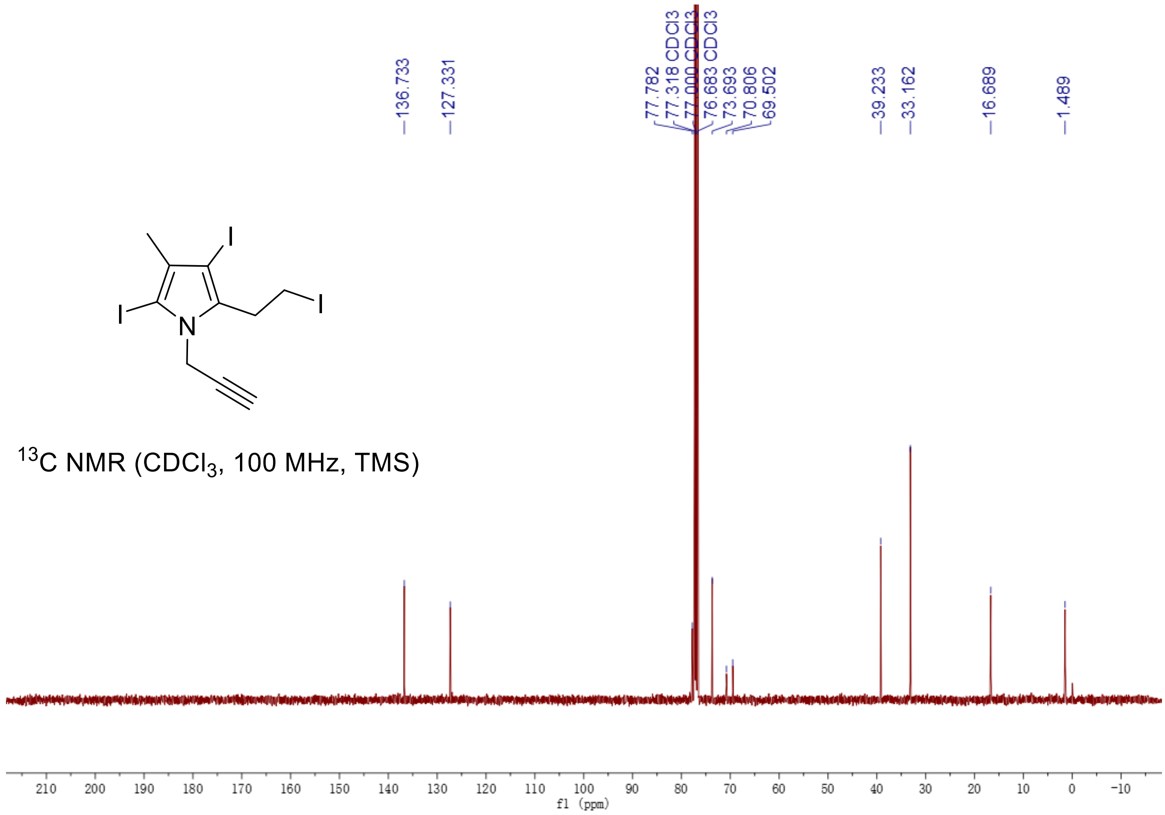

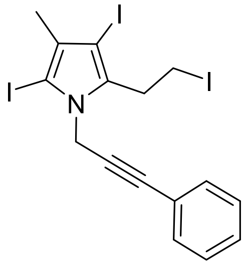

2,4-diiodo-5-(2-iodoethyl)-3-methyl-1-(3-phenylprop-2-yn-1-yl)-1H-pyrrole (3ia)

A colorless oil, 49.3 mg, 82% yield. ^{1}H NMR ($CDCl_3$, TMS, 600 MHz) δ 7.43 (d, J = 8.3 Hz, 2H), 7.36-7.26 (m, 3H), 4.96 (s, 2H), 3.44 - 3.36 (m, 2H), 3.35 - 3.27 (m, 2H), 2.06 (s, 3H). ^{13}C NMR ($CDCl_3$, TMS, 150 MHz) δ 136.8, 131.9, 128.8, 128.3, 127.1, 121.9, 85.3, 83.0, 70.9, 69.3, 40.1, 33.3, 16.7, 1.6. IR (Neat) ν 696, 735, 792, 1028, 1172, 1261, 1327, 1380, 1490, 2922 cm^{-1}. HRMS (ESI) calcd. for $C_{16}H_{15}I_3N$ (M+H): 601.8339, Found: 601.8547.

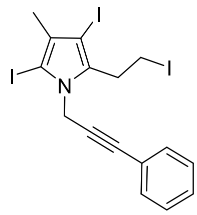

^1H NMR ($CDCl_3$, 600 MHz, TMS)

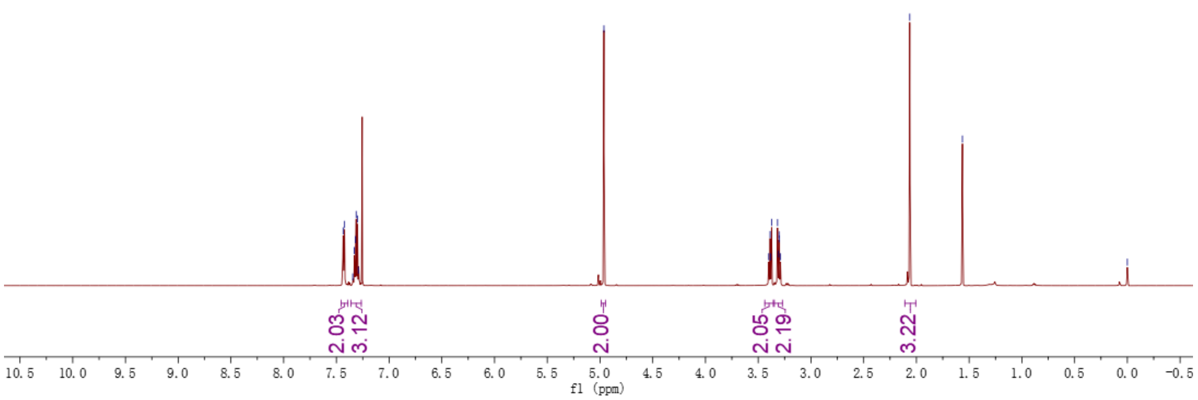

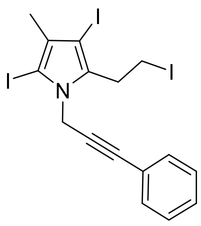

^{13}C NMR ($CDCl_3$, 150 MHz, TMS)

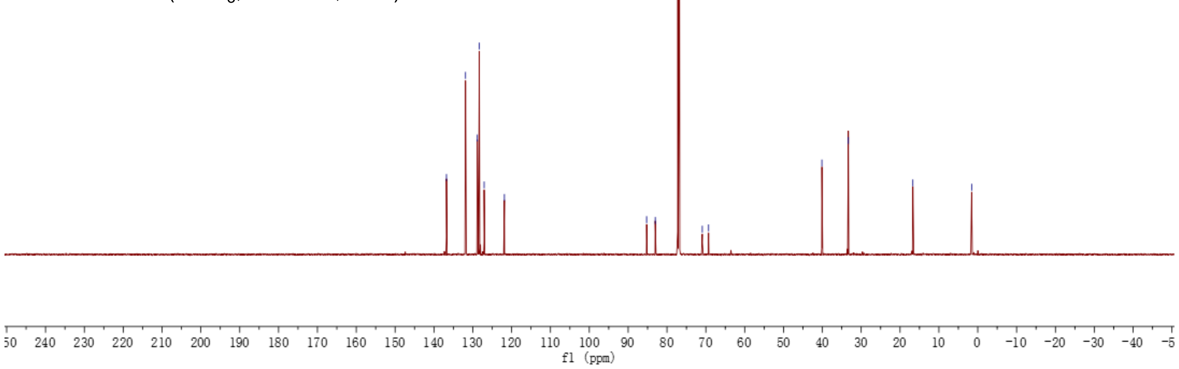

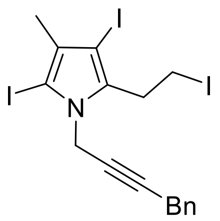

2,4-diiodo-5-(2-iodoethyl)-3-methyl-1-(4-phenylbut-2-yn-1-yl)-1H-pyrrole (3ja)

A colorless oil, 51.7 mg, 84% yield. ^{1}H NMR ($CDCl_3$, TMS, 600 MHz) δ 7.36 - 7.28 (m, 4H), 7.27 - 7.22 (m, 1H), 4.78 (t, *J* = 2.3 Hz, 2H), 3.60 - 3.58 (m, 2H), 3.39 - 3.31 (m, 2H), 3.30 - 3.21 (m, 2H), 2.06 (s, 3H). ^{13}C NMR ($CDCl_3$, TMS, 150 MHz) δ 136.7, 135.9, 128.6, 127.9, 127.0, 126.8, 83.6, 76.3, 70.8, 69.1, 39.8, 33.3, 25.1, 16.7, 1.6. IR (Neat) ν 703, 814, 926, 1062, 1169, 1362, 1723, 2951 cm^{-1}. HRMS (ESI) calcd. for $C_{17}H_{17}I_3N$ (M+H): 615.8456, Found: 615.9012.

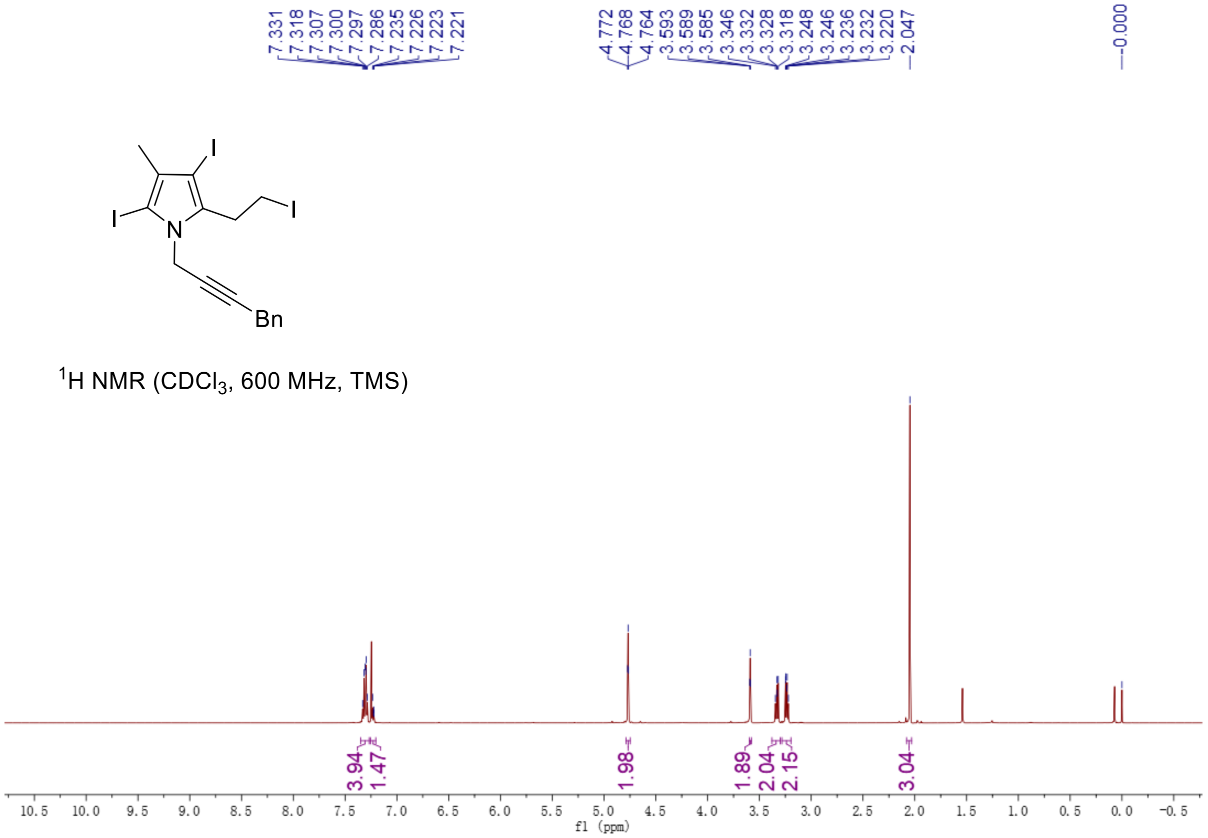

^{1}H NMR ($CDCl_3$, 600 MHz, TMS)

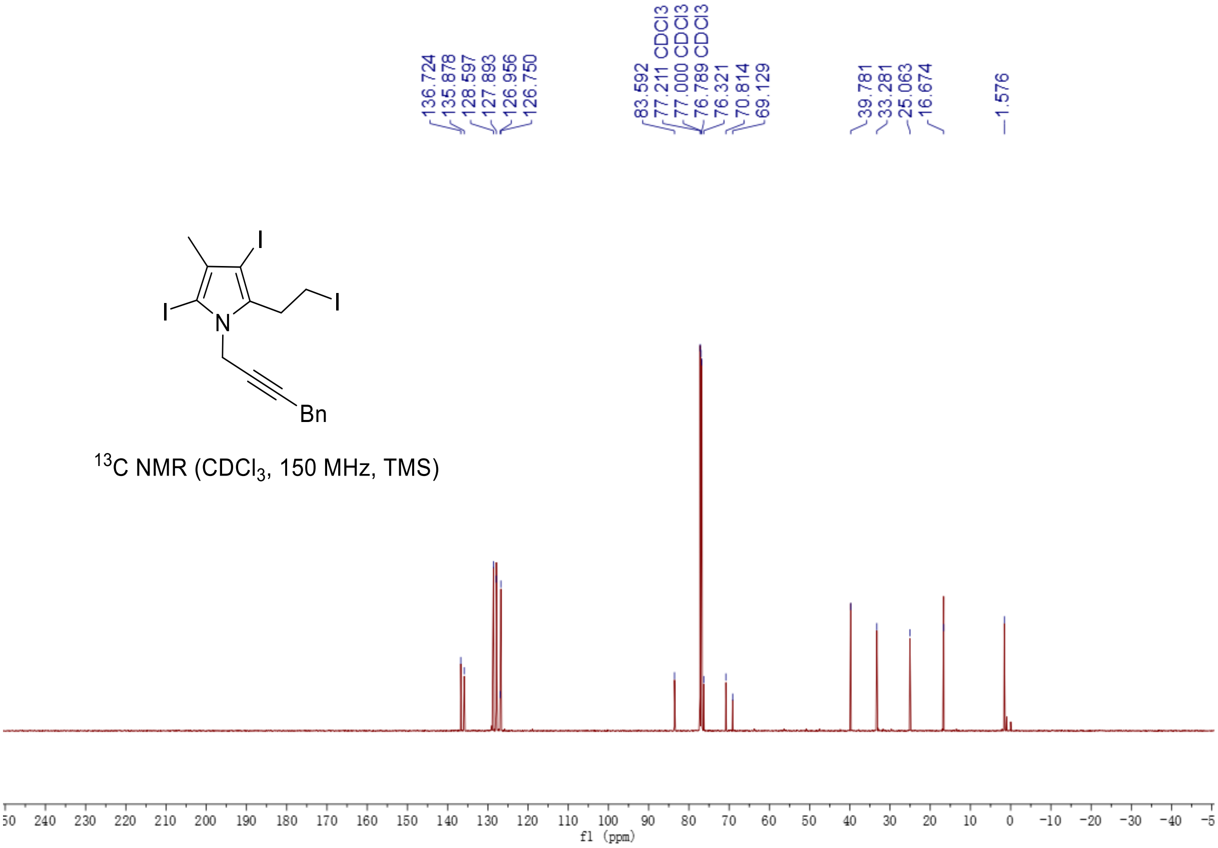

^{13}C NMR ($CDCl_3$, 150 MHz, TMS)

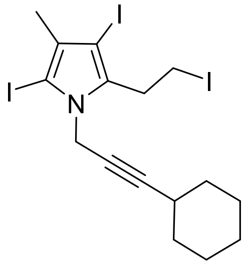

1-(3-cyclohexylprop-2-yn-1-yl)-2,4-diiodo-5-(2-iodoethyl)-3-methyl-1H-pyrrole (3ka)

A colorless oil, 50.4 mg, 83% yield. ^{1}H NMR ($CDCl_3$, TMS, 400 MHz) δ 4.71 (d, *J* = 2.1 Hz, 2H), 3.37 - 3.21 (m, 4H), 2.39 - 2.29 (m, 1H), 2.04 (s, 3H), 1.83 - 1.72 (m, 2H), 1.73 - 1.61 (m, 2H), 1.55 - 1.47 (m, 1H), 1.45 - 1.34 (m, 2H), 1.33 - 1.22 (m, 3H). ^{13}C NMR ($CDCl_3$, TMS, 100 MHz) δ 136.7, 126.7, 90.2, 74.1, 70.8, 68.9, 39.8, 33.3, 32.3, 29.0, 25.7, 24.8, 16.7, 1.7. IR (Neat) ν 721, 1173, 1326, 1442, 2213, 2849, 2919, 2934 cm^{-1}. HRMS (EI) calcd. for $C_{16}H_{20}I_3N$: 606.8750, Found: 606.8724.

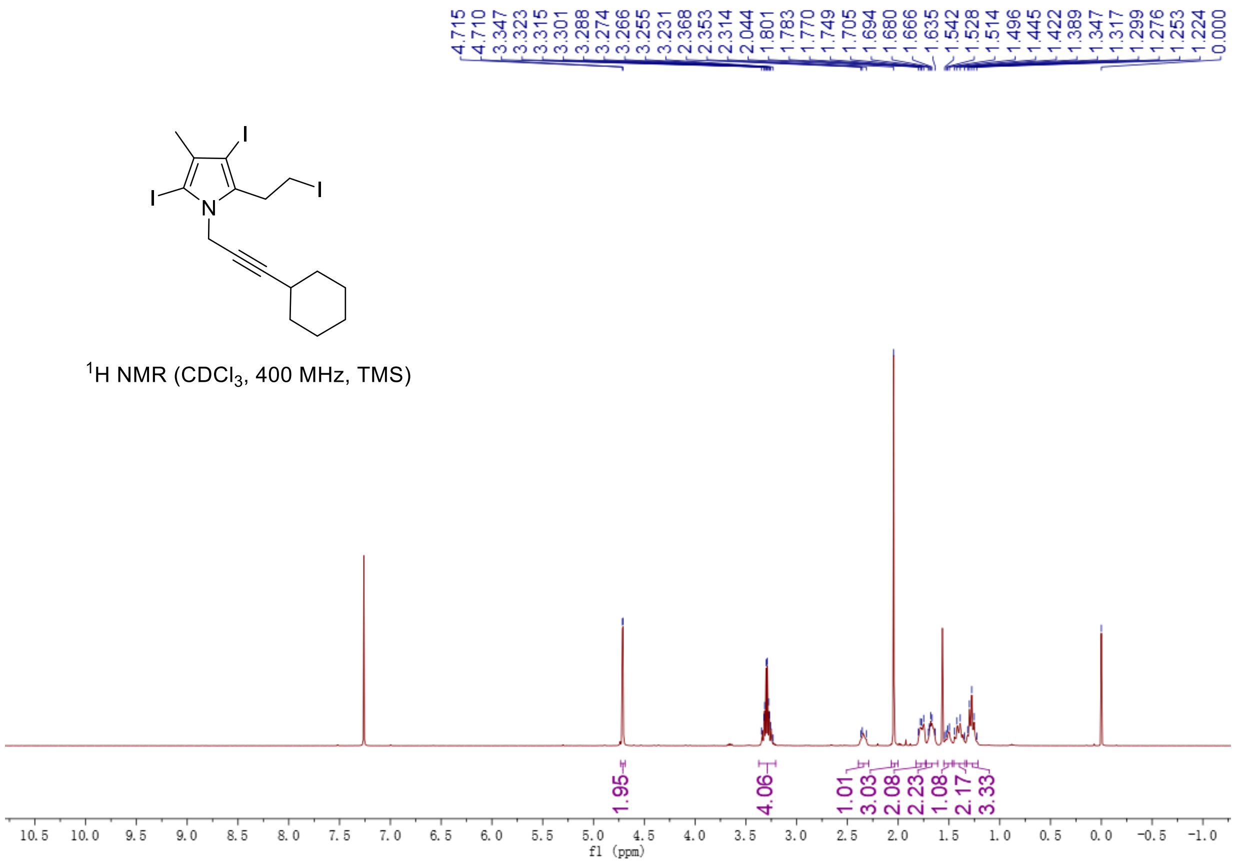

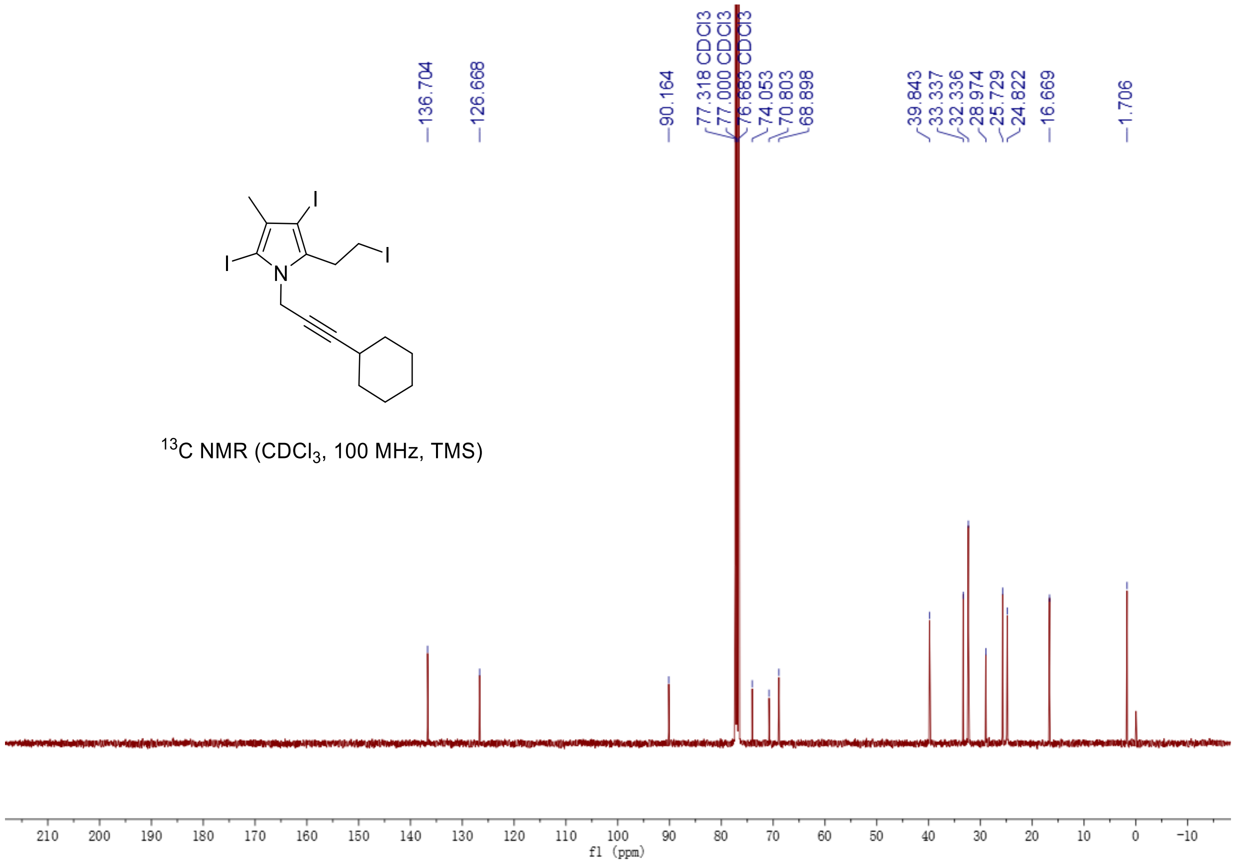

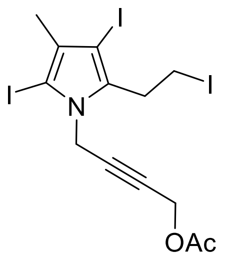

4-(2,4-diiodo-5-(2-iodoethyl)-3-methyl-1H-pyrrol-1-yl)but-2-yn-1-yl acetate (3la)

A colorless oil, 54.9 mg, 92% yield. ^{1}H NMR ($CDCl_3$, TMS, 400 MHz) δ 4.79 (t, *J* = 2.0 Hz, 2H), 4.66 (t, *J* = 1.9 Hz, 2H), 3.35 - 3.29 (m, 2H), 3.27 - 3.21 (m, 2H), 2.11 (s, 3H), 2.05 (s, 3H). ^{13}C NMR ($CDCl_3$, TMS, 100 MHz) δ 170.1, 136.7, 127.2, 80.6, 79.4, 70.8, 69.4, 52.0, 39.4, 33.1, 20.8, 16.7, 1.5. IR (Neat) ν 734, 1031, 1171, 1216, 1240, 1327, 1379, 1422, 1729 1742, 2917 cm^{-1}. HRMS (ESI) calcd. for $C_{13}H_{15}NO_2I_3$ (M+H): 597.8231, Found: 597.8246.

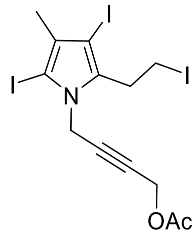

^{1}H NMR ($CDCl_3$, 400 MHz, TMS)

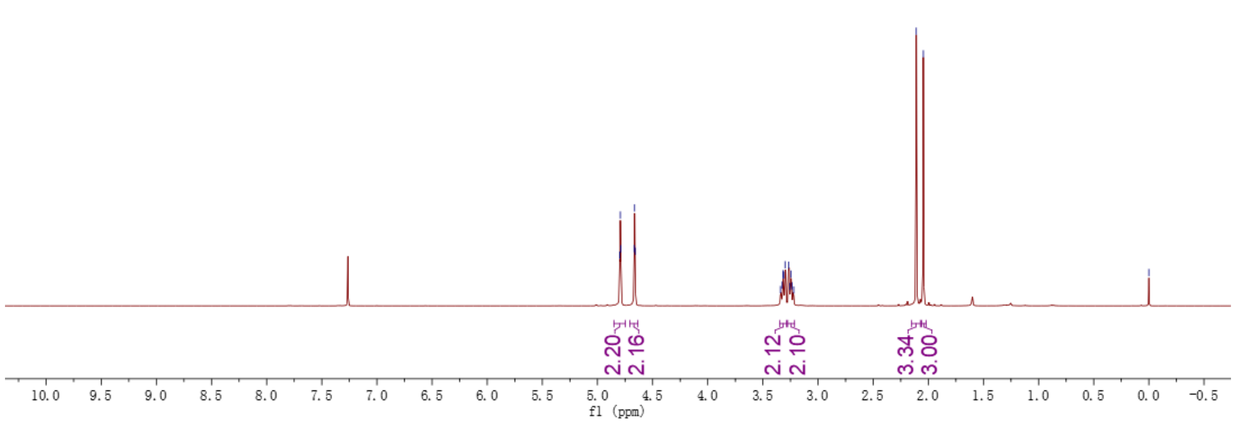

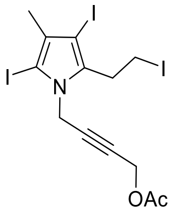

^{13}C NMR ($CDCl_3$, 100 MHz, TMS)

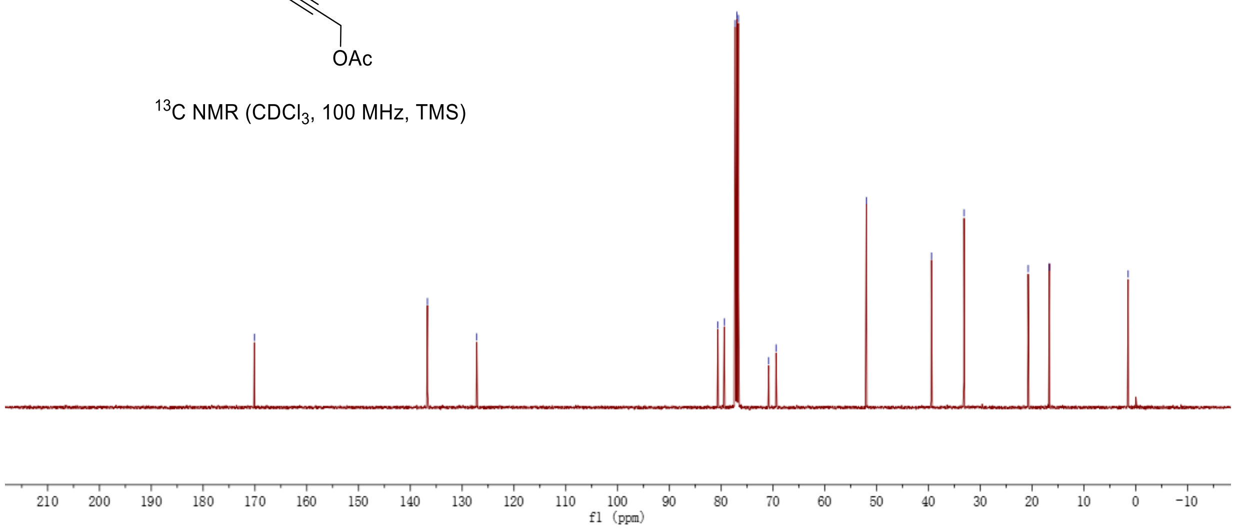

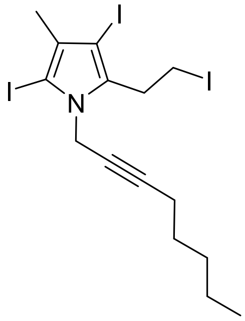

2,4-diiodo-5-(2-iodoethyl)-3-methyl-1-(oct-2-yn-1-yl)-1H-pyrrole (3ma)

A colorless oil, 50.6 mg, 85% yield. ^{1}H NMR ($CDCl_3$, TMS, 600 MHz) δ 4.70 (t, J = 2.3 Hz, 2H), 3.34 - 3.29 (m, 2H), 3.29 - 3.24 (m, 2H), 2.18 - 2.14 (m, 2H), 2.04 (s, 3H), 1.53 - 1.45 (m, 2H), 1.36 - 1.28 (m, 4H), 0.89 (t, J = 7.1 Hz, 3H). ^{13}C NMR ($CDCl_3$, TMS, 150 MHz) δ 136.7, 126.8, 86.3, 74.1, 70.8, 69.0, 39.8, 33.3, 31.0, 28.0, 22.2, 18.7, 16.7, 14.0, 1.6. IR (Neat) ν 716, 896, 965, 1020, 1066, 1104, 1180, 1325, 1406, 1445, 2920 cm^{-1}. HRMS (ESI) calcd. for $C_{15}H_{21}I_3N$ (M+H): 595.8739, Found: 595.8846.

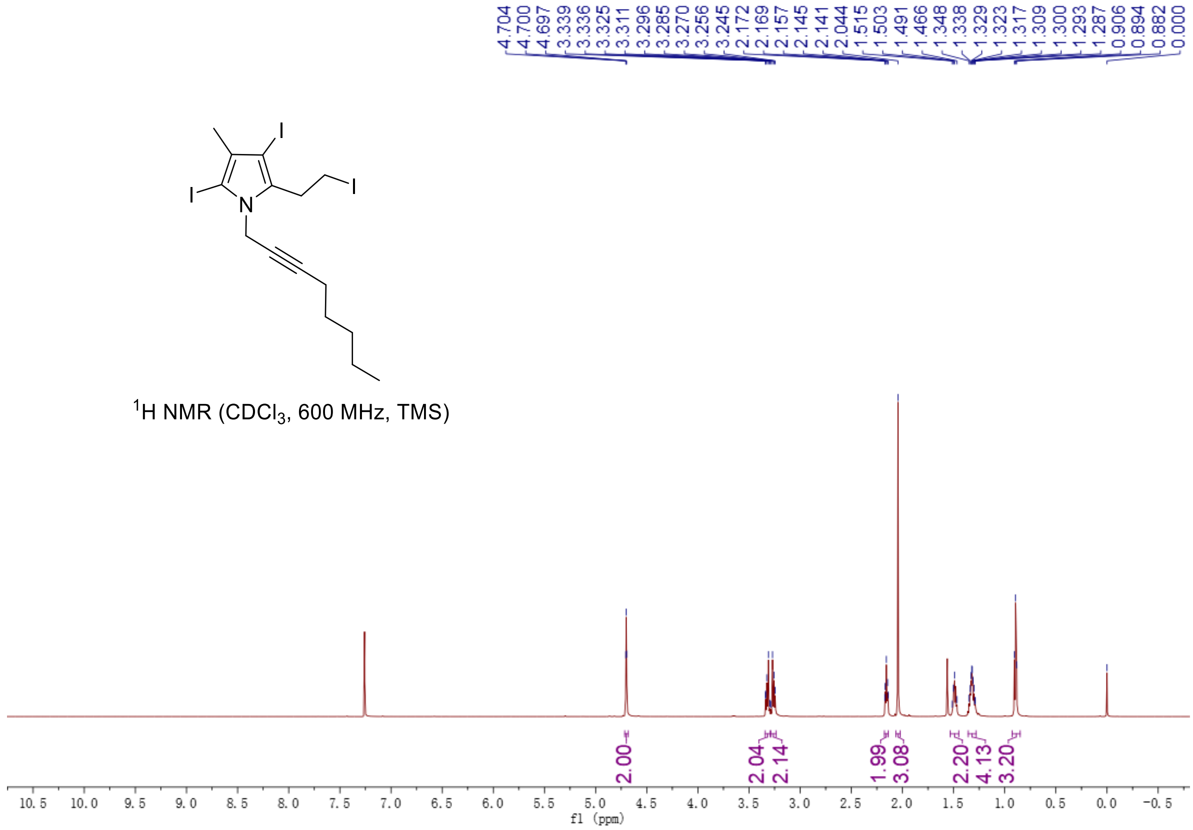

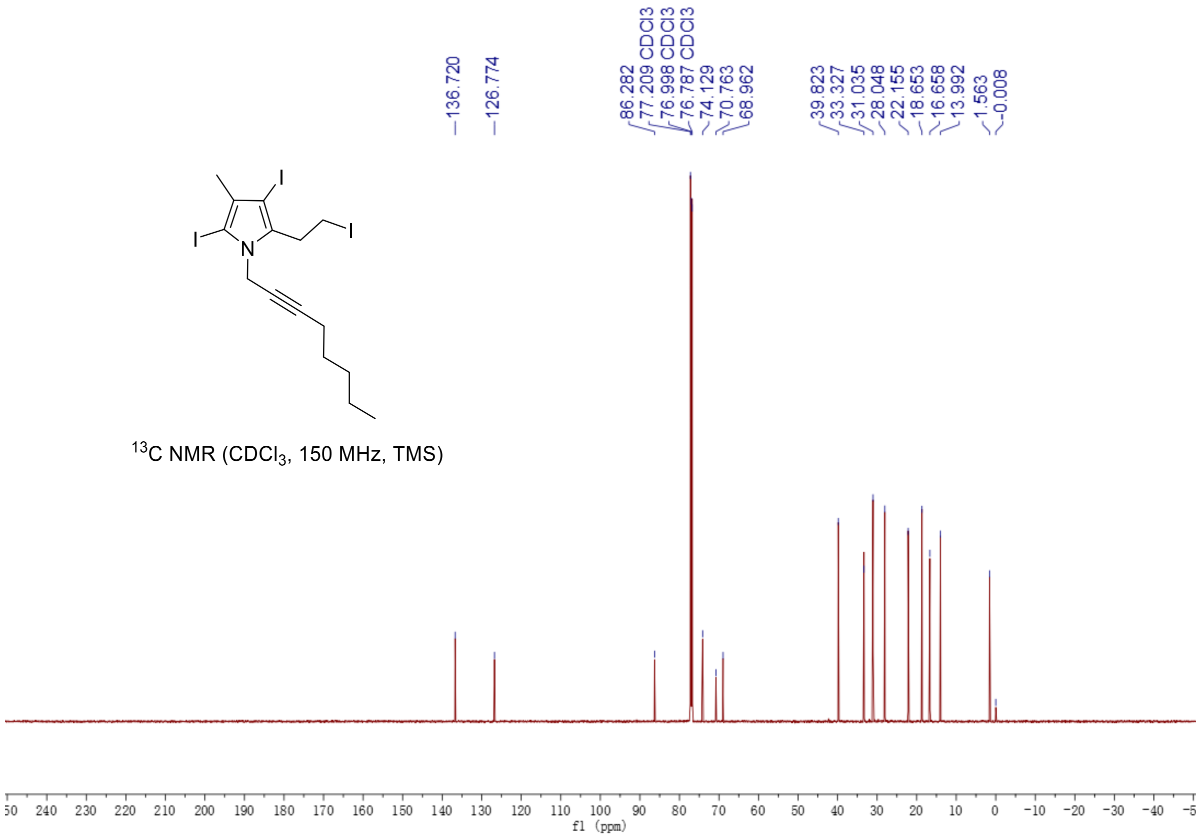

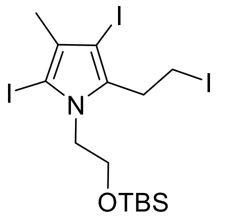

1-(2-((tert-butyldimethylsilyl)oxy)ethyl)-2,4-diiodo-5-(2-iodoethyl)-3-methyl-1H-pyrrole (3na)

A colorless oil, 48.4 mg, 75% yield. ^{1}H NMR ($CDCl_3$, TMS, 400 MHz) δ 4.06 (t, *J* = 5.8 Hz, 2H), 3.75 (t, *J* = 5.8 Hz, 2H), 3.38 - 3.29 (m, 2H), 3.20 - 3.11 (m, 2H), 2.03 (s, 3H), 0.84 (s, 9H), -0.05 (s, 6H). ^{13}C NMR ($CDCl_3$, TMS, 100 MHz) δ 137.8, 126.7, 69.8, 68.1, 62.6, 51.0, 33.0, 25.8, 18.3, 16.6, 1.9, -5.6. IR (Acetone) ν 725, 1027, 1171, 1183, 1306, 1329, 1439, 1705, 2927 cm^{-1}. HRMS (ESI) calcd. for $C_{15}H_{27}NOI_3Si$ (M+H): 645.8997, Found: 645.8996.

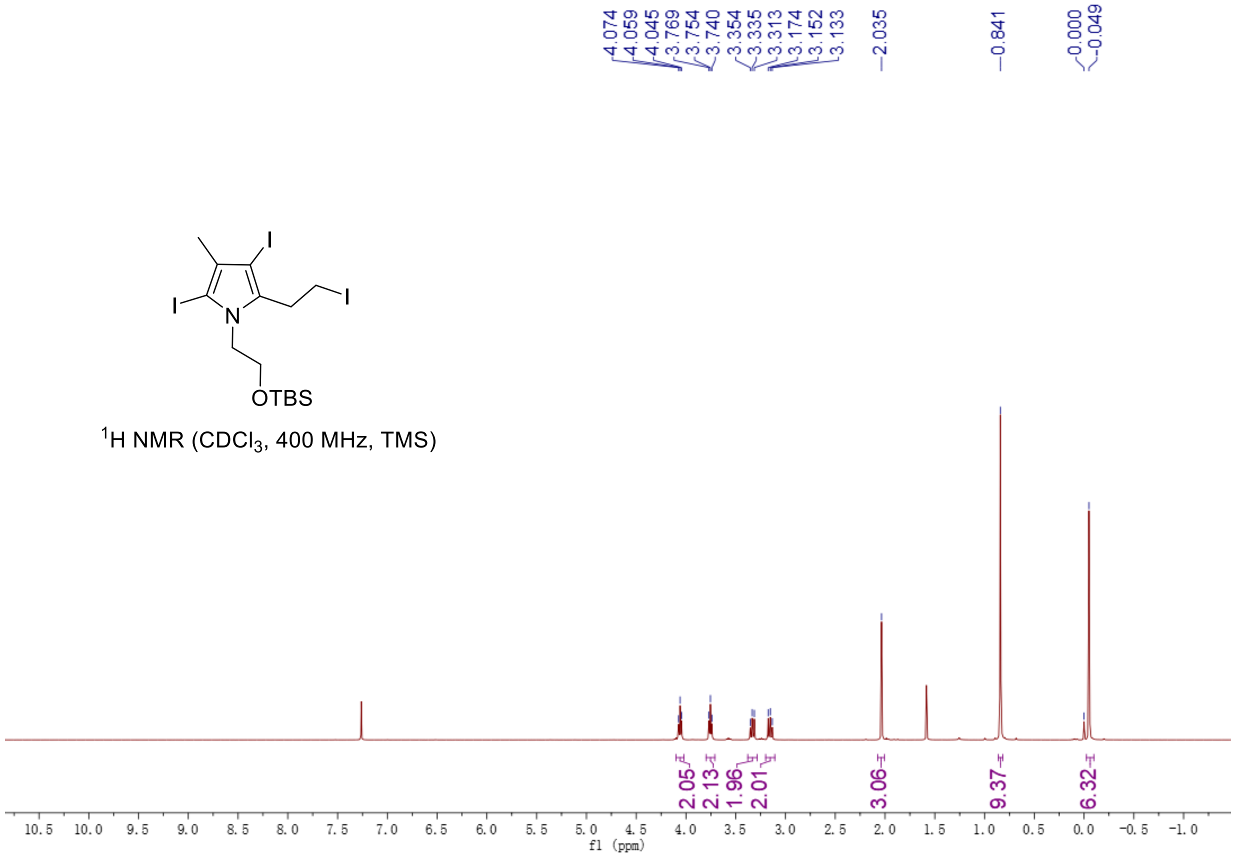
4.074
4.059
4.045
3.769
3.754
3.740
3.354
3.335
3.313
3.174
3.152
3.133
2.035
0.841
0.000
-0.049
OTBS
N
I
^{1}H NMR (CDCl$_3$, 400 MHz, TMS)
2.05
2.13
1.96
2.01
3.06
9.37
6.32
f1 (ppm)

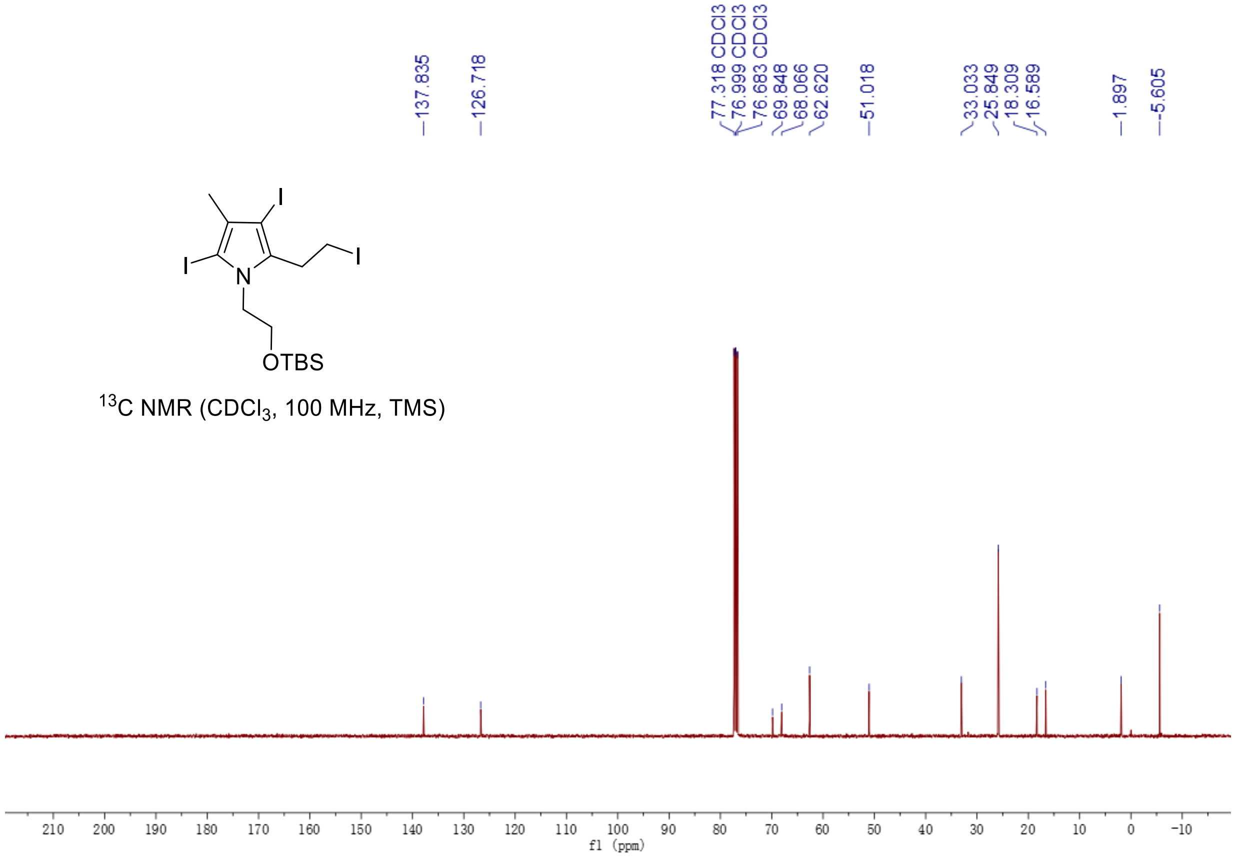
137.835
126.718
77.318 CDCl3
76.999 CDCl3
76.683 CDCl3
69.848
68.066
62.620
51.018
33.033
25.849
18.309
16.589
1.897
-5.605
OTBS
N
I
^{13}C NMR (CDCl$_3$, 100 MHz, TMS)
f1 (ppm)

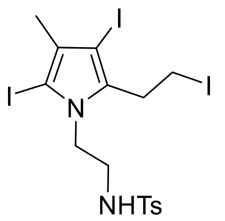

N-(2-(2,4-diiodo-5-(2-iodoethyl)-3-methyl-1H-pyrrol-1-yl)ethyl)-4-methylbenzenesulfonamide (3oa)

A colorless oil, 36.3 mg, 53% yield. ^{1}H NMR ($CDCl_3$, TMS, 400 MHz) δ 7.71 (d, *J* = 8.0 Hz, 2H), 7.31 (d, *J* = 8.0 Hz, 2H), 4.60 (t, *J* = 6.6 Hz, 1H), 4.11 (t, *J* = 6.8 Hz, 2H), 3.31 - 3.22 (m, 2H), 3.19 - 3.12 (m, 4H), 2.44 (s, 3H), 1.99 (s, 3H). ^{13}C NMR ($CDCl_3$, TMS, 100 MHz) δ 143.9, 136.9, 136.5, 129.9, 127.4, 127.1, 69.9, 69.1, 48.7, 43.2, 32.5, 21.6, 16.6, 1.7. IR (Neat) ν 758, 806, 1027, 1090, 1146, 1326, 1433, 1462, 1603, 3300 cm^{-1}. HRMS (ESI) calcd. for $C_{16}H_{19}N_2O_2NaSI_3$ (M+Na): 706.8194, Found: 706.8189.

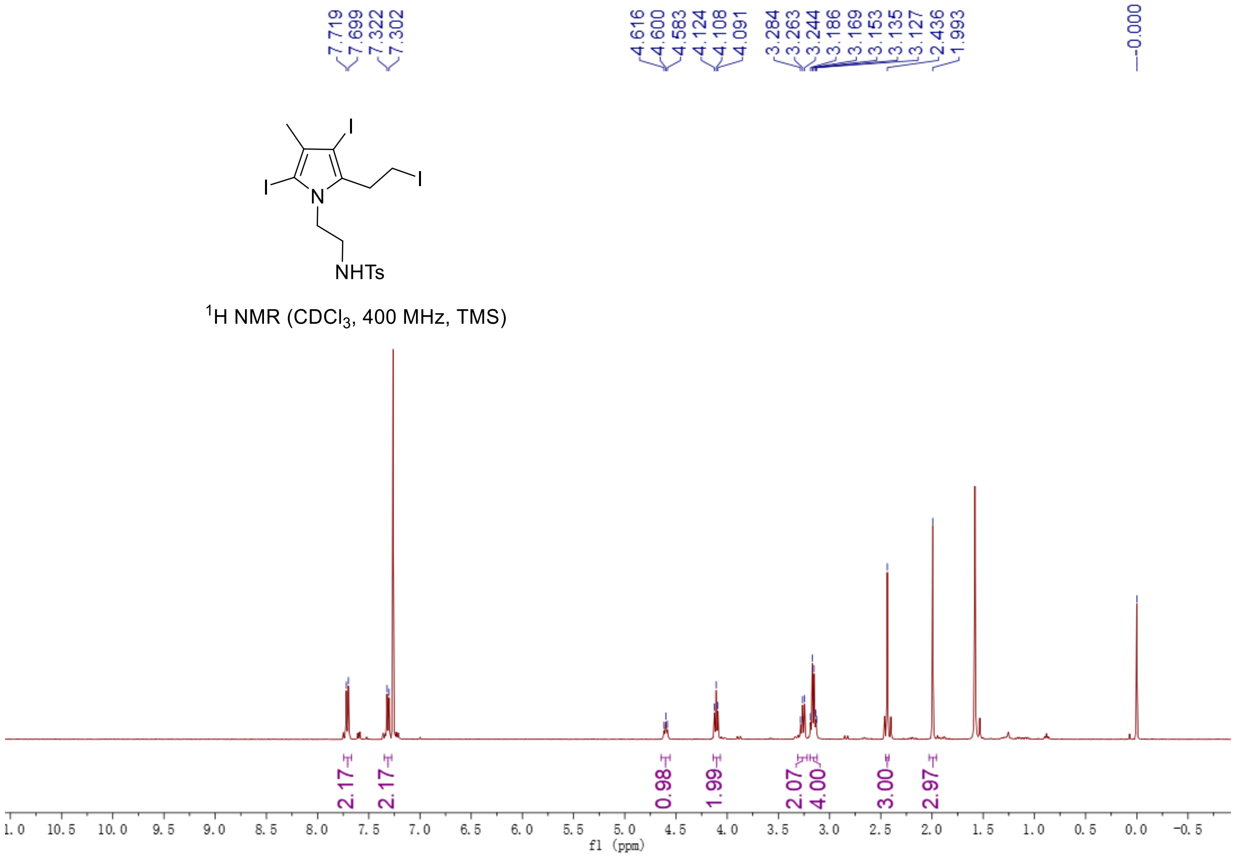

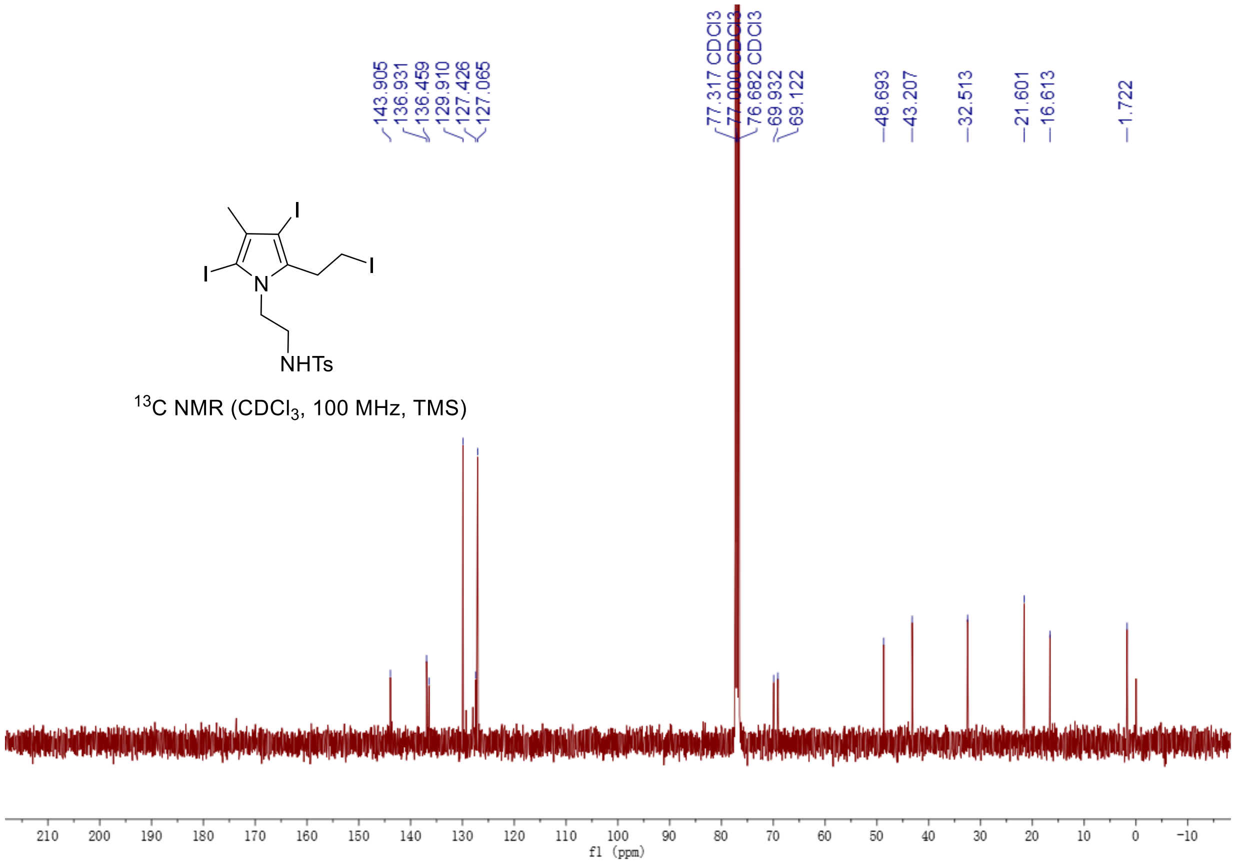

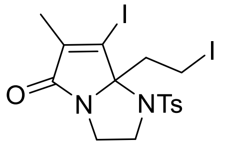

7-iodo-7a-(2-iodoethyl)-6-methyl-1-tosyl-1,2,3,7a-tetrahydro-*5H*-pyrrolo[1,2-*a*]imidazol-5-one (3oa’)

A colorless oil, 25.7 mg, 45% yield. ^{1}H NMR ($CDCl_3$, TMS, 400 MHz) δ 7.71 (d, J = 8.4 Hz, 2H), 7.31 (d, J = 8.0 Hz, 2H), 4.06 - 4.01 (m, 1H), 3.71 - 3.66 (m, 1H), 3.41 - 3.33 (m, 1H), 3.28 - 3.21 (m, 1H), 3.17 - 3.09 (m, 1H), 2.95 - 2.81 (m, 2H), 2.67 - 2.56 (m, 1H), 2.43 (s, 3H), 1.88 (s, 3H). ^{13}C NMR ($CDCl_3$, TMS, 100 MHz) δ 173.4, 163.0, 146.1, 144.3, 136.8, 129.8, 127.4, 115.9, 90.1, 77.3, 41.4, 21.6, 14.5, -5.7. IR (Neat) ν 2957, 1610, 1553, 1329, 1240, 809, 773 cm^{-1}. HRMS (ESI) calcd. for $C_{16}H_{19}N_2O_3NaSI_2$ (M+H): 594.9197, Found: 594.9200.

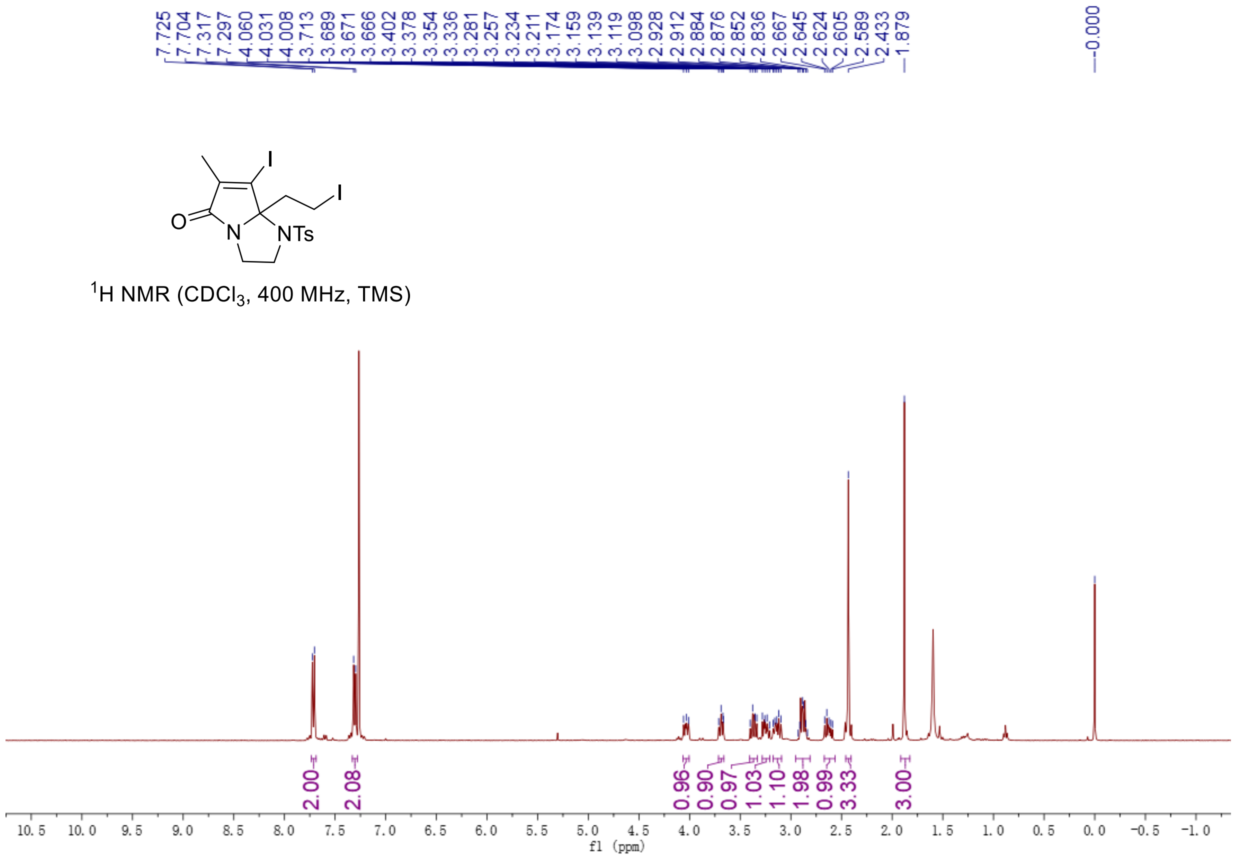

^{1}H NMR ($CDCl_3$, 400 MHz, TMS)

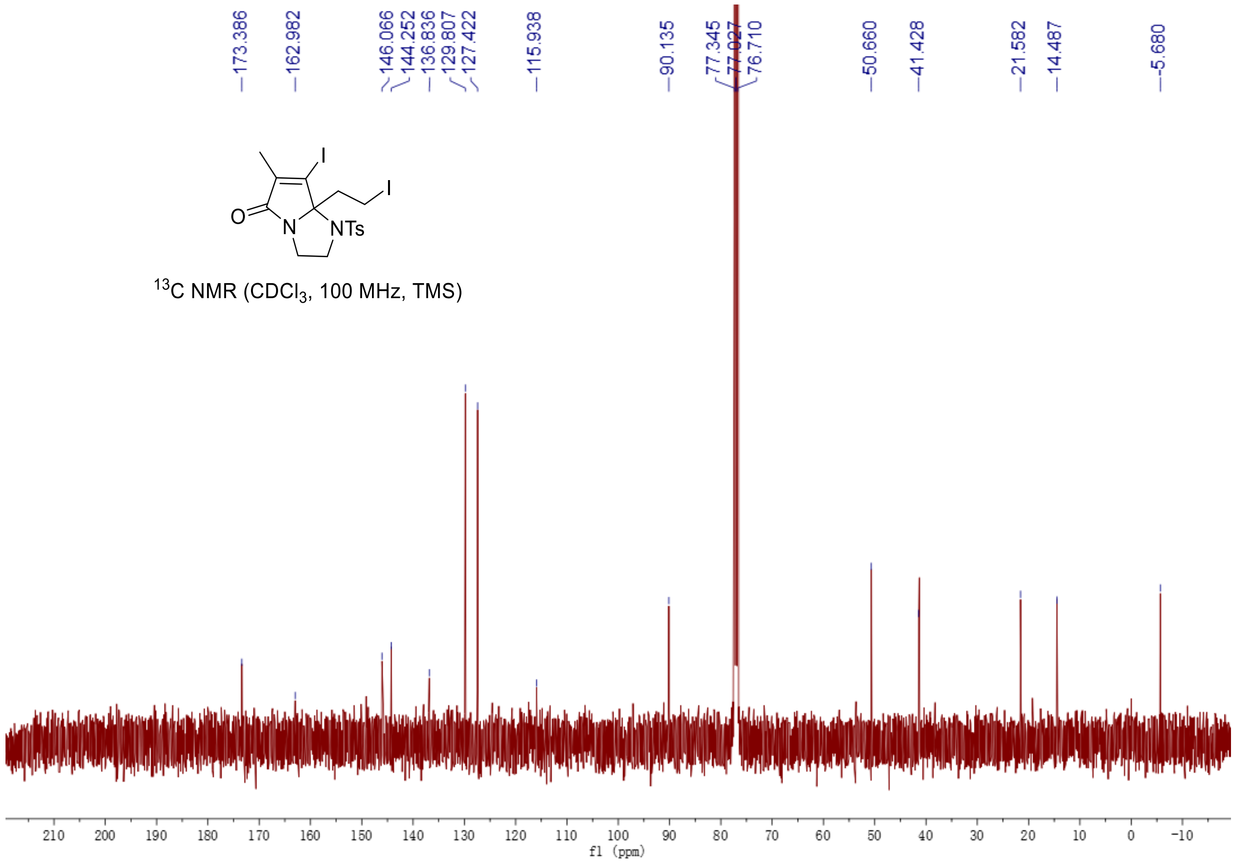

^{13}C NMR ($CDCl_3$, 100 MHz, TMS)

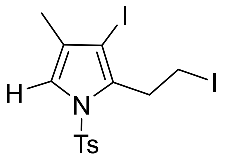

2,4-diiodo-5-(2-iodoethyl)-3-methyl-1-tosyl-1H-pyrrole (3pa)

A colorless oil, 38.5 mg, 60% yield. ^{1}H NMR ($CDCl_3$, TMS, 600 MHz) δ 7.67 (d, *J* = 8.4 Hz, 2H), 7.33 (d, *J* = 8.0 Hz, 2H), 7.17 (s, 1H), 3.31 - 3.23 (m, 2H), 3.16 - 3.06 (m, 2H), 2.43 (s, 3H), 1.97 (s, 3H). ^{13}C NMR ($CDCl_3$, TMS, 150 MHz) δ 145.4, 135.7, 134.2, 130.3, 126.8, 125.0, 119.2, 80.2, 33.1, 21.7, 13.7, 1.2. IR (Acetone) ν 735, 792, 1172, 1327, 1380, 2207, 2922 cm^{-1}. HRMS (ESI) calcd. for $C_{14}H_{15}NO_2NaSI_2$ (M+Na): 537.8913, Found: 537.8903.

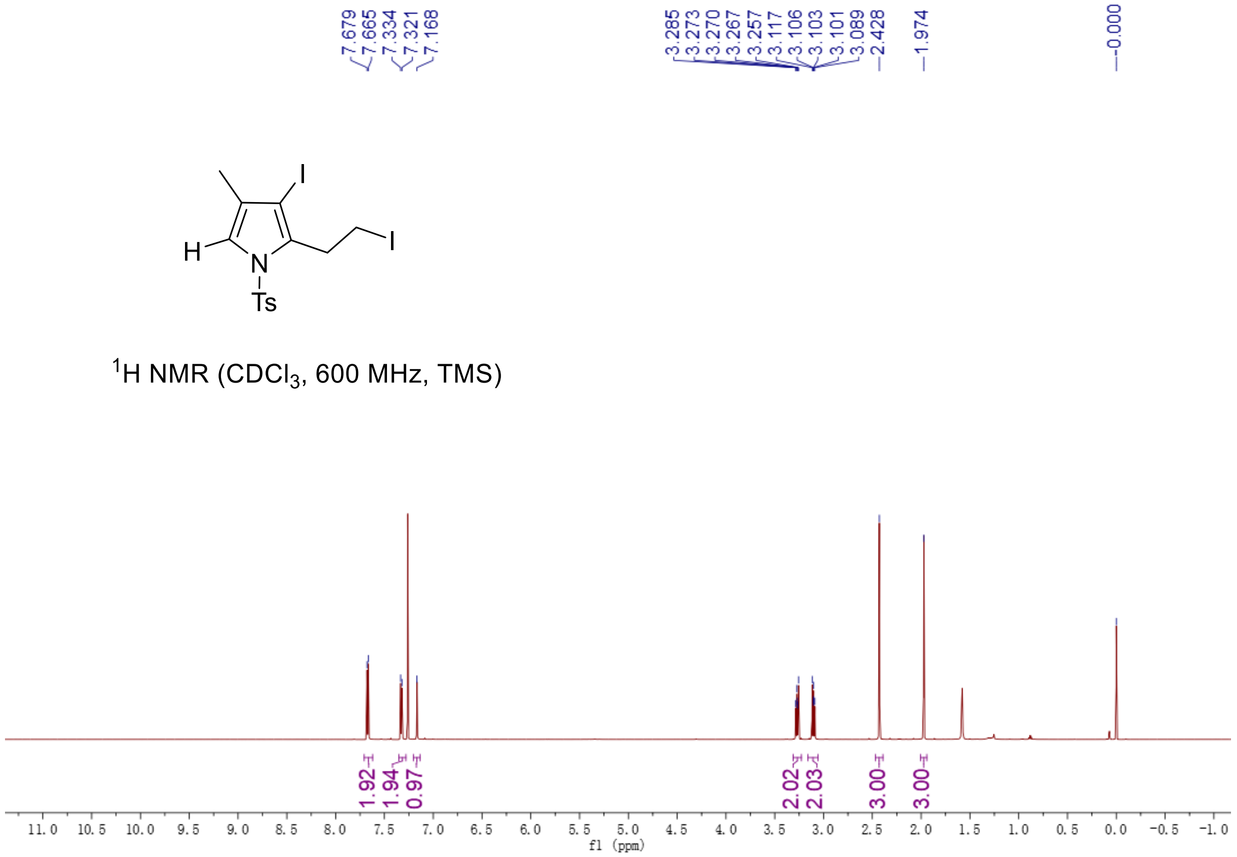

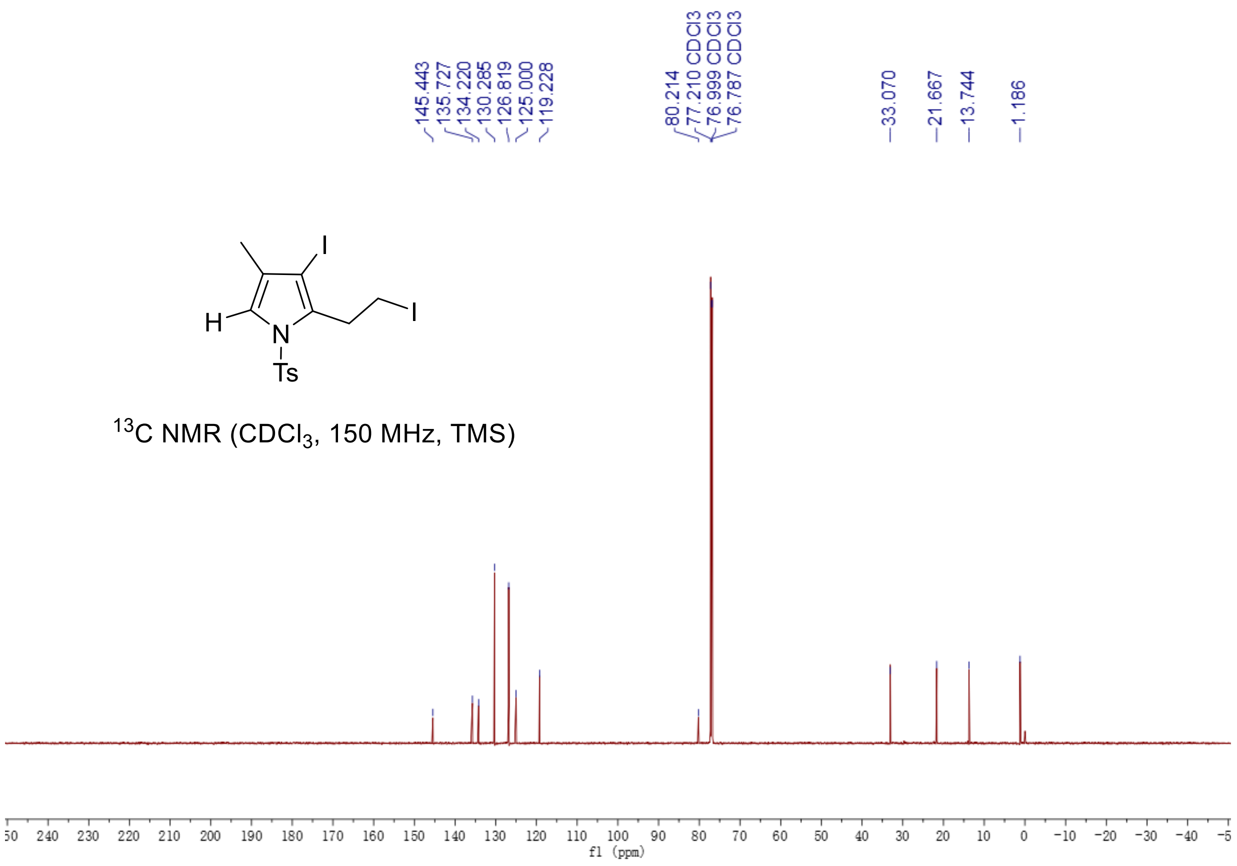

145.443
135.727
134.220
130.285
126.819
125.000
119.228
80.214
77.210 CDCl3
76.999 CDCl3
76.787 CDCl3
33.070
21.667
13.744
1.186
I
H
N
I
Ts
13C NMR (CDCl3, 150 MHz, TMS)
f1 (ppm)

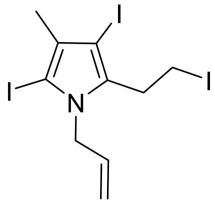

1-allyl-2,4-diiodo-5-(2-iodoethyl)-3-methyl-1H-pyrrole (3ra)

A colorless oil, 49.0 mg, 93% yield. ^{1}H NMR ($CDCl_3$, TMS, 600 MHz) δ 5.89 - 5.79 (m, 1H), 5.19 (d, *J* = 10.5 Hz, 1H), 4.81 (d, *J* = 17.1 Hz, 1H), 4.60 - 4.57 (m, 2H), 3.23 - 3.19 (m, 2H), 3.16 - 3.12 (m, 2H), 2.06 (s, 3H). ^{13}C NMR ($CDCl_3$, TMS, 150 MHz) δ 136.8, 133.2, 126.6, 117.0, 71.1, 68.5, 51.4, 33.1, 16.7, 1.9. IR (Neat) ν 927, 989, 1165, 1260, 1323, 1446, 2961 cm^{-1}. HRMS (EI) calcd. for $C_{10}H_{12}NI_3$: 526.8111, Found: 526.8098.

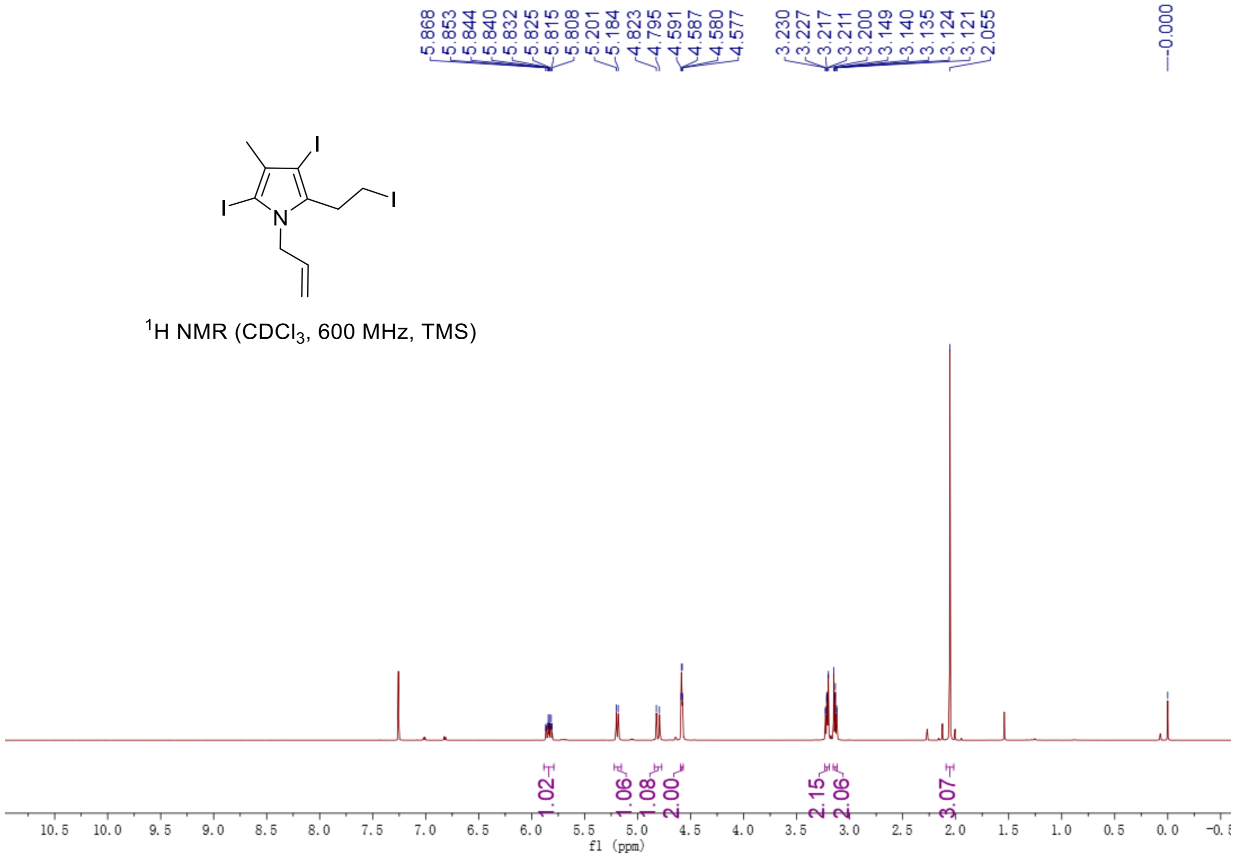

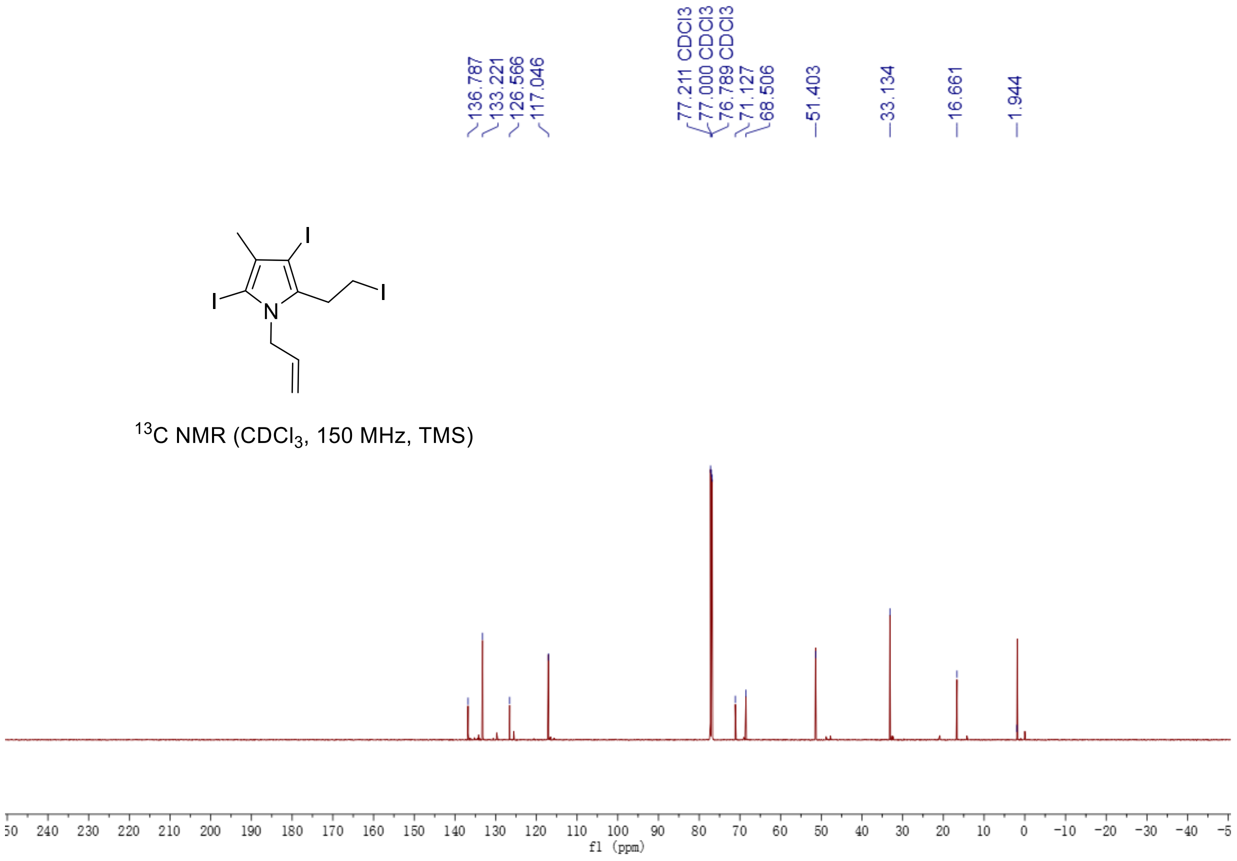

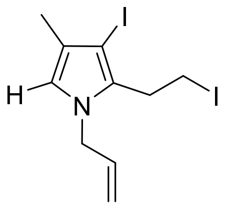

1-allyl-2,4-diiodo-5-(2-iodoethyl)-3-methyl-1H-pyrrole (3ra')

A colorless oil, 49.0 mg, 38% yield. ^{1}H NMR ($CDCl_3$, TMS, 400 MHz) δ 6.49 (s, 1H), 5.94 - 5.85 (m, 1H), 5.20 (d, *J* = 10.3 Hz, 1H), 5.01 (d, *J* = 17.1 Hz, 1H), 4.48 - 4.43 (m, 2H), 3.20 - 3.12 (m, 4H), 1.99 (s, 3H). ^{13}C NMR ($CDCl_3$, TMS, 100 MHz) δ 134.1, 132.5, 119.1, 117.3, 110.0, 69.0, 50.2, 31.6, 13.5, 2.4. IR (Neat) ν 2966, 1450, 1337, 1168, 1089, 989 cm^{-1}. HRMS (EI) calcd. for $C_{10}H_{13}NI_2$: 400.9137, Found: 400.9132.

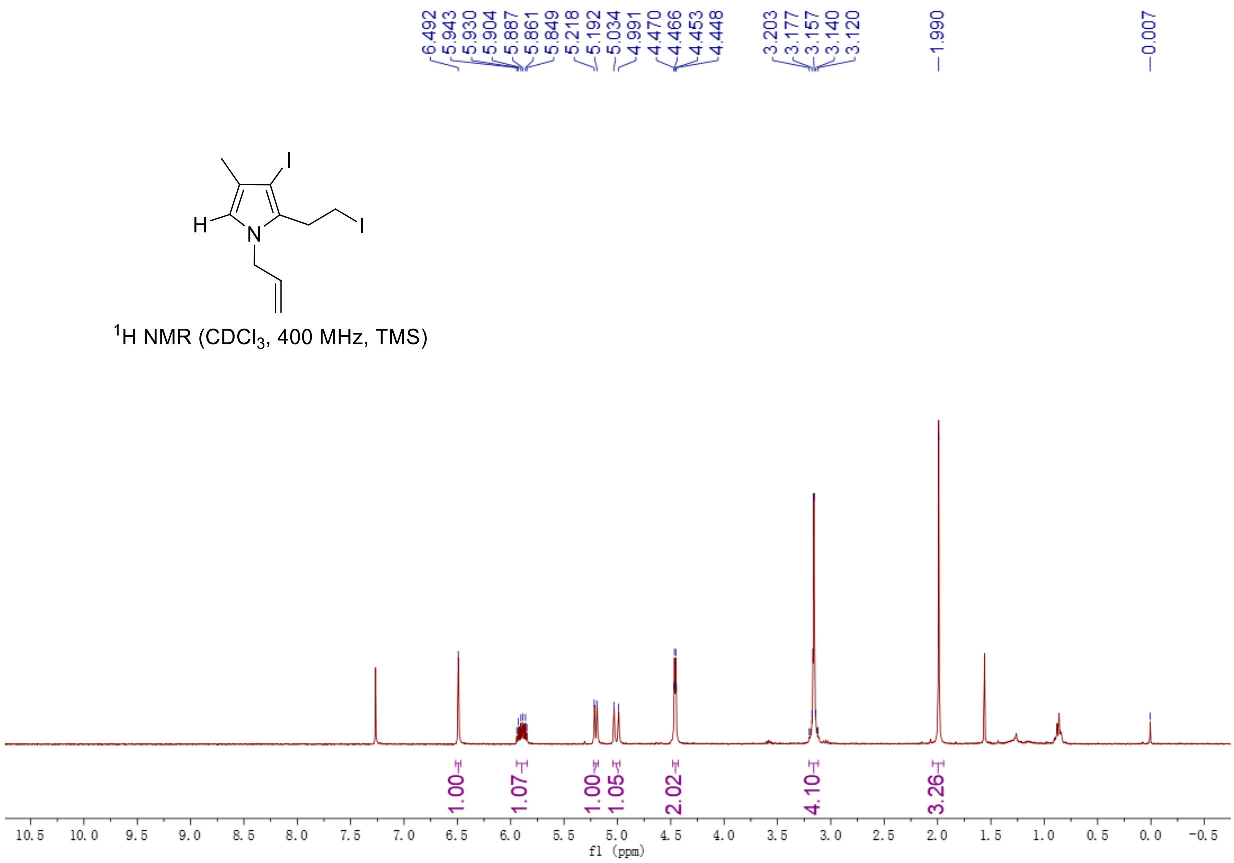

6.492
5.943
5.930
5.904
5.887
5.861
5.849
5.218
5.192
5.034
4.991
4.470
4.466
4.453
4.448
3.203
3.177
3.157
3.140
3.120
1.990
0.007
I
H
N
1H NMR (CDCl3, 400 MHz, TMS)
1.00
1.07
1.00
1.05
2.02
4.10
3.26
f1 (ppm)

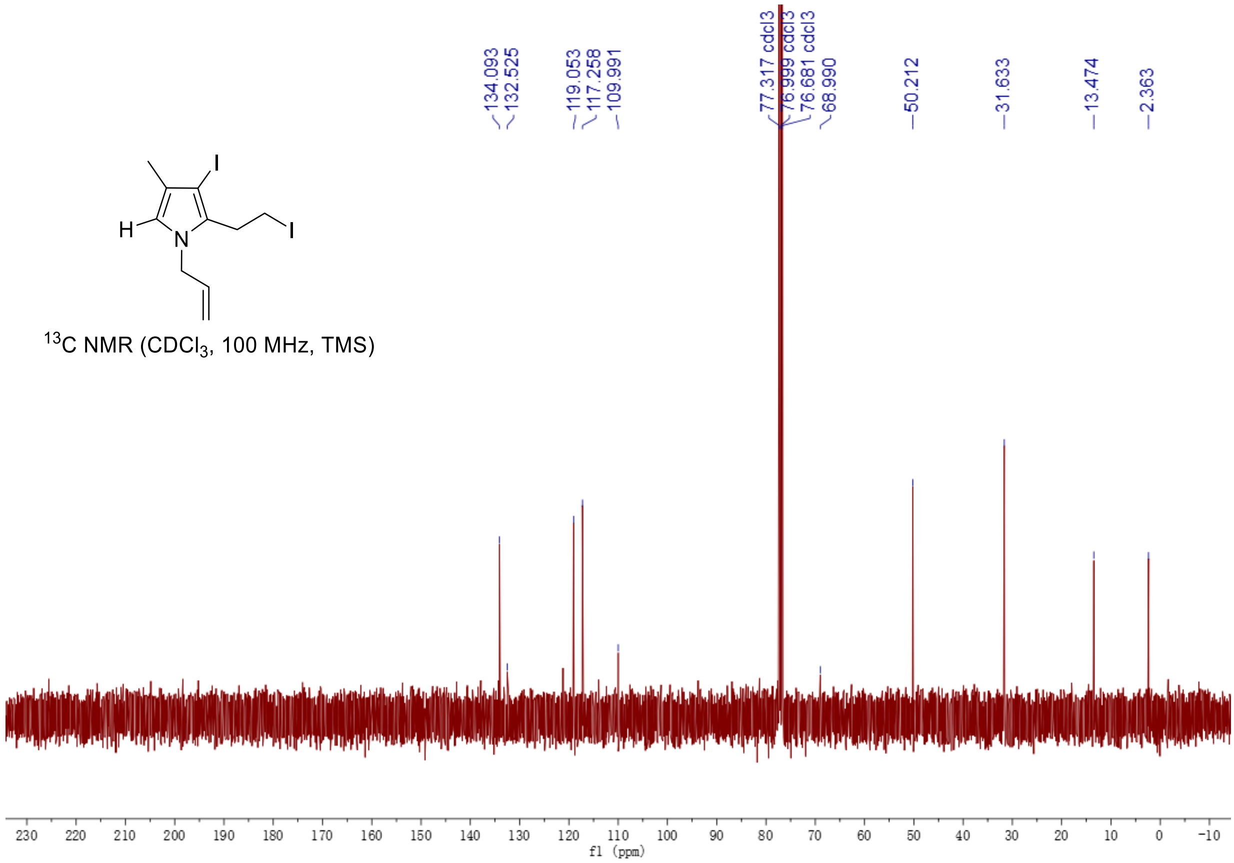

134.093
132.525
119.053
117.258
109.991
77.317 cdcl3
76.999 cdcl3
76.681 cdcl3
68.990
50.212
31.633
13.474
2.363
I
H
N
13C NMR (CDCl3, 100 MHz, TMS)
f1 (ppm)

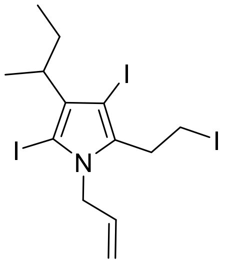

1-allyl-3-(sec-butyl)-2,4-diiodo-5-(2-iodoethyl)-1H-pyrrole (3sa)

A colorless oil, 52.3 mg, 92% yield. ^{1}H NMR ($CDCl_3$, TMS, 400 MHz) δ 5.93 - 5.79 (m, 1H), 5.19 (d, *J* = 10.4 Hz, 1H), 4.72 (d, *J* = 17.0 Hz, 1H), 4.66 - 4.56 (m, 2H), 3.28 - 3.17 (m, 2H), 3.20 - 3.08 (m, 2H), 2.81 - 2.67 (m, 1H), 1.98 - 1.81 (m, 1H), 1.66 - 1.51 (m, 1H), 1.27 (d, *J* = 7.2 Hz, 3H), 0.78 (t, *J* = 7.2 Hz, 3H). ^{13}C NMR ($CDCl_3$, TMS, 100 MHz) δ 137.4, 133.3, 131.5, 116.8, 51.2, 37.5, 33.2, 28.2, 19.5, 12.6, 1.9. IR (Neat) ν 733, 923, 989, 1087, 1169, 1326, 1374, 1451, 2875, 2925, 2956 cm^{-1}. HRMS (EI) calcd. for $C_{13}H_{18}NI_3$: 568.8579, Found: 568.8568.

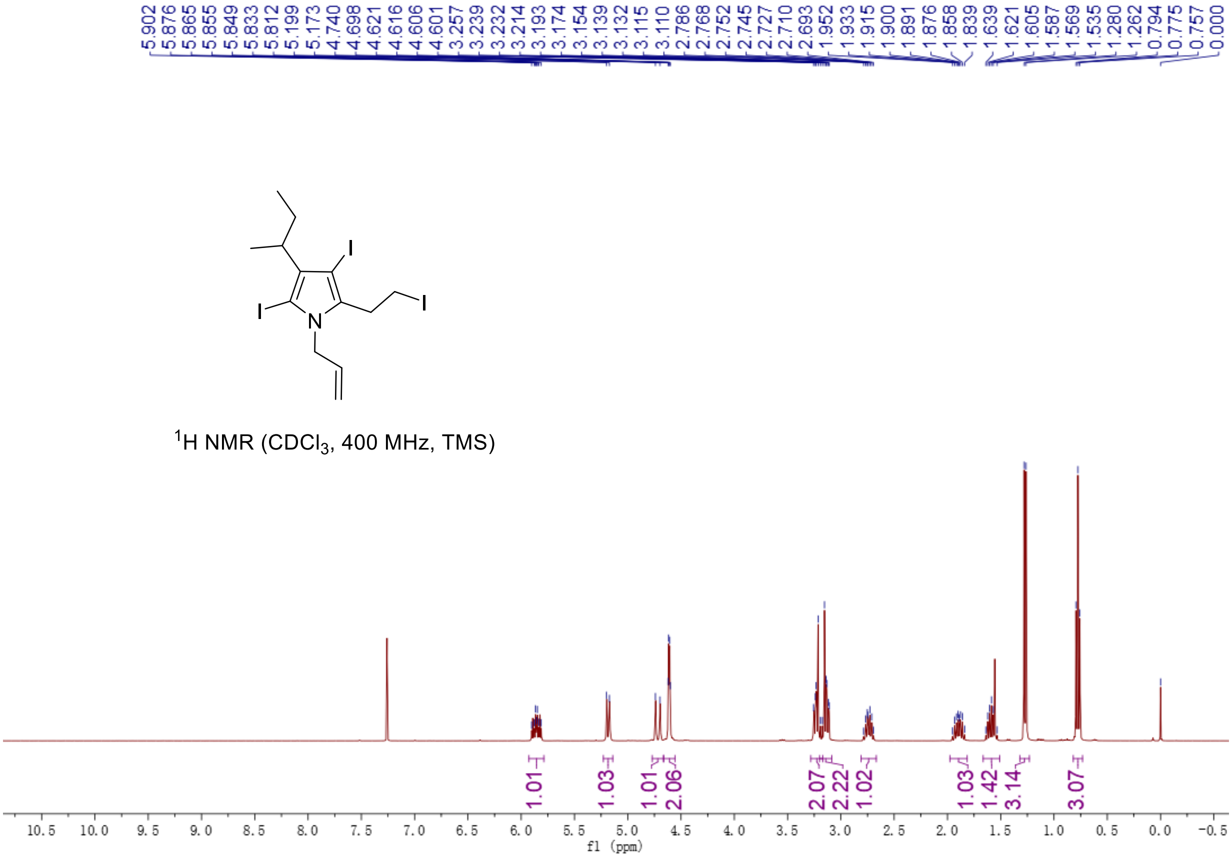

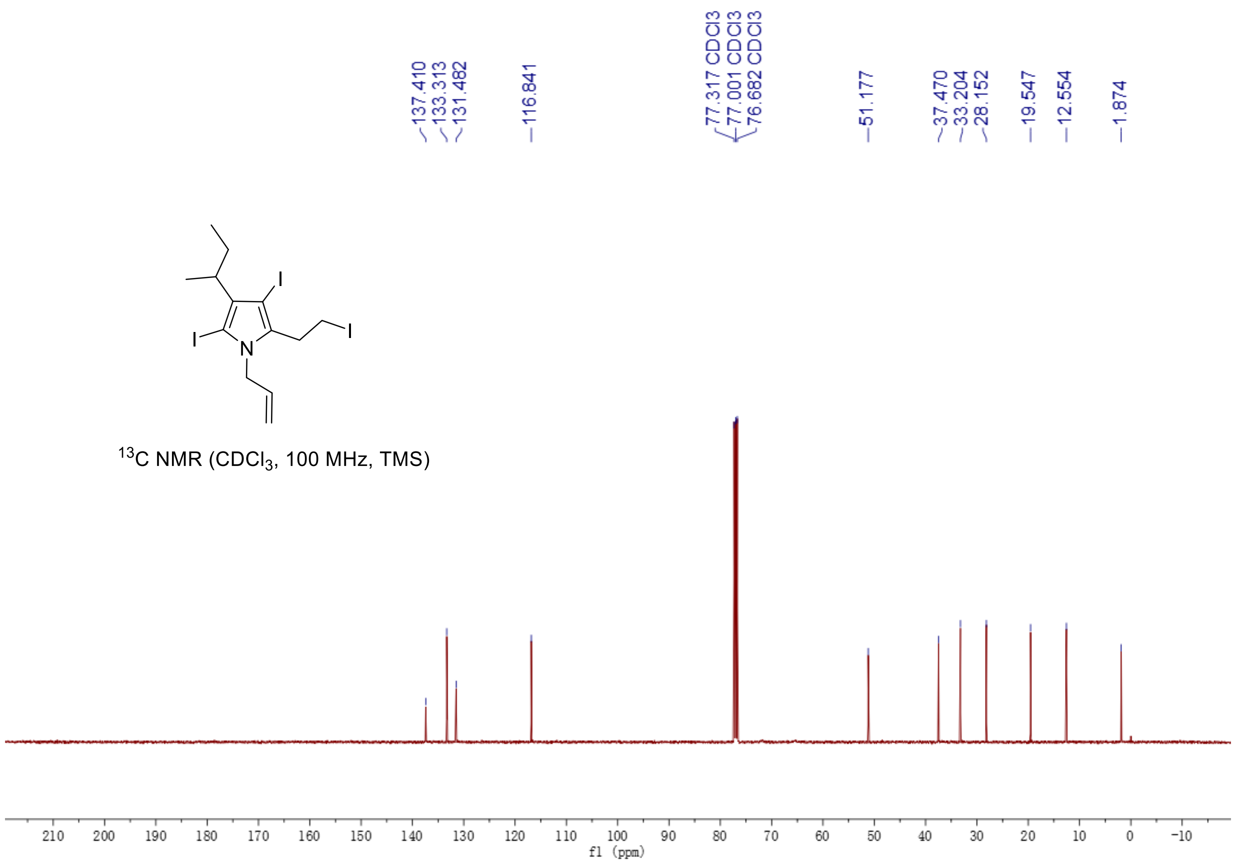

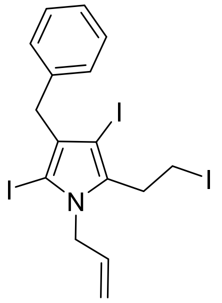

1-allyl-3-benzyl-2,4-diiodo-5-(2-iodoethyl)-1H-pyrrole (3ta)

A colorless oil, 48.2 mg, 80% yield. ^{1}H NMR ($CDCl_3$, TMS, 400 MHz) δ 7.30 - 7.13 (m, 7H), 5.91 - 5.81 (m, 1H), 5.21 (d, J = 10.4, 0.9 Hz, 1H), 4.80 (d, J = 17.0, 0.9 Hz, 1H), 4.67 - 4.59 (m, 2H), 3.82 (s, 2H), 3.28 - 3.08 (m, 4H). ^{13}C NMR ($CDCl_3$, TMS, 150 MHz) δ 139.8, 137.4, 133.1, 128.5, 128.2, 125.9, 117.1, 72.8, 68.7, 51.5, 36.1, 1.8. IR (Neat) ν 728, 925, 990, 1053 1171, 1348, 1493, 2917, 3021 cm^{-1}. HRMS (EI) calcd. for $C_{16}H_{16}I_3N$: 602.8433, Found: 602.8411.

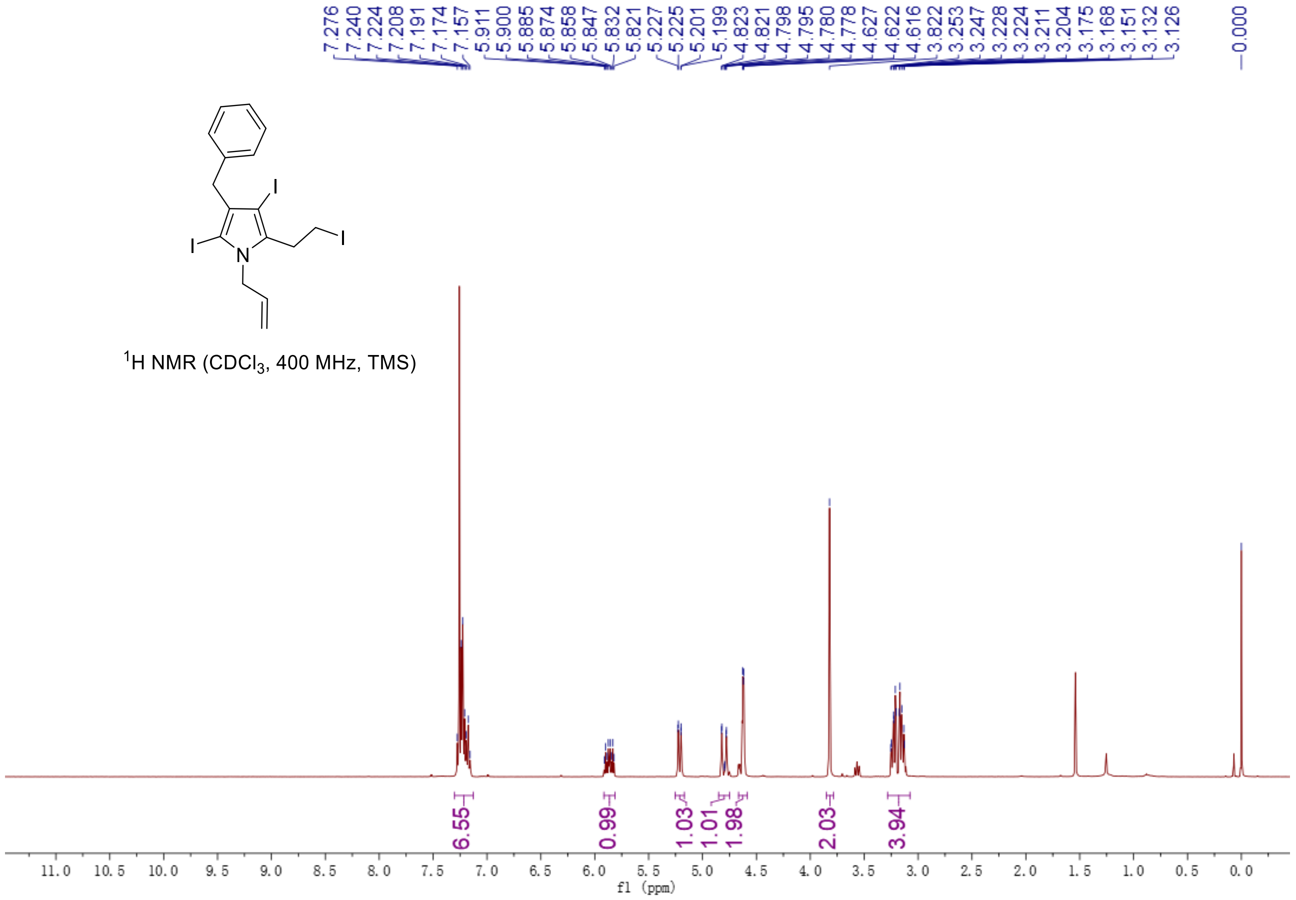
7.276 7.240 7.224 7.208 7.191 7.174 7.157 5.911 5.900 5.885 5.874 5.858 5.847 5.832 5.821 5.227 5.225 5.201 5.199 4.823 4.821 4.798 4.795 4.780 4.778 4.627 4.622 4.616 3.822 3.253 3.247 3.228 3.224 3.211 3.204 3.175 3.168 3.151 3.132 3.126 0.000
^{1}H NMR (CDCl$_3$, 400 MHz, TMS)
6.55 0.99 1.03 1.01 1.98 2.03 3.94
f1 (ppm)

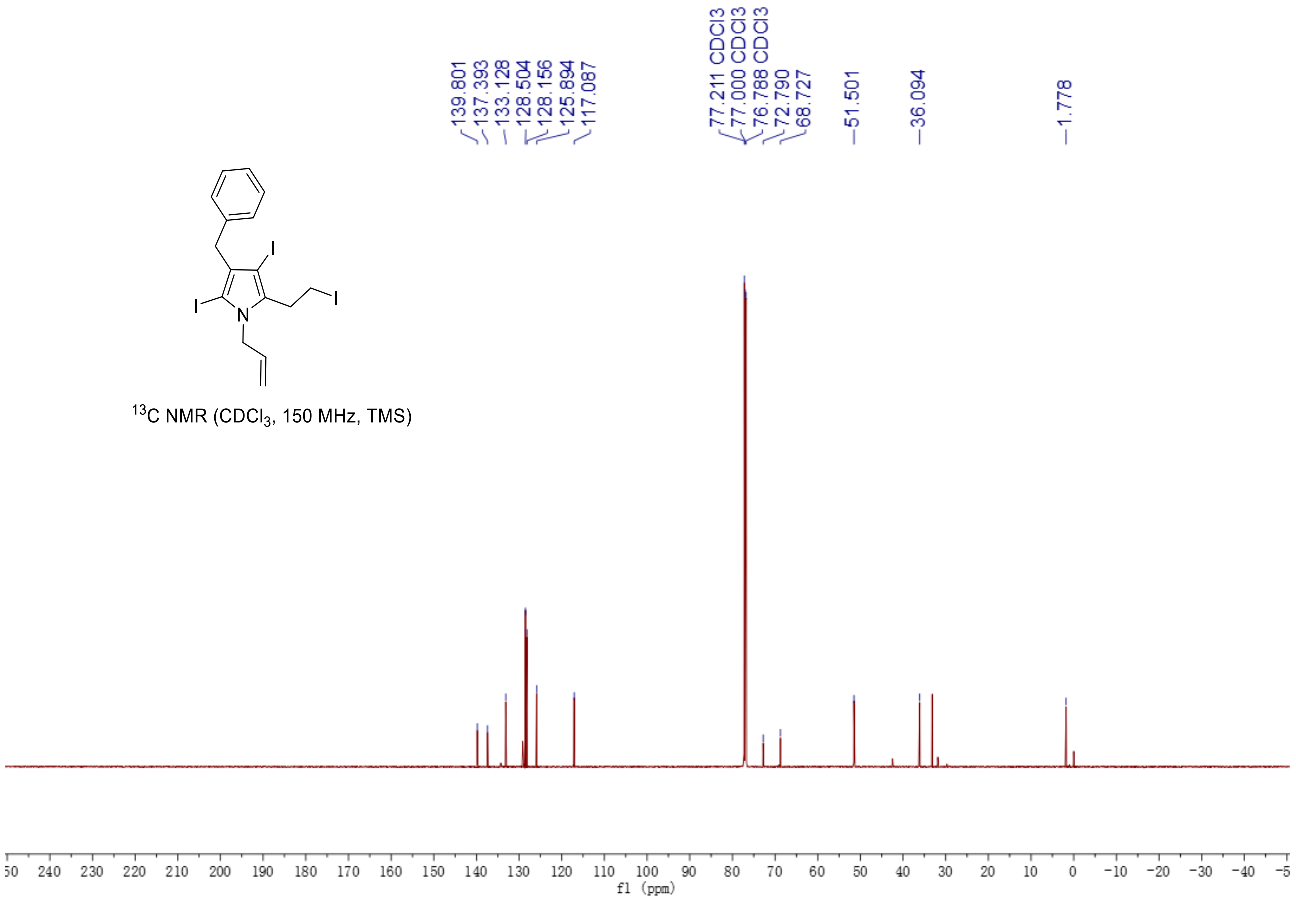
139.801 137.393 133.128 128.504 128.156 125.894 117.087 77.211 CDCl3 77.000 CDCl3 76.788 CDCl3 72.790 68.727 51.501 36.094 1.778
^{13}C NMR (CDCl$_3$, 150 MHz, TMS)
f1 (ppm)

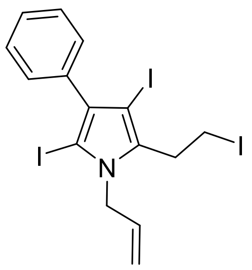

1-allyl-2,4-diiodo-5-(2-iodoethyl)-3-phenyl-1H-pyrrole (3ua)

A colorless oil, 5.89 mg, 10% yield. ^{1}H NMR ($CDCl_3$, TMS, 400 MHz) δ 7.44 - 7.39 (m, 2H), 7.38 - 7.32 (m, 3H), 5.96 - 5.86 (m, 1H), 5.26 (d, J = 10.3 Hz, 1H), 4.98 - 4.88 (m, 1H), 4.74 - 4.65 (m, 2H), 3.34 - 3.18 (m, 4H). ^{13}C NMR ($CDCl_3$, TMS, 100 MHz) δ 137.8, 133.1, 130.5, 127.9, 127.4, 117.4, 72.2, 67.8, 51.9, 33.5, 26.9, 1.5. IR (Acetone) ν 3059, 2969, 2928, 1603, 1492, 1461, 1445, 1359, 1339, 1302, 1219, 1062, 1046, 1028, 905, 810, 759, 738, 700 cm^{-1}. HRMS (ESI) calcd. for $C_{15}H_{14}I_3NNa$ (M+Na): 611.8758, Found: 611.9030.

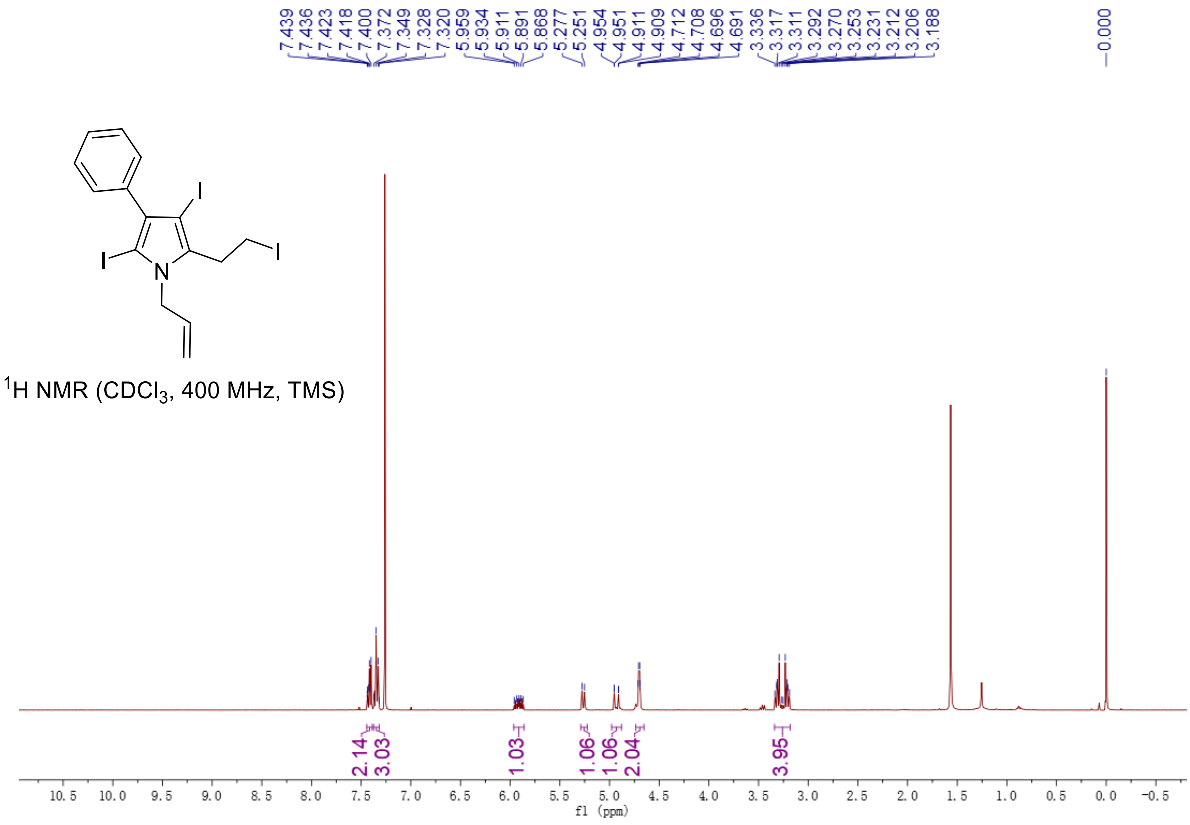

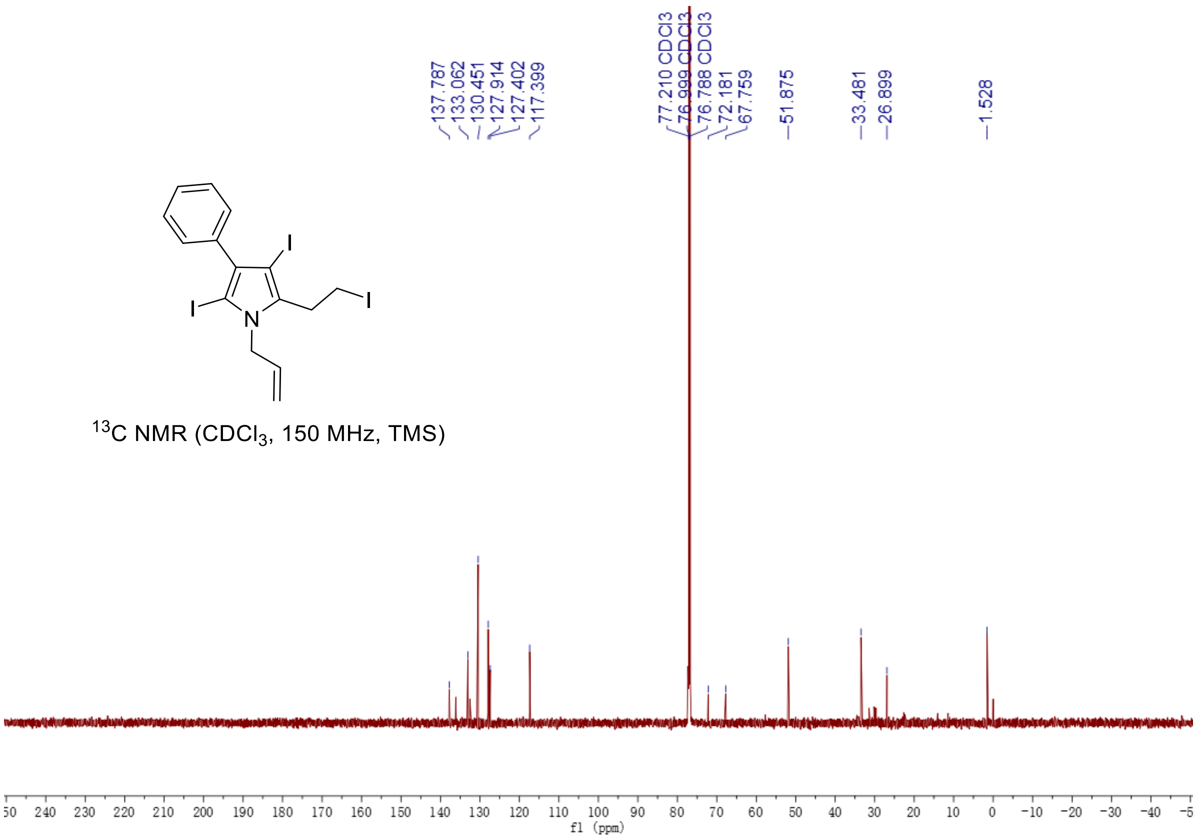

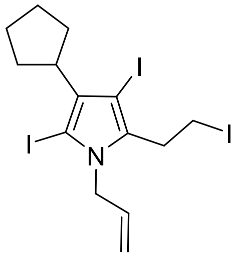

1-allyl-3-cyclopentyl-2,4-diiodo-5-(2-iodoethyl)-1H-pyrrole (3va)

A colorless oil, 50.5 mg, 87% yield. ^{1}H NMR ($CDCl_3$, TMS, 400 MHz) δ 5.92 - 5.78 (m, 1H), 5.21 (d, *J* = 10.4 Hz, 1H), 4.84 (d, *J* = 17.2 Hz, 1H), 4.68 - 4.55 (m, 2H), 3.31 - 3.20 (m, 2H), 3.17 - 3.09 (m, 2H), 3.09 - 2.96 (m, 1H), 1.99 - 1.86 (m, 4H), 1.84 - 1.75 (m, 2H), 1.73 - 1.62 (m, 2H). ^{13}C NMR ($CDCl_3$, TMS, 100 MHz) δ 137.5, 133.3, 130.0, 117.2, 71.7, 65.6, 51.4, 41.2, 33.3, 31.4, 26.4, 1.7. IR (Neat) ν 731, 906, 1169, 1341, 1398, 1448, 1647, 2859, 2949 cm^{-1}. HRMS (EI) calcd. for $C_{14}H_{18}I_3N$: 580.8584, Found: 580.8568.

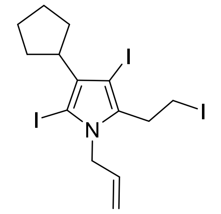

^1H NMR ($CDCl_3$, 400 MHz, TMS)

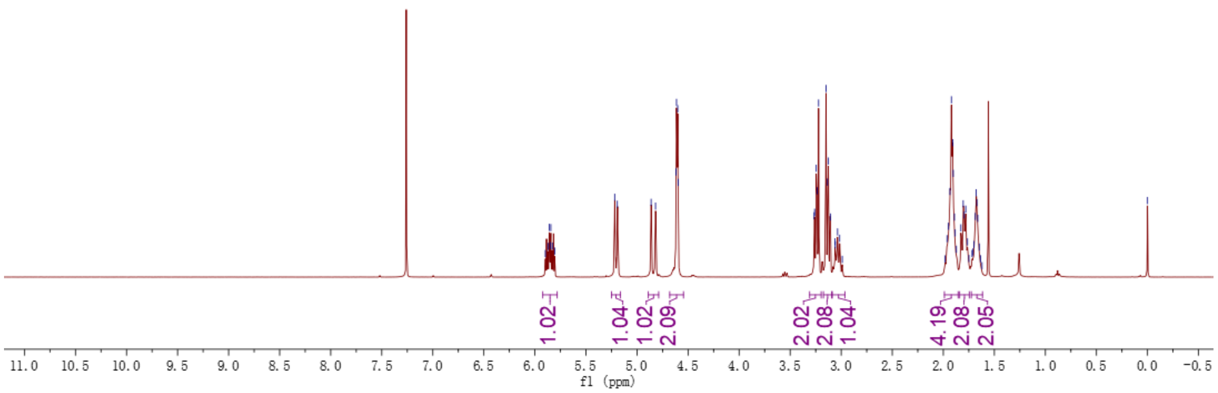

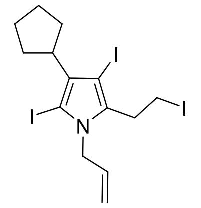

^{13}C NMR ($CDCl_3$, 100 MHz, TMS)

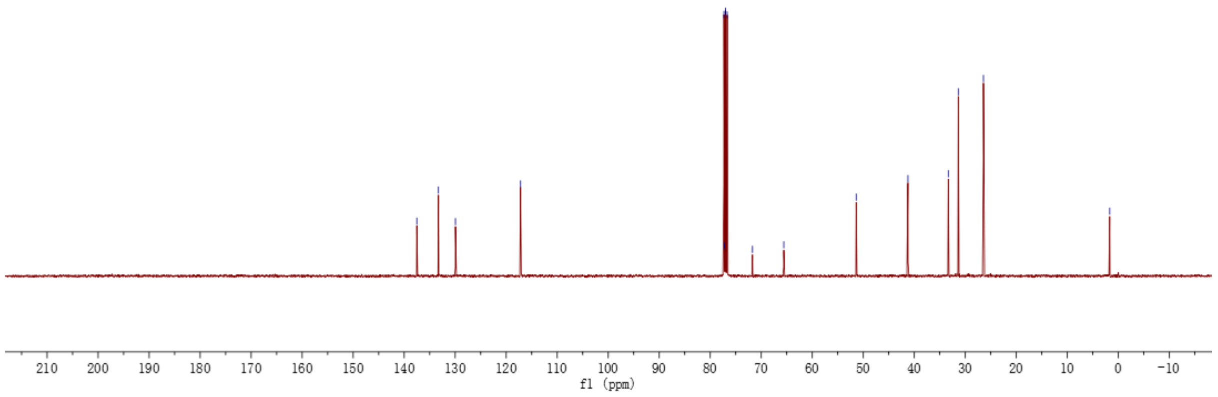

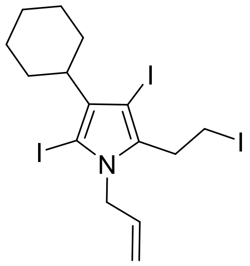

1-allyl-3-cyclohexyl-2,4-diiodo-5-(2-iodoethyl)-1H-pyrrole (3wa)

A colorless oil, 48.7 mg, 82% yield. ^{1}H NMR ($CDCl_3$, TMS, 400 MHz) δ 5.92 - 5.78 (m, 1H), 5.19 (d, J = 10.4 Hz, 1H), 4.79 (d, J = 17.1 Hz, 1H), 4.66 - 4.56 (m, 2H), 3.28 - 3.18 (m, 2H), 3.17 - 3.07 (m, 2H), 2.67 - 2.54 (m, 1H), 2.11 - 1.96 (m, 2H), 1.89 - 1.78 (m, 2H), 1.74 - 1.64 (m, 1H), 1.58 - 1.49 (m, 2H), 1.44 - 1.22 (m, 3H). ^{13}C NMR ($CDCl_3$, TMS, 100 MHz) δ 137.4, 133.3, 132.3, 117.1, 76.7, 51.3, 40.9, 33.4, 30.8, 27.0, 25.9, 1.7. IR (Acetone) ν 717, 921, 1172, 1337, 1402, 1431, 1645, 2847, 2925 cm^{-1}. HRMS (EI) calcd. for $C_{15}H_{20}I_3N$: 594.8753, Found: 594.8724.

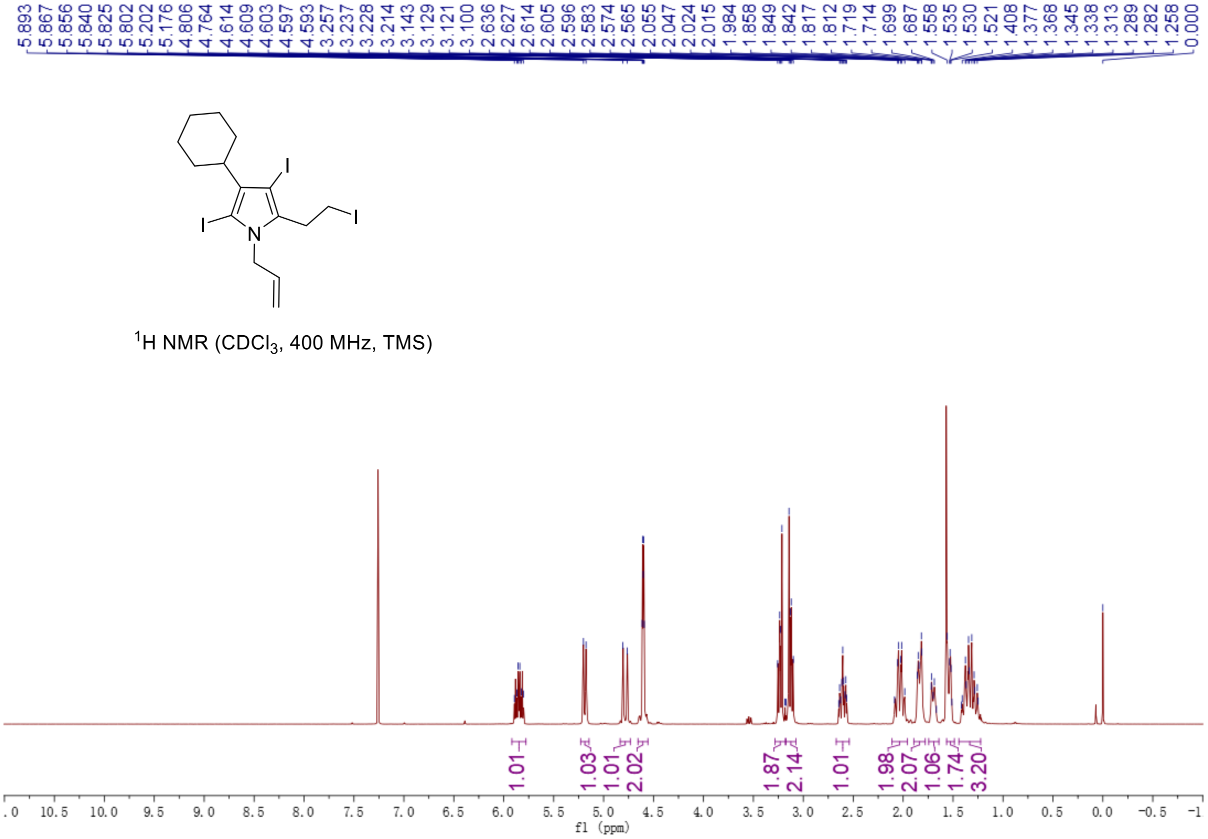

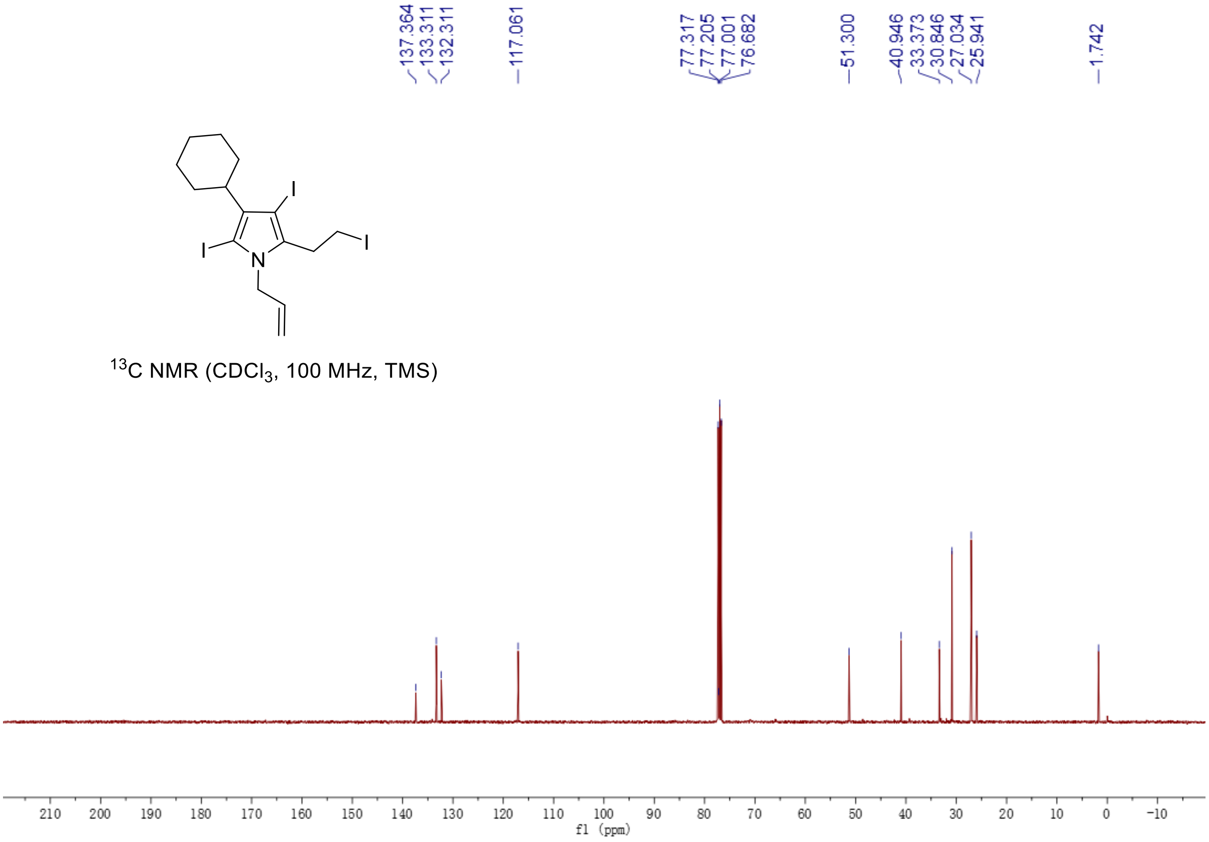

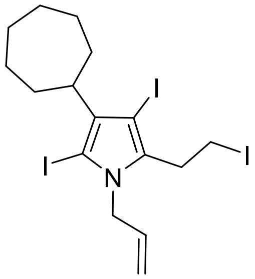

1-allyl-3-cycloheptyl-2,4-diiodo-5-(2-iodoethyl)-1H-pyrrole (3xa)

A colorless oil, 49.3 mg, 81% yield. ^{1}H NMR ($CDCl_3$, TMS, 400 MHz) δ 5.92 - 5.77 (m, 1H), 5.19 (d, J = 10.4 Hz, 1H), 4.77 (d, J = 17.2 Hz, 1H), 4.65 - 4.53 (m, 2H), 3.27 - 3.17 (m, 2H), 3.17 - 3.07 (m, 2H), 2.74 (tt, J = 11.2, 3.6 Hz, 1H), 2.16 - 2.00 (m, 2H), 1.85 - 1.74 (m, 2H), 1.75 - 1.60 (m, 4H), 1.59 - 1.45 (m, 4H). ^{13}C NMR ($CDCl_3$, TMS, 100 MHz) δ 137.3, 135.0, 133.3, 117.0, 76.7, 51.3, 42.6, 33.5, 33.3, 28.1, 27.8, 1.8. IR (Neat) ν 731, 921, 1170, 1327, 1401, 1444, 2235, 2848 cm^{-1}. HRMS (EI) calcd. for $C_{16}H_{22}I_3N$: 608.8910, Found: 608.8881.

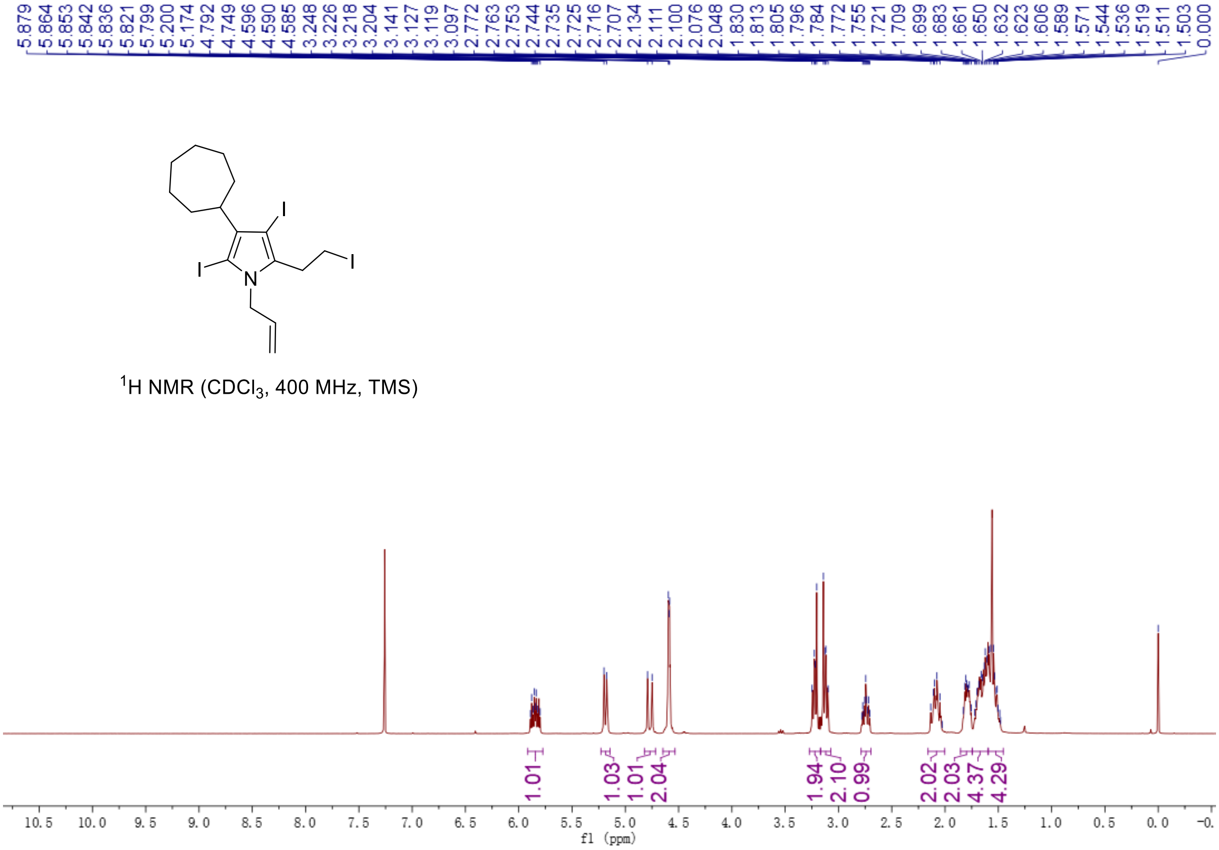

^{1}H NMR ($CDCl_3$, 400 MHz, TMS)

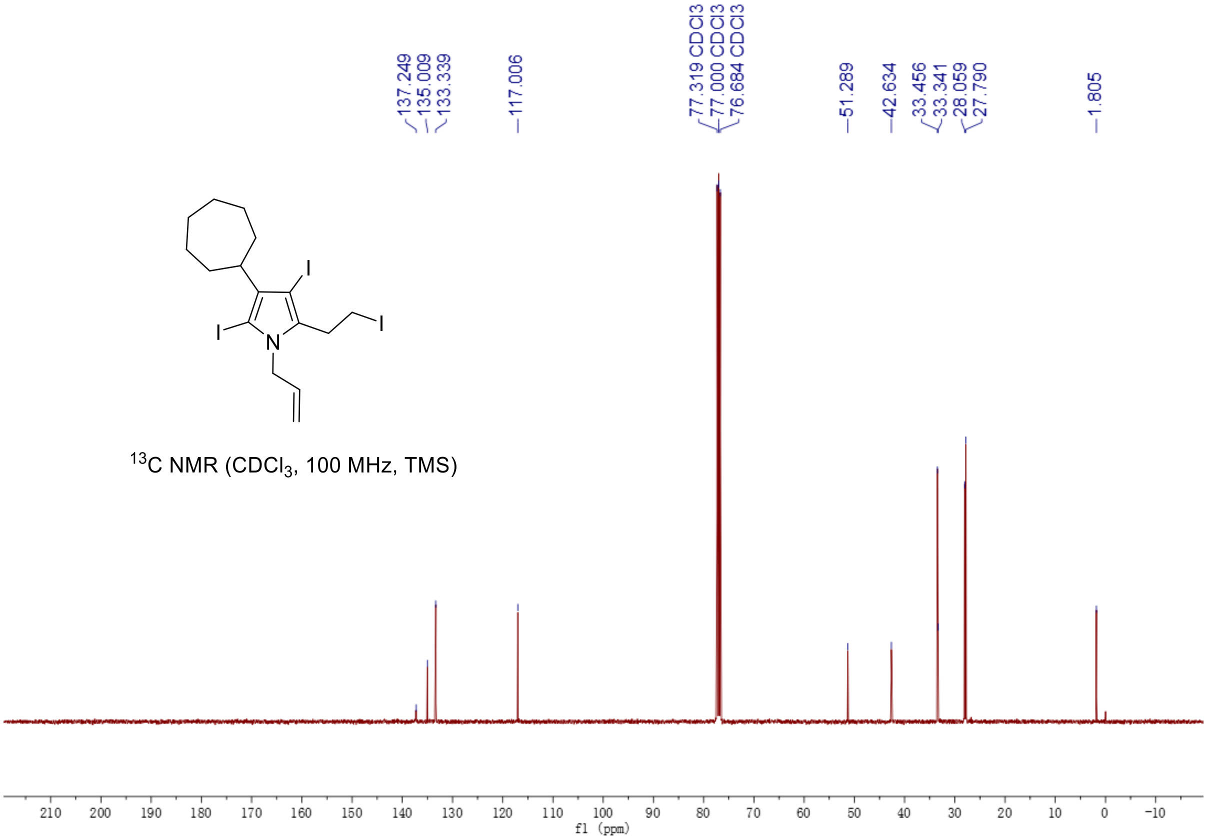

^{13}C NMR ($CDCl_3$, 100 MHz, TMS)

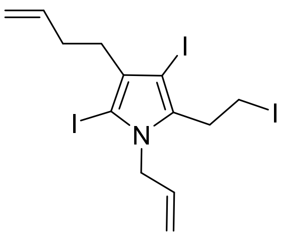

1-allyl-3-(but-3-en-1-yl)-2,4-diiodo-5-(2-iodoethyl)-1H-pyrrole (3ya)

A colorless oil, 41.4 mg, 73% yield. ^{1}H NMR ($CDCl_3$, TMS, 400 MHz) δ 5.96 - 5.78 (m, 2H), 5.19 (d, *J* = 10.1 Hz, 1H), 5.05 (d, *J* = 17.1 Hz, 1H), 4.97 (d, *J* = 10.9 Hz, 1H), 4.76 (d, *J* = 17.1 Hz, 1H), 4.65 - 4.54 (m, 2H), 3.26 - 3.18 (m, 2H), 3.17 - 3.10 (m, 2H), 2.56 - 2.45 (m, 2H), 2.21 (q, *J* = 7.1 Hz, 2H). ^{13}C NMR ($CDCl_3$, TMS, 100 MHz) δ 138.0, 137.0, 133.2, 129.7, 117.0, 114.9, 71.4, 67.9, 51.3, 34.0, 33.1, 30.0, 1.8. IR (Neat) ν 729, 906, 1050, 1169, 1300 1332, 1453, 2248, 2966 cm^{-1}. HRMS (ESI) calcd. for $C_{13}H_{17}I_3N$ (M+H): 567.8432, Found: 567.8661.

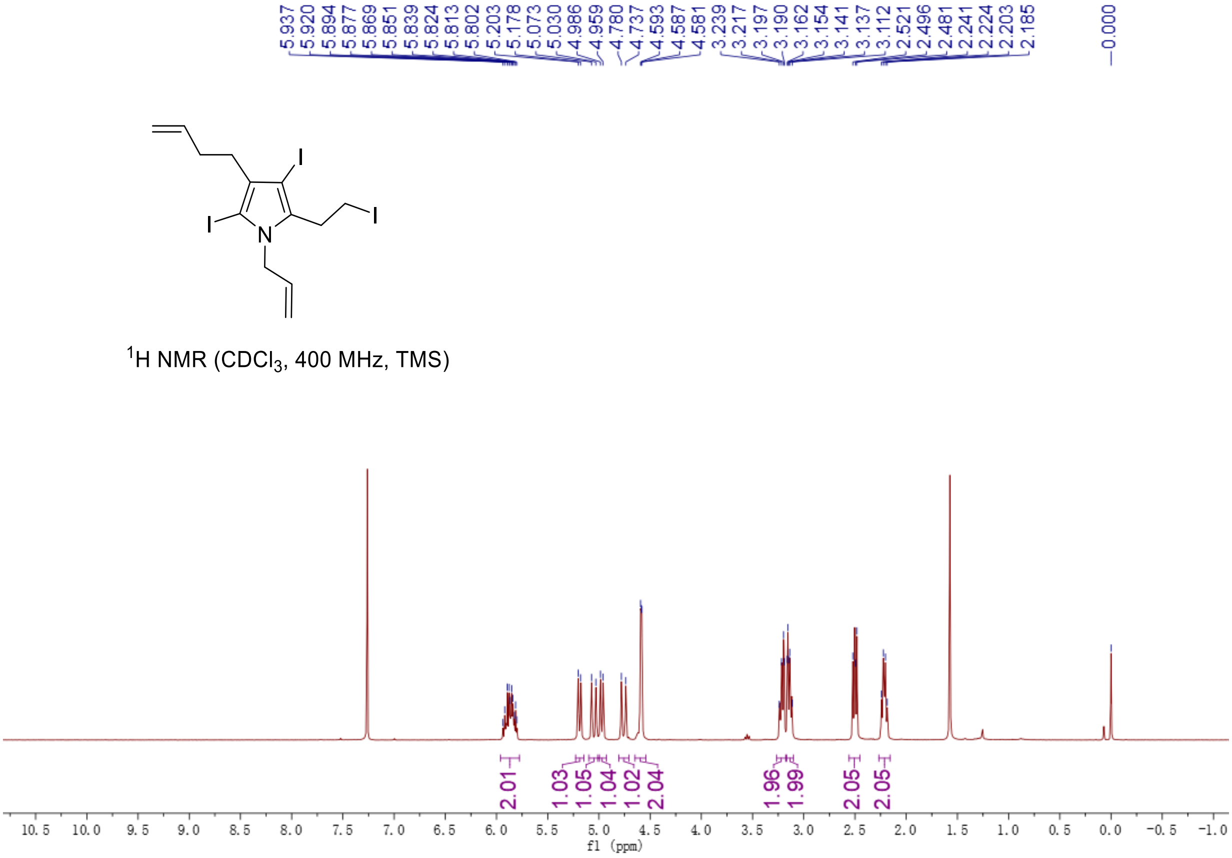

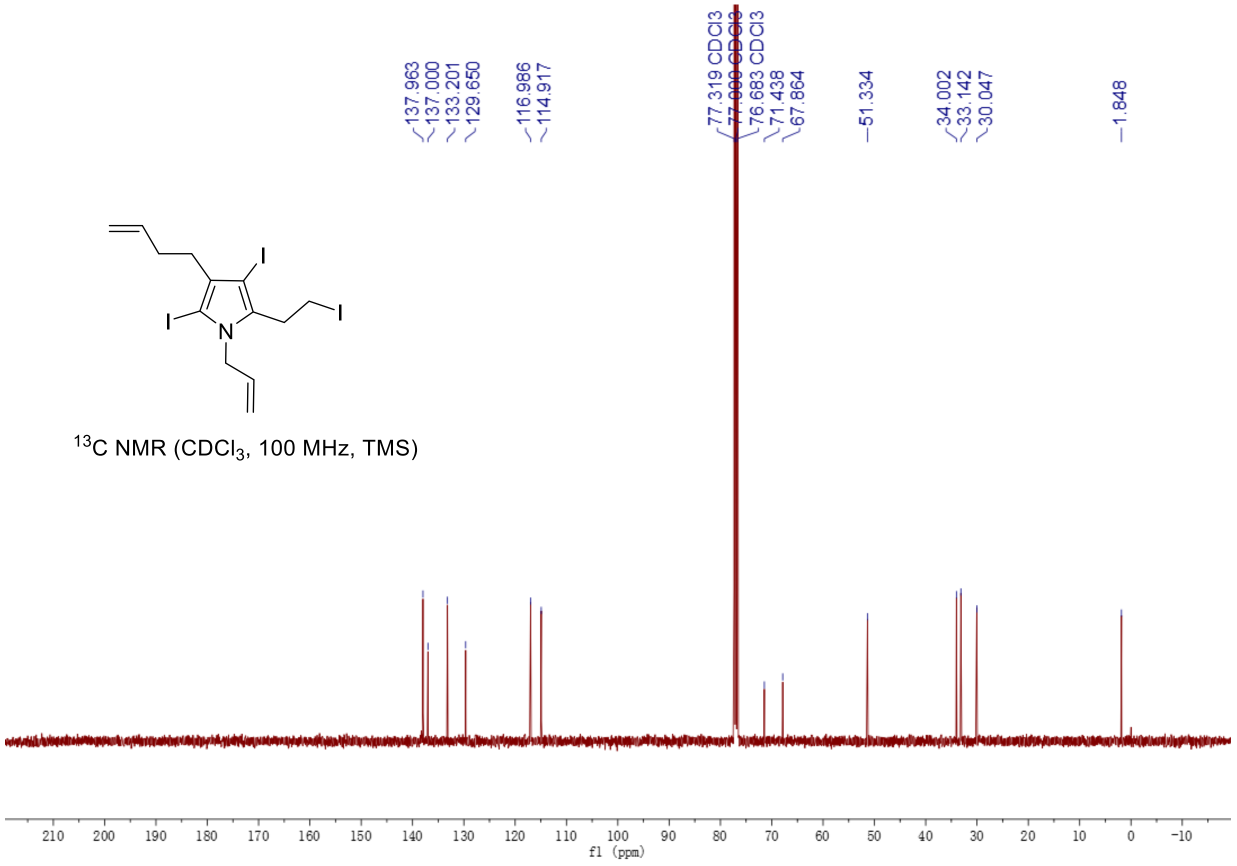

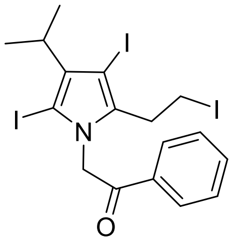

2-(2,4-diiodo-5-(2-iodoethyl)-3-isopropyl-1H-pyrrol-1-yl)-1-phenylethan-1-one (3za)

A colorless oil, 27.2 mg, 43% yield. ^{1}H NMR ($CDCl_3$, TMS, 400 MHz) δ 8.03 (d, *J* = 7.0 Hz, 2H), 7.69 (t, *J* = 7.5 Hz, 1H), 7.57 (t, *J* = 7.8 Hz, 2H), 5.45 (s, 2H), 3.25 - 3.09 (m, 4H), 3.02 (p, *J* = 7.1 Hz, 1H), 1.32 (d, *J* = 7.1 Hz, 6H). ^{13}C NMR ($CDCl_3$, TMS, 100 MHz) δ 192.4, 138.0, 134.4, 133.4, 129.1, 128.1, 117.7, 104.9, 55.0, 50.4, 33.5, 30.7, 21.3, 1.7. IR (Acetone) ν 733, 925, 995, 1210.60, 1271, 1313, 1353, 1449, 1715, 2950 cm^{-1}. HRMS (ESI) calcd. for $C_{17}H_{18}I_3NONa$ (M+Na): 655.8420, Found: 655.8408.

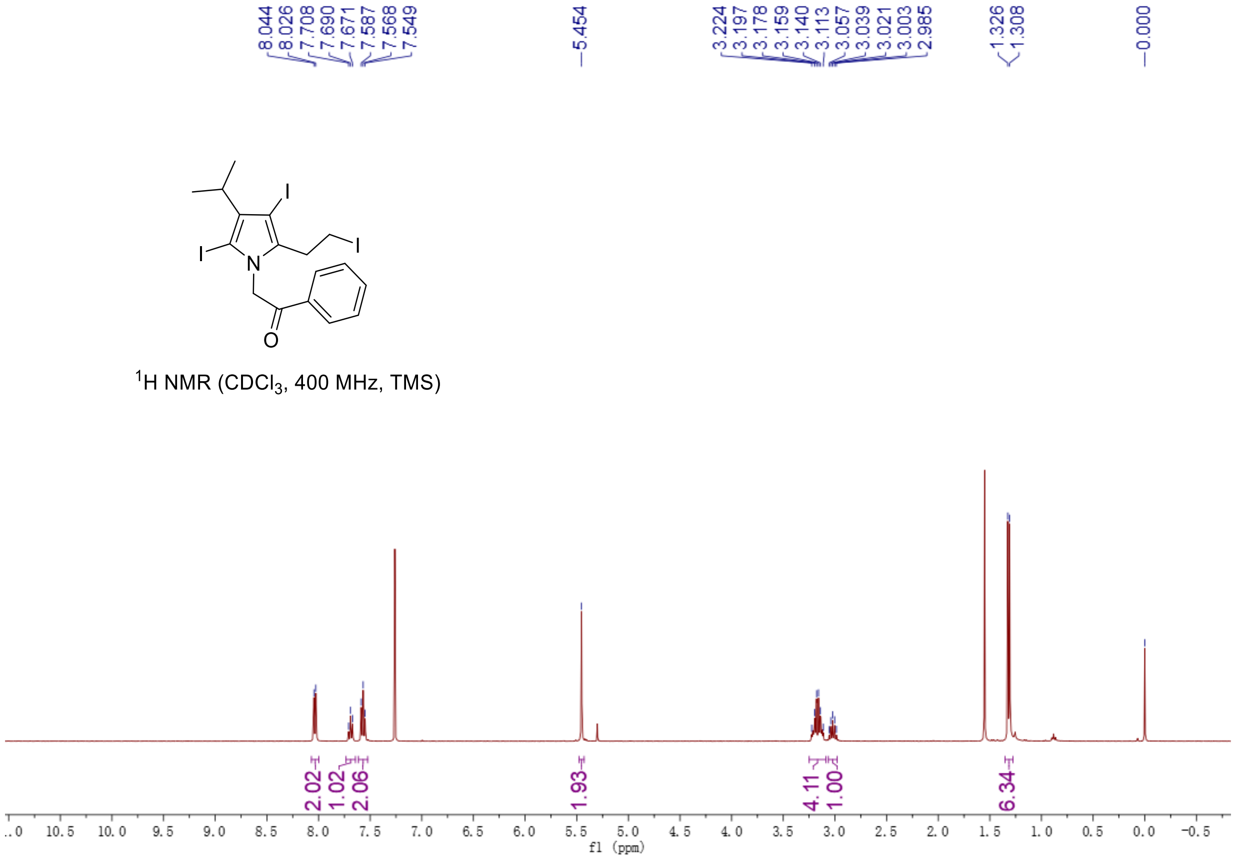
8.044
8.026
7.708
7.690
7.671
7.587
7.568
7.549
5.454
3.224
3.197
3.178
3.159
3.140
3.113
3.057
3.039
3.021
3.003
2.985
1.326
1.308
0.000
I
I
I
N
O
^{1}H NMR (CDCl$_3$, 400 MHz, TMS)
2.02
1.02
2.06
1.93
4.11
1.00
6.34
f1 (ppm)

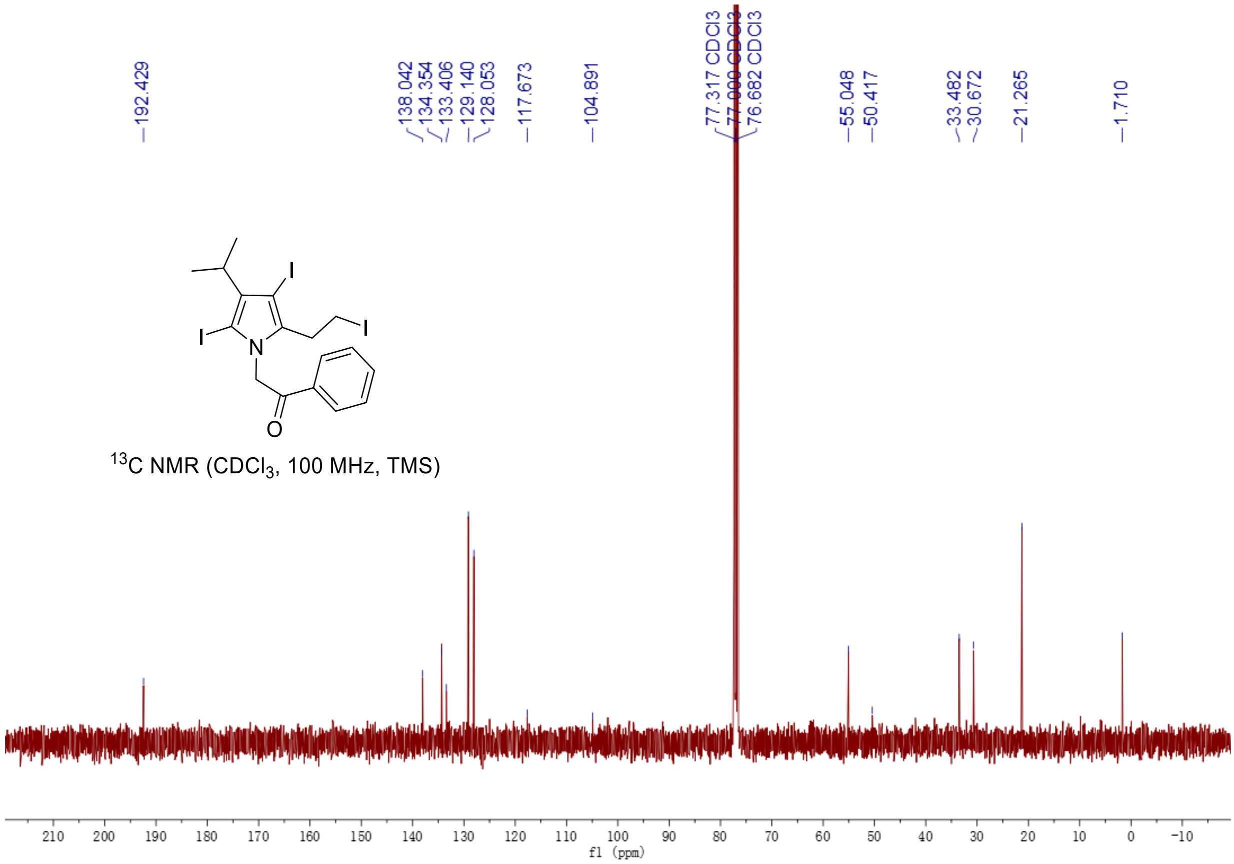
192.429
138.042
134.354
133.406
129.140
128.053
117.673
104.891
77.317 CDCl3
77.000 CDCl3
76.682 CDCl3
55.048
50.417
33.482
30.672
21.265
1.710
I
I
I
N
O
^{13}C NMR (CDCl$_3$, 100 MHz, TMS)
f1 (ppm)

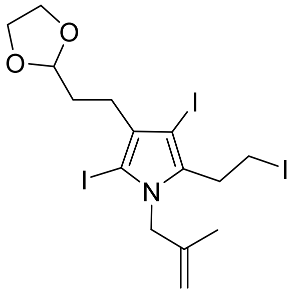

3-(2-(1,3-dioxolan-2-yl)ethyl)-2,4-diiodo-5-(2-iodoethyl)-1-(2-methylallyl)-1H-pyrrole (3afa)

A colorless oil, 50.8 mg, 81% yield. ^{1}H NMR ($CDCl_3$, TMS, 400 MHz) δ 4.91 (t, *J* = 4.7 Hz, 1H), 4.87 (s, 1H), 4.44 (s, 2H), 4.23 (s, 1H), 4.06 - 3.96 (m, 2H), 3.92 - 3.83 (m, 2H), 3.24 - 3.06 (m, 4H), 2.59 - 2.50 (m, 2H), 1.87 - 1.77 (m, 2H), 1.75 (s, 3H). ^{13}C NMR ($CDCl_3$, TMS, 100 MHz) δ 140.9, 137.2, 129.3, 111.8, 103.8, 72.1, 67.7, 64.9, 54.5, 33.8, 33.2, 25.2, 20.0, 1.8. IR (Acetone) ν 716, 896, 965, 1066, 1180, 1325, 1445, 2920 cm^{-1}. HRMS (ESI) calcd. for $C_{15}H_{20}NO_2NaI_3$ (M+Na): 649.8520, Found: 649.8517.

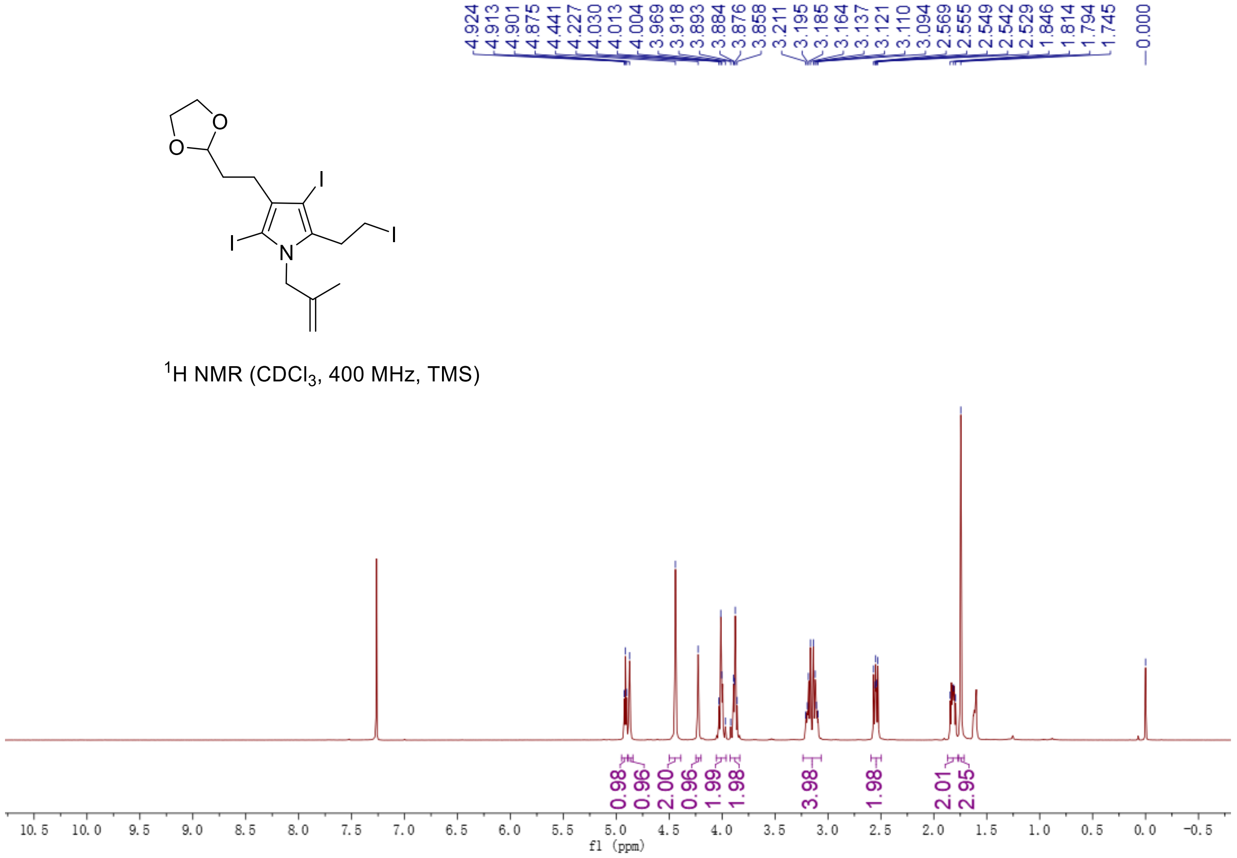

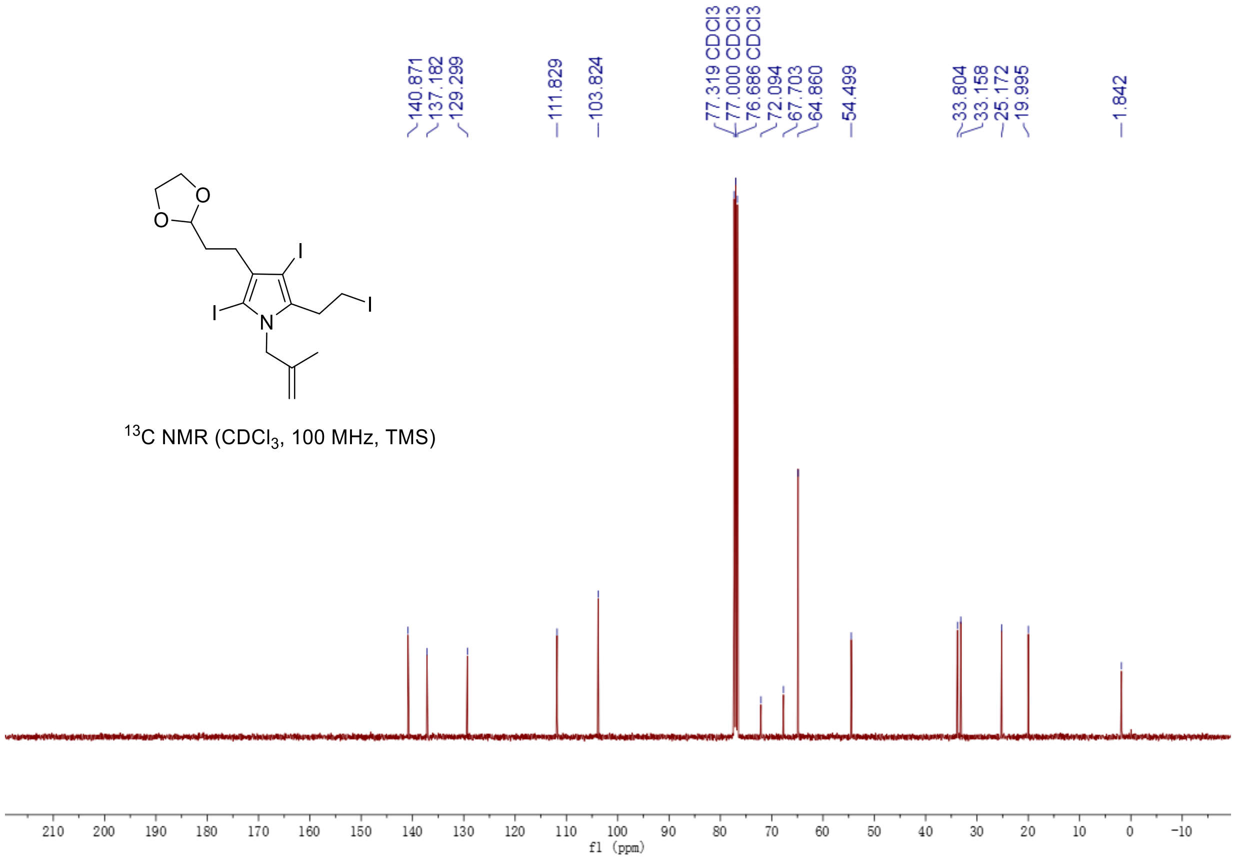

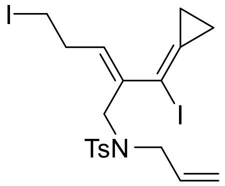

(*E*)-*N*-allyl-*N*-(2-(cyclopropylideneiodomethyl)-5-iodopent-2-en-1-yl)-4-methylbenzenesulfonamide (3aja)

A colorless oil, 50.8 mg, 40% yield. ^{1}H NMR ($CDCl_3$, TMS, 400 MHz) δ 7.71 (d, J = 8.3 Hz, 2H), 7.28 (d, J = 8.2 Hz, 2H), 5.63 - 5.52 (m, 1H), 5.40 (t, J = 7.0 Hz, 1H), 5.26 - 5.12 (m, 2H), 3.95 (s, 2H), 3.84 (d, J = 6.5 Hz, 2H), 3.14 (t, J = 7.0 Hz, 2H), 2.61 (q, J = 7.0 Hz, 2H), 2.43 (s, 3H), 1.49 - 1.43 (m, 2H), 1.16 - 1.11 (m, 2H). ^{13}C NMR ($CDCl_3$, TMS, 100 MHz) δ 143.1, 138.1, 137.7, 136.1, 132.3, 130.6, 129.6, 127.2, 119.8, 81.8, 50.0, 49.4, 32.7, 21.5, 13.9, 9.6, 3.3. IR (Acetone) ν 719, 890, 993, 1036, 1200, 1325, 1455, 2930 cm^{-1}. HRMS (ESI) calcd. for $C_{19}H_{23}NO_2NaSI_2$ (M+Na): 605.9431, Found: 605.9442

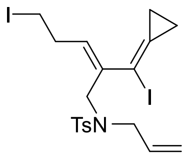

^{1}H NMR ($CDCl_3$, 400 MHz, TMS)

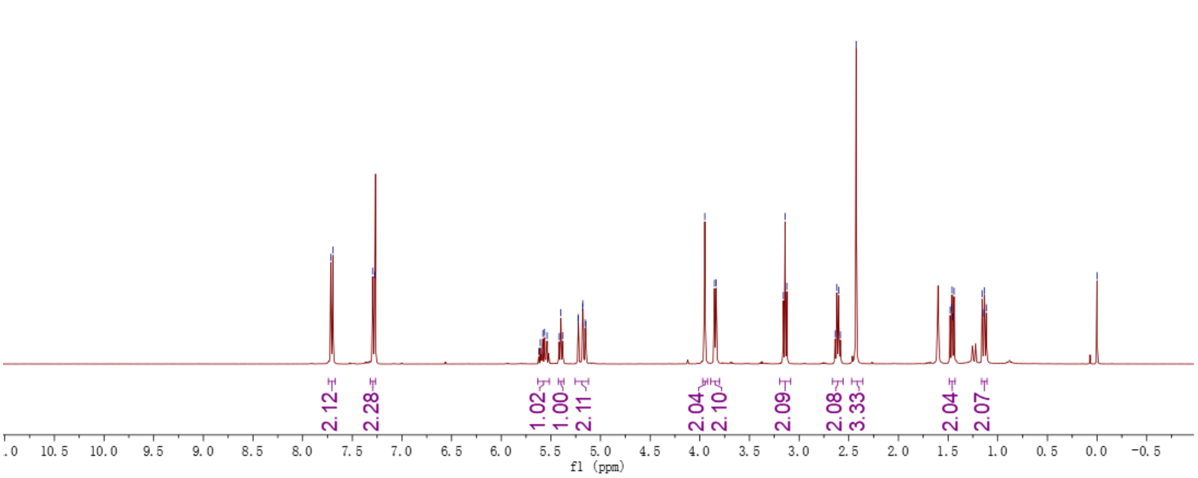

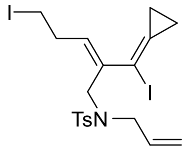

^{13}C NMR ($CDCl_3$, 100 MHz, TMS)

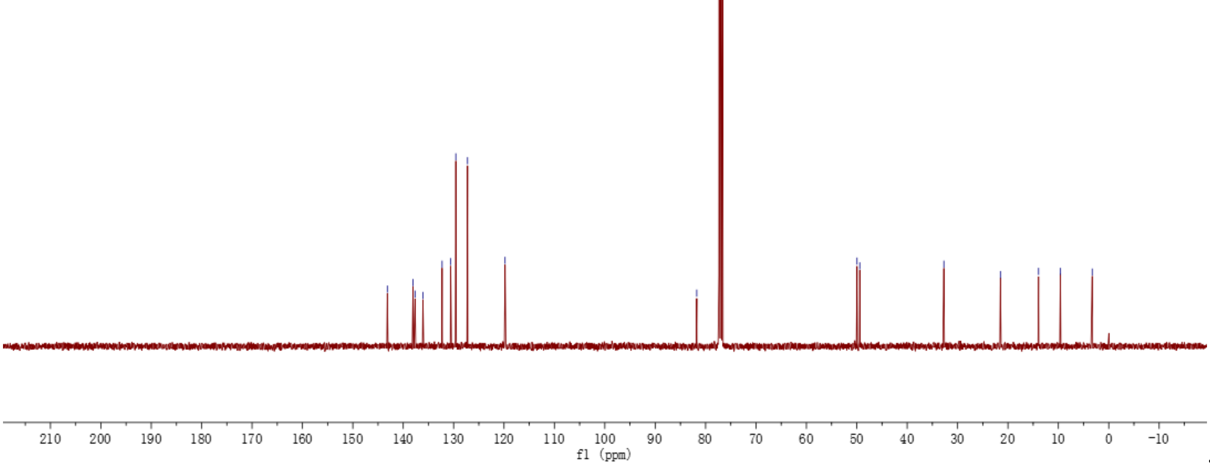

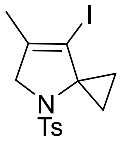

7-iodo-6-methyl-4-tosyl-4-azaspiro[2.4]hept-6-ene (3aka')

A colorless oil, 35.8 mg, 46% yield. ^{1}H NMR ($CDCl_3$, TMS, 400 MHz) δ 7.93 (d, *J* = 8.4 Hz, 2H), 7.36 (d, *J* = 8.1 Hz, 2H), 4.23 (d, *J* = 9.7 Hz, 1H), 3.79 (d, *J* = 9.6 Hz, 1H), 2.46 (s, 3H), 1.76 (s, 4H), 1.69 - 1.61 (m, 1H), 1.13 - 0.99 (m, 2H). ^{13}C NMR ($CDCl_3$, TMS, 100 MHz) δ 150.8, 145.7, 136.2, 134.1, 129.9, 128.3, 91.1, 82.7, 55.6, 25.5, 21.8, 15.7, 8.3. IR (Acetone) ν 694, 778, 901, 957, 1123, 1210, 1335, 1445, 2960 cm^{-1}. HRMS (ESI) calcd. for $C_{14}H_{17}NO_2SI$ (M+H): 390.0019, Found: 390.0001

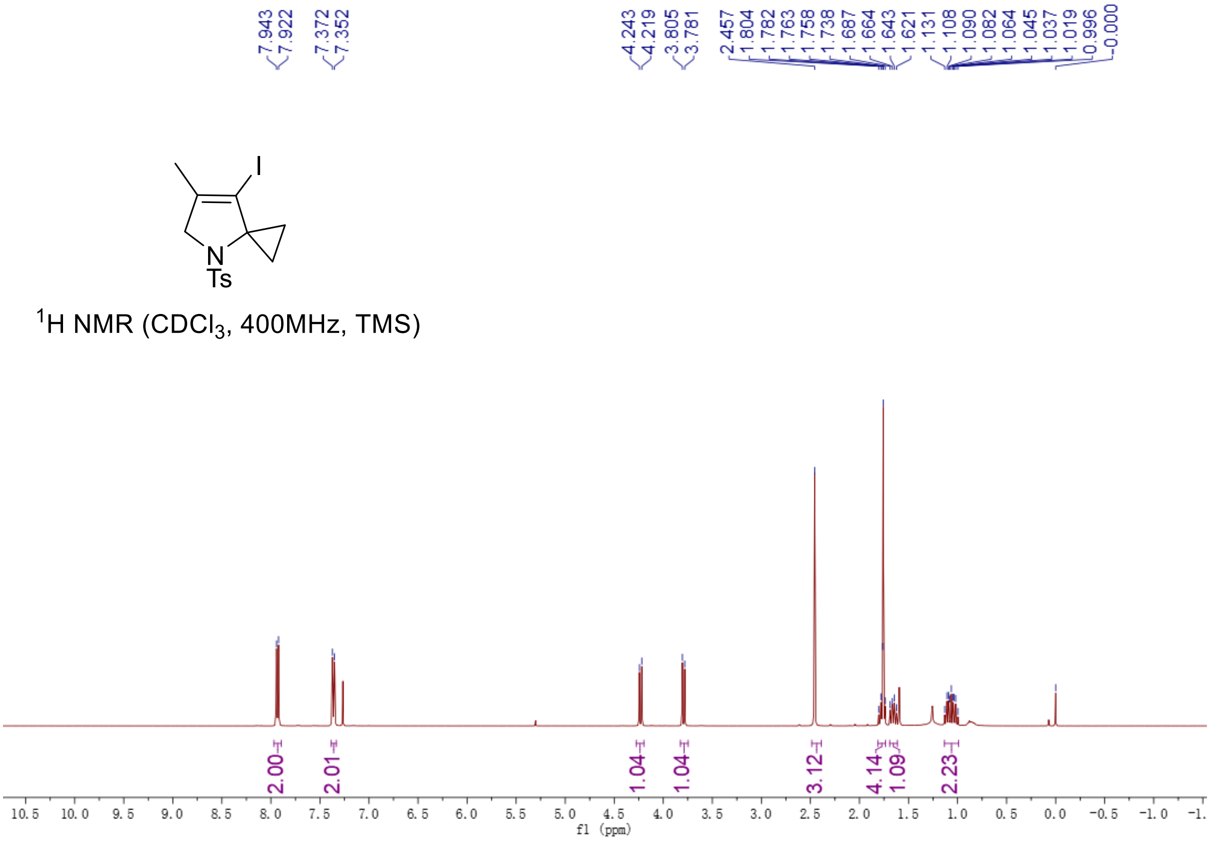

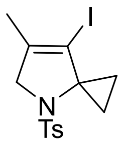

^{13}C NMR ($CDCl_3$, 100MHz, TMS)

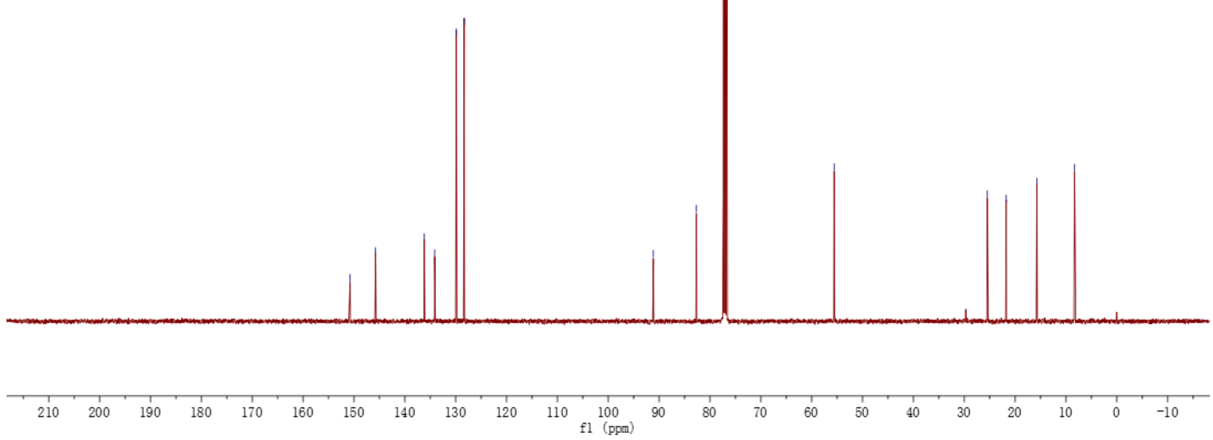

9. Spectroscopic data of products 4.

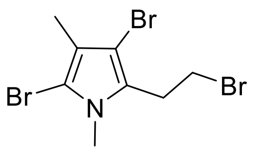

2,4-dibromo-5-(2-bromoethyl)-1,3-dimethyl-1H-pyrrole (4a)

A colorless oil, 21.6 mg, 60% yield. ^{1}H NMR ($CDCl_3$, TMS, 400 MHz) δ 3.55 (s, 3H), 3.42 (t, *J* = 7.8 Hz, 2H), 3.19 (t, *J* = 7.8 Hz, 2H), 1.99 (s, 3H). ^{13}C NMR ($CDCl_3$, TMS, 100 MHz) δ 128.1, 117.4, 101.2, 98.1, 33.4, 29.7, 29.6, 11.6. IR (Neat) ν 731, 757, 909, 943, 1165, 1348, 1530, 2922 cm^{-1}. HRMS (ESI) calcd. for $C_8H_{11}NBr_3$ (M+H): 360.8240, Found: 360.8343.

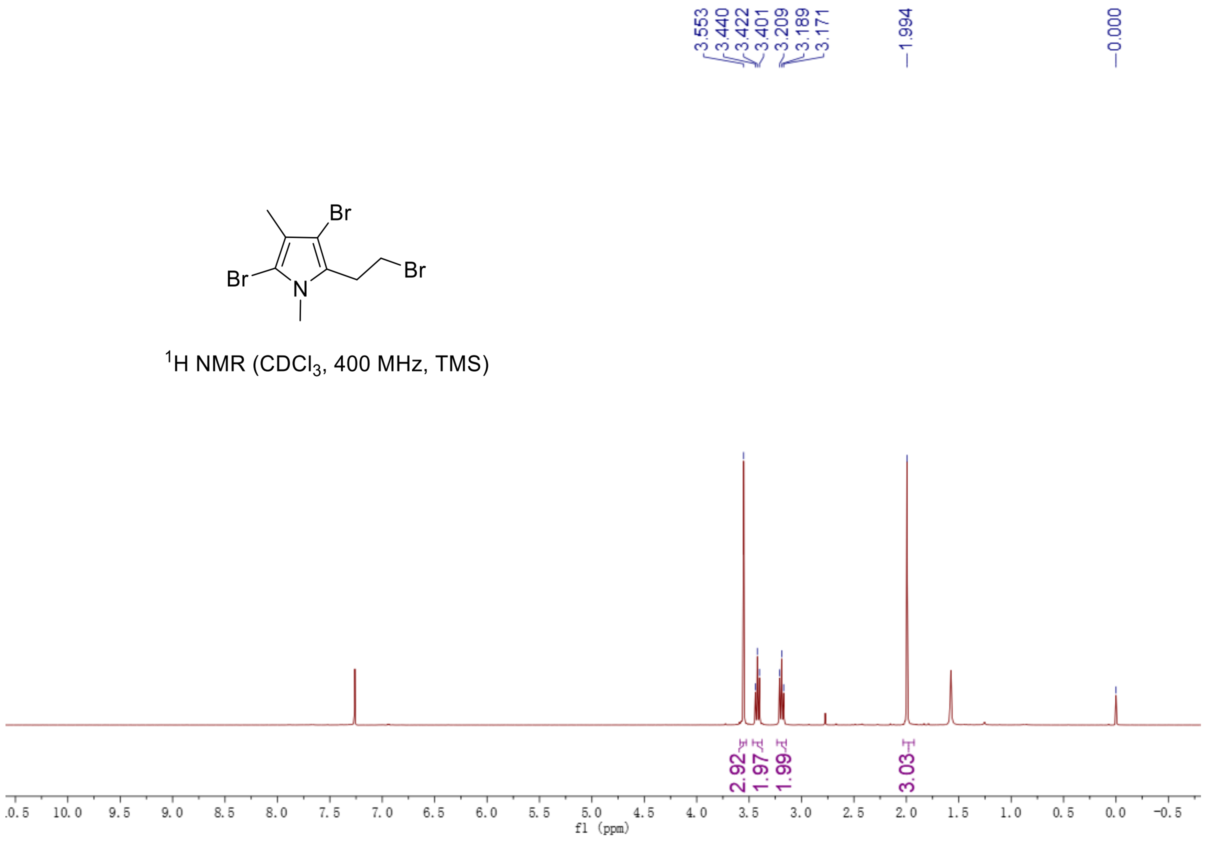

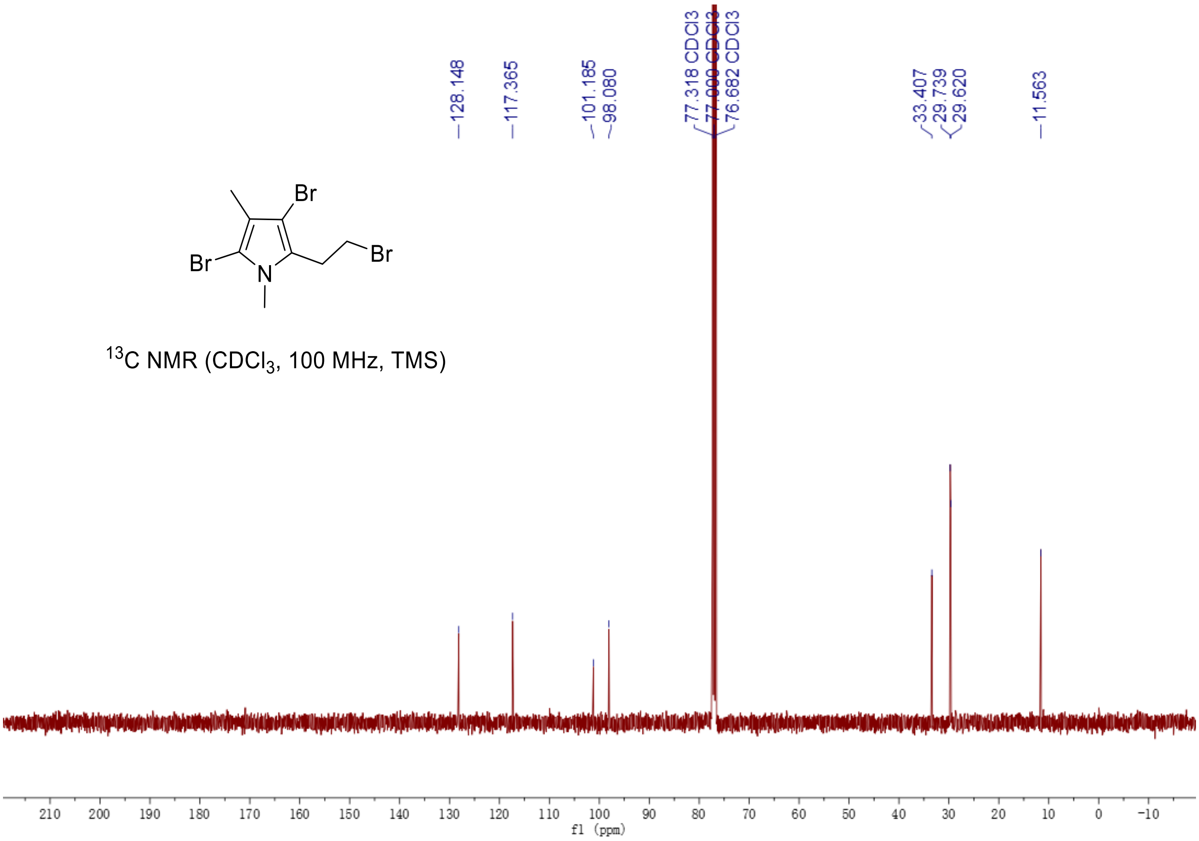

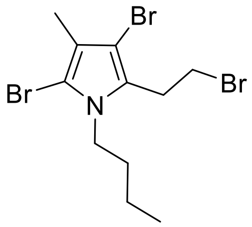

2,4-dibromo-5-(2-bromoethyl)-1-butyl-3-methyl-1H-pyrrole (4b)

A colorless oil, 28.1 mg, 70% yield. ^{1}H NMR ($CDCl_3$, TMS, 400 MHz) δ 3.88 (t, J = 7.8 Hz, 2H), 3.42 (t, J = 8.0 Hz, 2H), 3.16 (t, J = 8.0 Hz, 2H), 1.99 (s, 3H), 1.65 - 1.56 (m, 2H), 1.43 - 1.29 (m, 2H), 0.96 (t, J = 7.4 Hz, 3H). ^{13}C NMR $CDCl_3$, TMS, 100 MHz) δ 127.5, 117.5, 100.3, 98.2, 46.3, 33.3, 29.8, 29.6, 19.9, 13.8, 11.5. IR (Neat) ν 762, 878, 1165, 1352, 1453, 1524, 2959 cm^{-1}. HRMS (ESI) calcd. for $C_{11}H_{17}NBr_3$ (M+H): 403.8870, Found: 403.9716.

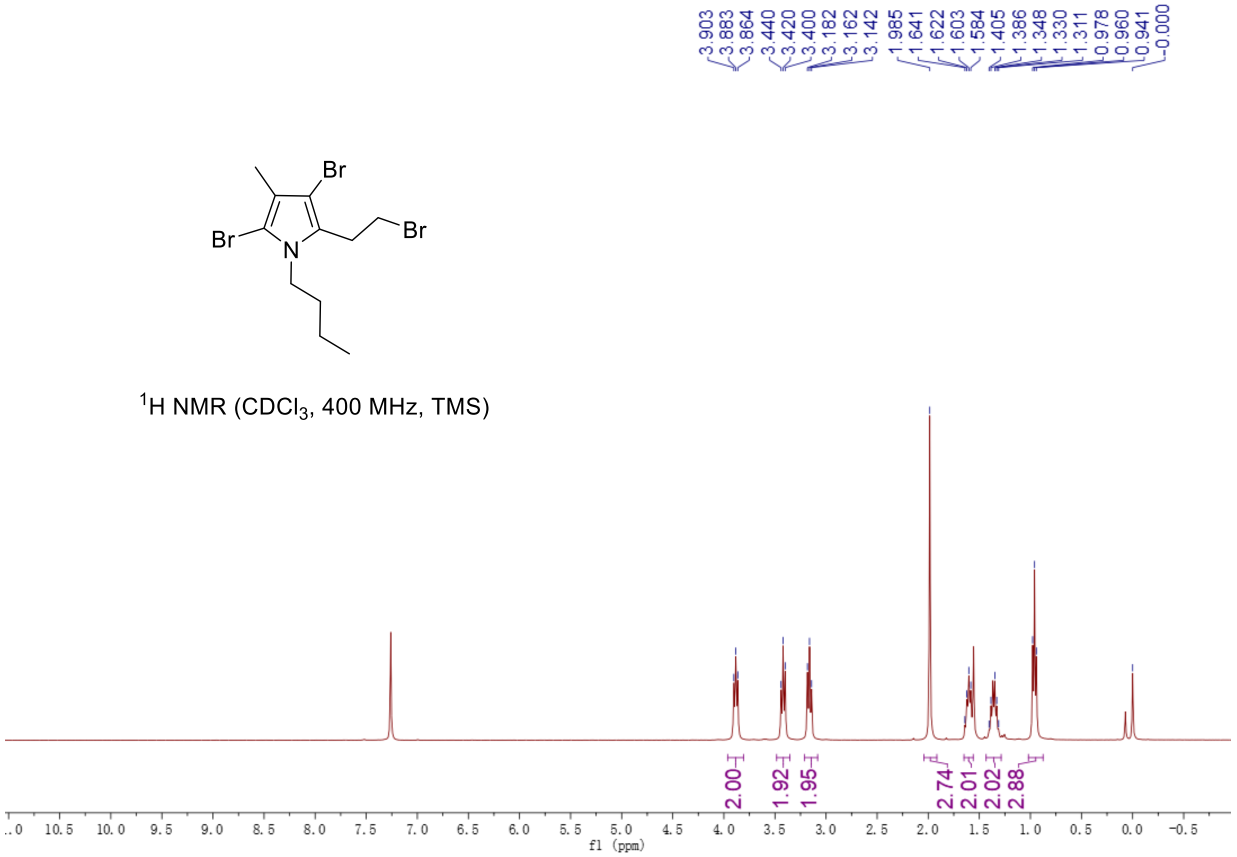

^{1}H NMR ($CDCl_3$, 400 MHz, TMS)

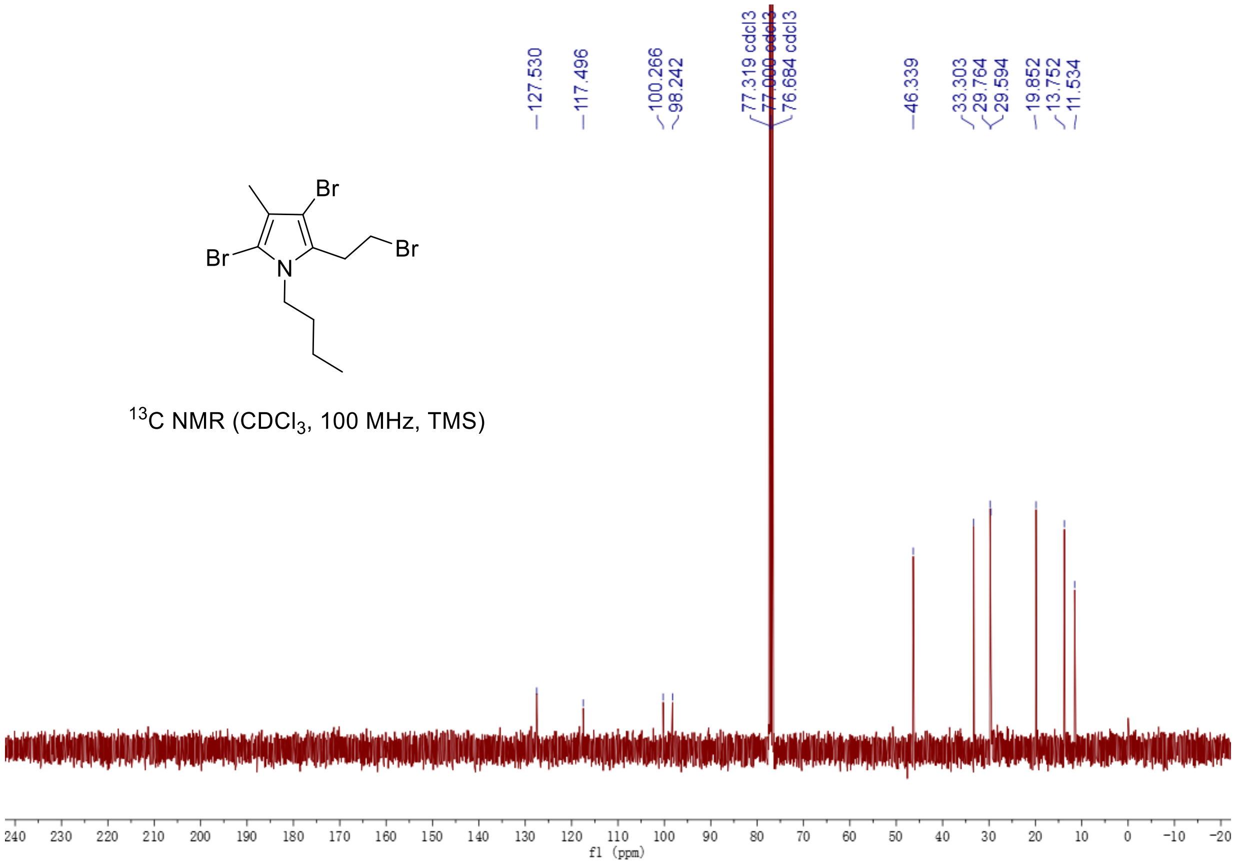

^{13}C NMR ($CDCl_3$, 100 MHz, TMS)

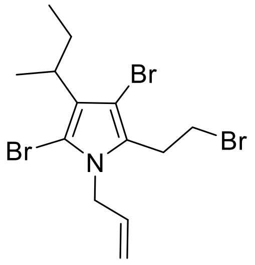

1-allyl-2,4-dibromo-5-(2-bromoethyl)-3-(sec-butyl)-1H-pyrrole (4c)

A colorless oil, 29.1 mg, 68% yield. ^{1}H NMR ($CDCl_3$, TMS, 400 MHz) δ 5.93 - 5.79 (m, 1H), 5.17 (d, *J* = 10.4 Hz, 1H), 4.74 (d, *J* = 17.1 Hz, 1H), 4.57 (s, 2H), 3.40 (t, *J* = 8.1 Hz, 2H), 3.13 (t, *J* = 8.1 Hz, 2H), 2.83 - 2.68 (m, 1H), 1.90 - 1.73 (m, 1H), 1.66 - 1.56 (m, 1H), 1.27 (d, *J* = 7.2 Hz, 3H), 0.79 (t, *J* = 7.4 Hz, 3H). ^{13}C NMR ($CDCl_3$, TMS, 100 MHz) δ 133.2, 128.6, 124.4, 116.5, 100.4, 97.1, 48.2, 34.5, 29.7, 29.6, 28.2, 19.3, 12.5. IR (Neat) ν 728, 905, 1147, 1376, 2918, 2950 cm^{-1}. HRMS (ESI) calcd. for $C_{13}H_{19}Br_3N$ (M+H): 429.9026, Found: 429.9027.

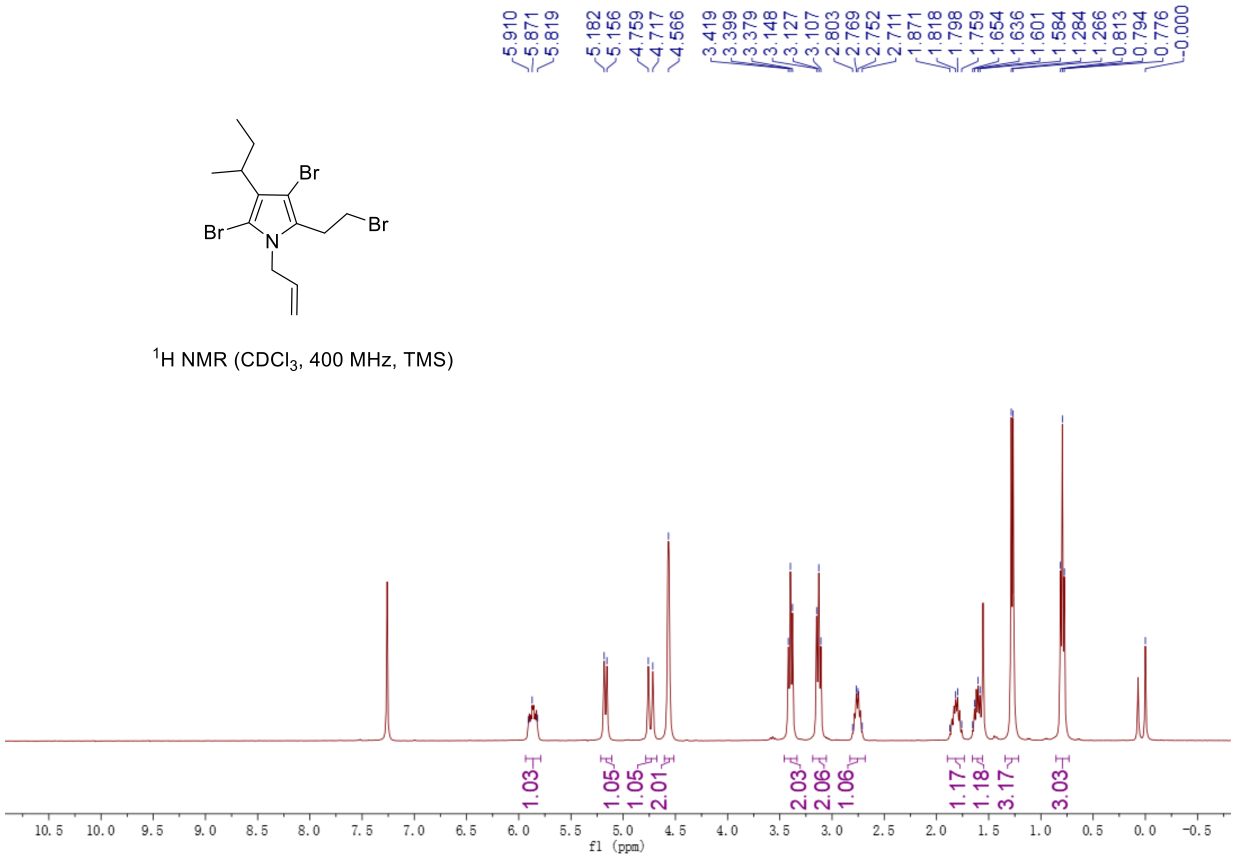

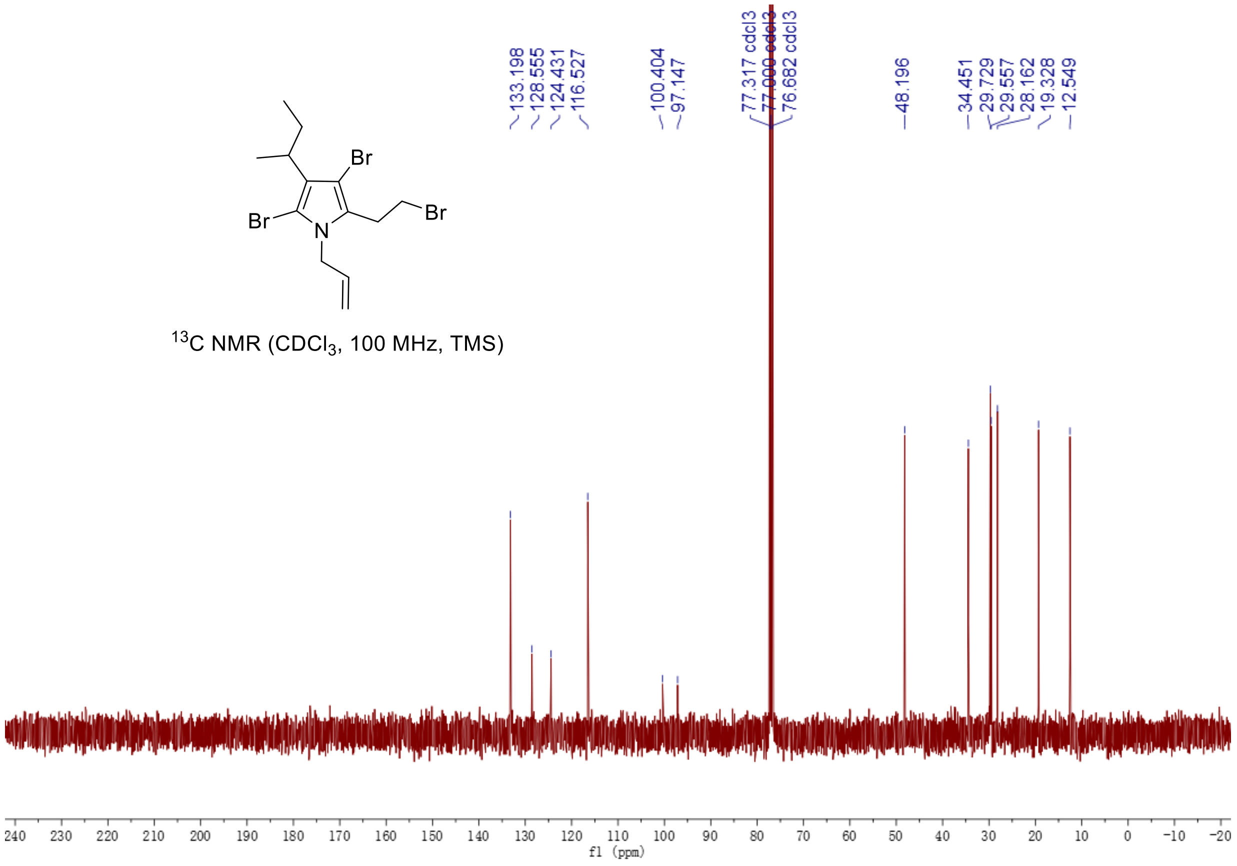

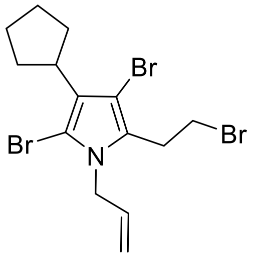

1-allyl-2,4-dibromo-5-(2-bromoethyl)-3-cyclopentyl-1H-pyrrole (4d)

A colorless oil, 28.6 mg, 65% yield. ^{1}H NMR (CDCl$_3$, TMS, 400 MHz) δ 5.94 - 5.78 (m, 1H), 5.18 (d, J = 10.1 Hz, 1H), 4.84 (d, J = 17.1 Hz, 1H), 4.56 (s, 2H), 3.45 - 3.34 (m, 2H), 3.18 - 3.09 (m, 2H), 3.08 - 2.98 (m, 1H), 1.95 - 1.77 (m, 6H), 1.72 - 1.56 (m, 2H). ^{13}C NMR (CDCl$_3$, TMS, 100 MHz) δ 133.2, 128.7, 123.3, 116.9, 100.3, 97.2, 48.3, 37.9, 31.3, 29.8, 29.4, 26.2. IR (Acetone) ν 759, 1023, 1120, 1433, 2921, 2930 cm^{-1}. HRMS (ESI) calcd. for $C_{14}H_{19}NBr_3$ (M+H): 441.9023, Found: 441.9027.

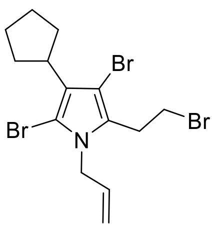

^{1}H NMR ($CDCl_3$, 400 MHz, TMS)

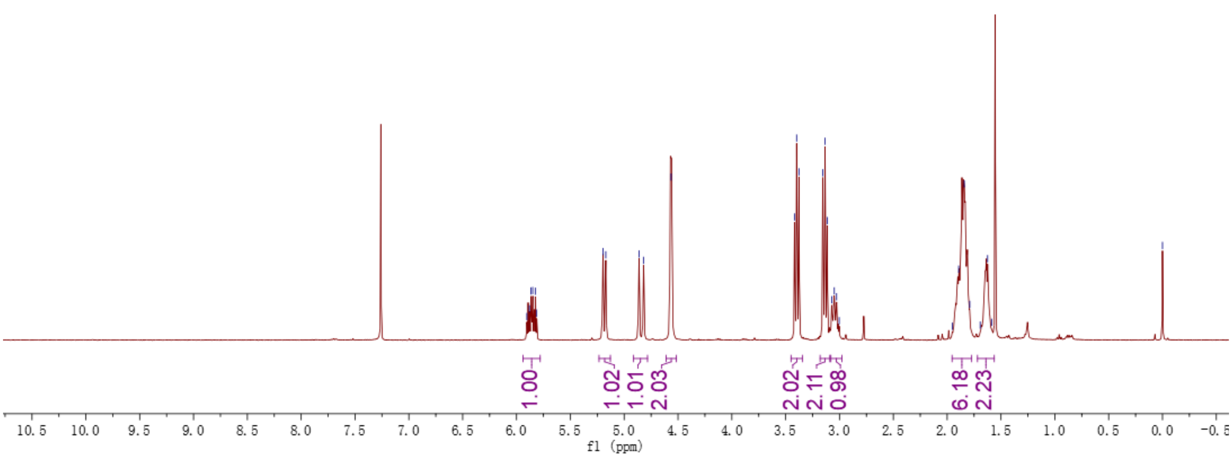

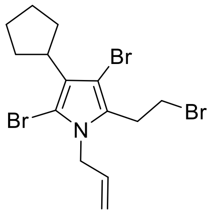

^{13}C NMR ($CDCl_3$, 100 MHz, TMS)

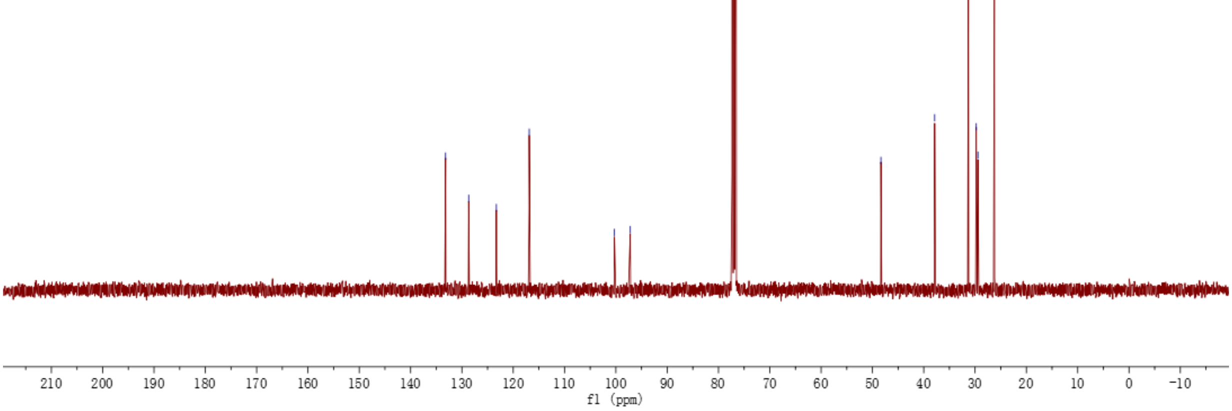

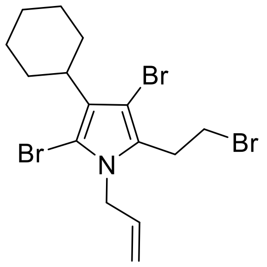

1-allyl-2,4-dibromo-5-(2-bromoethyl)-3-cyclohexyl-1H-pyrrole (4e)

A colorless oil, 26.3 mg, 58% yield. ^{1}H NMR ($CDCl_3$, TMS, 400 MHz) δ 5.94 - 5.73 (m, 1H), 5.15 (d, *J* = 10.3 Hz, 1H), 4.77 (d, *J* = 17.1 Hz, 1H), 4.54 (s, 2H), 3.37 (t, *J* = 8.1 Hz, 2H), 3.11 (t, *J* = 8.1 Hz, 2H), 2.69 - 2.52 (m, 1H), 1.98 - 1.84 (m, 2H), 1.83 - 1.73 (m, 2H), 1.72 - 1.64 (m, 1H), 1.63 - 1.55 (m, 2H), 1.39 - 1.16 (m, 3H). ^{13}C NMR ($CDCl_3$, TMS, 100 MHz) δ 133.2, 128.5, 125.3, 116.8, 99.8, 97.1, 48.3, 37.7, 30.9, 29.8, 29.5, 27.0, 26.0. IR (Neat) ν 699, 1021, 1129, 1448, 2931, 2950, 2977 cm^{-1}. HRMS (ESI) calcd. for $C_{15}H_{22}NBr_3$ (M+H): 453.9204, Found: 453.9184.

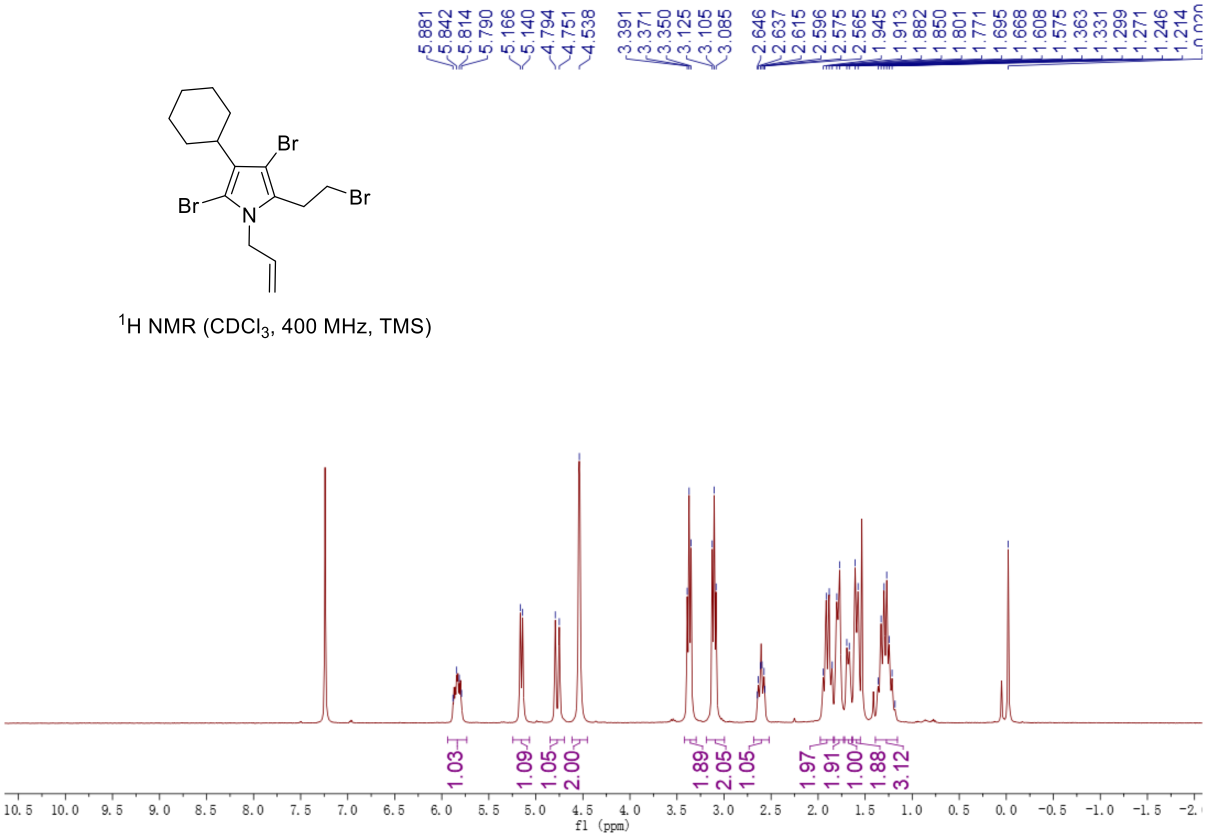

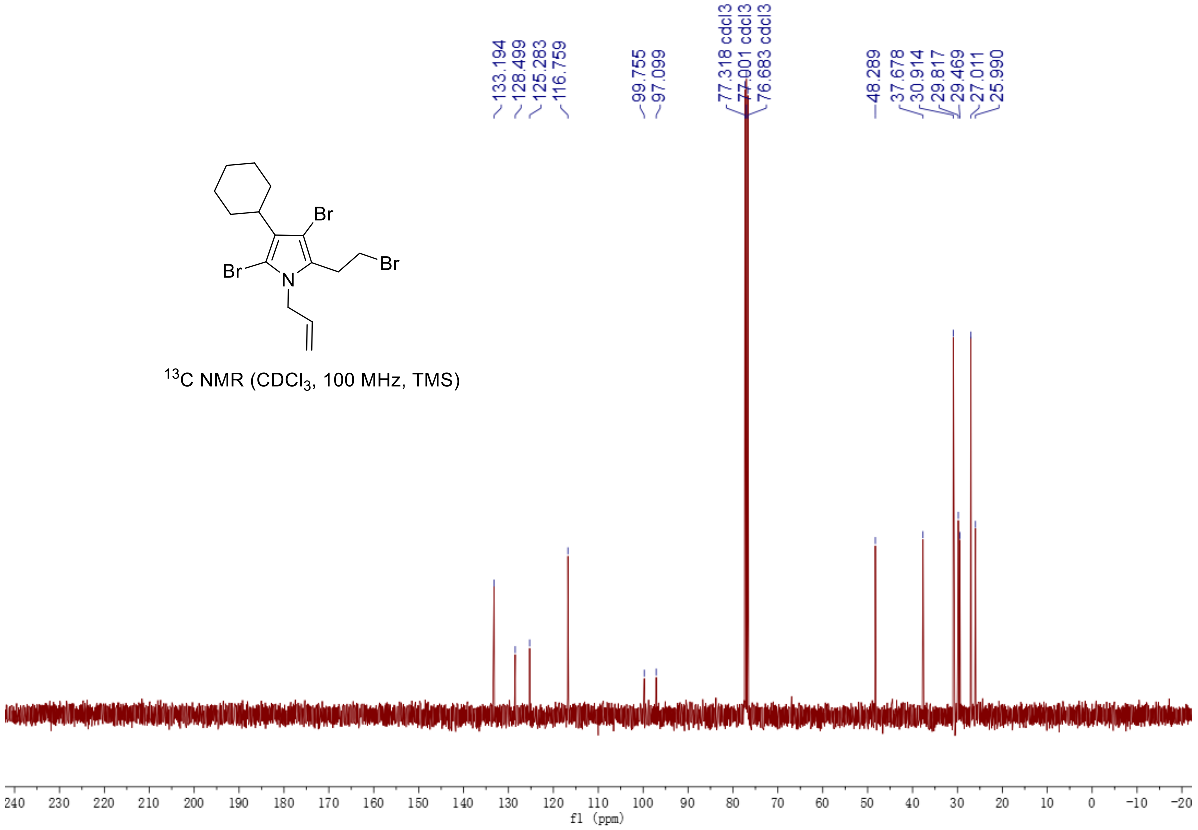

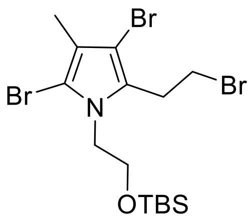

2,4-dibromo-5-(2-bromoethyl)-1-(2-((tert-butyldimethylsilyl)oxy)ethyl)-3-methyl-1H-pyrrole (4f)

A colorless oil, 36.8 mg, 73% yield. ^{1}H NMR ($CDCl_3$, TMS, 400 MHz) δ 4.04 (t, *J* = 5.7 Hz, 2H), 3.77 (t, *J* = 5.8 Hz, 2H), 3.43 (t, *J* = 7.8 Hz, 2H), 3.24 (t, *J* = 7.9 Hz, 2H), 1.99 (s, 3H), 0.84 (s, 9H), -0.05 (s, 6H). ^{13}C NMR ($CDCl_3$, TMS, 100 MHz) δ 129.1, 117.9, 99.8, 98.3, 62.4, 48.4, 29.8, 29.7, 25.8, 18.3, 11.5, -5.7. IR (Neat) ν 732, 1025, 1146, 1188, 1308, 1345, 1705, 2956 cm^{-1}. HRMS (ESI) calcd. for $C_{15}H_{27}NOSiBr_3$ (M+H): 503.9392, Found: 503.9392.

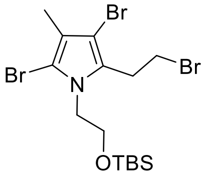

^{1}H NMR ($CDCl_3$, 400 MHz, TMS)

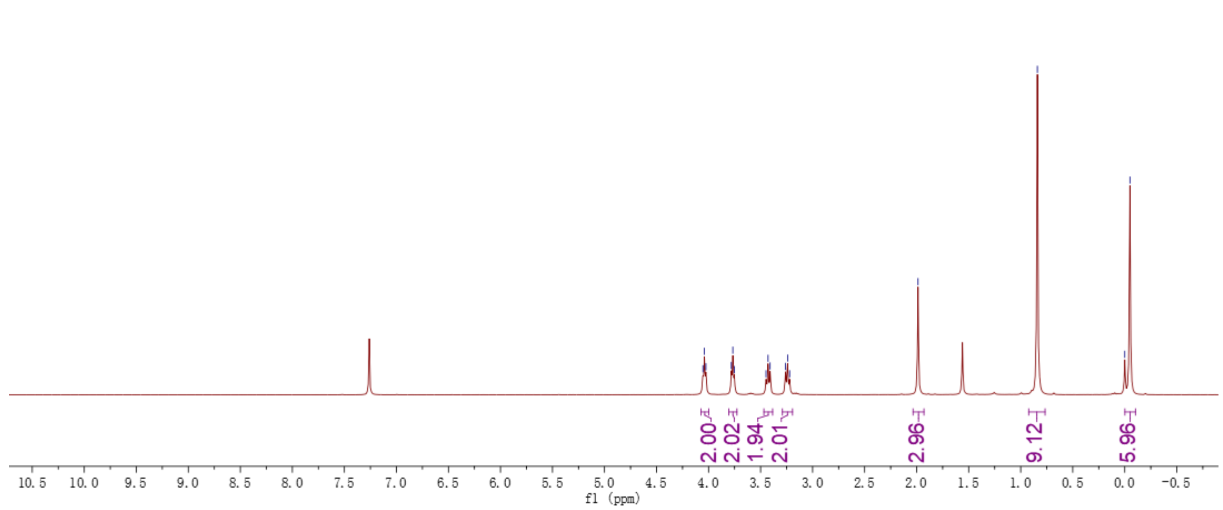

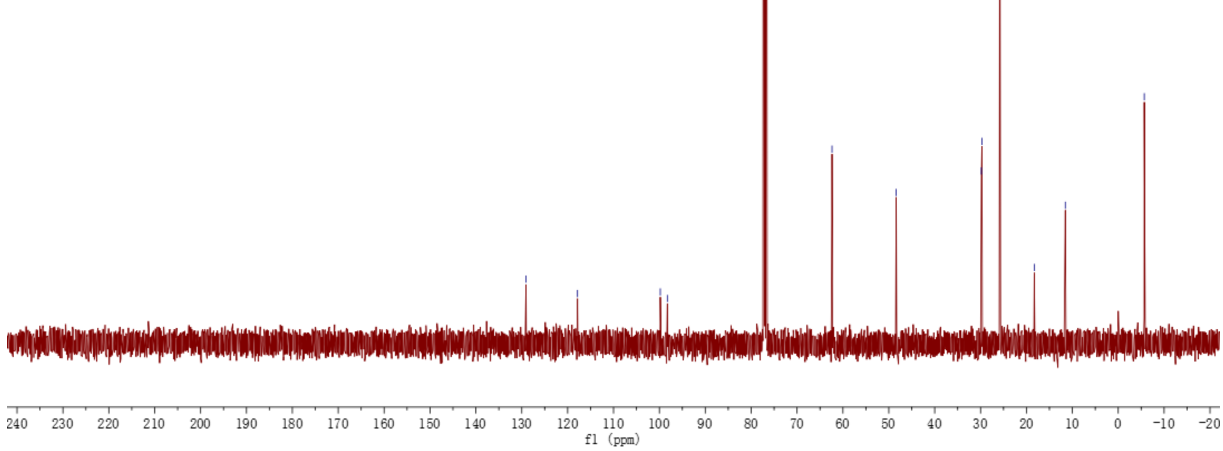

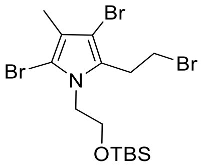

^{13}C NMR ($CDCl_3$, 100 MHz, TMS)

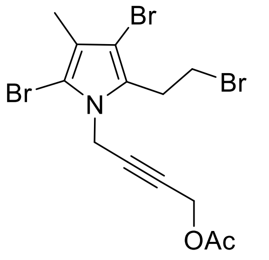

4-(2,4-dibromo-5-(2-bromoethyl)-3-methyl-1H-pyrrol-1-yl)but-2-yn-1-yl acetate (4g)

A colorless oil, 36.5 mg, 80% yield. ^{1}H NMR ($CDCl_3$, TMS, 400 MHz) δ 4.77 (s, 2H), 4.66 (s, 2H), 3.50 (t, J = 7.9 Hz, 2H), 3.24 (t, J = 7.9 Hz, 2H), 2.10 (s, 3H), 2.00 (s, 3H). ^{13}C NMR ($CDCl_3$, TMS, 100 MHz) δ 170.1, 128.1, 118.4, 100.5, 99.6, 80.5, 79.1, 51.9, 36.1, 29.8, 29.5, 20.7, 11.6. IR (Acetone) ν 730, 1047, 1165, 1203, 1444, 1760 1799, 2963 cm^{-1}. HRMS (ESI) calcd. for $C_{13}H_{15}NO_2Br_3$ (M+H): 455.8632, Found: 455.8626.

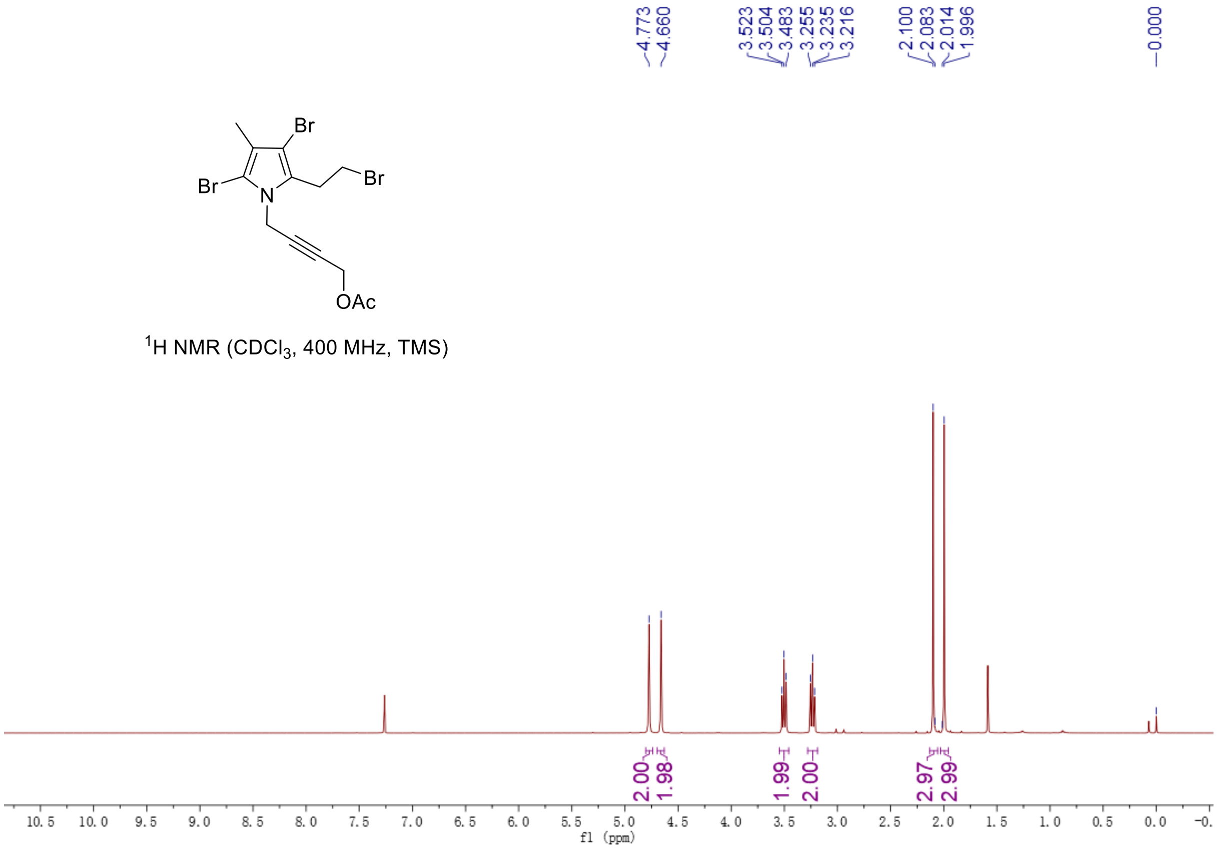

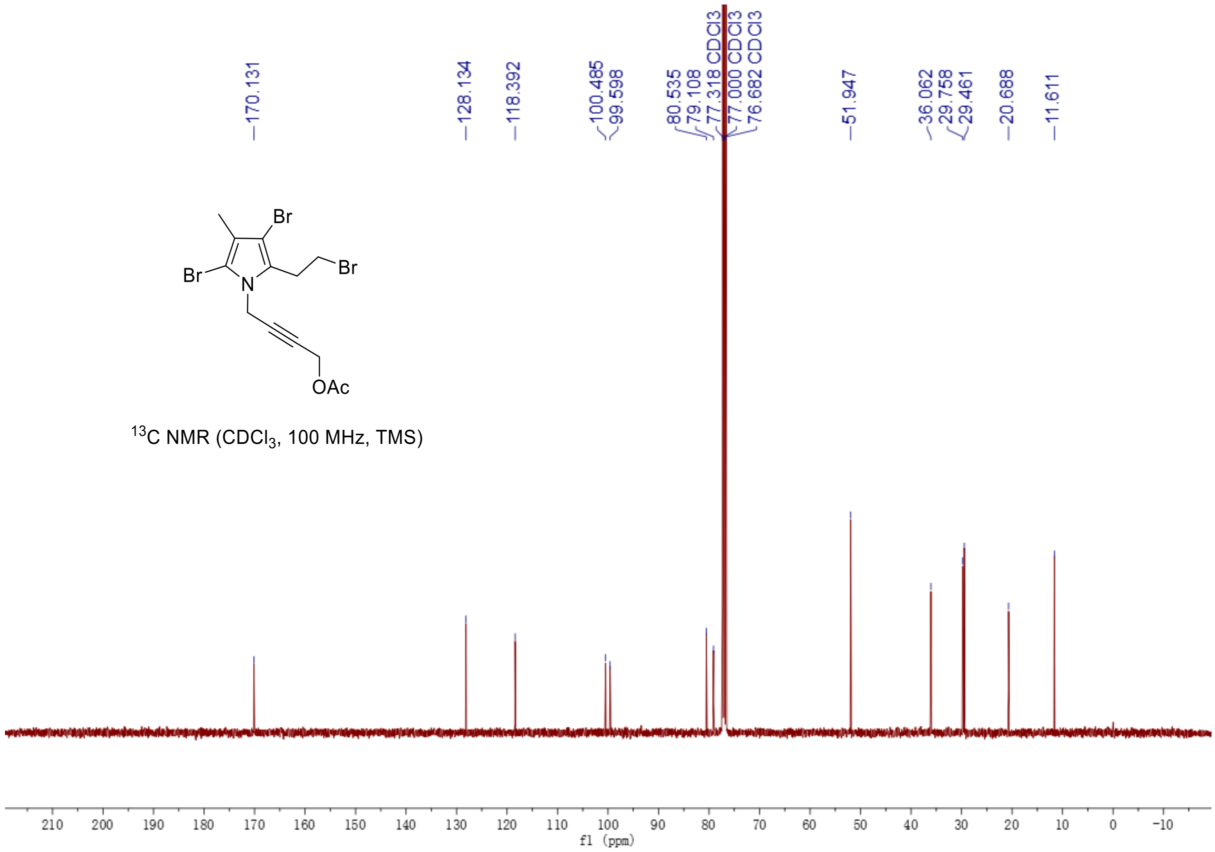

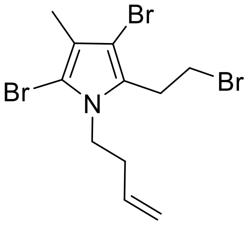

2,4-dibromo-5-(2-bromoethyl)-1-(but-3-en-1-yl)-3-methyl-1H-pyrrole (4h)

A colorless oil, 31.2 mg, 78% yield. ^{1}H NMR ($CDCl_3$, TMS, 400 MHz) δ 5.82 - 5.65 (m, 1H), 5.17 - 5.04 (m, 2H), 3.95 (t, *J* = 7.7 Hz, 2H), 3.42 (t, *J* = 8.0 Hz, 2H), 3.15 (t, *J* = 8.0 Hz, 2H), 2.37 (q, *J* = 7.7 Hz, 2H), 1.98 (s, 3H). ^{13}C NMR ($CDCl_3$, TMS, 100 MHz) δ 133.5, 127.7, 117.9, 117.8, 100.1, 98.4, 45.9, 35.2, 29.8, 29.6, 11.5. IR (Acetone) ν 2936, 2924, 2853, 1611, 1589, 1497, 1457, 1356, 1310, 1272, 1250, 1230, 1214, 1150, 1058, 1029, 959, 823, 788, 744, 700 cm^{-1}. HRMS (ESI) calcd. for $C_{13}H_{21}Br_3N$ (M+H): 427.9224, Found: 427.9231.

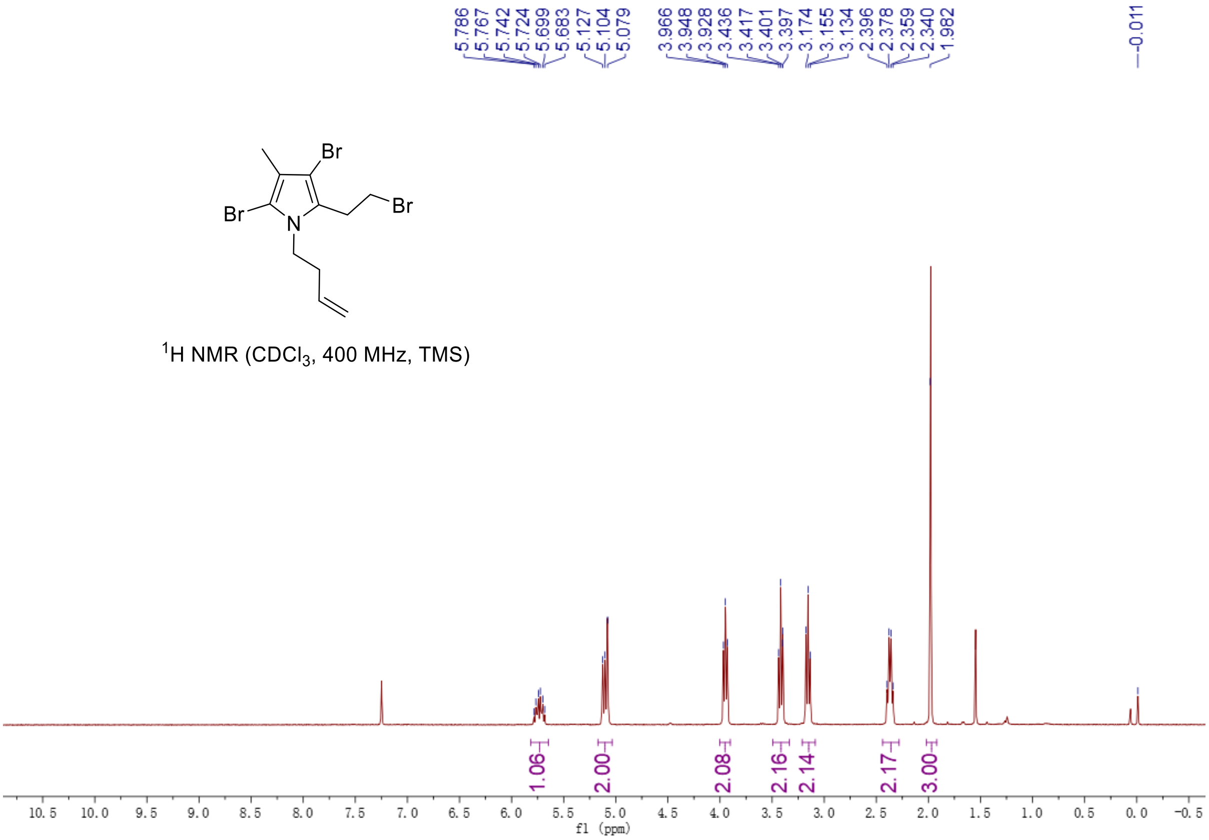

^{1}H NMR ($CDCl_3$, 400 MHz, TMS)

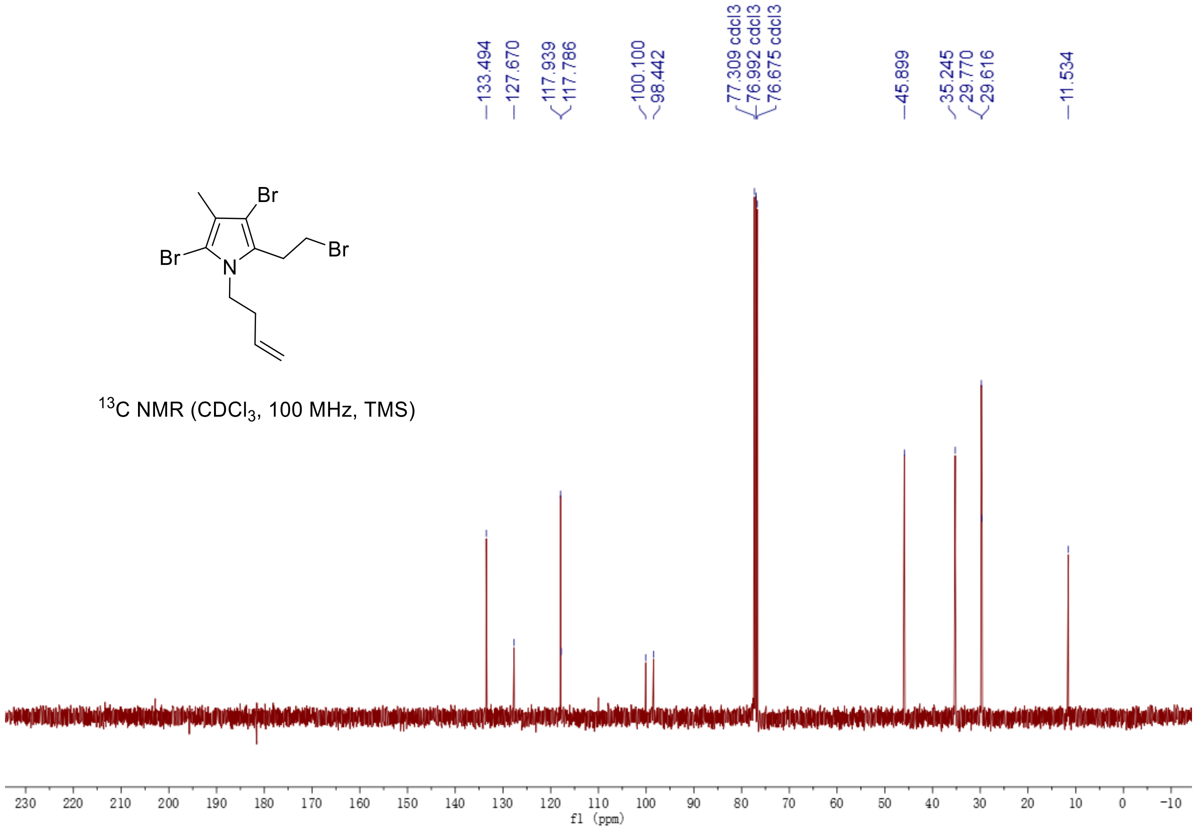

^{13}C NMR ($CDCl_3$, 100 MHz, TMS)

9. Spectroscopic data of products 6.

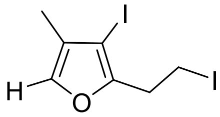

3-iodo-2-(2-iodoethyl)-4-methylfuran (6a)

A colorless oil, 27.5 mg, 76% yield. ^{1}H NMR ($CDCl_3$, TMS, 400 MHz) δ 7.20 (s, 1H), 3.38 - 3.29 (m, 2H), 3.27 - 3.20 (m, 2H), 1.94 (s, 3H). ^{13}C NMR ($CDCl_3$, TMS, 100 MHz) δ 154.3, 138.1, 123.8, 71.4, 32.3, 11.3, 0.7. IR (Acetone) ν 2936, 2924, 2853, 1611, 1589, 1497, 1457, 1356, 1310, 1272, 1250, 1230, 1214, 1150, 1058, 1029, 959, 823, 788, 744, 700 cm^{-1}. HRMS (ESI) calcd. for $C_7H_8I_2ONa$ (M+Na): 384.8562, Found: 384.8673.

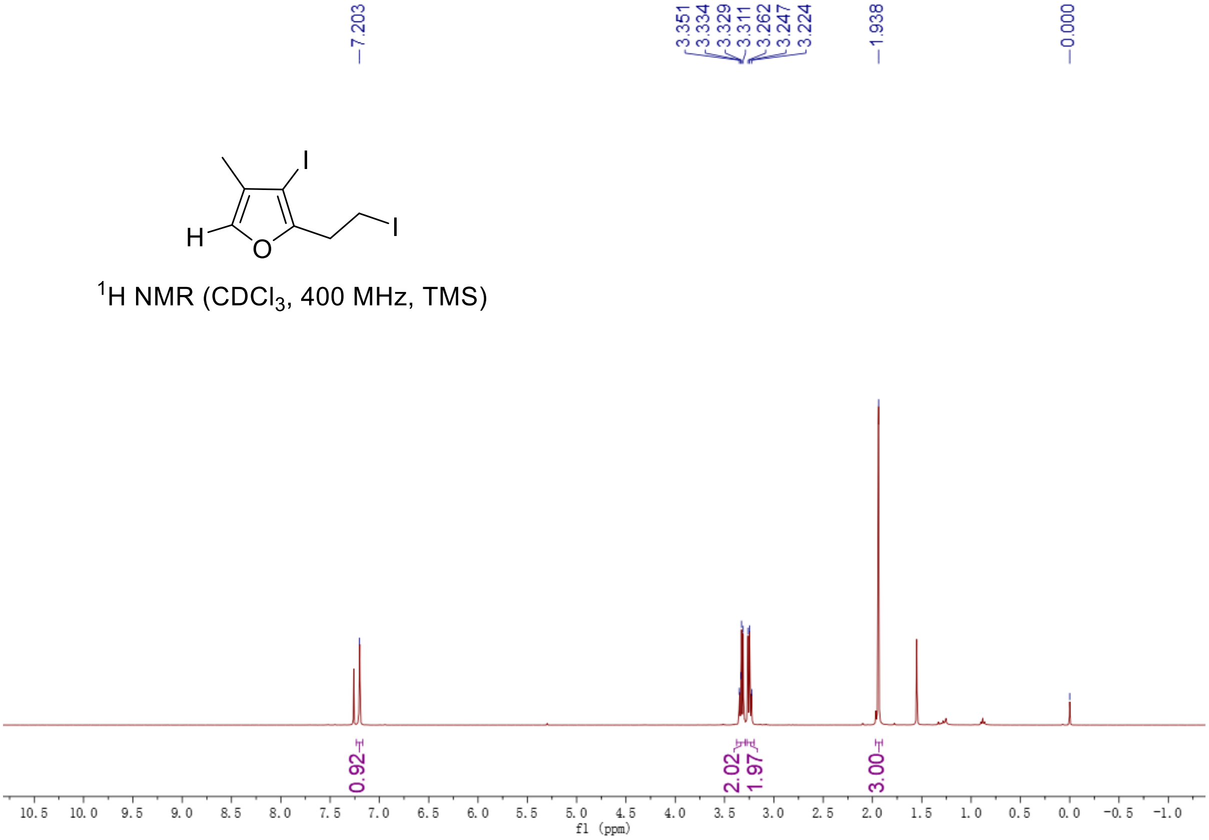

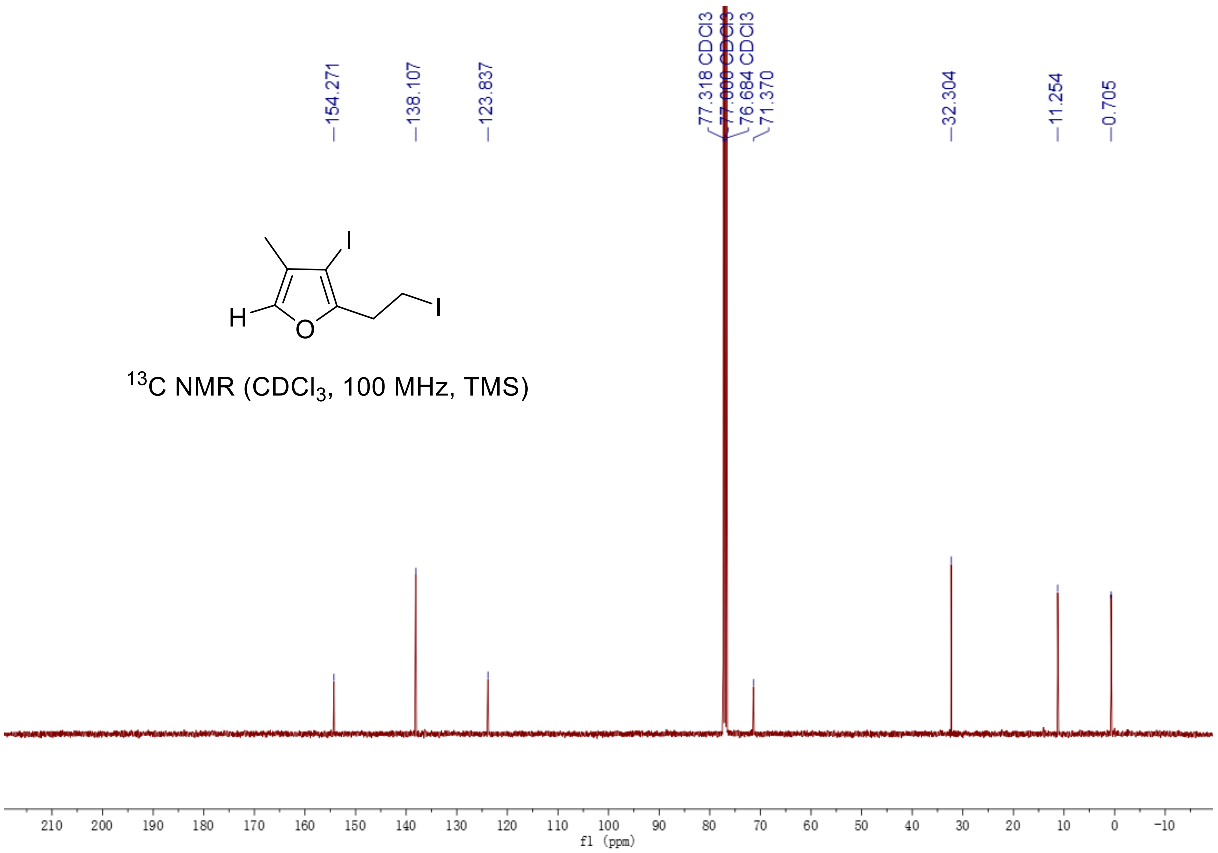

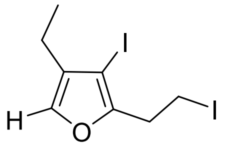

4-ethyl-3-iodo-2-(2-iodoethyl)furan (6b)

A colorless oil, 27.1 mg, 72% yield. ^{1}H NMR ($CDCl_3$, TMS, 600 MHz) δ 7.16 (s, 1H), 3.35 - 3.31 (m, 2H), 3.27 - 3.23 (m, 2H), 2.33 (q, J = 7.5 Hz, 2H), 1.18 (t, J = 7.5 Hz, 3H). ^{13}C NMR ($CDCl_3$, TMS, 150 MHz) δ 154.3, 137.7, 129.9, 70.1, 32.2, 29.7, 19.8, 13.2, 0.7. IR (Acetone) ν 2972, 2929, 2892, 1735, 1484, 1247, 1086, 1044, 879 cm^{-1}. HRMS (ESI) calcd. for $C_8H_{11}I_2O$ (M+H): 376.8899, Found: 376.8912.

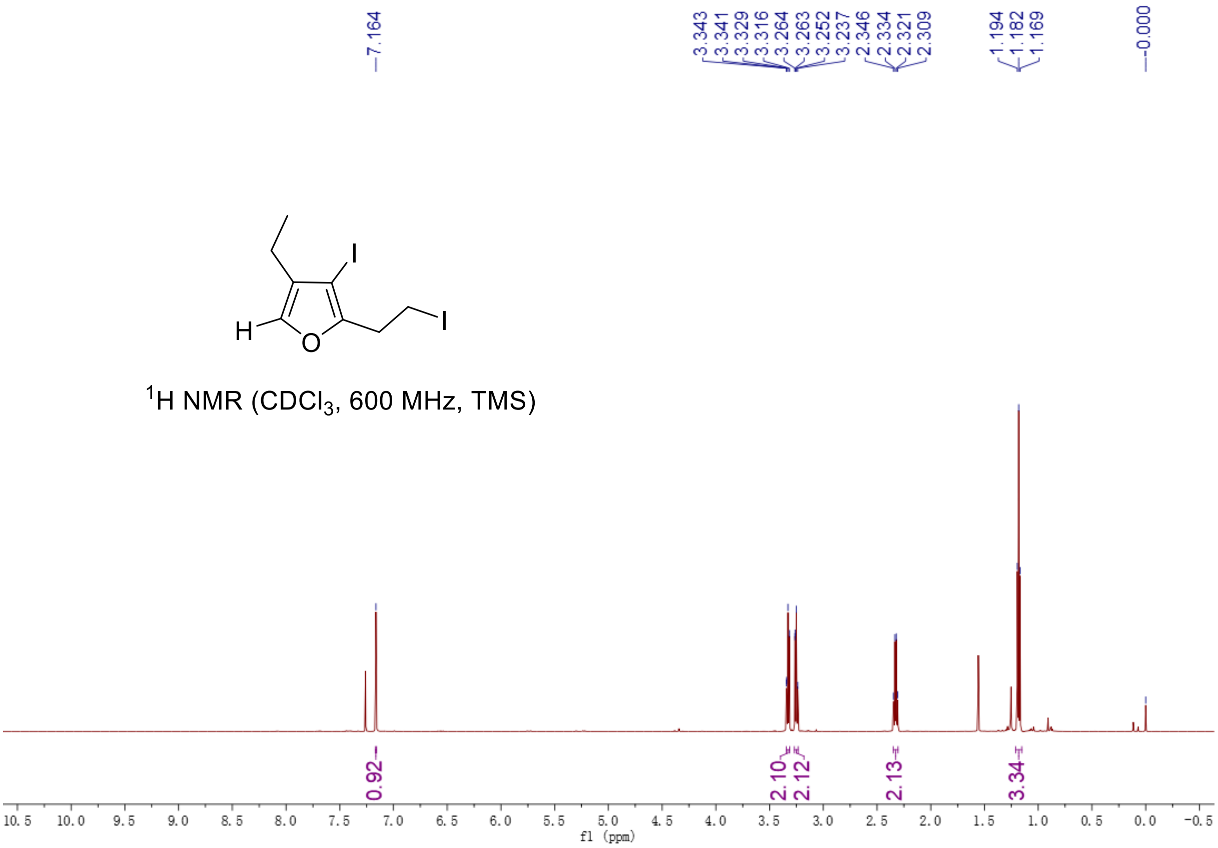
7.164
3.343
3.341
3.329
3.316
3.264
3.263
3.252
3.237
2.346
2.334
2.321
2.309
1.194
1.182
1.169
0.000
H
O
I
I
^{1}H NMR ($CDCl_3$, 600 MHz, TMS)
0.92
2.10
2.12
2.13
3.34
f1 (ppm)

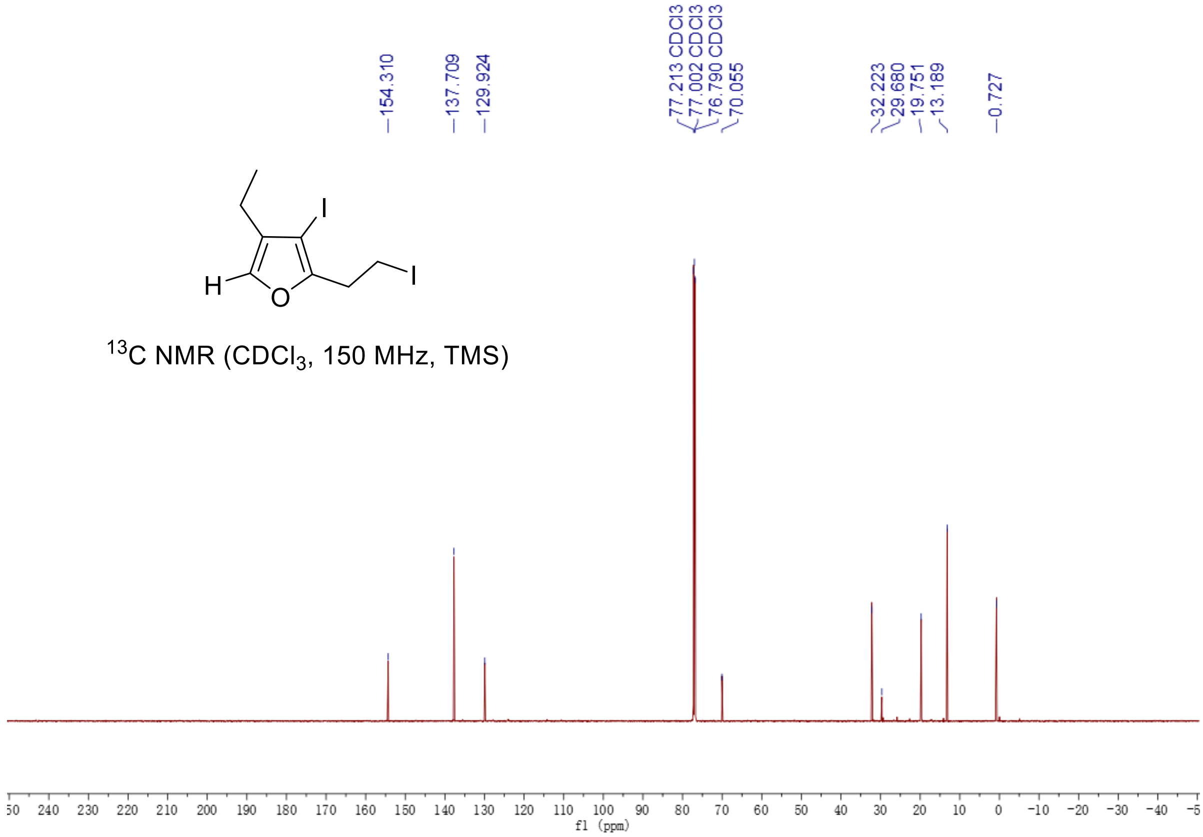
154.310
137.709
129.924
77.213 CDCl3
77.002 CDCl3
76.790 CDCl3
70.055
32.223
29.680
19.751
13.189
0.727
H
O
I
I
^{13}C NMR ($CDCl_3$, 150 MHz, TMS)
f1 (ppm)

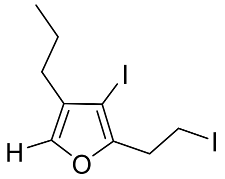

3-iodo-2-(2-iodoethyl)-4-propylfuran (6c)

A colorless oil, 27.3 mg, 70% yield ^{1}H NMR ($CDCl_3$, TMS, 600 MHz) δ 7.16 (s, 1H), 3.35 - 3.31 (m, 2H), 3.27 - 3.23 (m, 2H), 2.28 (t, *J* = 7.6 Hz, 2H), 1.61 - 1.56 (m, 2H), 0.96 (t, *J* = 7.3 Hz, 3H). ^{13}C NMR ($CDCl_3$, TMS, 150 MHz) δ 154.2, 138.1, 128.0, 70.4, 32.2, 28.2, 22.0, 13.8, 0.8. IR (Acetone) ν 2958, 2890, 1735, 1462, 1445, 1362, 1342, 1243, 1035, 904, 811 cm^{-1}. HRMS (ESI) calcd. for $C_9H_{12}I_2ONa$ (M+Na): 412.8875, Found: 412.8903.

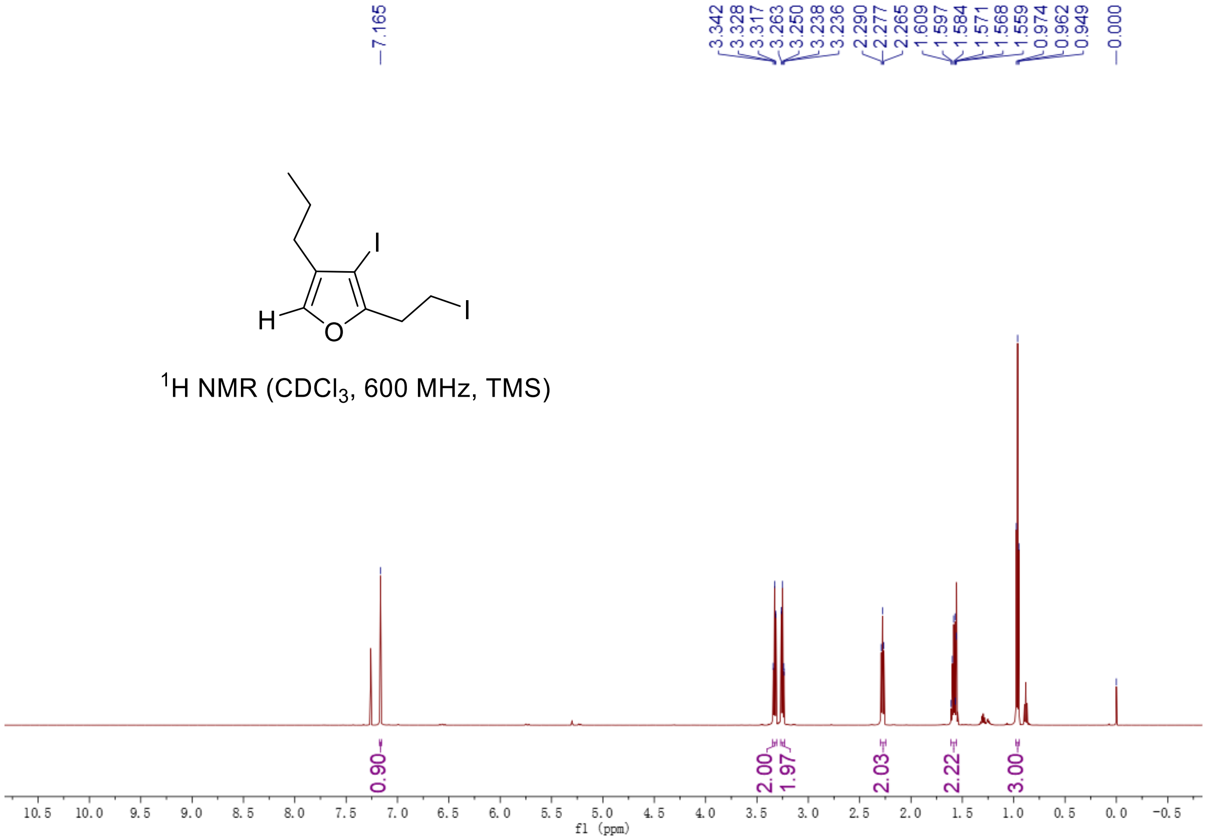

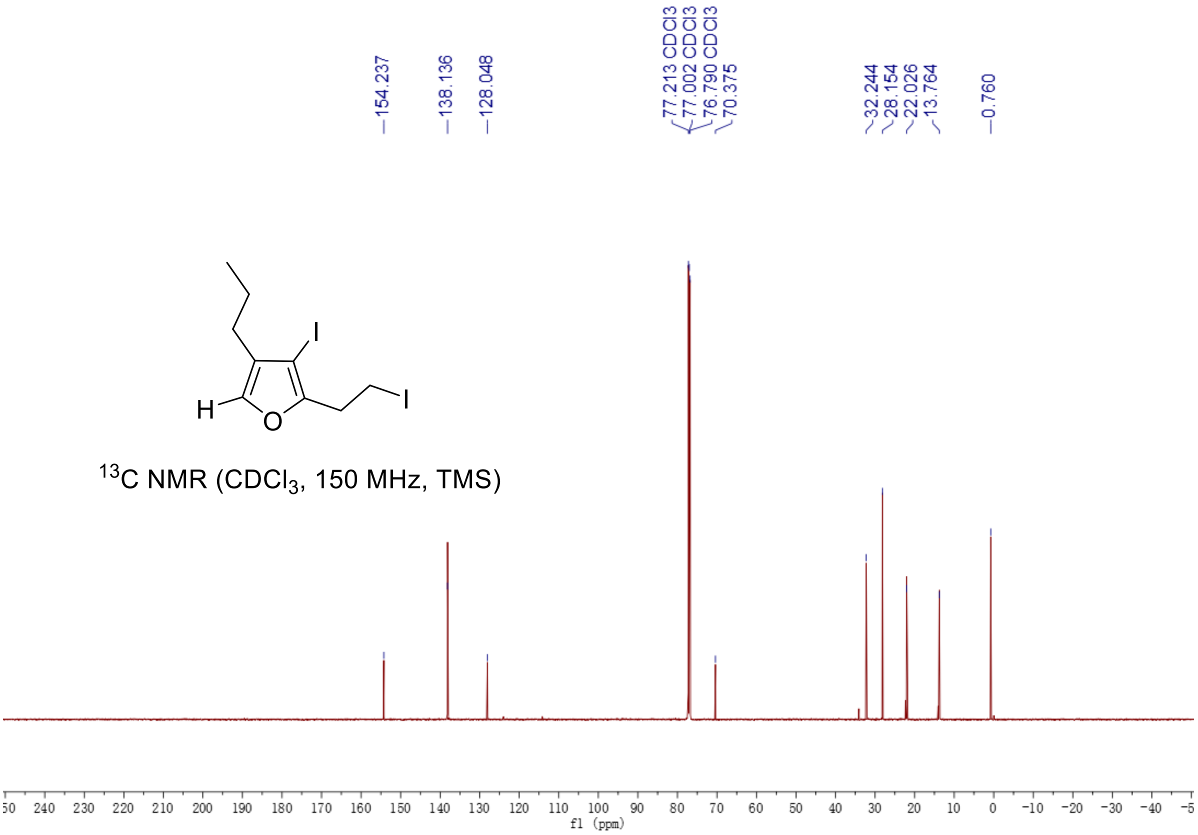

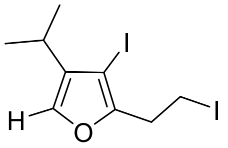

3-iodo-2-(2-iodoethyl)-4-isopropylfuran (6d)

A colorless oil, 25.7 mg, 66% yield. ^{1}H NMR ($CDCl_3$, TMS, 600 MHz) δ 7.14 (s, 1H), 3.34 - 3.31 (m, 2H), 3.27 - 3.23 (m, 2H), 2.66 - 2.60 (m, 1H), 1.22 (s, 3H), 1.20 (s, 3H). ^{13}C NMR ($CDCl_3$, TMS, 150 MHz) δ 154.4, 137.0, 134.5, 69.2, 32.3, 26.6, 22.4, 0.6. IR (Acetone) ν 2969, 2890, 1734, 1458, 1341, 1240, 1035, 896, 809 764, 736 cm^{-1}. HRMS (ESI) calcd. for $C_9H_{13}I_2O$ (M+H): 390.9065, Found: 390.9143.

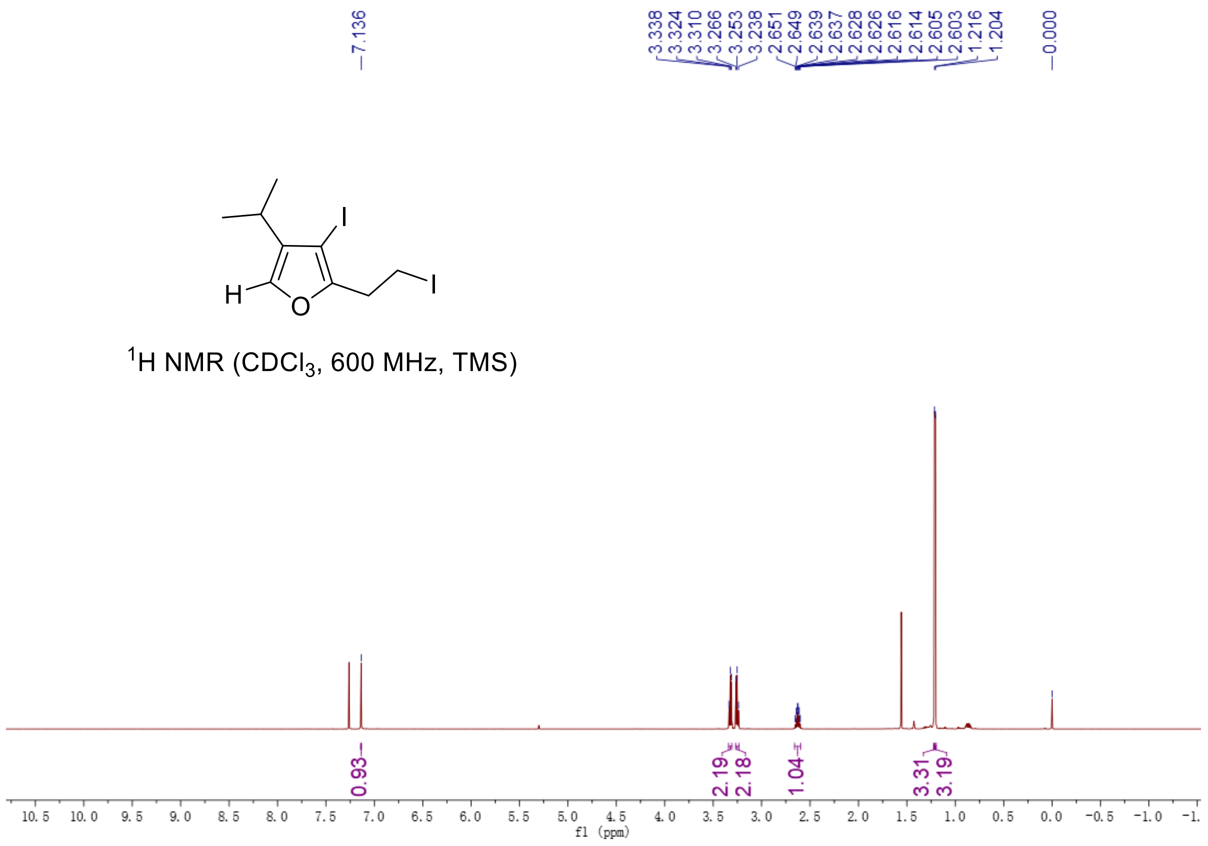
7.136
3.338
3.324
3.310
3.266
3.253
3.238
2.651
2.649
2.639
2.637
2.628
2.626
2.616
2.614
2.605
2.603
1.216
1.204
0.000
I
I
H
O
1H NMR (CDCl3, 600 MHz, TMS)
0.93
2.19
2.18
1.04
3.31
3.19
f1 (ppm)

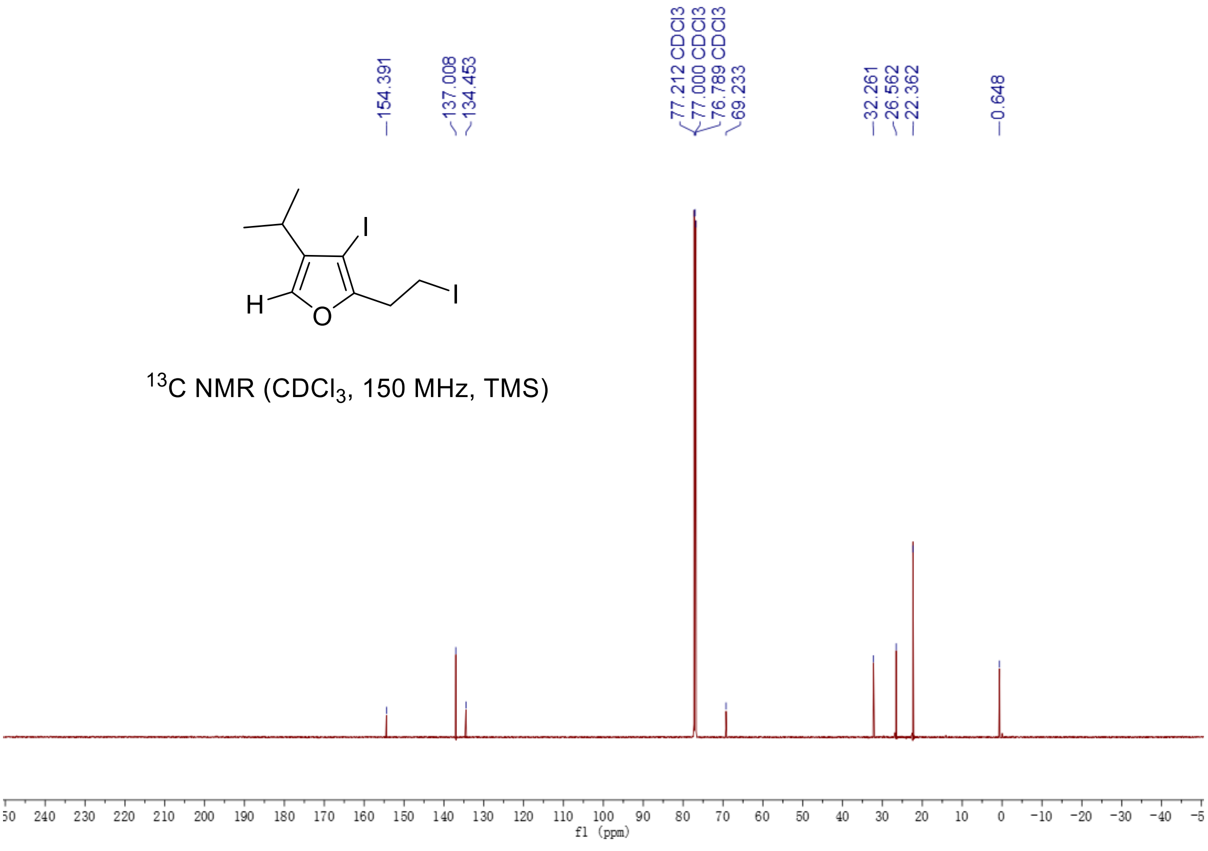
154.391
137.008
134.453
77.212 CDCl3
77.000 CDCl3
76.789 CDCl3
69.233
32.261
26.562
22.362
0.648
I
I
H
O
13C NMR (CDCl3, 150 MHz, TMS)
f1 (ppm)

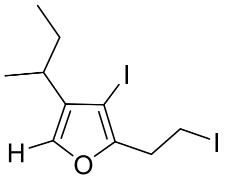

4-(sec-butyl)-3-iodo-2-(2-iodoethyl)furan (6e)

A colorless oil, 27.5 mg, 68% yield. ^{1}H NMR ($CDCl_3$, TMS, 600 MHz) δ 7.11 (s, 1H), 3.34 - 3.31 (m, 2H), 3.27 - 3.24 (m, 2H), 2.46 - 2.39 (m, 1H), 1.70 - 1.63 (m, 1H), 1.51 - 1.43 (m, 1H), 1.18 (d, J = 6.9 Hz, 3H), 0.90 (t, J = 7.4 Hz, 3H). ^{13}C NMR ($CDCl_3$, TMS, 150 MHz) δ 154.2, 137.6, 133.1, 69.7, 33.1, 32.3, 29.2, 19.7, 11.6, 0.7. IR (Acetone) ν 3021, 2960, 2919, 1735, 1456, 1445, 1361, 1342, 1243, 1035, 807, 770 cm^{-1}. HRMS (ESI) calcd. for $C_{10}H_{15}I_2O$ (M+H): 404.9212, Found: 404.9352.

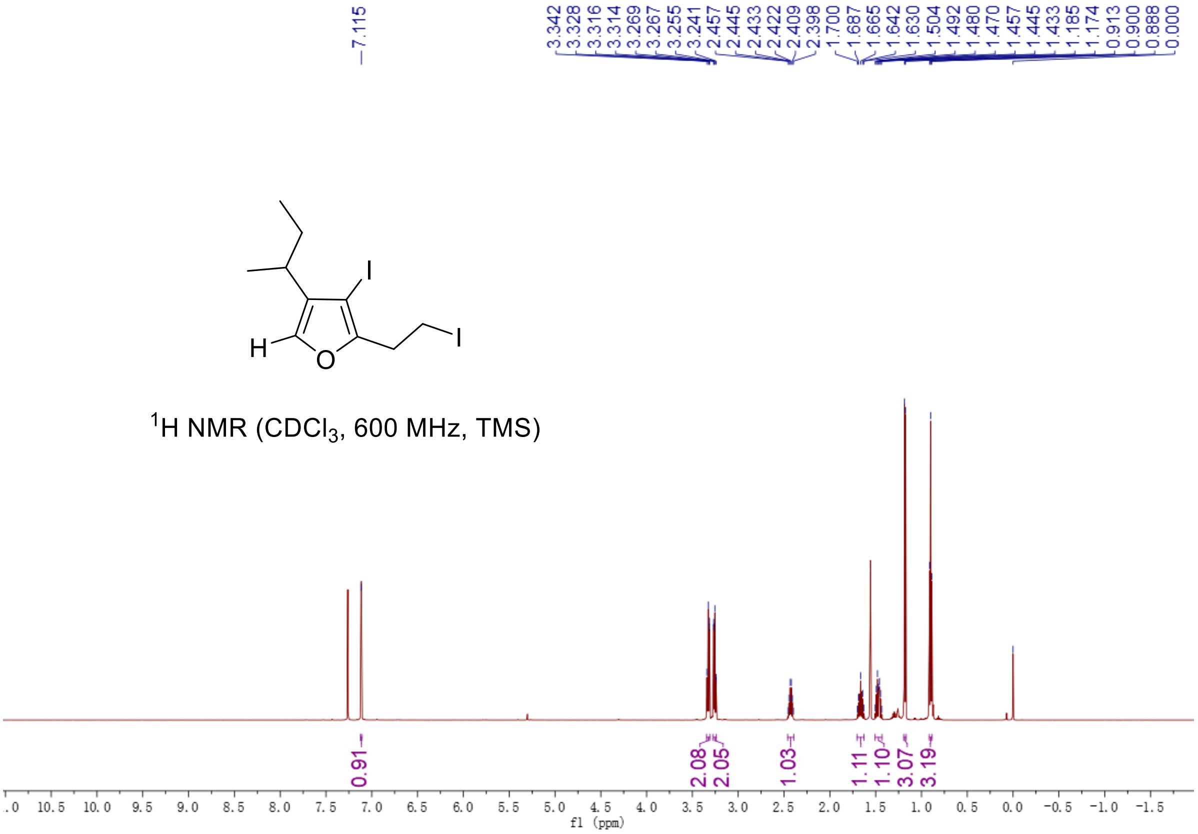

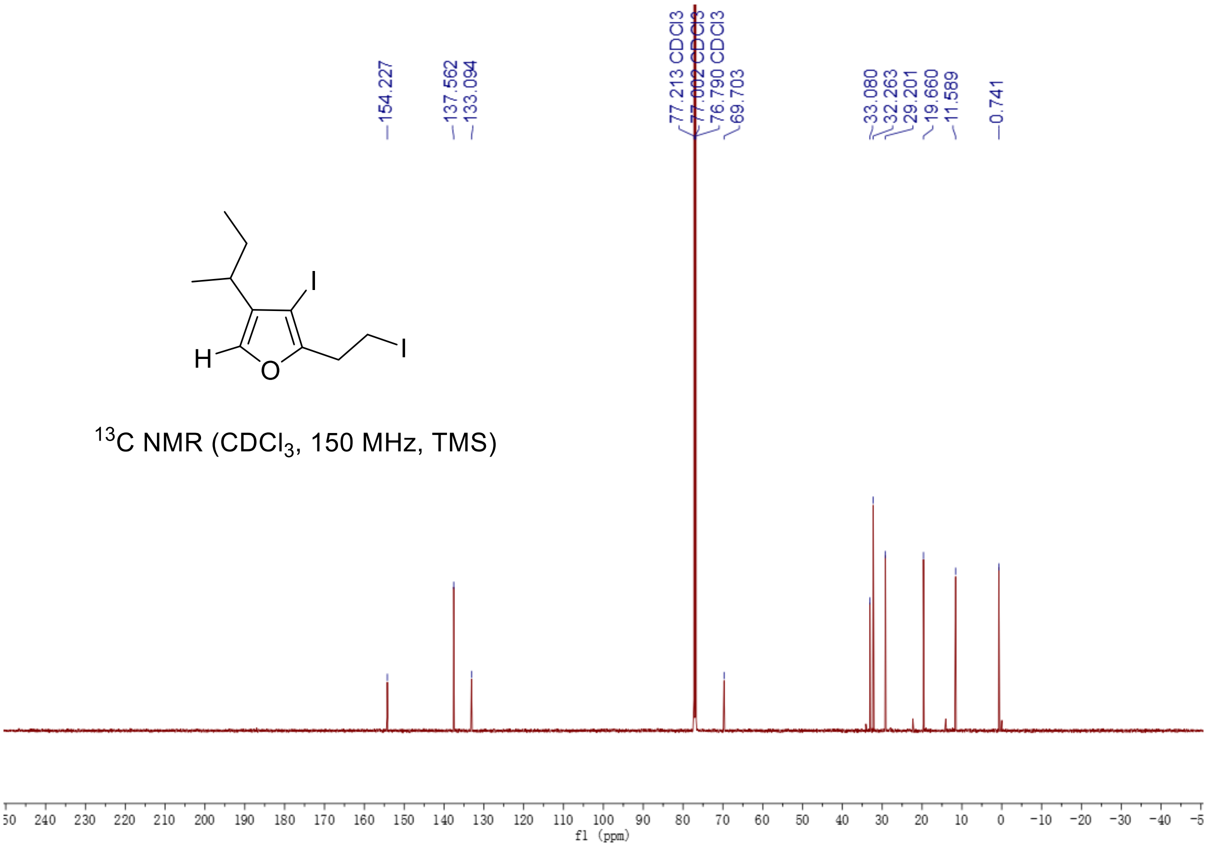

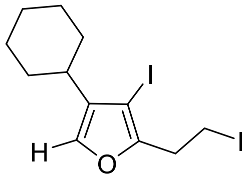

4-cyclohexyl-3-iodo-2-(2-iodoethyl)furan (6f)

A colorless oil, 71.1 mg, 65% yield. ^{1}H NMR ($CDCl_3$, TMS, 600 MHz) δ 7.11 (s, 1H), 3.33 - 3.30 (m, 2H), 3.27 - 3.23 (m, 2H), 2.24 (tt, J = 11.8, 3.4 Hz, 1H), 2.01-1.94 (m, 2H), 1.83 - 1.75 (m, 2H), 1.76 - 1.71 (m, 1H), 1.42 - 1.33 (m, 2H), 1.26 - 1.18 (m, 3H). ^{13}C NMR ($CDCl_3$, TMS, 150 MHz) δ 154.1, 137.2, 133.5, 69.3, 36.2, 33.0, 32.3, 26.4, 26.1, 0.7. IR (Acetone) ν 2936, 2924, 2853, 1611, 1589, 1497, 1457, 1356, 1250, 1230, 1214, 1029, 959, 823, 788, 744, 700 cm^{-1}. HRMS (ESI) calcd. for $C_{12}H_{16}I_2ONa$ (M+Na): 452.9188, Found: 452.9236.

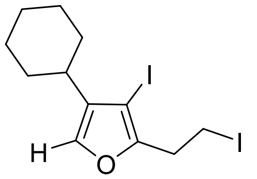

^{1}H NMR ($CDCl_3$, 600 MHz, TMS)

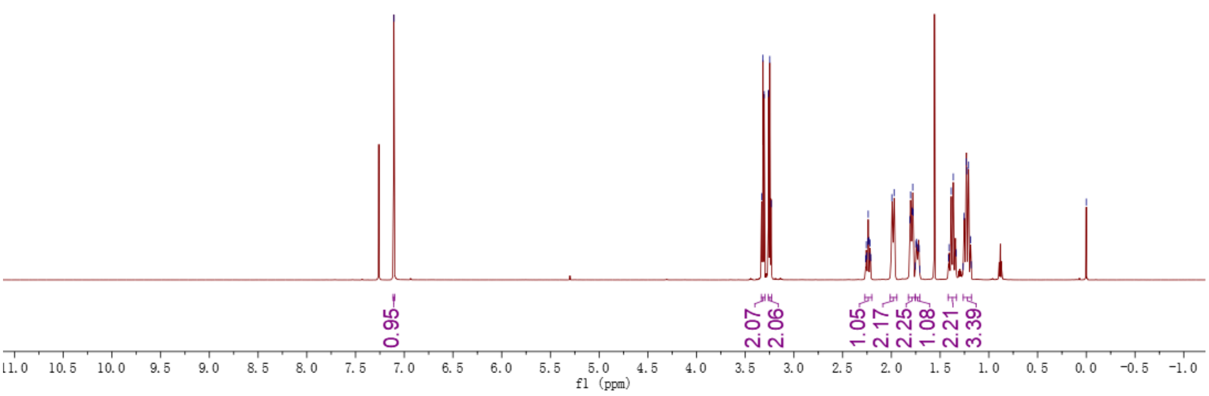

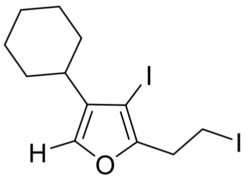

^{13}C NMR ($CDCl_3$, 150 MHz, TMS)

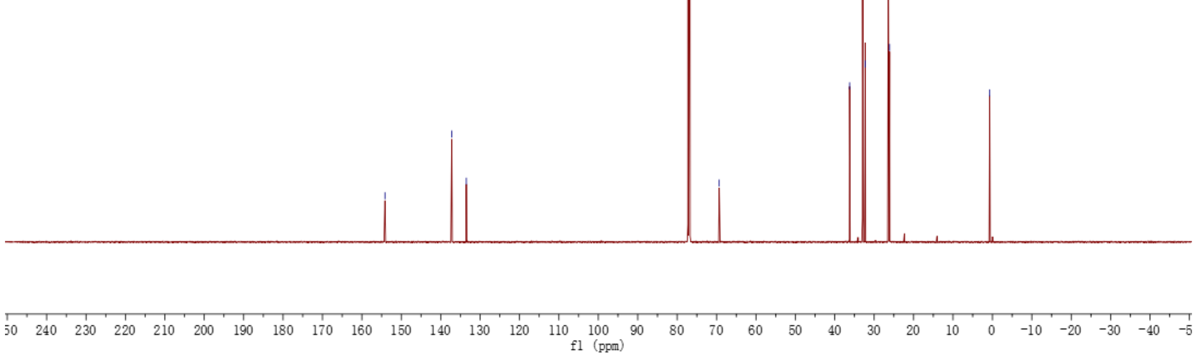

9. Spectroscopic data of products 8.

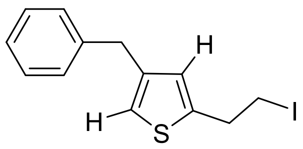

4-benzyl-2-(2-iodoethyl)thiophene (8a)

A colorless oil, 27.9 mg, 85% yield. ^{1}H NMR ($CDCl_3$, TMS, 600 MHz) δ 7.22 (t, *J* = 7.5 Hz, 2H), 7.13 (dd, *J* = 12.5, 7.2 Hz, 3H), 6.69 (s, 1H), 6.58 (s, 1H), 3.83 (s, 2H), 3.26 - 3.19 (m, 4H). ^{13}C NMR ($CDCl_3$, TMS, 150 MHz) δ 143.4, 141.4, 140.3, 128.7, 128.4, 126.8, 126.2, 119.8, 36.7, 34.6, 4.8. IR (Acetone) ν 927, 961, 1245, 1730, 1978, 2027, 2174, 2920 cm^{-1}. HRMS (EI) calcd. for $C_{13}H_{13}IS$ (M^+): 327.9785, Found: 327.9777.

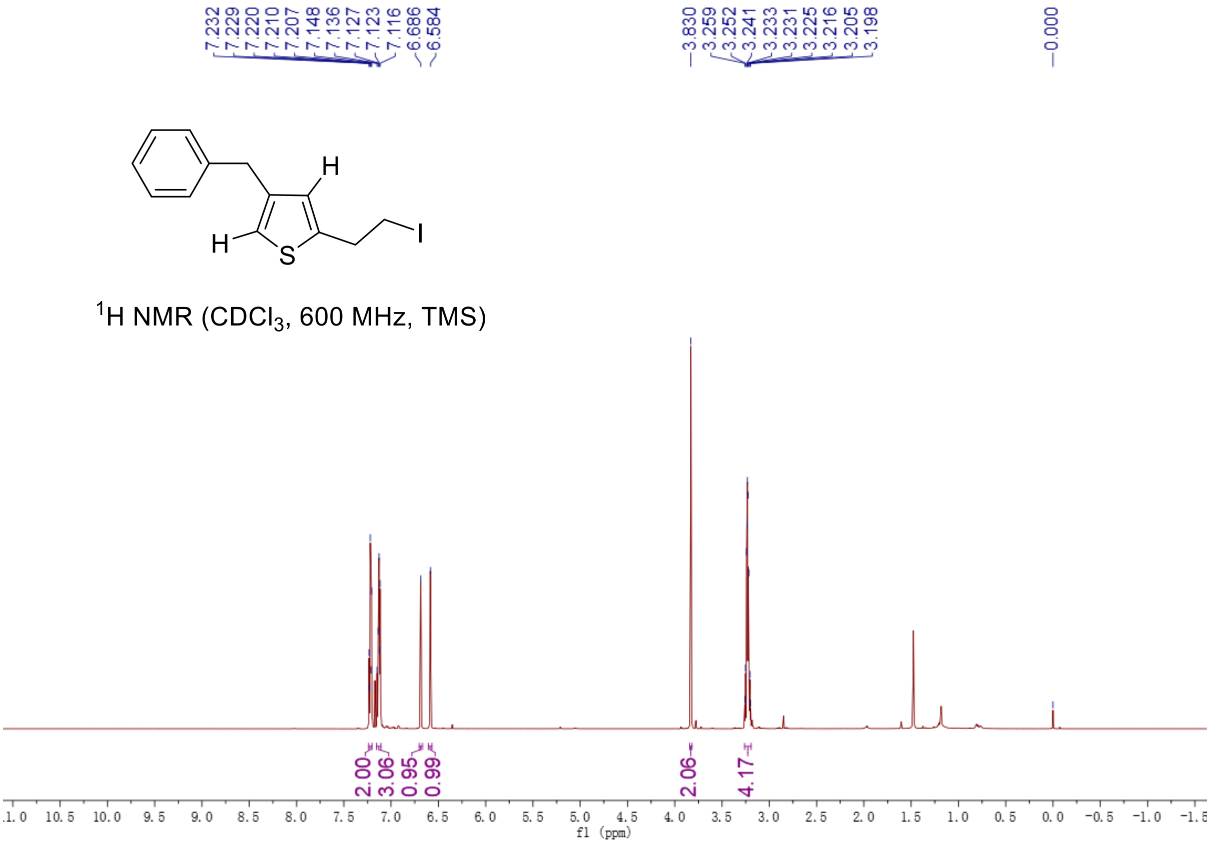

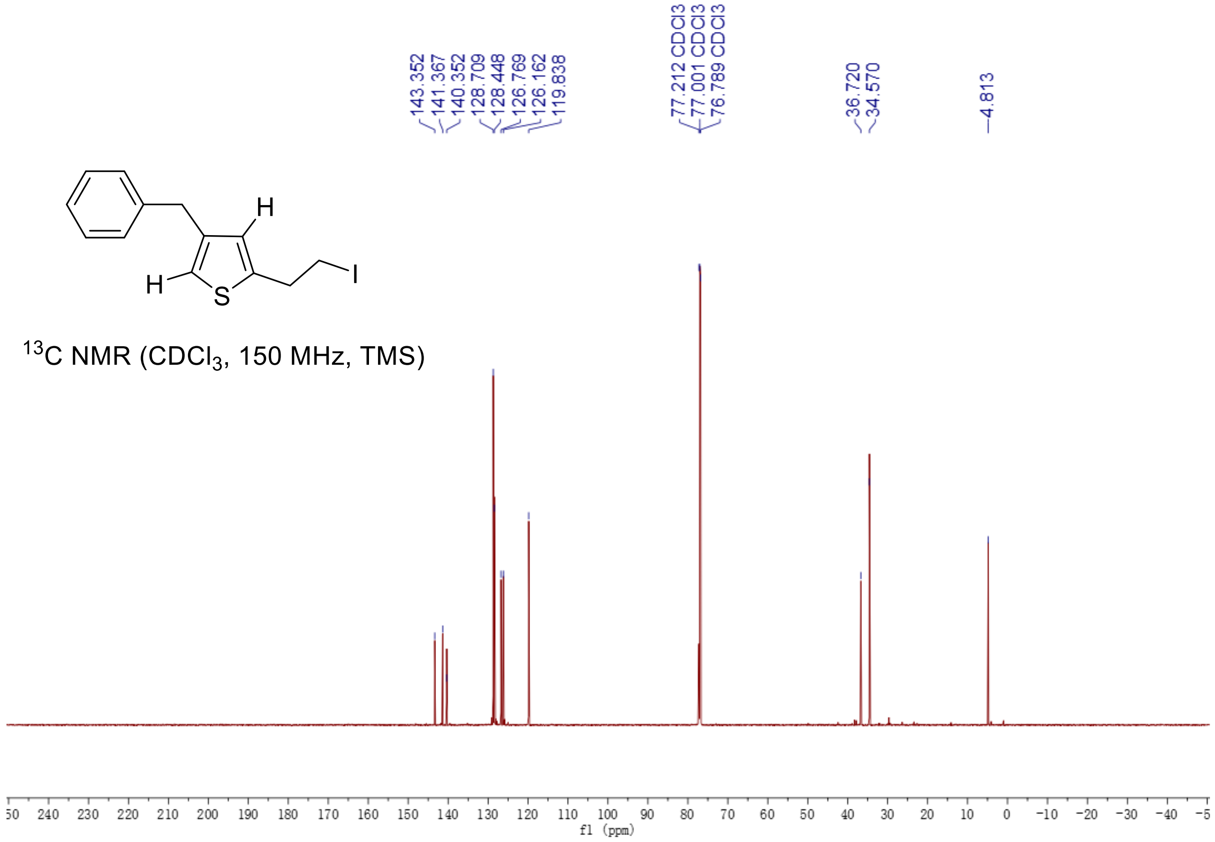

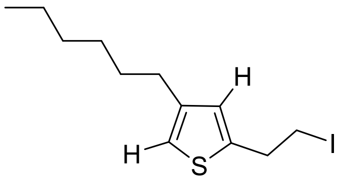

4-hexyl-2-(2-iodoethyl)thiophene (8b)

A colorless oil, 28.7 mg, 89% yield. ^{1}H NMR (CDCl$_3$, TMS, 600 MHz) δ 6.75 (s, 1H), 6.69 (s, 1H), 3.37 - 3.29 (m, 4H), 2.54 (t, J = 7.5 Hz, 2H), 1.61 - 1.55 (m, 3H), 1.38 - 1.25 (m, 7H), 0.92 - 0.85 (m, 3H). ^{13}C NMR (CDCl$_3$, TMS, 150 MHz) δ 143.2, 142.8, 126.7, 118.4, 34.6, 31.6, 30.5, 30.3, 29.0, 22.6, 14.1, 5.0. IR (Acetone) ν 927, 961, 1245, 1730, 1978, 2027, 2174, 2920 cm^{-1}. HRMS (EI) calcd. for $C_{12}H_{19}IS$ (M^+): 322.0255, Found: 322.0247.

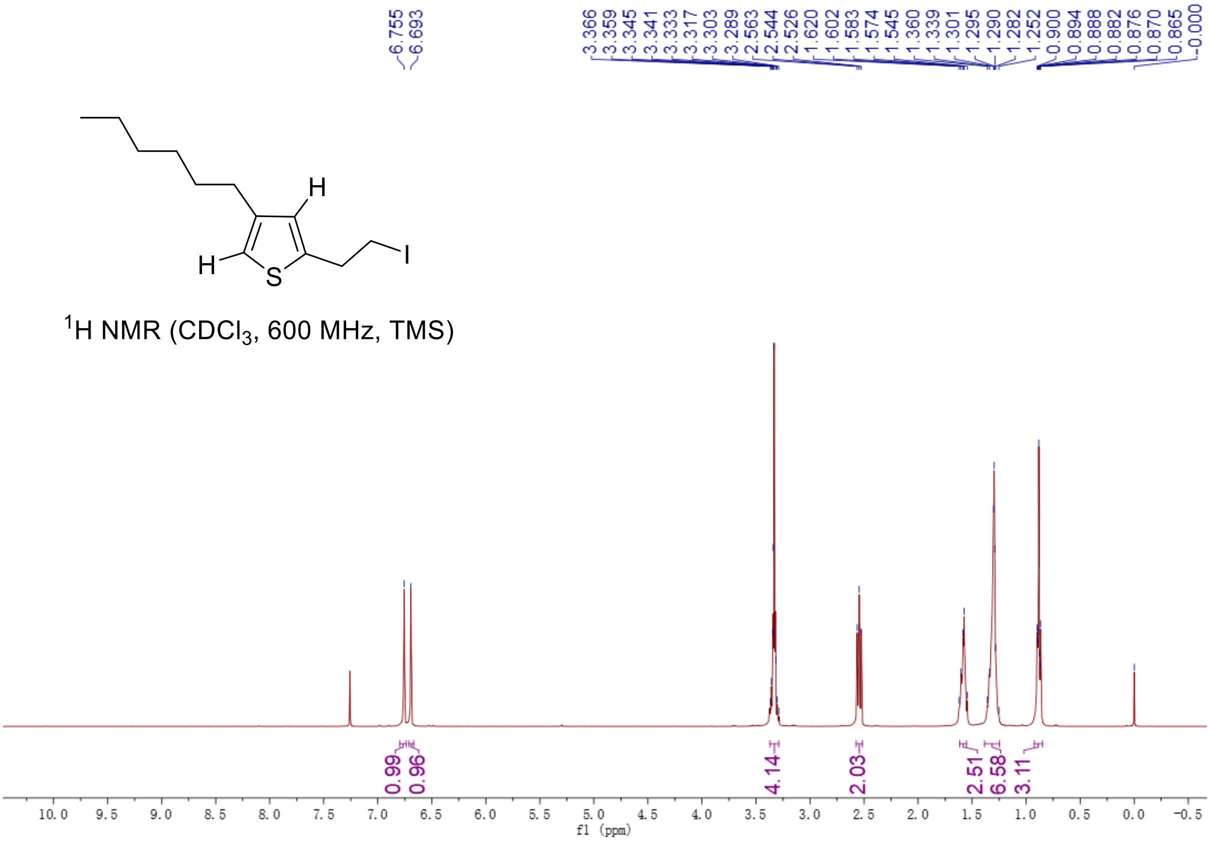
^{1}H NMR ($CDCl_3$, 600 MHz, TMS)
6.755
6.693
3.366 3.359 3.345 3.341 3.333 3.317 3.303 3.289 2.563 2.544 2.526 1.620 1.602 1.583 1.574 1.545 1.360 1.339 1.301 1.295 1.290 1.282 1.252 0.900 0.894 0.888 0.882 0.876 0.870 0.865 0.000
0.99 0.96 4.14 2.03 2.51 6.58 3.11
f1 (ppm)

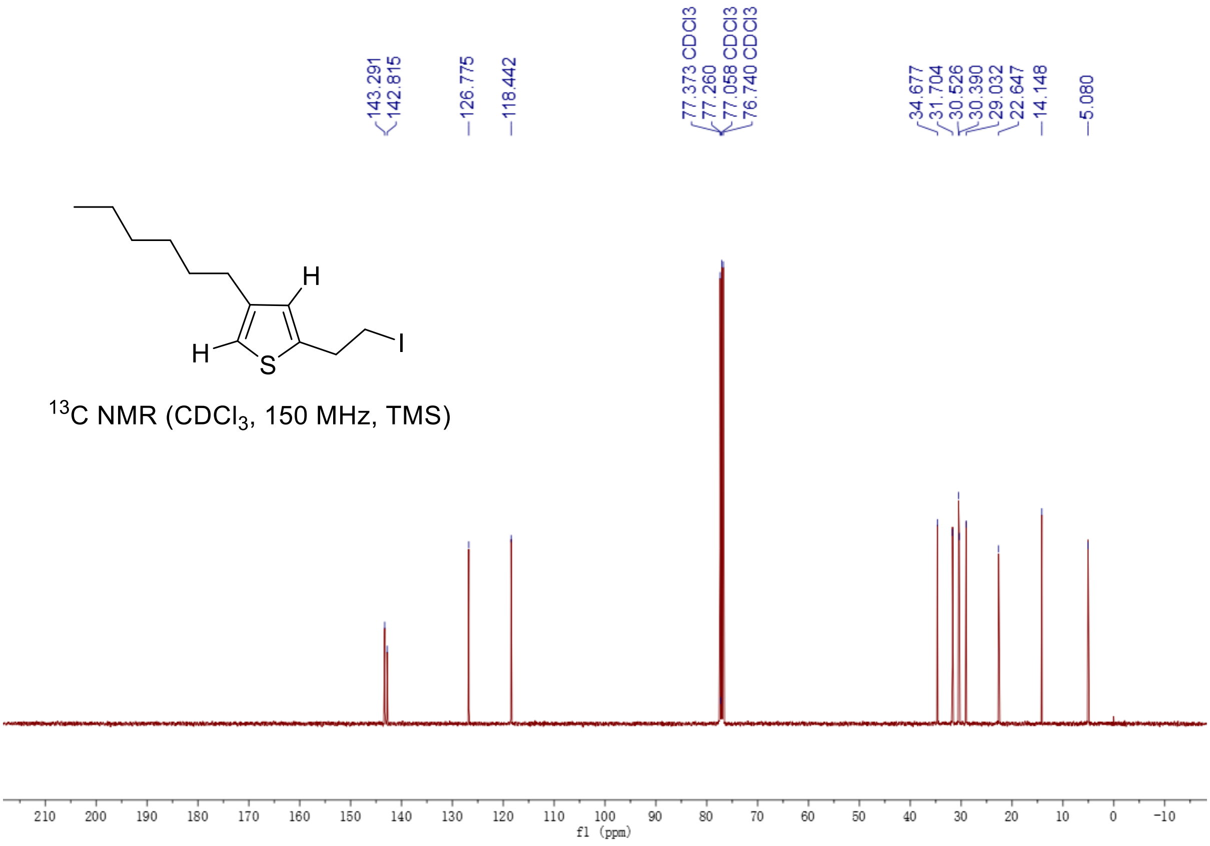
^{13}C NMR ($CDCl_3$, 150 MHz, TMS)
143.291
142.815
126.775
118.442
77.373 CDCl3
77.260
77.058 CDCl3
76.740 CDCl3
34.677 31.704 30.526 30.390 29.032 22.647 14.148
5.080
f1 (ppm)

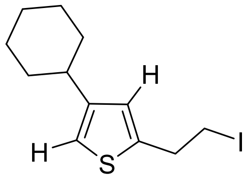

4-cyclohexyl-2-(2-iodoethyl)thiophene (8c)

A colorless oil, 25.6 mg, 80% yield. ^{1}H NMR ($CDCl_3$, TMS, 400 MHz) δ 6.76 (s, 1H), 6.75 (s, 1H), 3.37 - 3.29 (m, 4H), 2.58 - 2.46 (m, 1H), 1.94 (d, J = 5.7 Hz, 2H), 1.84 - 1.77 (m, 2H), 1.75 - 1.68 (m, 1H), 1.39 - 1.30 (m, 4H), 1.28 - 1.18 (m, 1H). ^{13}C NMR ($CDCl_3$, TMS, 100 MHz) δ 149.0, 142.6, 125.5, 116.9, 39.6, 34.8, 34.0, 26.5, 26.1, 4.9. IR (neat) ν 700, 732, 906, 1074, 1167, 1443, 1602, 2915 cm^{-1}. HRMS (EI) calcd. for $C_{12}H_{17}IS$ (M^+): 320.0098, Found: 320.0090.

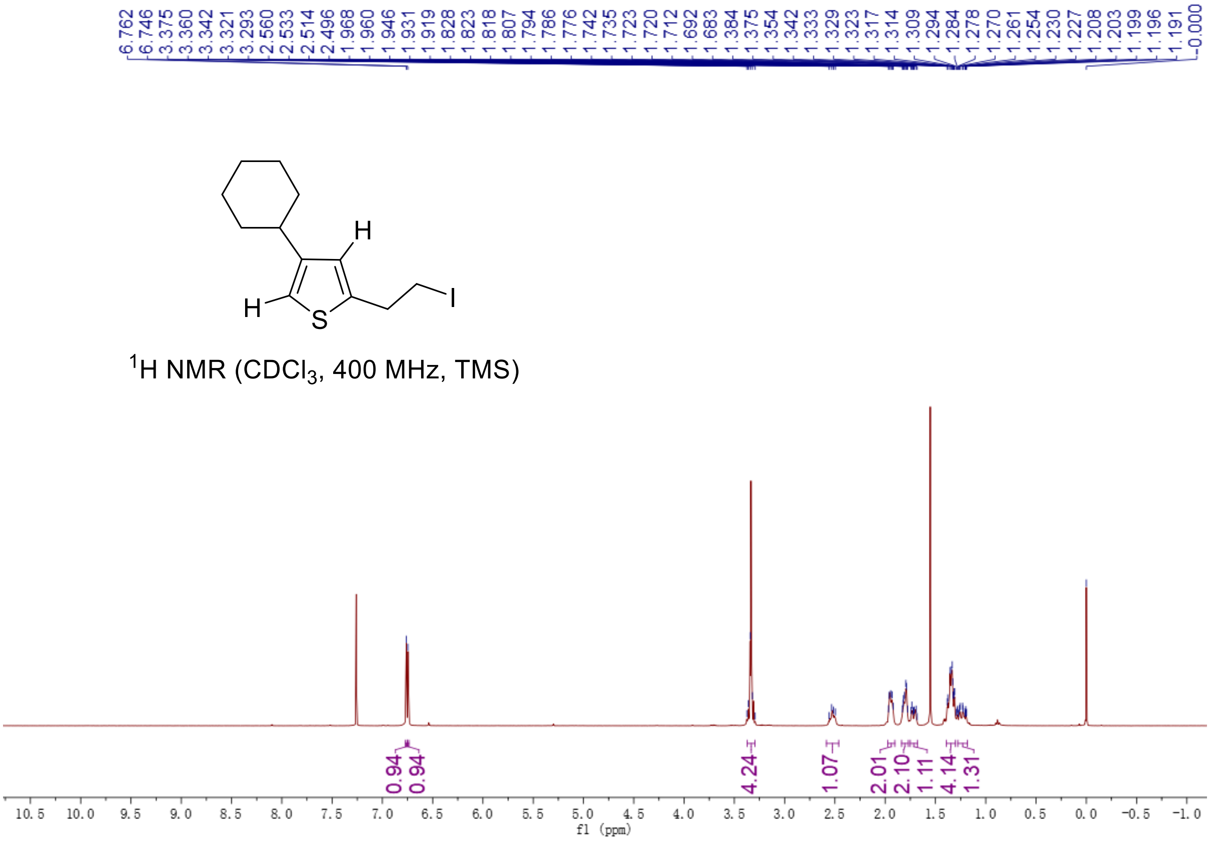

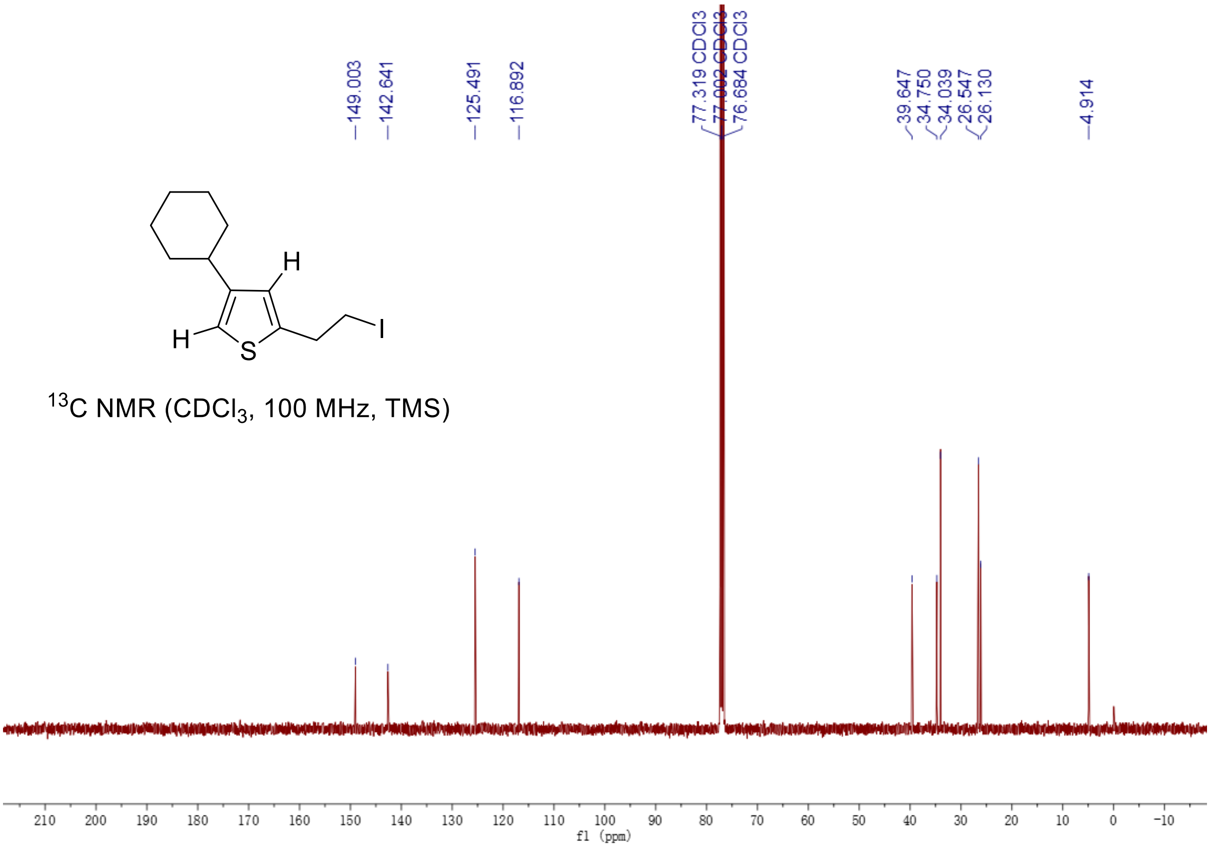

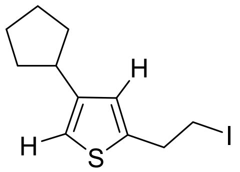

4-cyclopentyl-2-(2-iodoethyl)thiophene (8d)

A colorless oil, 25.1 mg, 82% yield. ^{1}H NMR ($CDCl_3$, TMS, 400 MHz) δ 6.78 (s, 1H), 6.74 (s, 1H), 3.40 - 3.27 (m, 4H), 3.03 - 2.92 (m, 1H), 2.08 - 1.95 (m, 2H), 1.83 - 1.69 (m, 2H), 1.68 - 1.59 (m, 2H), 1.56 - 1.48 (m, 2H). ^{13}C NMR ($CDCl_3$, TMS, 150 MHz) δ 147.3, 142.9, 125.8, 117.2, 53.4, 41.3, 34.7, 33.8, 25.1, 4.9. IR (Acetone) ν 930, 965, 1233, 1720, 2013, 2177, 2900 cm^{-1}. .

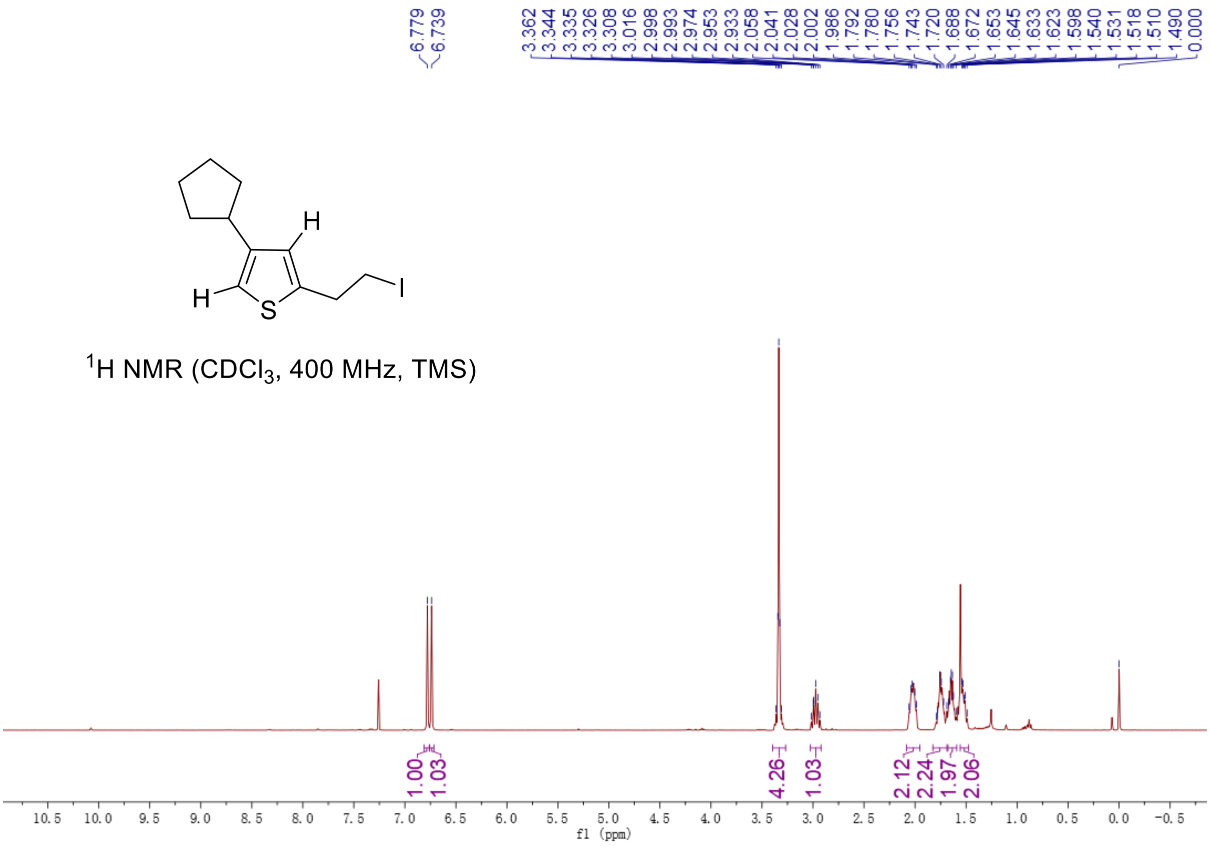
6.779
6.739
3.362
3.344
3.335
3.326
3.308
3.016
2.998
2.993
2.974
2.953
2.933
2.058
2.041
2.028
2.002
1.986
1.792
1.780
1.756
1.743
1.720
1.688
1.672
1.653
1.645
1.633
1.623
1.598
1.540
1.531
1.518
1.510
1.490
0.000
H
H
S
I
^{1}H NMR ($CDCl_3$, 400 MHz, TMS)
1.00
1.03
4.26
1.03
2.12
2.24
1.97
2.06
f1 (ppm)

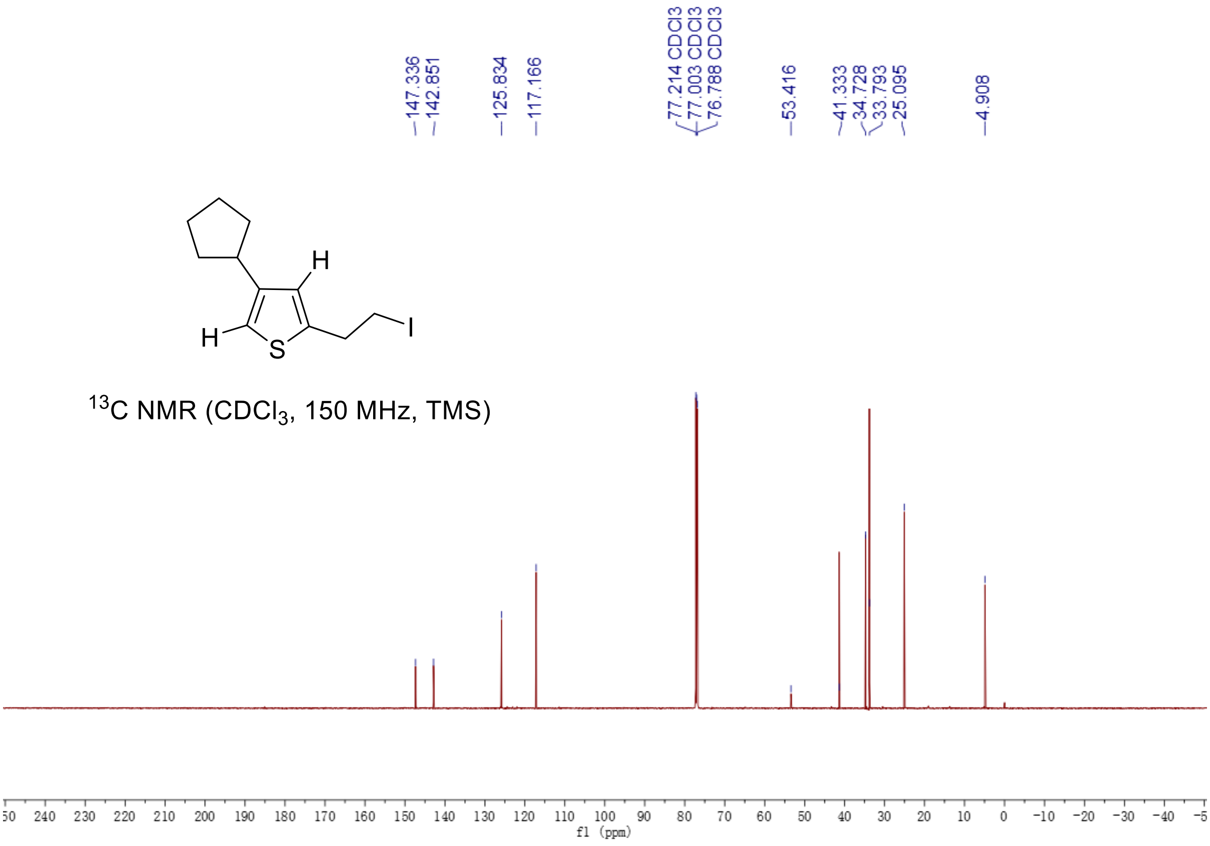
147.336
142.851
125.834
117.166
77.214 CDCl3
77.003 CDCl3
76.788 CDCl3
53.416
41.333
34.728
33.793
25.095
4.908
H
H
S
I
^{13}C NMR ($CDCl_3$, 150 MHz, TMS)
f1 (ppm)

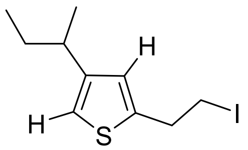

4-(sec-butyl)-2-(2-iodoethyl)thiophene (8e)

A colorless oil, 25.1 mg, 88% yield. ^{1}H NMR ($CDCl_3$, TMS, 400 MHz) δ 6.75 (s, 1H), 6.71 (s, 1H), 3.38 - 3.29 (m, 4H), 2.71 - 2.59 (m, 1H), 1.61 - 1.50 (m, 2H), 1.20 (d, *J* = 6.9 Hz, 3H), 0.84 (t, *J* = 7.5 Hz, 3H). ^{13}C NMR ($CDCl_3$, TMS, 100 MHz) δ 148.5, 142.7, 125.3, 117.5, 36.9, 34.7, 34.1, 30.7, 22.3, 21.0, 14.1, 11.9, 5.1. IR (Acetone) ν 937, 980, 1223, 1713, 2003, 2167, 2912 cm^{-1}. HRMS (EI) calcd. for $C_{10}H_{15}IS$ (M^+): 293.9941, Found: 293.9934.

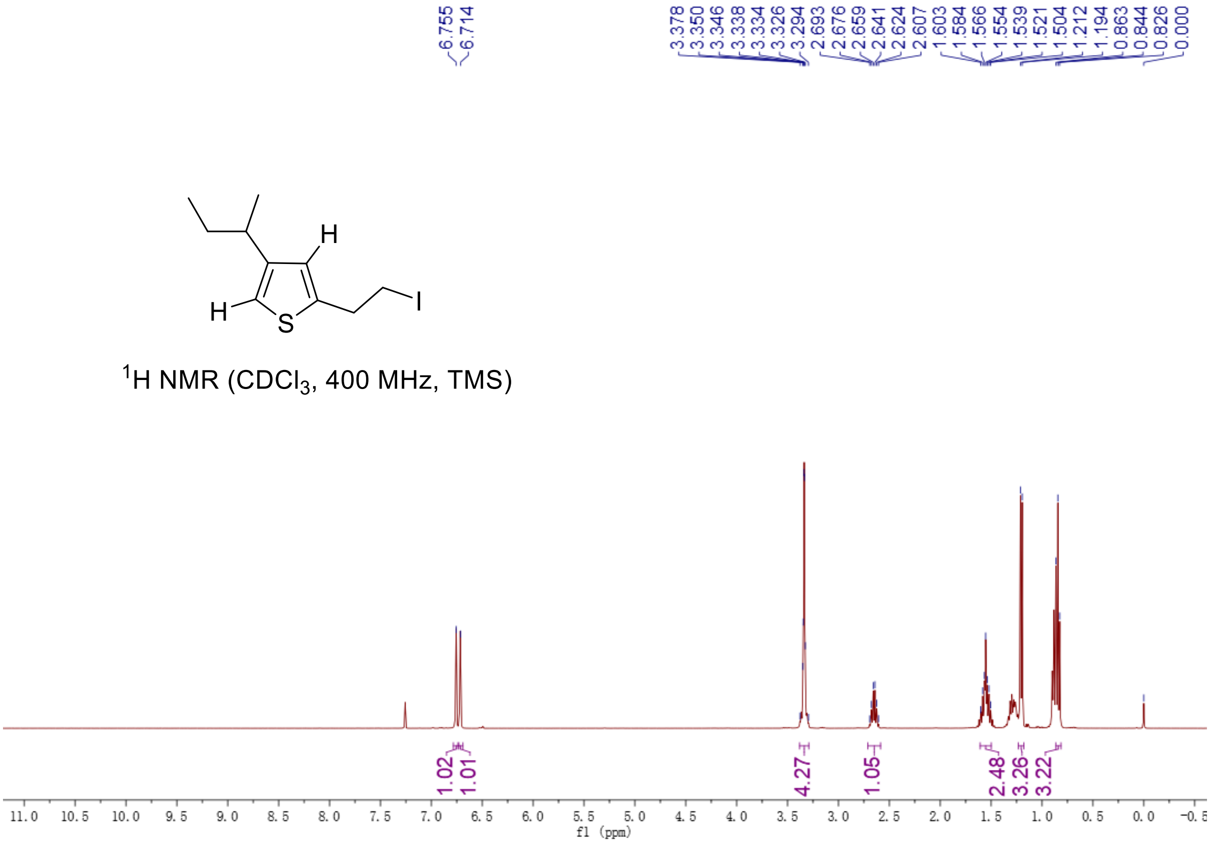

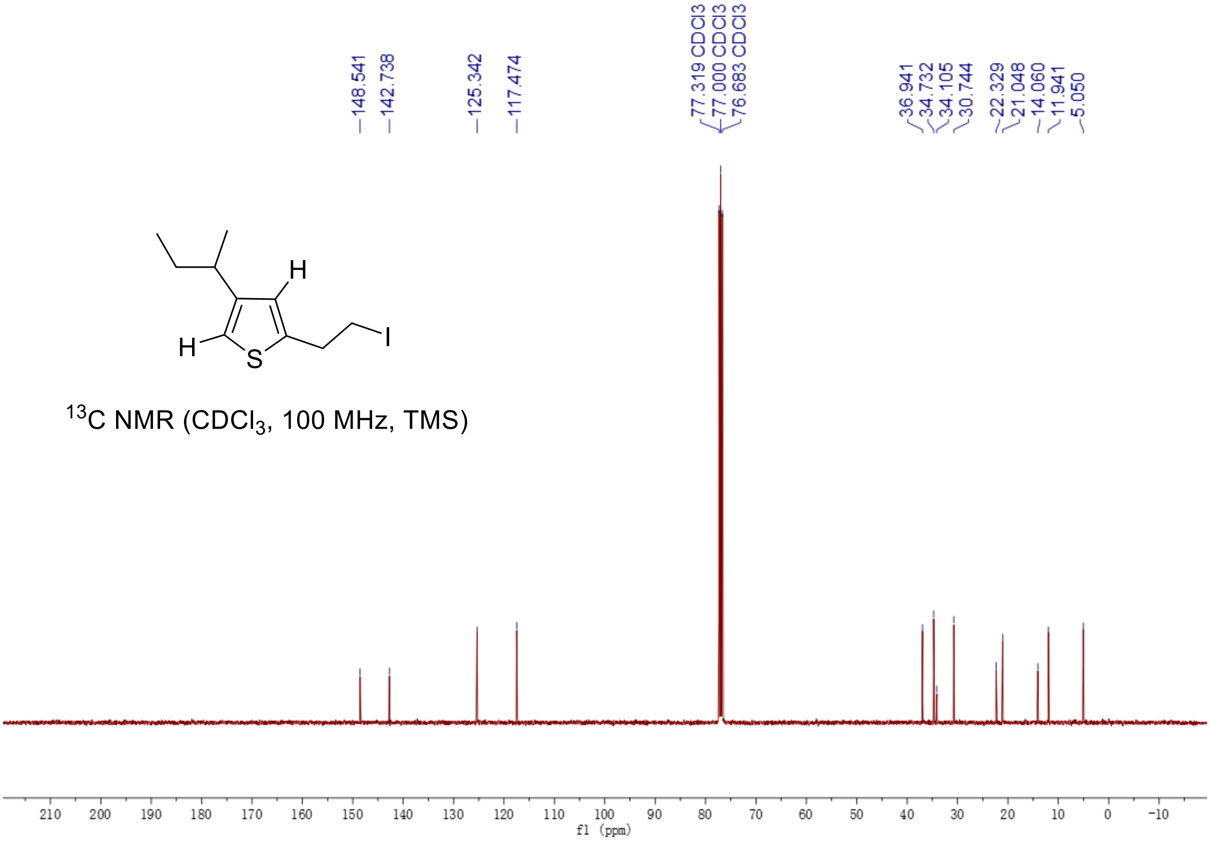

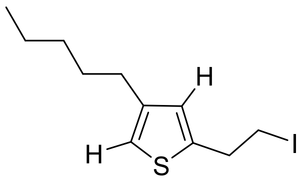

2-(2-iodoethyl)-4-pentylthiophene (8f)

A colorless oil, 25.6 mg, 83% yield. ^{1}H NMR ($CDCl_3$, TMS, 400 MHz) δ 6.76 (s, 1H), 6.70 (s, 1H), 3.38 - 3.28 (m, 4H), 2.54 (t, J = 7.7 Hz, 2H), 1.64 - 1.56 (m, 2H), 1.37 - 1.27 (m, 4H), 0.89 (t, J = 6.8 Hz, 3H). ^{13}C NMR ($CDCl_3$, TMS, 100 MHz) δ 143.2, 142.8, 126.7, 118.4, 76.7, 34.6, 31.5, 30.4, 30.1, 22.5, 14.0, 5.0. IR (Acetone) ν 927, 960, 1263, 1733, 2022, 2155, 2908 cm^{-1}. HRMS (EI) calcd. for $C_{11}H_{17}IS$ (M^+): 308.0095, Found: 308.0090.

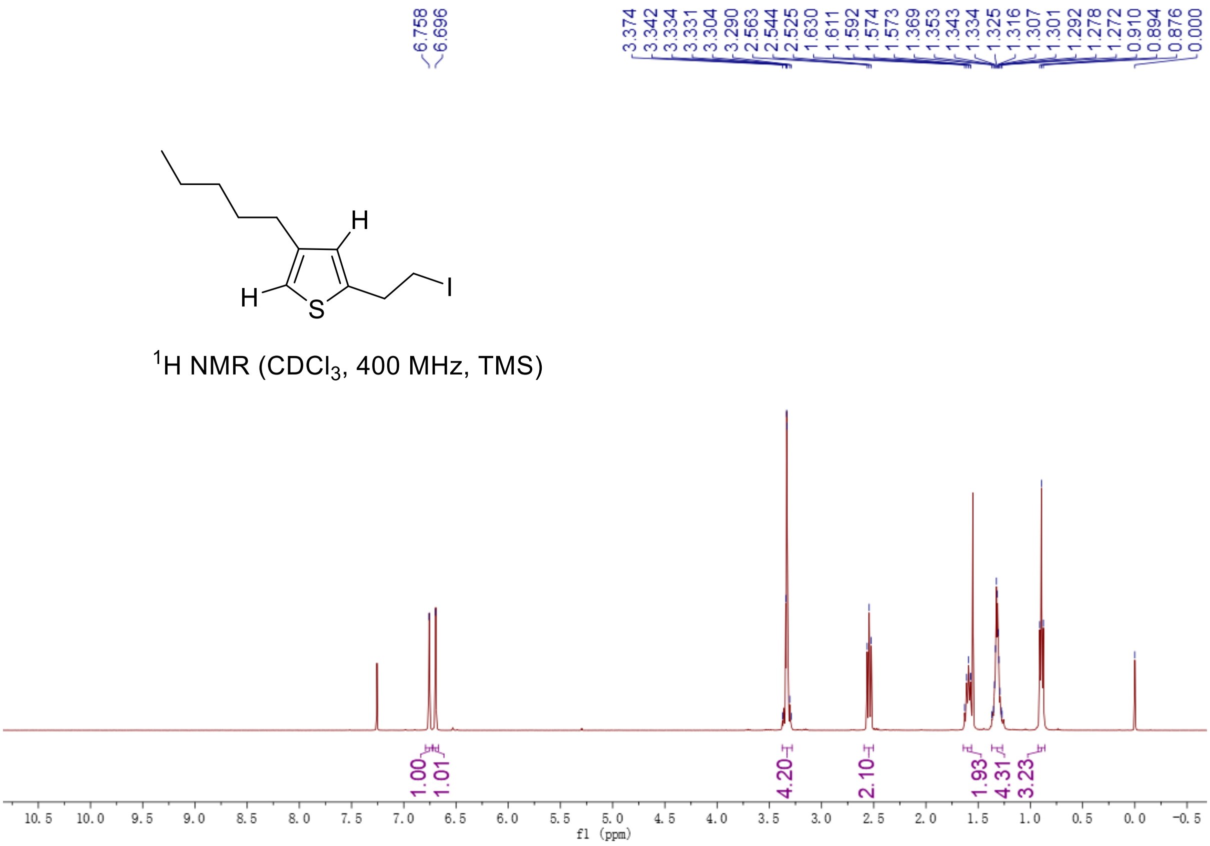

^{1}H NMR ($CDCl_3$, 400 MHz, TMS)

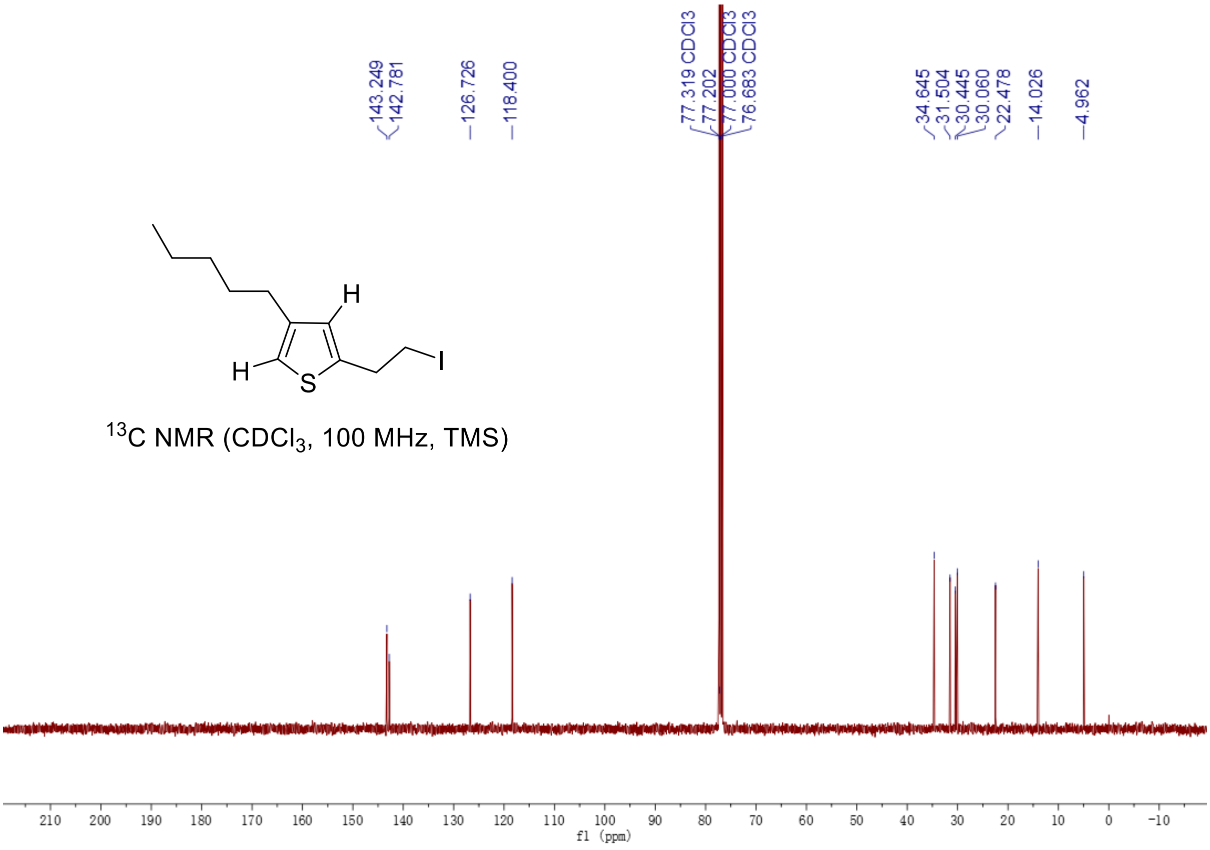

^{13}C NMR ($CDCl_3$, 100 MHz, TMS)

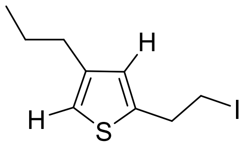

2-(2-iodoethyl)-4-propylthiophene (8g)

A colorless oil, 26.1 mg, 93% yield. ^{1}H NMR ($CDCl_3$, TMS, 400 MHz) δ 6.76 (s, 1H), 6.70 (s, 1H), 3.38 - 3.28 (m, 4H), 2.53 (t, *J* = 7.6 Hz, 2H), 1.66 - 1.58 (m, 2H), 0.94 (t, *J* = 7.4 Hz, 3H). ^{13}C NMR ($CDCl_3$, TMS, 100 MHz) δ 143.0, 142.8, 126.7, 118.5, 34.6, 32.6, 23.5, 13.9, 5.0. IR (neat) ν 937, 954, 1241, 1761, 2035, 2124, 2911 cm^{-1}. HRMS (EI) calcd. for $C_9H_{13}IS$ (M^+): 279.9780, Found: 279.9777.

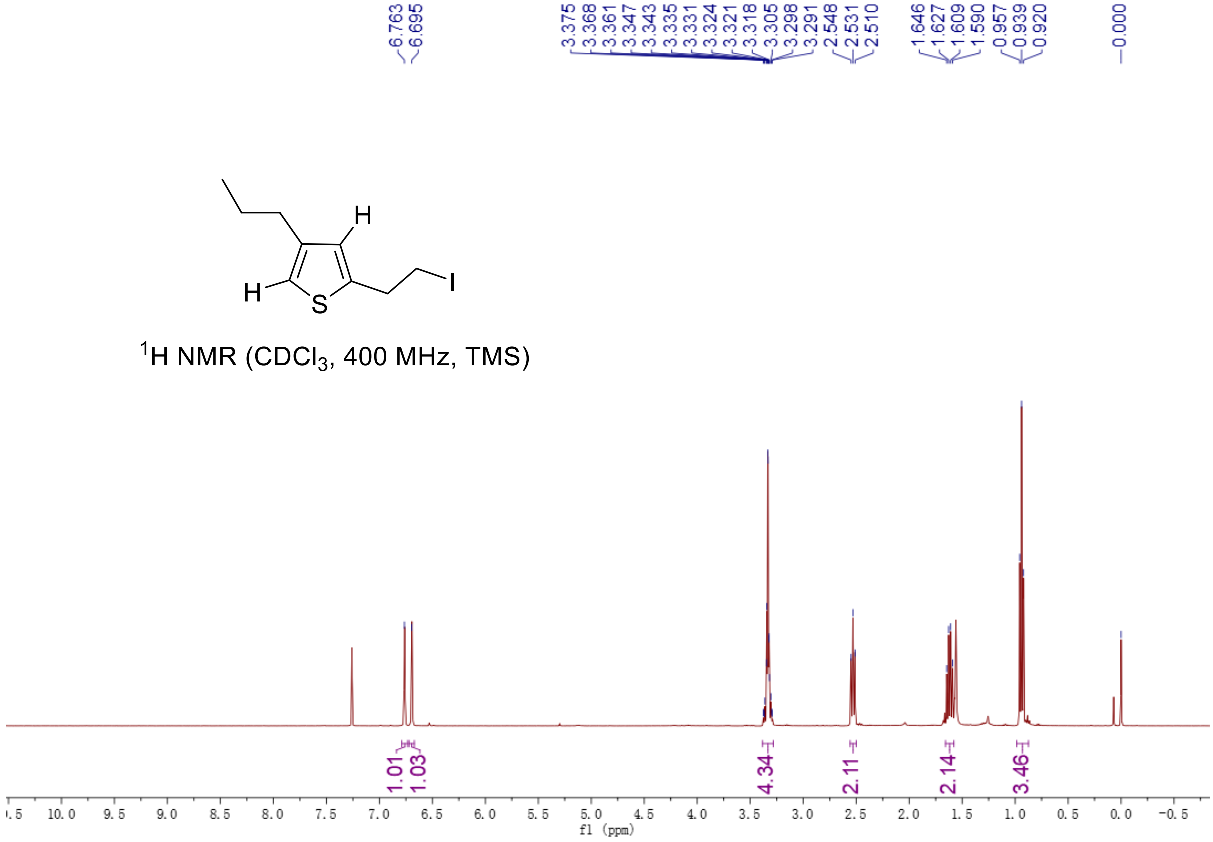

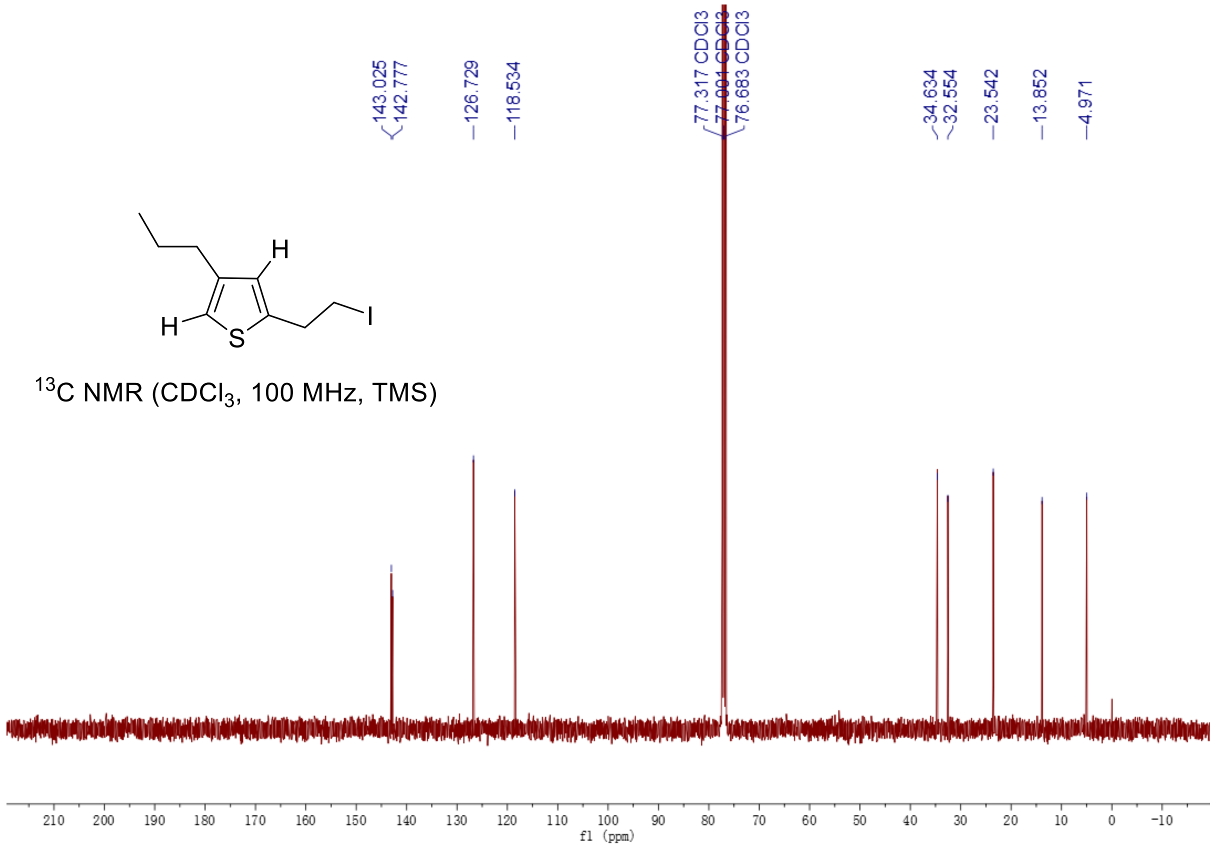

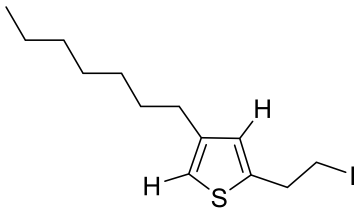

4-heptyl-2-(2-iodoethyl)thiophene (8h)

A colorless oil, 27.2 mg, 81% yield. ^{1}H NMR ($CDCl_3$, TMS, 400 MHz) δ 6.76 (s, 1H), 6.69 (s, 1H), 3.38 - 3.28 (m, 4H), 2.57 - 2.51 (m, 2H), 1.63 - 1.57 (m, 2H), 1.35 - 1.25 (m, 8H), 0.91 - 0.84 (m, 3H). ^{13}C NMR ($CDCl_3$, TMS, 100 MHz) δ 143.2, 142.8, 126.7, 118.4, 34.6, 31.8, 30.5, 30.4, 29.3, 29.1, 22.6, 14.1, 5.0. IR (neat) ν 927, 934, 1221, 1781, 2015, 2144, 2931 cm^{-1}. HRMS (EI) calcd. for $C_{13}H_{21}IS$ (M^+): 336.0414, Found: 336.0403.

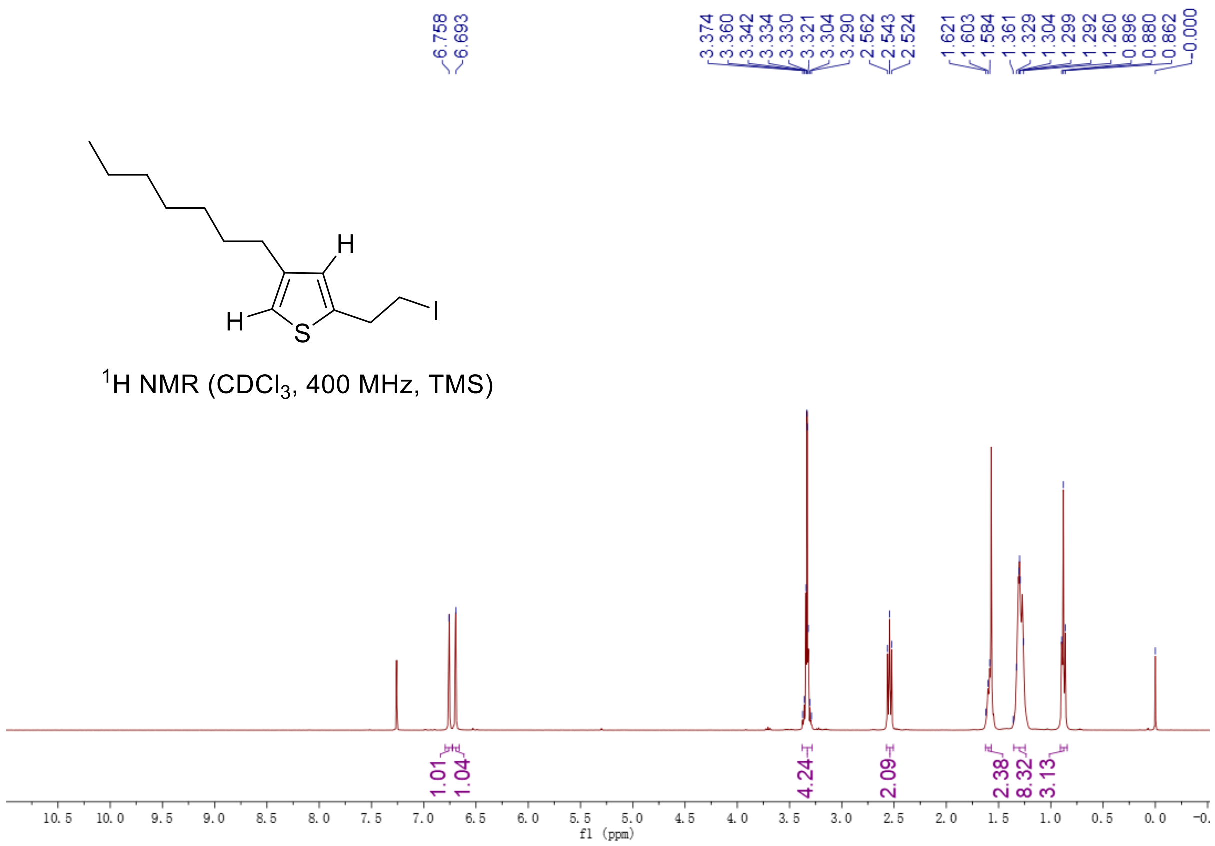
6.758
6.693
3.374
3.360
3.342
3.334
3.330
3.321
3.304
3.290
2.562
2.543
2.524
1.621
1.603
1.584
1.361
1.329
1.304
1.299
1.292
1.260
0.896
0.880
0.862
-0.000
H
H
S
I
^{1}H NMR ($CDCl_3$, 400 MHz, TMS)
1.01
1.04
4.24
2.09
2.38
8.32
3.13
f1 (ppm)

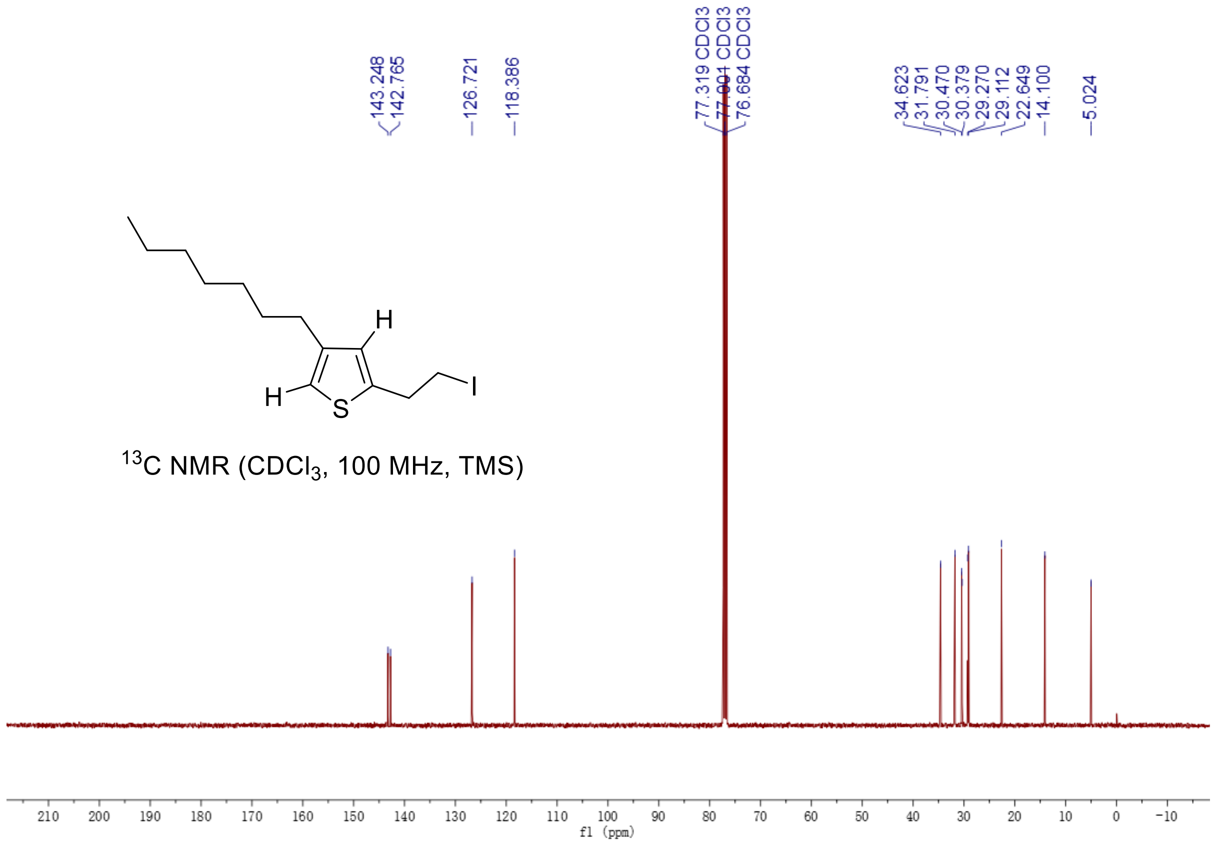
143.248
142.765
126.721
118.386
77.319 CDCl3
77.001 CDCl3
76.684 CDCl3
34.623
31.791
30.470
30.379
29.270
29.112
22.649
14.100
5.024
H
H
S
I
^{13}C NMR ($CDCl_3$, 100 MHz, TMS)
f1 (ppm)

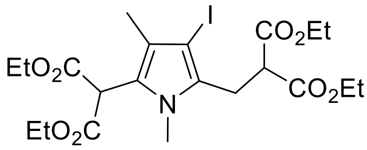

diethyl2-((5-(1,3-diethoxy-1,3-dioxopropan-2-yl)-3-iodo-1,4-dimethyl-1H-pyrrol-2-yl)methyl) malonate (9)

A yellow oil, 37.5 mg, 68% yield. ^{1}H NMR ($CDCl_3$, TMS, 400 MHz) δ 4.27 - 4.10 (m, 8H), 3.59 (s, 3H), 3.40 (t, J = 7.1 Hz, 1H), 3.36 (s, 1H), 2.84 - 2.73 (m, 2H), 2.09-2.01 (m, 5H), 1.33 - 1.23 (m, 12H). ^{13}C NMR ($CDCl_3$, TMS, 150 MHz) δ 169.0, 166.6, 136.3, 125.7, 70.8, 67.3, 61.6, 51.0, 41.7, 36.7, 28.1, 26.4, 16.7, 14.1. IR (neat) ν 615, 803, 933, 1179, 1316, 1758, 2901, 2988 cm^{-1}. HRMS (EI) calcd. for $C_{20}H_{30}INO_8Na$ (M+Na): 574.0914, Found: 574.0918.

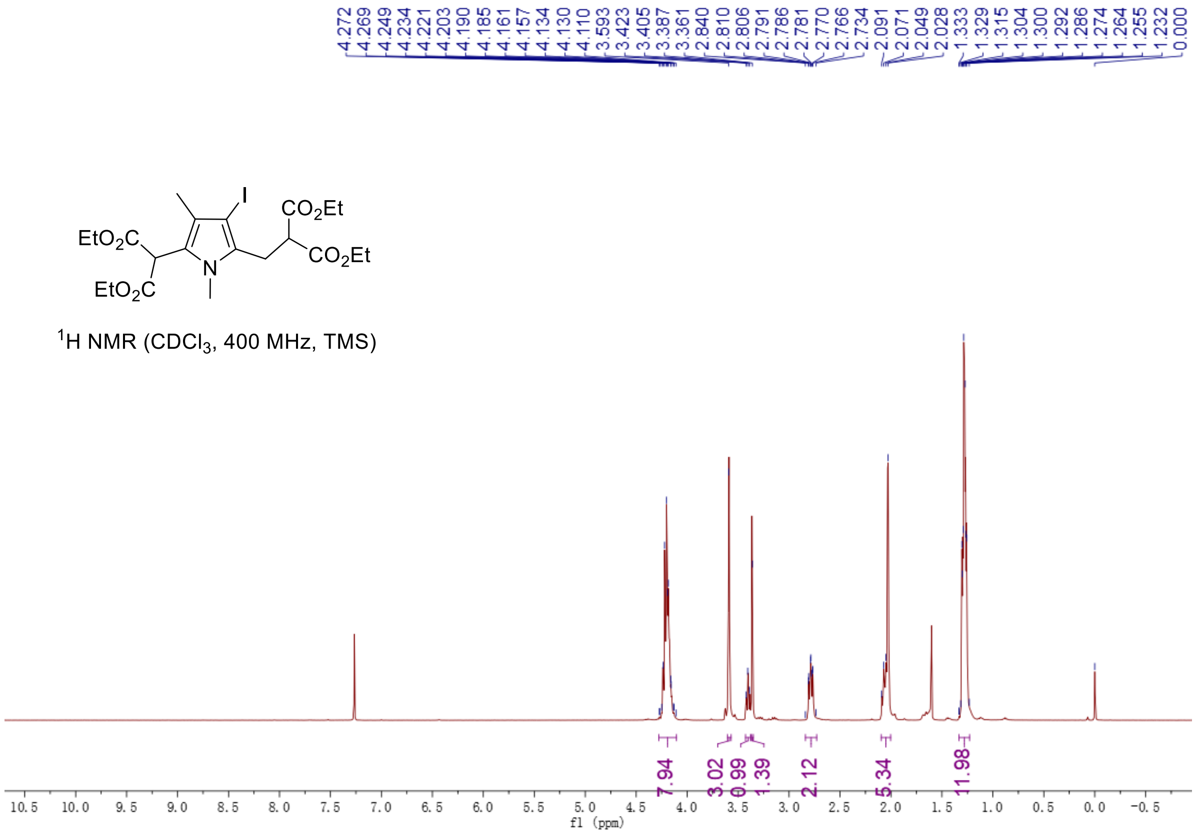

4.272 4.269 4.249 4.234 4.221 4.203 4.190 4.185 4.161 4.157 4.134 4.130 4.110 3.593 3.423 3.405 3.387 3.361 2.840 2.810 2.806 2.791 2.786 2.781 2.770 2.766 2.734 2.091 2.071 2.049 2.028 1.333 1.329 1.315 1.304 1.300 1.292 1.286 1.274 1.264 1.255 1.232 0.000
EtO2C
EtO2C
CO2Et
CO2Et
I
N
1H NMR (CDCl3, 400 MHz, TMS)
7.94
3.02
0.99
1.39
2.12
5.34
11.98
f1 (ppm)

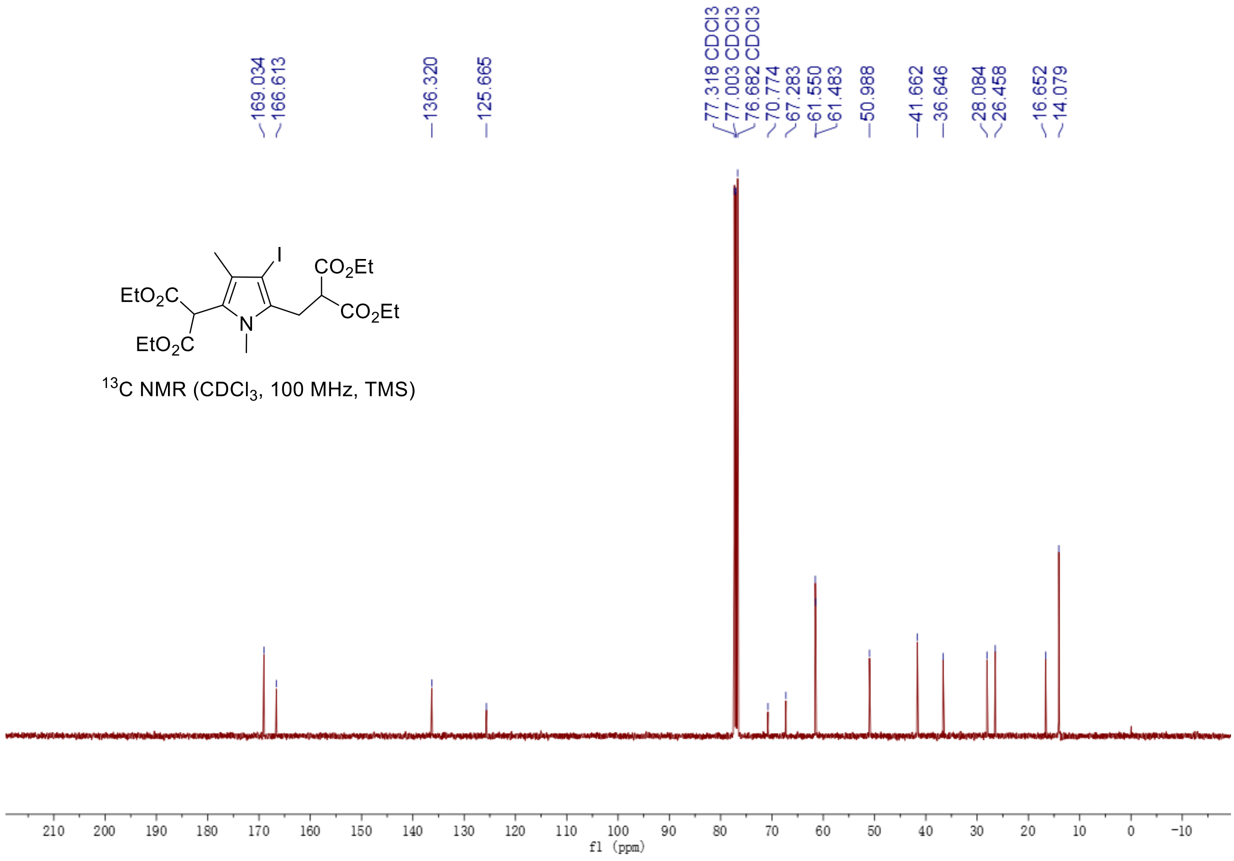

169.034 166.613 136.320 125.665 77.318 CDCl3 77.003 CDCl3 76.682 CDCl3 70.774 67.283 61.550 61.483 50.988 41.662 36.646 28.084 26.458 16.652 14.079
EtO2C
EtO2C
CO2Et
CO2Et
I
N
13C NMR (CDCl3, 100 MHz, TMS)
f1 (ppm)

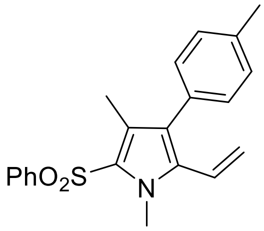

1,3-dimethyl-2-(phenylsulfonyl)-4-(p-tolyl)-5-vinyl-1H-pyrrole (11)

A yellow oil, 16.9 mg, 68% yield. ^{1}H NMR (CDCl$_3$, TMS, 600 MHz) δ 7.91 - 7.89 (m, 2H), 7.59 - 7.56 (m, 1H), 7.54 - 7.50 (m, 2H), 7.20 - 7.17 (m, 2H), 7.09 - 7.06 (m, 2H), 6.40 (dd, *J* = 17.9, 11.9 Hz, 1H), 5.27 (d, *J* = 11.8 Hz, 1H), 5.16 (d, *J* = 17.8 Hz, 1H), 3.77 (s, 3H), 2.38 (s, 3H), 2.30 (s, 3H). ^{13}C NMR (CDCl$_3$, TMS, 150 MHz) δ 143.5, 136.5, 134.4, 132.7, 131.4, 130.5, 129.2, 129.1, 127.8, 126.4, 125.3, 124.7, 123.6, 122.3, 120.3, 33.2, 26.9, 21.2, 11.2. IR (neat) ν 603, 815, 957, 1179, 1316, 2921, 2978 cm^{-1}. HRMS (EI) calcd. for $C_{21}H_{21}NO_2S$ (M+H): 352.1371, Found: 352.1399.

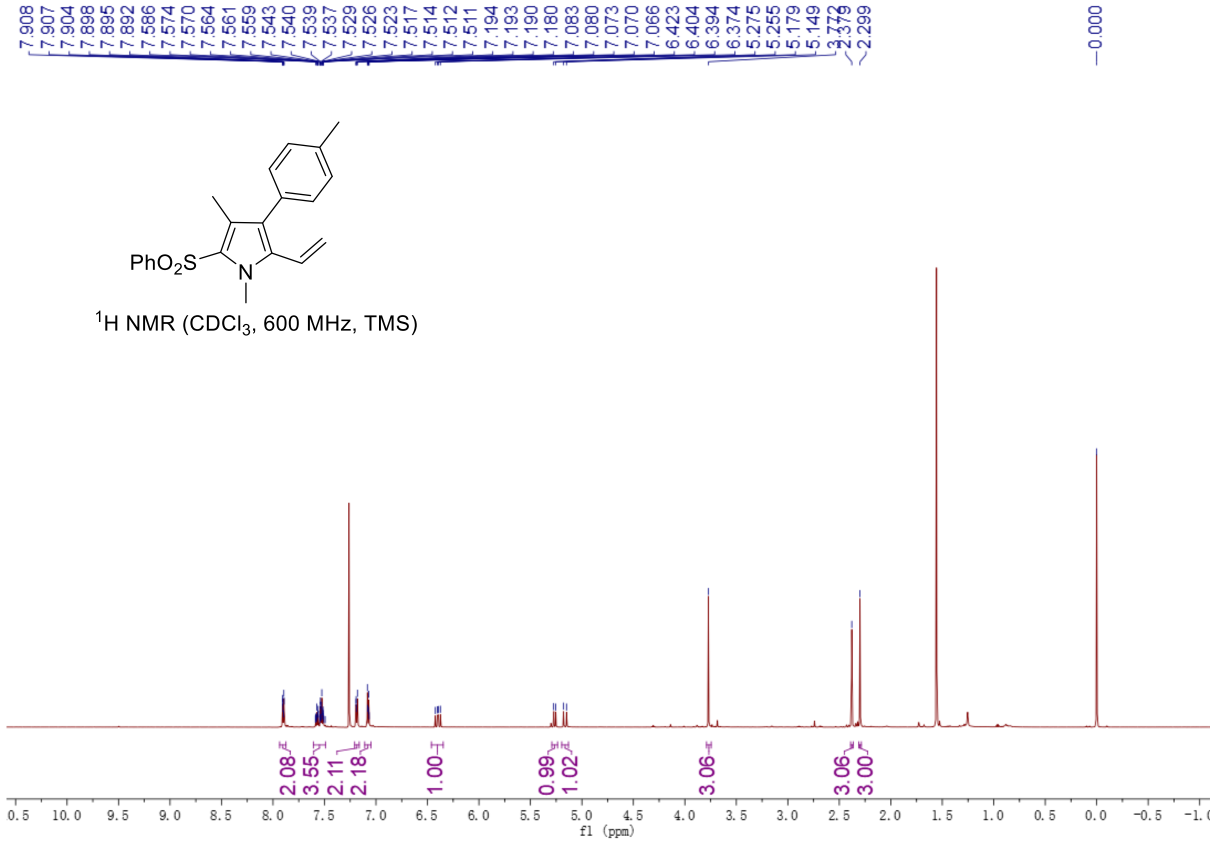

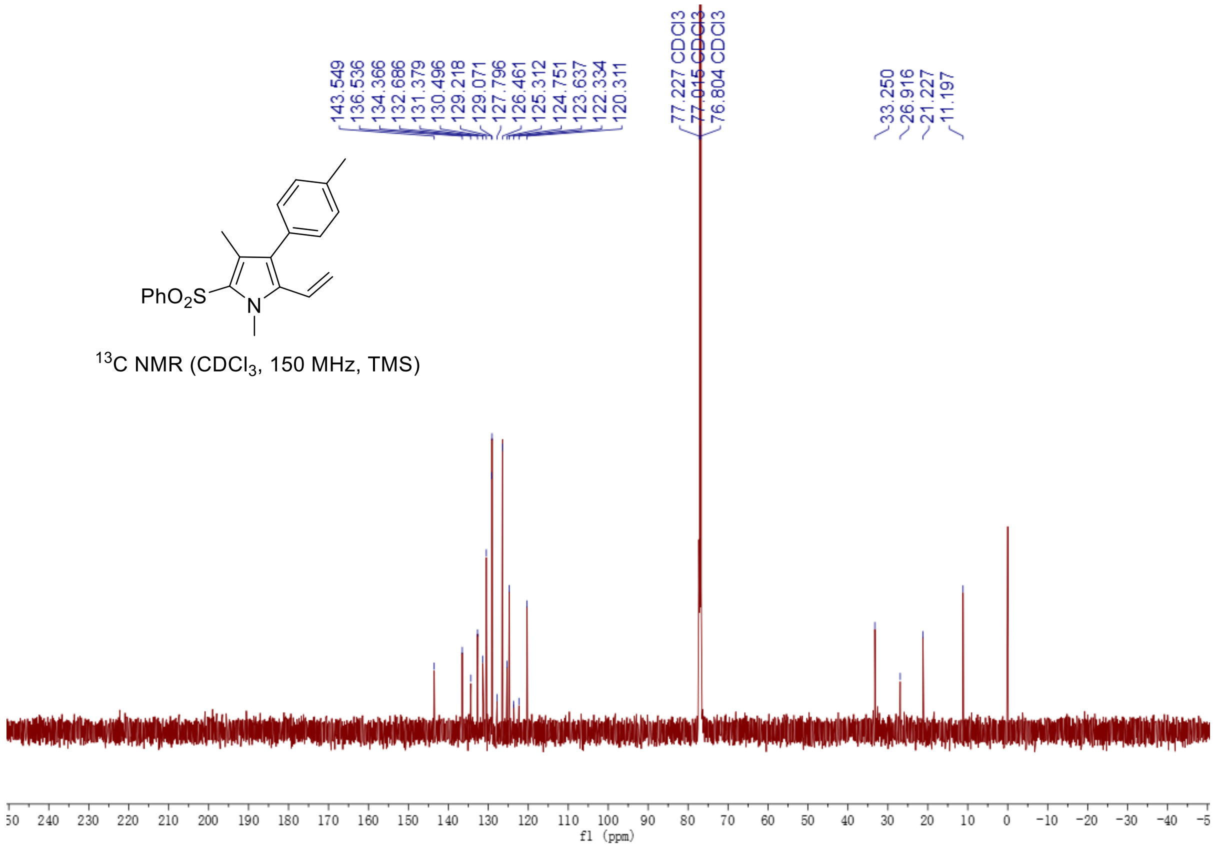
143.549
136.536
134.366
132.686
131.379
130.496
129.218
129.071
127.796
126.461
125.312
124.751
123.637
122.334
120.311
77.227 CDCl3
77.015 CDCl3
76.804 CDCl3
33.250
26.916
21.227
11.197
PhO2S
N
13C NMR (CDCl3, 150 MHz, TMS)
f1 (ppm)

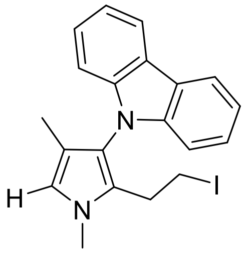

9-(2-(2-iodoethyl)-1,4-dimethyl-1H-pyrrol-3-yl)-9H-carbazole (12)

A yellow oil, 14.5 mg, 35% yield. ^{1}H NMR ($CDCl_3$, TMS, 600 MHz) δ 8.09 (d, *J* = 7.7 Hz, 2H), 7.43 (t, *J* = 7.7 Hz, 2H), 7.31 (d, *J* = 8.2 Hz, 2H), 7.22 (t, *J* = 7.4 Hz, 2H), 6.26 (s, 1H), 4.45 (t, *J* = 7.1 Hz, 2H), 3.11 (t, *J* = 7.1 Hz, 2H), 3.00 (s, 3H), 2.00 (s, 3H). ^{13}C NMR (CDCl3, TMS, 150 MHz) δ 140.0, 130.4, 125.8, 122.9, 120.7, 120.3, 119.9, 119.0, 108.5, 68.3, 42.5, 34.1, 26.3, 13.4. IR (neat) ν 927, 934, 1221, 1781, 2015, 2144, 2931 cm^{-1}. HRMS (EI) calcd. for $C_{20}H_{19}N_2I$ (M+): 414.0593, Found: 414.0593.

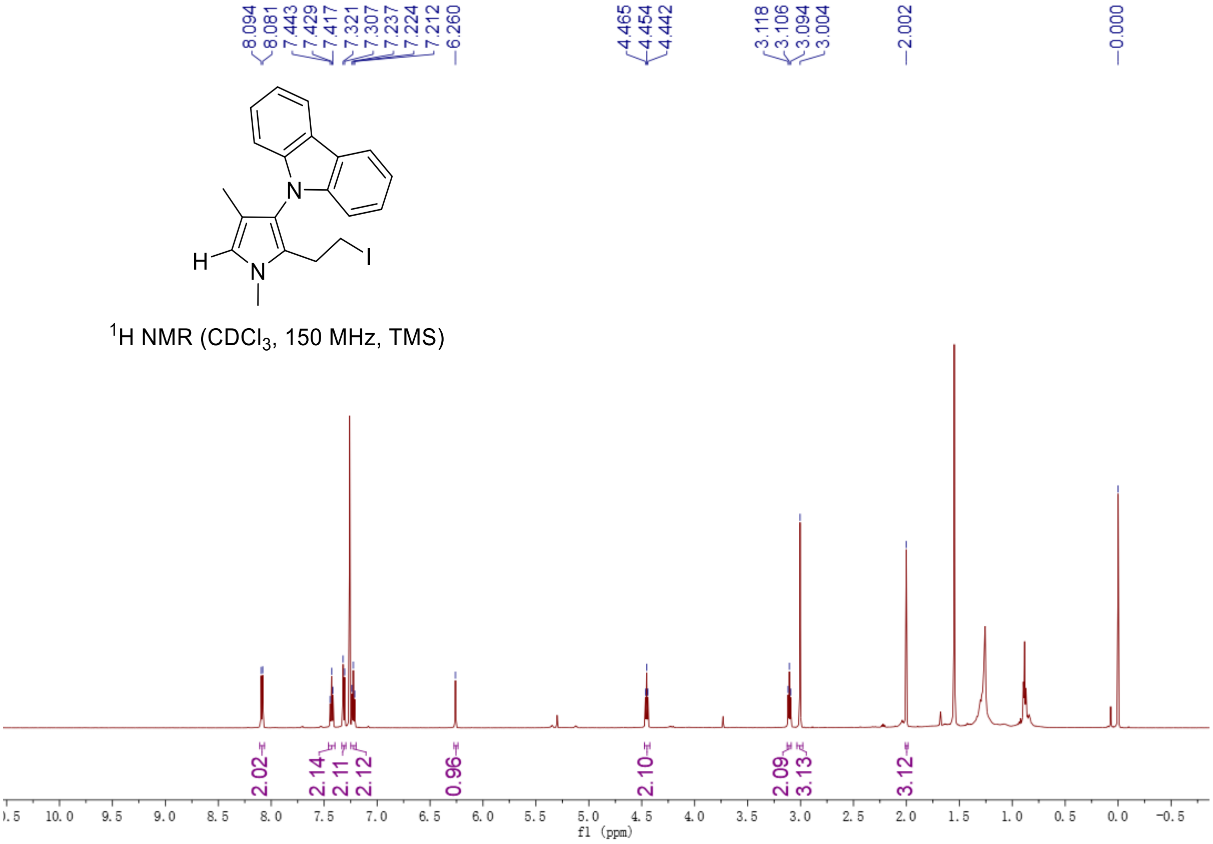

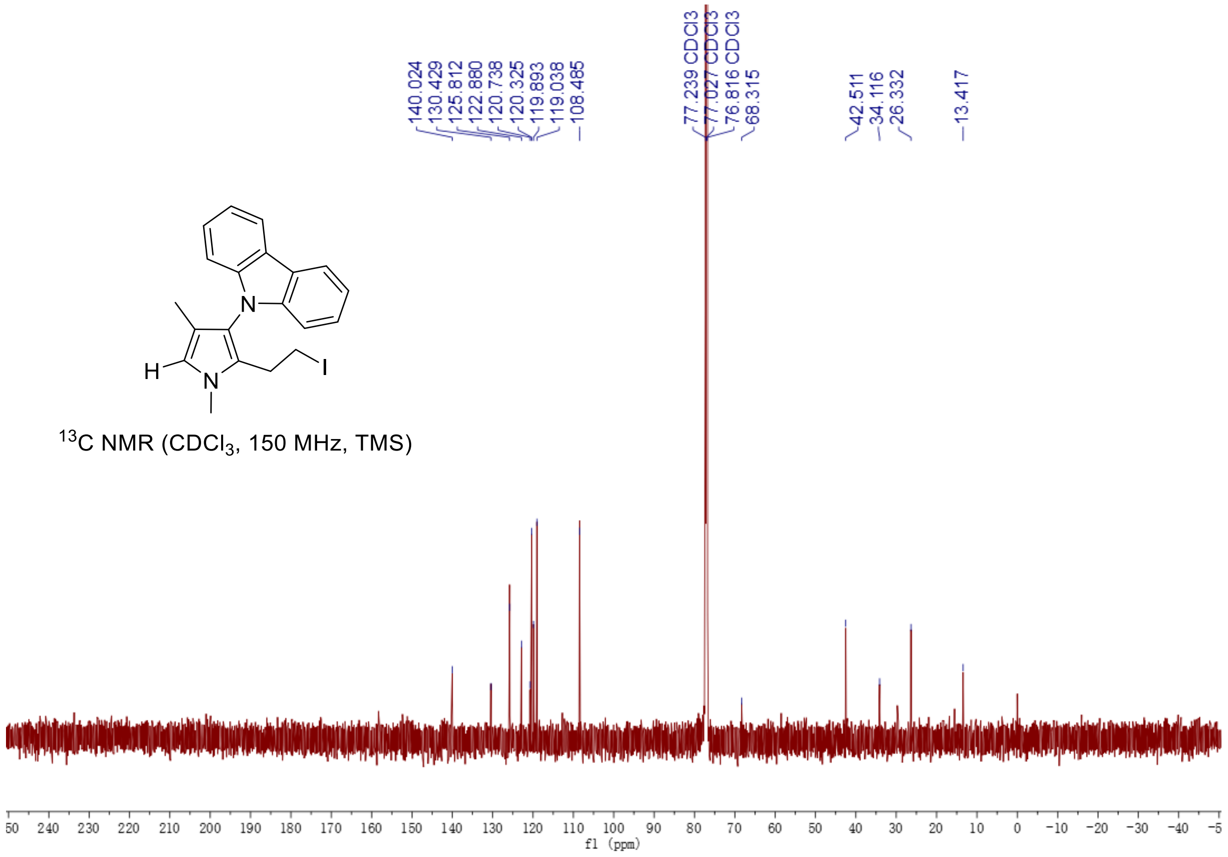

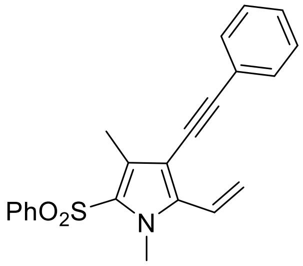

1,3-dimethyl-4-(phenylethynyl)-2-(phenylsulfonyl)-5-vinyl-1H-pyrrole (13)

A yellow oil, 23.1 mg, 64% yield. ^{1}H NMR ($CDCl_3$, TMS, 400 MHz) δ 7.85 (d, J = 7.0 Hz, 2H), 7.60 - 7.46 (m, 5H), 7.37 - 7.30 (m, 3H), 6.57 (dd, J = 17.5, 11.6 Hz, 1H), 6.38 (d, J = 17.5 Hz, 1H), 5.61 (d, J = 11.6 Hz, 1H), 3.71 (s, 3H), 2.57 (s, 3H). ^{13}C NMR ($CDCl_3$, TMS, 100 MHz) δ 143.0, 138.1, 133.0, 132.1, 131.2, 129.3, 128.4, 128.0, 126.4, 123.8, 123.6, 123.3, 120.8, 105.4, 94.8, 82.9, 32.7, 11.7. IR (neat) ν 754, 913, 1079, 1326, 2901, 2988 cm^{-1}. HRMS (EI) calcd. for $C_{22}H_{19}NO_2SNa$ (M+Na): 384.1034, Found: 384.1022.

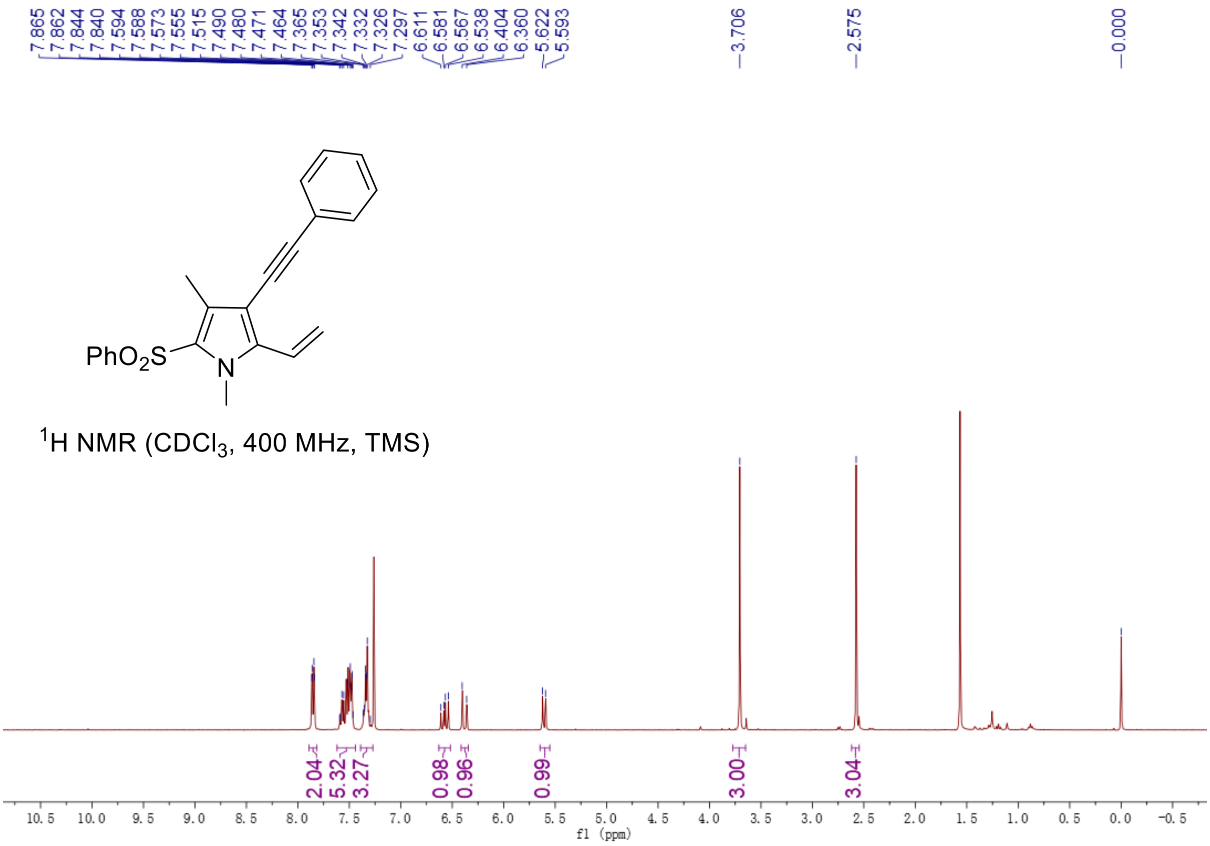
7.865
7.862
7.844
7.840
7.594
7.588
7.573
7.555
7.515
7.490
7.480
7.471
7.464
7.365
7.353
7.342
7.332
7.326
7.297
6.611
6.581
6.567
6.538
6.404
6.360
5.622
5.593
3.706
2.575
0.000
PhO2S
N
1H NMR (CDCl3, 400 MHz, TMS)
2.04
5.32
3.27
0.98
0.96
0.99
3.00
3.04
f1 (ppm)

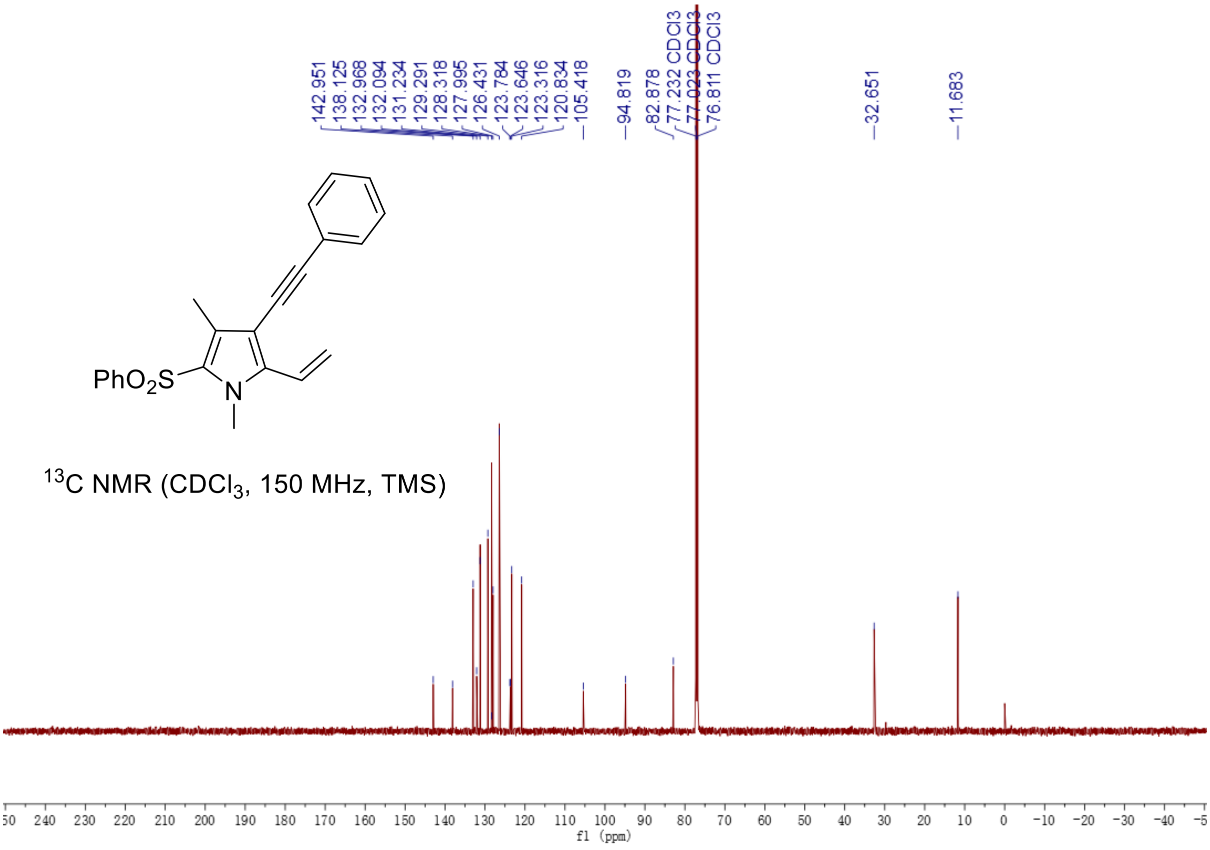
142.951
138.125
132.968
132.094
131.234
129.291
128.318
127.995
126.431
123.784
123.646
123.316
120.834
105.418
94.819
82.878
77.232 CDCl3
77.023 CDCl3
76.811 CDCl3
32.651
11.683
PhO2S
N
13C NMR (CDCl3, 150 MHz, TMS)
f1 (ppm)

10. Computational studies

All quantum mechanical calculations have been performed with Gaussian 16. The geometries of all species have been optimized at B3LYP/6-31G(d) level. The subsequent frequency calculations on the stationary points were carried out at the same level of theory to ascertain the nature of the stationary points as minima on the respective potential energy surfaces. The conformational space of flexible systems has first been searched manually and checked by xtb 6.0 program. Thermochemical corrections to 298.15 K have been calculated for all minima from unscaled vibrational frequencies obtained at this same level. The thermochemical corrections have been combined with single-point energies calculated at the SMD/B3LYP/6-311+G(d,p)// B3LYP/6-31G(d) level to yield free energy G_{298} at 298.15 K. The solvent effect was estimated by the IEFPCM method with radii and nonelectrostatic terms for SMD salvation model in dichloromethane (ε = 8.93).

Table S7. The total energies, enthalpies and free energies of all species in dichloromethane shown in Schemes 7a, 7c.

	E_{tot}	H_{298}	G_{298}
**1a+NIS**	-1558.078019	-1557.652874	-1557.755107
**TS1**	-1558.055398	-1557.63175	-1557.726428
**INT1**	-1558.076812	-1557.650978	-1557.740193
**TS2**	-1558.051763	-1557.632819	-1557.731027
**TsH**	-820.2882496	-820.139865	-820.1866468
**INT2**	-737.825833	-737.551705	-737.623488
**I^-**	-11.563306	-11.560946	-11.580154
**INT3**	-749.396661	-749.12022	-749.201193
**TS3**	-749.3817925	-749.1067992	-749.1861772
**INT4**	-749.405308	-749.128639	-749.209718
**INT1'**	-1273.629836	-1273.294532	-1273.385194
**TS1'**	-1273.618541	-1273.284287	-1273.365089
**INT2'**	-1273.682114	-1273.343481	-1273.429288
**TS2'**	-1273.650194	-1273.313635	-1273.390803
**INT3'**	-1273.731928	-1273.390522	-1273.466922
**TS3'**	-1273.706467	-1273.372462	-1273.450496
**INT4'**	-1273.755657	-1273.415319	-1273.499759

Archive entries

1a+NIS

```
Opt @ B3LYP/6-31G(d)
SCF Done: E(B3LYP) = -1558.078019a.u.
Zero-point correction = 0.393975Hartree/Particle
Sum of electronic and thermal Free Energies = -1557.755107a.u.
------------------------------------------------------------------

 N 1.56217,1.362111,-0.046872
 C 2.705964,2.201257,-0.418152
 C 2.318108,3.638975,-0.742306
 S 1.8037,0.222429,1.170006
 O 2.646675,0.858896,2.184742
 O 0.468696,-0.293113,1.506987
 C 2.736396,-1.138038,0.460066
 C 4.133319,-1.102539,0.512313
 C 4.86347,-2.133077,-0.075795
 C 4.221957,-3.204073,-0.715396
 C 2.820439,-3.216667,-0.749224
 C 2.070018,-2.195473,-0.167203
 C 5.020665,-4.333934,-1.320895
 C 3.388104,4.439291,-1.455200
 C 1.163493,4.15861,-0.389059
 C 0.027586,4.673324,-0.055968
 C -1.389944,4.951193,-0.386216
 C -0.796536,5.371849,0.956405
 I -3.520698,-0.811767,-1.385803
 H 3.408205,2.209325,0.421191
 H 3.236635,1.76916,-1.284933
 H 4.63504,-0.290768,1.028161
 H 5.949661,-2.109055,-0.030966
 H 2.302169,-4.041182,-1.232416
 H 0.985981,-2.240413,-0.184135
 H 6.00462,-3.993591,-1.659984
 H 5.187276,-5.133917,-0.587247
 H 4.50017,-4.779938,-2.174702
 H 3.068029,5.471641,-1.615594
 H 4.320449,4.457056,-0.873075
 H 3.629035,3.996135,-2.431651
 H -1.607233,5.728803,-1.117173
 H -2.089061,4.116529,-0.398599
 H -1.093083,4.818293,1.845588
 H -0.614595,6.431855,1.127373
```

C 0.693918,0.953504,-1.156112
H -0.200481,0.472097,-0.762026
H 0.39534,1.856272,-1.692789
H 1.202203,0.271822,-1.855607
C -2.391798,-1.026737,2.815701
C -1.70839,-2.297121,2.285301
H -1.654095,-0.268685,3.097268
H -3.060656,-1.18723,3.665314
H -0.64162,-2.343316,2.507414
H -2.179659,-3.216216,2.652415
N -2.814844,-1.226639,0.51498
C -1.896075,-2.253647,0.773091
O -1.387921,-2.967394,-0.062769
C -3.187321,-0.466969,1.641063
O -3.969710,0.452043,1.637592

TS1

Opt @ B3LYP/6-31G(d)
SCF Done: E(B3LYP) = -1558.055398a.u.
Zero-point correction = 0.394176Hartree/Particle
Sum of electronic and thermal Free Energies = -1557.726428a.u.

C -1.775465,1.150147,0.207488
C -0.269438,1.41033,0.153173
I 2.86208,0.200859,0.012134
C -2.168754,-0.34,-1.715454
C 0.187566,2.836778,0.225166
C 0.46724,0.29817,0.100992
C -0.195221,-0.952746,0.089408
C -0.044289,-2.153823,0.957849
C 0.083229,-2.26544,-0.555325
N 5.16087,0.158109,-0.102746
C 5.948883,1.290206,-0.029584
C 7.426398,0.869848,-0.13969
C 7.384325,-0.655071,-0.291285
C 5.88564,-1.006872,-0.256882
O 5.429789,-2.134098,-0.352072
O 5.556527,2.437025,0.099687
N -1.99824,-0.253182,-0.243328
C -4.812228,-0.311077,0.218971
C -5.263206,0.755891,1.003926
C -6.462303,1.377239,0.66748
C -7.222007,0.948034,-0.432759
C -6.748925,-0.132095,-1.193089
C -5.551758,-0.769819,-0.875794

C -8.535876,1.611245,-0.765447
S -3.283504,-1.124225,0.646169
O -2.992921,-0.891198,2.057009
O -3.268758,-2.459209,0.048137
H -2.145676,1.250572,1.232776
H -2.343579,1.833401,-0.436152
H -3.064753,0.203627,-2.031931
H -2.238343,-1.384096,-2.015746
H -1.284895,0.11471,-2.166226
H -0.205574,3.427375,-0.614845
H -0.165443,3.32198,1.146649
H 1.277744,2.897244,0.207273
H -0.923671,-2.610144,1.399283
H 0.843635,-2.164539,1.584312
H 1.059809,-2.374425,-1.020006
H -0.729281,-2.793613,-1.045352
H 7.877181,1.386651,-0.993511
H 7.956806,1.21015,0.755958
H 7.89116,-1.193616,0.516459
H 7.810022,-1.017563,-1.232973
H -4.696412,1.074652,1.871926
H -6.819744,2.204714,1.274563
H -7.329839,-0.485045,-2.040924
H -5.20561,-1.622455,-1.449859
H -9.351219,1.173,-0.175086
H -8.512059,2.682477,-0.541085
H -8.792838,1.486699,-1.821749

INT1

Opt @ B3LYP/6-31G(d)
SCF Done: E(B3LYP) = -1558.076812a.u.
Zero-point correction = 0.396900Hartree/Particle
Sum of electronic and thermal Free Energies = -1557.740193a.u.

N -3.199615,-1.160023,-0.011127
C -3.11547,-1.097837,1.495715
C -1.634882,-0.972132,1.77072
C -0.920743,-1.275981,0.675584
C -1.836775,-1.664391,-0.438164
C -4.389726,-1.914521,-0.489923
C -1.463441,-1.756973,-1.897235
C -1.69645,-3.020242,-1.119494
C -1.164245,-0.556581,3.129138
I 1.243356,-1.017502,0.353173

N 3.64598,-0.358098,-0.173803
C 4.75565,-1.15877,0.002138
C 6.03153,-0.391727,-0.411092
C 5.509091,0.978026,-0.848983
C 3.982404,0.868081,-0.655957
O 3.208958,1.79828,-0.920357
O 4.752526,-2.307378,0.420335
C -2.135903,1.698942,-0.347784
C -2.156212,2.490253,0.811585
C -1.032801,3.253717,1.101707
C 0.110948,3.216647,0.28333
C 0.09479,2.415544,-0.873068
C -1.025704,1.666299,-1.205177
C 1.345429,4.006391,0.622731
S -3.540893,0.717098,-0.71337
O -4.683361,1.094225,0.11946
O -3.67079,0.45069,-2.143124
H -3.550627,-2.02745,1.890615
H -3.73569,-0.269352,1.853372
H -5.286441,-1.411372,-0.123993
H -4.346093,-2.92917,-0.090676
H -4.400403,-1.933216,-1.579582
H -0.443019,-1.476824,-2.141273
H -2.206021,-1.47943,-2.637673
H -2.581004,-3.604338,-1.352664
H -0.839462,-3.603714,-0.796838
H -1.523097,0.45048,3.385538
H -1.537306,-1.236122,3.908241
H -0.073072,-0.552202,3.175305
H 6.541056,-0.946832,-1.206273
H 6.718485,-0.354049,0.441326
H 5.887032,1.811663,-0.246184
H 5.720936,1.222798,-1.89589
H -3.033575,2.516181,1.447801
H -1.033952,3.880002,1.989689
H 1.002139,2.330268,-1.463057
H -1.034864,1.0532,-2.098114
H 1.367843,4.943339,0.049025
H 1.376475,4.268056,1.685116
H 2.238922,3.433969,0.351303

TS2

Opt @ B3LYP/6-31G(d)
SCF Done: E(B3LYP) = -1558.051763a.u.
Zero-point correction = 0.388545Hartree/Particle

Sum of electronic and thermal Free Energies = -1557.731027a.u.

N -1.160879,-3.678937,0.294615
C -1.669682,-2.938223,1.401466
C -0.778616,-1.794314,1.597353
C 0.177122,-1.780599,0.574926
C -0.095647,-2.924372,-0.269937
C -2.02071,-4.57738,-0.456091
C 0.138839,-2.85006,-1.822618
C 1.063344,-3.663762,-1.035835
C -0.883832,-0.863637,2.760824
I 1.892046,-0.361037,0.34998
S -2.551461,-0.559878,-0.438058
O -3.606507,-1.438431,0.27084
H -2.68035,-2.399248,1.052922
C -3.312901,1.082352,-0.391415
O -2.263707,-0.89481,-1.869285
C -4.172991,1.437339,0.647795
C -4.675658,2.736808,0.686401
C -4.319695,3.685675,-0.283688
C -3.449193,3.294818,-1.311232
C -2.932705,2.001452,-1.369385
C -4.84006,5.101286,-0.209119
H -1.979992,-3.543691,2.262963
H -2.61098,-5.169436,0.248768
H -2.705868,-4.030188,-1.117592
H -1.415618,-5.267116,-1.051828
H 0.445278,-1.867019,-2.162389
H -0.638226,-3.324229,-2.412114
H 0.958865,-4.744932,-1.038975
H 2.052473,-3.278314,-0.807226
H -1.928727,-0.700195,3.046486
H -0.369995,-1.291746,3.632844
H -0.413543,0.097488,2.540792
H -4.465283,0.70356,1.390611
H -5.361826,3.016811,1.481999
H -3.168975,4.012268,-2.078412
H -2.264317,1.701694,-2.169346
H -4.143183,5.745864,0.34278
H -4.963563,5.536009,-1.206447
H -5.805201,5.14777,0.305589
N 3.69488,1.182674,0.035046
C 3.802603,2.390021,0.690085
C 5.080105,3.115068,0.220748

C 5.694466,2.166836,-0.814216
C 4.724804,0.969024,-0.854553
O 4.863831,-0.0124,-1.570975
O 3.018665,2.832486,1.517333
H 5.722706,3.299099,1.08852
H 4.804048,4.094687,-0.184095
H 5.769711,2.596525,-1.818993
H 6.693671,1.80646,-0.546834

TsH

Opt @ B3LYP/6-31G(d)
SCF Done: E(B3LYP) = -820.2882496a.u.
Zero-point correction = 0.138006Hartree/Particle
Sum of electronic and thermal Free Energies = -820.1866468a.u.

S -2.141367,0.197988 ,0.500172
O -2.612191,-1.187909,0.208215
O -2.488909,1.229288,-0.788787
C -0.339472,0.149202,0.252693
C 0.290668,-1.085068,0.113768
C 1.678149,-1.129676,-0.029054
C 2.445678,0.042082,-0.019263
C 1.785443,1.272830,0.128408
C 0.401716,1.3343790,0.268831
C 3.950229,-0.012591,-0.139602
H -0.310356,-1.989456,0.107040
H 2.172735,-2.090810,-0.148324
H 2.364784,2.193303,0.130143
H -0.099076,2.293521,0.369627
H 4.283326,-0.958310,-0.578307
H 4.332825,0.804350,-0.761210
H 4.428078,0.079359,0.844678
H -2.143793,0.824268,-1.610045

INT2

Opt @ B3LYP/6-31G(d)
SCF Done: E(B3LYP) = -737.825833a.u.
Zero-point correction = 0.254550Hartree/Particle
Sum of electronic and thermal Free Energies = -737.623488a.u.

N -4.135332,-0.182618,0.000134
C -3.999965,1.14504,-0.000487

C -2.647203,1.546584,-0.000726
C -1.876441,0.389026,-0.000142
C -2.80193,-0.742607,0.000389
C -5.38651,-0.92106,0.000765
C -2.504512,-2.059586,-0.738274
C -2.504526,-2.058858,0.740367
C -2.166338,2.9698,-0.001723
I 0.345355,0.201429,-0.000118
H -4.882103,1.778292,-0.000671
H -6.215794,-0.211283,0.000272
H -5.466494,-1.552404,-0.890349
H -5.46641,-1.551076,0.892827
H -3.339159,-2.515676,-1.26273
H -1.554093,-2.077606,-1.26242
H -1.554125,-2.076346,1.264559
H -3.339184,-2.514409,1.265274
H -3.002945,3.676309,0.004288
H -1.540559,3.172,0.874079
H -1.551275,3.174614,-0.884556
N 2.737905,-0.067521,-0.000103
C 3.644596,0.968465,0.001132
C 5.077885,0.39683,0.001011
C 4.877551,-1.121743,-0.000582
C 3.345471,-1.301059,-0.001061
O 2.77698,-2.385607,-0.00214
O 3.37695,2.161687,0.002185
H 5.610442,0.772706,0.881426
H 5.611058,0.774501,-0.87826
H 5.294591,-1.623081,-0.880878
H 5.294303,-1.624937,0.878788

I⁻

Opt @ B3LYP/6-31G(d)
SCF Done: E(B3LYP) = -11.563306a.u.
Zero-point correction = 0.000000Hartree/Particle
Sum of electronic and thermal Free Energies = -11.580154a.u.

I 0.000000,0.000000,0.000000

INT3

Opt @ B3LYP/6-31G(d)
SCF Done: E(B3LYP) = -749.396661a.u.
Zero-point correction = 0.254538Hartree/Particle
Sum of electronic and thermal Free Energies = -749.201193a.u.

N 2.161431,-1.783219,-0.674182
C 1.56355,-2.896618,-0.27006
C 0.1967,-2.689989,0.037672
C -0.067183,-1.351583,-0.203494
C 1.181153,-0.727822,-0.633229
C 3.544648,-1.665926,-1.125315
C 1.267621,0.475638,-1.587829
C 1.589788,0.700511,-0.168782
C -0.750148,-3.750171,0.522162
I -2.005923,-0.372404,-0.034588
H 2.125367,-3.823346,-0.213595
H 4.039878,-2.624889,-0.958136
H 3.569122,-1.432418,-2.195284
H 4.084694,-0.878611,-0.57336
H 2.091481,0.455778,-2.295795
H 0.310041,0.801837,-1.983294
H 0.847164,1.172942,0.466465
H 2.635507,0.83319,0.122112
H -0.251661,-4.722689,0.60108
H -1.158837,-3.495539,1.506355
H -1.604308,-3.857176,-0.155809
N -4.30726,0.712573,0.104976
C -5.353642,0.208402,0.833052
C -6.595878,1.120773,0.684244
C -6.123553,2.234379,-0.25082
C -4.651528,1.863801,-0.555759
O -3.929805,2.529809,-1.290539
O -5.348643,-0.812305,1.51381
H -6.900504,1.471929,1.676934

H -7.428506,0.528315,0.287571
H -6.679889,2.291062,-1.193597
H -6.153555,3.23462,0.196404
I 5.494852,1.17752,0.472685

TS3

Opt @ B3LYP/6-31G(d)
SCF Done: E(B3LYP) = -749.396661a.u.
Zero-point correction = 0.253259Hartree/Particle
Sum of electronic and thermal Free Energies = -749.201046a.u.

N -2.147824,2.685058,0.45645
C -1.36345,3.680128,-0.01917
C -0.072775,3.214108,-0.273661
C -0.051439,1.849743,0.076451
C -1.361281,1.509917,0.521312
C -3.517148,2.824838,0.920965
C -2.168696,0.089272,-0.233726
C -1.868858,0.25436,1.183314
C 1.060802,4.031709,-0.821029
I 1.695908,0.555248,0.028847
H -1.771132,4.674106,-0.163085
H -3.911641,3.780229,0.56733
H -4.147128,2.020975,0.527743
H -3.569115,2.804663,2.016345
H -3.095072,0.440019,-0.659238
H -1.483567,-0.445281,-0.879321
H -1.079929,-0.373422,1.591703
H -2.713941,0.380624,1.856282
H 0.751521,5.06773,-1.00093
H 1.910931,4.044633,-0.129135
H 1.431336,3.616502,-1.765287
N 3.831963,-0.905576,0.02875
C 4.726268,-0.964894,-1.00718
C 5.882059,-1.939074,-0.665071
C 5.536938,-2.436444,0.73809
C 4.214849,-1.701986,1.076037
O 3.629158,-1.841981,2.145373
O 4.664211,-0.352093,-2.069151
H 6.834341,-1.39912,-0.725962
H 5.916646,-2.730427,-1.422962
H 5.36861,-3.517966,0.799045
H 6.287865,-2.188445,1.497424
I -4.544385,-1.942958,-0.313642\

INT4

Opt @ B3LYP/6-31G(d)
SCF Done: E(B3LYP) = -749.405308a.u.
Zero-point correction = 0.254642Hartree/Particle
Sum of electronic and thermal Free Energies = -749.209718a.u.

N -2.519047,2.741229,0.299388
C -1.751681,3.838537,-0.02659
C -0.432671,3.45261,-0.149418
C -0.389929,2.050428,0.12599
C -1.680832,1.635602,0.413945
C -3.928458,2.78889,0.624378
C -2.568055,-0.4336,-0.577651
C -2.209638,0.268786,0.729846
C 0.721708,4.339947,-0.510163
I 1.374892,0.811054,0.092023
H -2.210148,4.812763,-0.142622
H -4.325977,3.761443,0.322091
H -4.487722,2.012434,0.08999
H -4.108639,2.661999,1.70061
H -3.278394,0.124816,-1.186527
H -1.686936,-0.691811,-1.16253
H -1.441456,-0.307214,1.25354
H -3.086412,0.318564,1.385919
H 0.389256,5.371384,-0.680117
H 1.480795,4.355063,0.281905
H 1.229064,3.992025,-1.418404
N 3.655202,-0.761854,0.048723
C 4.58825,-0.745118,-0.949889
C 5.740419,-1.744026,-0.648525
C 5.343243,-2.354195,0.692674
C 4.000665,-1.647778,1.030882
O 3.376372,-1.894242,2.061842
O 4.573405,-0.05658,-1.969561
H 6.691695,-1.198176,-0.628908
H 5.808553,-2.469549,-1.468405
H 5.179176,-3.438261,0.661169
H 6.060505,-2.165757,1.500925
I -3.608152,-2.397644,-0.247052

INT1'

Opt @ B3LYP/6-31G(d)
SCF Done: E(B3LYP) = -1273.629836a.u.

Zero-point correction = 0.310225Hartree/Particle
Sum of electronic and thermal Free Energies = -1273.385194a.u.

S 4.058511,2.891923,1.357998
C 3.748336,1.073729,1.33139
C 2.683948,0.685956,0.324034
C 1.59868,2.373319,-1.342697
C 0.528565,3.35288,-1.64772
C 1.618167,3.035157,-2.667569
C 2.297368,-0.79117,0.322113
C 2.140166,1.541714,-0.516067
H 5.024257,2.836171,2.301252
H 4.68237,0.552054,1.094899
H 3.436056,0.748254,2.3317
H -0.476603,2.971634,-1.818622
H 0.571623,4.317758,-1.144282
H 2.386931,3.783418,-2.852443
H 1.347133,2.442496,-3.539794
H 1.932768,-1.072167,1.320883
H 1.459505,-0.928911,-0.371367
N -3.075612,0.076923,0.539203
C -2.326196,1.142278,0.019886
C -1.423266,1.637063,1.146653
C -1.760226,0.761507,2.36541
C -2.820044,-0.22952,1.887577
I -4.455894,-0.968522,-0.593475
O -3.349694,-1.108527,2.522374
O -2.40038,1.565701,-1.110935
H -0.381781,1.547867,0.821305
H -1.619143,2.701773,1.306039
H -2.173293,1.326538,3.206902
H -0.904123,0.196924,2.747065
C 3.438197,-1.724943,-0.059451
C 3.844037,-2.754269,0.797946
C 4.097213,-1.577876,-1.288858
C 4.878552,-3.621916,0.438006
H 3.342647,-2.881939,1.75513
C 5.130331,-2.441311,-1.651428
H 3.798438,-0.776292,-1.959946
C 5.524928,-3.467718,-0.7885
H 5.177926,-4.41582,1.117379
H 5.628688,-2.313574,-2.608928
H 6.330155,-4.140439,-1.071266

TS1'

Opt @ B3LYP/6-31G(d)

SCF Done: E(B3LYP) = -1273.618541a.u.

Zero-point correction = 0.311345Hartree/Particle

Sum of electronic and thermal Free Energies = -1273.365089a.u.

C -3.338183,1.4052,-0.799831
C -2.026389,0.610891,-0.742219
C -2.130917,-0.818635,-1.229001
C -0.942627,1.254811,-0.305355
C -1.035658,2.622449,0.108419
C -0.317625,3.380886,1.189236
C -0.163048,3.793564,-0.256996
I 1.265814,0.304408,-0.163423
H -4.17321,0.891765,-0.313157
H -3.644084,1.644748,-1.826036
H -1.125016,-1.249369,-1.247706
H -2.499307,-0.828848,-2.265176
H -0.888187,4.037046,1.842392
H 0.497481,2.850586,1.67372
H 0.759734,3.550449,-0.775254
H -0.63778,4.718608,-0.572306
C 5.138102,-2.193718,-0.208159
C 5.655338,-0.924681,0.479814
H 5.217357,-3.094625,0.409278
H 5.634387,-2.413653,-1.159226
H 6.030425,-1.094133,1.494663
H 6.451092,-0.415076,-0.073672
N 3.351011,-0.642678,-0.016111
C 3.649733,-1.909386,-0.480916
O 2.875268,-2.68186,-1.017839
C 4.429982,0.004084,0.553388
O 4.426495,1.12349,1.037449
S -3.073874,3.010893,0.041087
H -3.29954,2.585819,1.30305
C -3.053671,-1.670233,-0.367774
C -4.26587,-2.161692,-0.867702
C -2.70454,-1.968125,0.958322
C -5.111482,-2.934933,-0.067034
H -4.545201,-1.949667,-1.898015
C -3.544748,-2.741392,1.758826
H -1.762598,-1.598352,1.355971
C -4.753109,-3.226046,1.249274
H -6.0454,-3.312489,-0.475382

H -3.253387,-2.972237,2.78022
H -5.405822,-3.831058,1.872826

INT2'

Opt @ B3LYP/6-31G(d)
SCF Done: E(B3LYP) = -1273.682114a.u.
Zero-point correction = 0.315190Hartree/Particle
Sum of electronic and thermal Free Energies = -1273.429288a.u.

S -4.157832,-2.553241,0.669711
C -3.655532,-0.861476,1.214307
C -2.455525,-0.478478,0.37643
C -1.778297,0.850268,0.621741
C -2.095552,-1.367021,-0.558912
C -2.90108,-2.607006,-0.666705
C -3.233232,-3.212782,-2.015488
C -2.286416,-3.92663,-1.082188
H -3.414899,-0.877598,2.284925
H -4.483024,-0.15619,1.074449
H -1.426784,0.895882,1.66283
H -0.886971,0.910392,-0.015198
H -1.251525,-1.210167,-1.227424
H -2.827886,-2.727943,-2.900857
H -4.230489,-3.624282,-2.146321
H -2.639604,-4.824888,-0.582132
H -1.225723,-3.938346,-1.325059
N 3.053622,-0.51174,0.214698
C 1.839398,-0.99116,-0.290277
C 1.476167,-2.227446,0.528268
C 2.582864,-2.365827,1.587005
C 3.576001,-1.240946,1.299703
I 4.003997,1.15362,-0.562931
O 4.607137,-0.999508,1.876823
O 1.214307,-0.501848,-1.204764
H 0.47375,-2.086736,0.943823
H 1.422032,-3.083575,-0.152082
H 3.115498,-3.320506,1.545101
H 2.217629,-2.246024,2.611929
C -2.67664,2.054151,0.365731
C -2.92607,2.995352,1.371214
C -3.263778,2.248186,-0.892957
C -3.736916,4.107088,1.128667
H -2.478166,2.859477,2.353472
C -4.073712,3.356292,-1.139407

H -3.085592,1.520293,-1.680819
C -4.313402,4.290956,-0.128311
H -3.917588,4.826822,1.92298
H -4.518752,3.491012,-2.121948
H -4.944991,5.154285,-0.320013

TS2'

Opt @ B3LYP/6-31G(d)
SCF Done: E(B3LYP) = -1273.33181495a.u.
Zero-point correction = 0.314033Hartree/Particle
Sum of electronic and thermal Free Energies = -1273.072436a.u.

S -3.7821313398,-3.1321385918,0.2007489455
C -2.6549494554,-1.6839463302,0.1403372394
C -2.6278061279,-1.2337504719,-1.2895786011
C -1.7574325946,-0.0721223616,-1.6860181621
C -3.3228525619,-2.0290669942,-2.1380812123
C -3.9280838278,-3.183030819,-1.5298553792
C -4.6696715269,-4.2317417268,-2.2342616703
C -3.3584204707,-4.9791560192,-2.4569034321
H -1.640536306,-1.9676169208,0.4522965536
H -3.0320005551,-0.9108249647,0.817049265
H -0.7278900588,-0.4569581879,-1.6655761491
H -1.9822477186,0.2083929004,-2.7215983932
H -3.3656963596,-1.8798896228,-3.2124330752
H -5.1439444151,-3.876548856,-3.152606985
H -5.3983562034,-4.7649462356,-1.6195595145
H -3.3072769145,-5.9018536416,-1.8852308909
H -3.0999380194,-5.0836486786,-3.5073936937
N 1.1997546174,-3.8504218482,-1.142380932
C 1.451601399,-2.6986064284,-0.4481272778
C 2.956129827,-2.598919593,-0.1628674018
C 3.5504791593,-3.8671991516,-0.7876198661
C 2.3442034598,-4.6018457392,-1.3961516348
I -0.911950104,-4.4221863548,-1.8000636364
O 2.3860170729,-5.655762881,-1.996173186
O 0.6241001686,-1.8584846053,-0.0959910234
H 3.3379319852,-1.6703397899,-0.6003854692
H 3.1055650122,-2.5268582738,0.9195625674
H 4.0364271371,-4.5281333749,-0.0627514329
H 4.2793877395,-3.668700196,-1.5798209107
C -1.8483736426,1.1351847785,-0.7668281697
C -0.8934584582,1.3291984693,0.2407757653
C -2.8910743517,2.0613905667,-0.9028266609
C -0.982858415,2.4312472032,1.0944949002

H -0.0849789325,0.6101058165,0.3513436547
C -2.9790084102,3.1633167597,-0.0510076828
H -3.6354999512,1.9215387282,-1.6841334982
C -2.0238317174,3.3505534424,0.9510629937
H -0.2345515348,2.572696451,1.8699717676
H -3.7902671548,3.8765391052,-0.1716051047
H -2.0896716601,4.2096212474,1.6133797339

INT3’

Opt @ B3LYP/6-31G(d)
SCF Done: E(B3LYP) = -1273.42187615 a.u.
Zero-point correction = 0.319756 Hartree/Particle
Sum of electronic and thermal Free Energies = -1273.156870 a.u.

S -2.6154103326,-2.5804373944,0.8156167995
C -2.0152010275,-0.9493176506,1.4254627454
C -1.0867360424,-0.3557801379,0.3412308507
C -0.9931546241,1.2005199028,0.4339785933
C -1.6624129271,-0.8891336799,-0.9620420642
C -2.3878976509,-2.0109042225,-0.8603203565
C -3.0081551882,-2.7790178778,-1.9924064637
C -2.6173819064,-4.2524312965,-2.0800006131
H -1.5339792232,-1.0597442256,2.3929892679
H -2.8878412406,-0.2984792227,1.509301256
H -0.5512092934,1.4393010989,1.4086971767
H -0.2782876731,1.5166649814,-0.3329971114
H -1.4135878286,-0.4373370867,-1.9124638027
H -2.7877934641,-2.2735556869,-2.9381539946
H -4.1034265783,-2.7688772929,-1.8688866487
H -2.728419817,-4.7775751644,-1.1314835644
H -3.1798910652,-4.7697670402,-2.8561513235
N 0.3281726142,-0.8685355179,0.481300708
C 1.2232176773,-0.8429040645,-0.6033374955
C 2.5273165441,-1.5007626837,-0.1733230643
C 2.3802615383,-1.7104651468,1.335578352
C 0.9780247928,-1.2135687313,1.6760483721
I -0.5033564397,-4.5816306466,-2.6470730006
O 0.526408154,-1.1166957831,2.8004043718
O 0.9927160926,-0.3527889035,-1.689398387
H 2.6356230306,-2.4365720776,-0.731579492
H 3.3634855861,-0.8553409981,-0.4562466643
H 3.1023134173,-1.1436525225,1.9309718467
H 2.4597418918,-2.7574129769,1.6448045921
C -2.290155363,1.9627407723,0.2606014298
C -3.0322646496,2.3836532972,1.373702653

C -2.7696424369,2.286463746,-1.017348523
C -4.2256590224,3.0916127537,1.2174834506
H -2.6658707913,2.1627600194,2.3739455682
C -3.9613252281,2.993839104,-1.1784430974
H -2.197611816,1.9914184019,-1.8933198129
C -4.6957939747,3.3963762212,-0.0607313831
H -4.7826879673,3.409917524,2.094752361
H -4.3127461337,3.2371060513,-2.1777844987
H -5.6223392034,3.9502681574,-0.1854426229

TS3'

Opt @ B3LYP/6-31G(d)
SCF Done: E(B3LYP) = -1273.39066515 a.u.
Zero-point correction = 0.311719 Hartree/Particle
Sum of electronic and thermal Free Energies = -1273.134694 a.u.

S,0,1.0998705263,0.8744672144,2.9769269029
C -0.3331268693,1.6935007589,2.3737369128
C -1.2070443256,0.7804285833,1.7512683642
C -2.5540090419,1.2178732795,1.2170691232
C -0.6060712108,-0.4888976898,1.5951114379
C 0.6446471407,-0.5876919475,2.1788635331
C 1.6282429699,-1.7194818384,2.0691674523
C 2.5611767439,-1.6068461264,0.8544488819
H -0.6413209111,2.622762866,2.8433835774
H -3.1931982182,1.4625852471,2.0778379922
H -2.3993974158,2.1558497111,0.6662872228
H -1.0597799828,-1.2986031348,1.0371843083
H 1.0971520019,-2.6768755515,2.0596811647
H 2.2756106231,-1.7274051668,2.9574882712
H 2.8966030081,-0.5845898007,0.6743175651
H 3.4076563501,-2.2866100566,0.9449896039
N 0.7430292249,2.5678476693,-0.1741953343
C -0.039633498,3.1714927198,-1.1277847968
C 0.8261773611,3.4648350737,-2.3618544306
C 2.2040973726,2.88365134,-2.0090046156
C 2.0279470957,2.3078150016,-0.5955719694
I 1.6063411688,-2.2101556997,-1.0482163741
O 2.8833055508,1.7085676085,0.0407476101
O -1.233071642,3.4240415341,-1.0163088821
H 0.3640569054,3.0025008777,-3.2397981947
H 0.8367916182,4.5456548056,-2.5380294001
H 3.0059636822,3.6291206071,-1.9932400901
H 2.5262578497,2.0803143877,-2.679004432
C -3.2542353717,0.2162452755,0.3209508602

C -3.9851921469,-0.8506527072,0.861720484
C -3.1796539267,0.3471856952,-1.072224743
C -4.6218294971,-1.7732102548,0.029834009
H -4.0613507429,-0.9564014851,1.9422273626
C -3.8178951369,-0.5737275867,-1.9054436903
H -2.6294480397,1.1834802474,-1.4962228344
C -4.5381390326,-1.6370917296,-1.3576989346
H -5.1874717058,-2.5931903257,0.4647577507
H -3.7554997474,-0.4568560741,-2.9841992828
H -5.0361677328,-2.3522809262,-2.0069533383
H 0.2087390026,2.1723305978,1.0785849528

INT4'

Opt @ B3LYP/6-31G(d)
SCF Done: E(B3LYP) = -1273.43625870 a.u.
Zero-point correction = 0.317087 Hartree/Particle
Sum of electronic and thermal Free Energies = -1273.180361 a.u.

S -0.2581231778,-1.5056150896,-1.3421239879
C 0.979854784,-0.3449139768,-0.9627634331
C 1.1517793816,-0.1915832778,0.3903406121
C 2.2040990278,0.6988728041,1.0232786583
C 0.2506673313,-1.0254400486,1.1335257106
C -0.5710123732,-1.7936928536,0.3547272932
C -1.6225483764,-2.7668396965,0.805560693
C -3.0504164005,-2.4722488034,0.3473783145
H 1.5248048866,0.1248021386,-1.7716720506
H 3.070827166,0.0807077509,1.2951556458
H 2.5668355039,1.4075063442,0.267409486
H 0.210329325,-1.0330764161,2.2177291185
H -1.5887870287,-2.835954465,1.8978876886
H -1.3880272523,-3.7737536795,0.4234152932
H -3.1222690857,-2.3186990169,-0.7292984893
H -3.7395846561,-3.2588547112,0.6540139961
N -0.6310559268,2.3861128309,-1.9434681164
C -1.1747945337,3.5984340083,-1.5362244071
C -1.5921631233,4.3510463698,-2.8016094869
C -1.1792340142,3.439449622,-3.9717176721
C -0.5594388249,2.2016326384,-3.3196775584
I -3.8977009382,-0.6279862386,1.2226164485
O -0.0797359275,1.2333344721,-3.8716765285
O -1.2780688296,3.9690722867,-0.3859223489
H -2.6695417965,4.5390212173,-2.7569123188
H -1.0975507283,5.3270492719,-2.8111304518
H -0.4399559571,3.8936745934,-4.6387411698

H -2.0205776866,3.1229077985,-4.596060236
C 1.7396262871,1.4513715645,2.260211852
C 2.3028117453,1.1720062325,3.5115932558
C 0.7449065865,2.43727554,2.1777327593
C 1.8883628105,1.8584055838,4.6559137448
H 3.0775452802,0.4119915197,3.5910228807
C 0.3301029094,3.1245371396,3.3182213008
H 0.2823591294,2.6793429966,1.2241214033
C 0.8996850005,2.8379504578,4.5617925921
H 2.3397998424,1.6269001166,5.6173360344
H -0.4406923357,3.885390334,3.2303477369
H 0.5748669361,3.3747142595,5.4492849453
H -0.2967551304,1.6801910319,-1.2902140585

11. X-Ray structures

(a) **3ga**

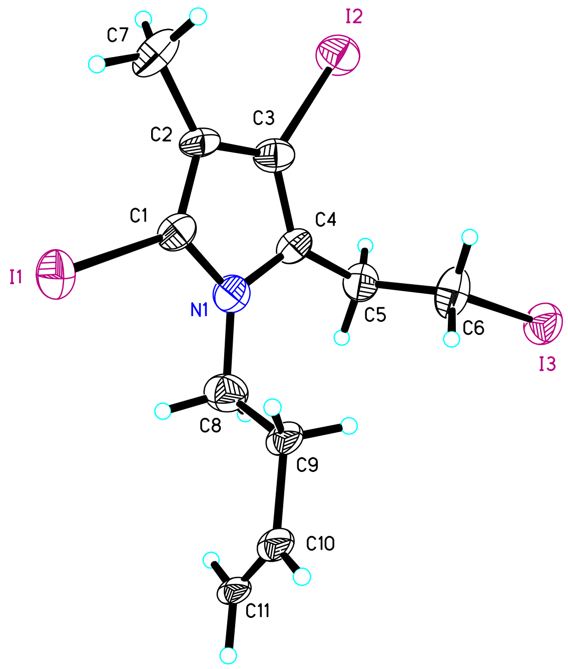

The crystal data of **3ga** have been deposited in CCDC with number 2083431. Empirical Formula: $C_{11}H_{14}I_3N$; Formula Weight: 540.93; Crystal Color, Habit: colorless, Crystal Dimensions: 0.170 x 0.140 x 0.060 mm^3; Crystal System: Triclinic; Lattice Parameters: a = 8.6649(8) Å, b = 8.8569(8)Å, c = 9.8661(9) Å, α = 84.893(3)°, β = 88.222(3)°, γ = 84.477(3)°, V = 750.47(12) Å^3; Space group: P -1; Z = 2; D_{calc} = 2.394 g/cm^3; F_{000} = 492; Final R indices [I>2sigma(I)] R1 = 0.0798, wR2 = 0.2460.

(b) **3oa’**

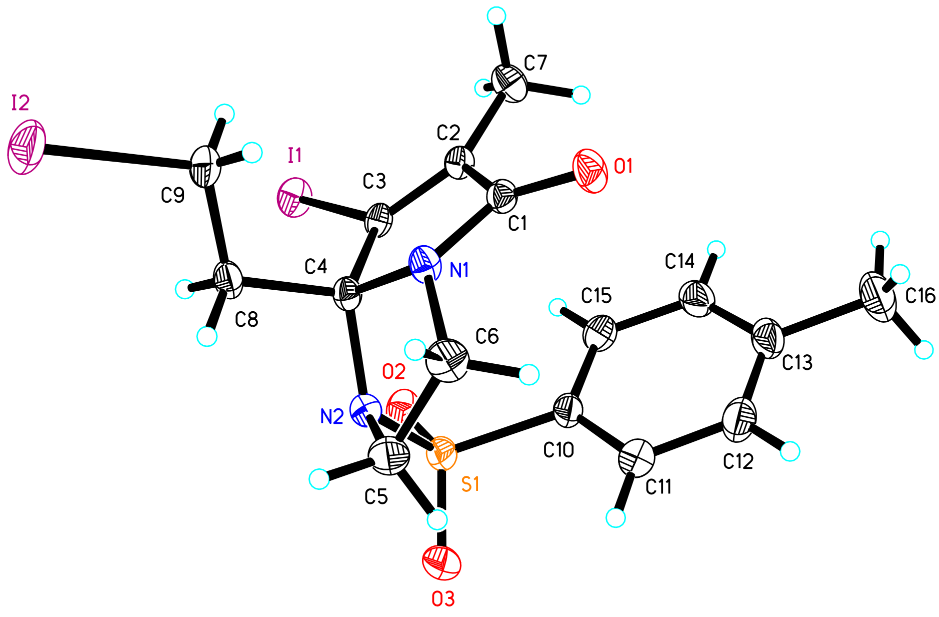

The crystal data of **3oa’** have been deposited in CCDC with number 2142800. Empirical Formula: $C_{16}H_{18}I_2N_2O_3S$; Formula Weight: 572.18; Crystal Color, Habit: colorless, Crystal Dimensions: 0.200 x 0.150 x 0.120 mm3; Crystal System: Monoclinic; Lattice Parameters: a = 11.5550(4) Å, b = 8.3352(2) Å, c = 20.4748(6) Å, α = 90°, β = 105.3480(10)°, γ = 90°, V = 1901.66(10) Å^3; Space group: P 21/c; Z = 4; Dcalc = 1.999 g/cm^3; F000 = 1096; Final R indices [I>2sigma(I)] R1 = 0.0245, wR2 = 0.0571.

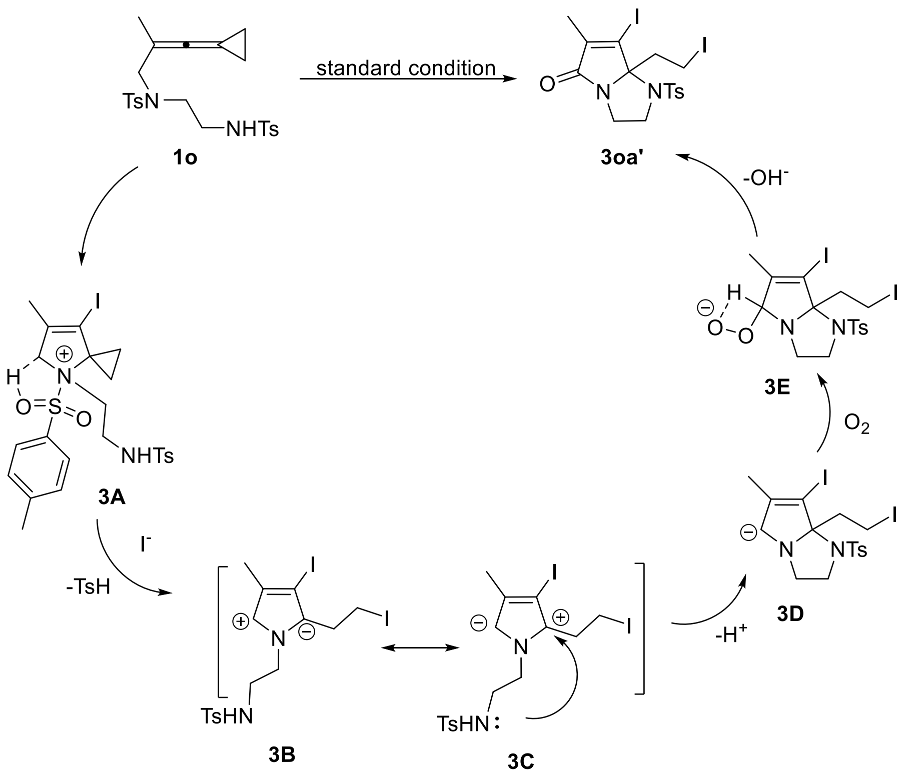

Figure S5. Proposed reaction mechanism of **3oa’**

According to Scheme 7a in the main text, intermediate **3A** is firstly generated through the cyclization reaction. The Ts moiety in intermediate **3A** promotes subsequent dehydrogenation to afford intermediate **3B**. The NHTs moiety in intermediate **3C**, which is the resonance structure of intermediate **3B** undergoes the intramolecular nucleophilic attack to give the cyclized intermediate **3D**. Intermediate **3D** reacts with molecular oxygen under ambient atmosphere to obtain intermediate **3E**. Finally the hydroxide anion in intermediate **3E** is released to deliver the corresponding product **3oa'**. This result indicates that the zwitterionic intermediate **1D** shown in Scheme 7b might be indeed involved in the reaction system.

12. References

[1] K. H. Rui, S. Yang, Y. Wei, M. Shi, *Org. Chem. Front.*, **2019**, *6*, 2506-2513.

[2] M. Z. Miao, X. Xu, L. J. Xu, H. J. Ren, *Eur. J. Org. Chem.*, **2014**, *27*, 5896-5900.

[3] N. Miyaura, K. J. Yamada, A. Suzuki, *Tetrahedron Lett.* **1979**, *20*, 3437-3440.

[4] E. C. Taylor, W. B. Young, *J. Org. Chem.*, **1995**, *24*, 7947-7952.

[5] E. R. Wearing, D. E. Blackmun, M. R. Becker, C. S. Schindler, *J. Am. Chem. Soc.* **2021**, *39*, 16235-16242.

[6] L. L. Liao, Z. H. Wang, K. G. Cao, G. Q. Sun, W. Zhang, C. K. Ran, Y. W. Li, L. Chen, G. M. Cao, D. G. Yu, *J. Am. Chem. Soc.*, **2022**, *144*, 2062-2068.

[7] W. Yuan, X. Dong, M. Shi, P. McDowell, G. G. Li, *Org. Lett.*, **2012**, *14*, 5582-5585.

[8] K. H. Rui, M. Shi, *Org. Chem. Front.*, **2019**, *6*, 1816-1820.

[9] Z. Meng, X. Y. Zhang, M. Shi, *Org. Chem. Front.*, **2021**, *8*, 3796-3801.

[10] S. Yang, K. H. Rui, X. Y. Tang, Q. Xu, M. Shi, *J. Am. Chem. Soc.*, **2017**, *16*, 5957-5964.

[11] C. Ning, K. H. Rui, Y. Wei, M. Shi, *Chem. Sci.*, **2022**, *13*, 7310-7317.
